# Stereoselective Synthesis of Highly Functionalized Bicyclo[2.1.0]pentanes by Sequential [2 + 1] and [2 + 2] Cycloadditions

**DOI:** 10.1021/acs.orglett.5c00054

**Published:** 2025-02-10

**Authors:** Brockton Keen, Christina Cong, Alberto Castanedo, Geraint H. M. Davies, Robert R. Knowles, Huw M. L. Davies

**Affiliations:** † Department of Chemistry, Emory University, 1515 Dickey Drive, Atlanta, Georgia 30322, United States; ‡ Department of Chemistry, Princeton University, Princeton, New Jersey 08544, United States; § PostEra, One Broadway, 14th Floor, Cambridge, Massachusetts 02142, United States

## Abstract

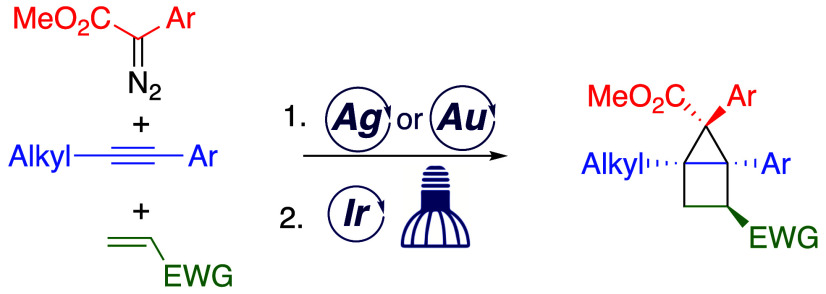

This study describes a method for the stereoselective synthesis of highly functionalized bicyclo[2.1.0]pentanes (housanes). The approach utilizes a two-step sequence, a silver- or gold-catalyzed cyclopropenation of alkynes followed by an intermolecular [2 + 2] photocycloaddition reaction with electron-deficient alkenes. The cyclopropenation is an established reaction of aryldiazoacetates. A regioselective [2 + 2] cycloaddition of the cyclopropane was developed using blue LED irradiation, a commercially available photocatalyst as a triplet-sensitizer, and low reaction temperature (−40 °C). The [2 + 2] cycloaddition is highly diastereoselective, and when enantioenriched cyclopropenes are used, it proceeds with enantioretention.

Interest in the synthesis of complex, strained aliphatic rings has increased in recent years.^1−3^ Many studies have focused on the synthesis and manipulation of bioisosteres, such as bicyclo[1.1.1]pentanes (BCPs),^4−7^ bicyclo[2.1.1]hexanes,^8^ and cubanes,^9^ which can be used in medicinal chemistry in place of phenyl rings with minimal loss in binding efficiency and increased metabolic stability. However, one challenging scaffold that has received less attention is bicyclo[2.1.0]pentanes, also known as “housanes”. Many of the traditional methods for the synthesis of housanes are not very direct.^10−16^ New methods for their synthesis continue to be examined, and a recent advance has been the intramolecular cyclopropanation of alkenes.^17,18^ A particularly attractive method is the [2 + 2] cycloaddition with cyclopropenes.^19−21^ In this study, we describe a two-step sequence to rapidly generate highly functionalized housanes (**5**) in a regioselective and stereoselective manner (Scheme 1A). Previously, the Davies group demonstrated that the cyclopropenation of mono- and disubstituted alkynes (**1**) with aryldiazoacetates (**2**) readily generates cyclopropenes (**3**) (Scheme 1B). In the case of monosubstituted alkynes, dirhodium catalysts Rh_2_(*S*-DOSP)_4_ result in cyclopropenation with high levels of asymmetric induction, but this catalyst system fails to cyclopropenate disubstituted alkynes.^22^ Instead, silver triflate was found to be an effective alternative catalyst for the reaction with 1,2-disubstituted alkynes.^23^ Later, chiral gold catalysts were developed for the enantioselective variant.^24^ The second step is a visible-light-photosensitized [2 + 2] cycloaddition between the cyclopropene **3** and a suitable alkene **4** to form the housane **5**.

**Scheme 1:**
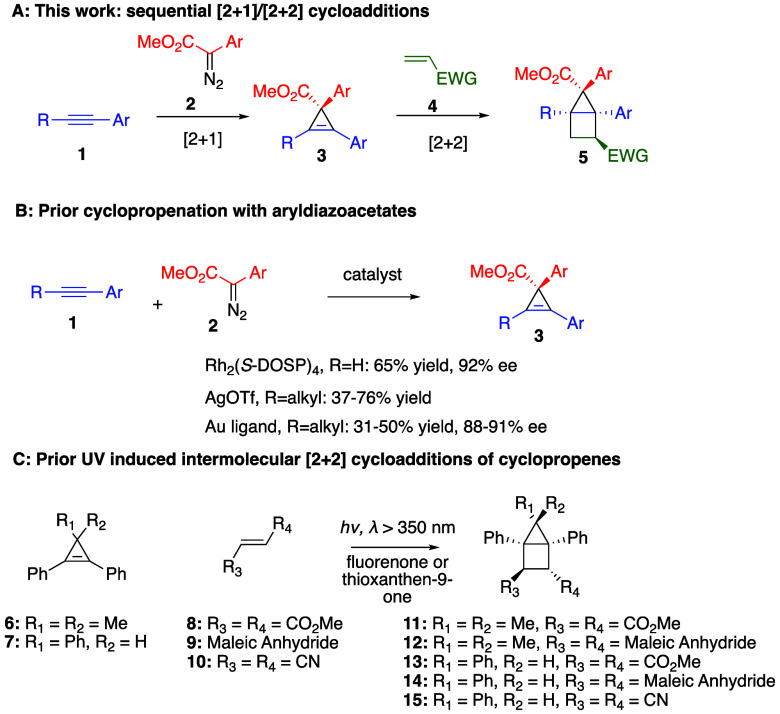
Summary of Current and Prior Work

Photodimerization of cyclopropene was first reported in 1964 using 1,3,3-trimethylcyclopropene, creating the tricyclo[3.1.0.0^2,4^]hexane.^25^ The use of cyclopropene in UV-promoted intermolecular [2 + 2] cycloadditions was first reported in 1977 by Arnold using 3,3-dimethyl-1,2-triphenylcyclopropene (**6**) and dimethylfumarate (**8**) to form the housane **11**.^26^ The same paper also discloses the [2 + 2] product between **6** and maleic anhydride (**9**), as well as the triphenylcyclopropene (**7**) with the same olefins. In a later paper, Arnold also described the reaction between **7** and fumaronitrile (**10**) (Scheme 1C).^27^ Other examples of UV-induced intermolecular [2 + 2] cycloadditions were published by Farid, Padwa, and de Meijere.^28−30^ Additionally, intramolecular [2 + 2] cycloadditions involving cyclopropenes were studied extensively by Padwa.^31^

In 2012, Yoon demonstrated the use of visible-light-absorbing organometallic chromophores as triplet sensitizers for [2 + 2] cycloadditions, eliminating the need for high-energy UV light.^32^ Since then, many methods have been reported which utilize these mild conditions to facilitate efficient cross-selective intermolecular [2 + 2] cycloadditions.^33−38^ Building on these approaches, we proposed that cyclopropenation followed by energy-transfer-based [2 + 2] photocycloaddition of the resulting cyclopropene could be developed into an effective method for the rapid generation of housanes. During the course of preparing this manuscript, a report appeared conducting a combined cyclopropenation of alkynes followed by a photoinduced [2 + 2] cycloaddition.^39^ However, the alkenes used in the [2 + 2] cycloaddition were limited to *N*-substituted maleimides, and it was reported that other alkenes, such as the ones used in this study, were unsuccessful.

The key requirement needed to make this proposed two-step process a reality was to identify suitable conditions for the [2 + 2] cycloaddition reaction. Our study began with the test reaction, the photosensitization of the cyclopropene **16** (Table 1) using [Ru(bpy)_3_](PF_6_)_2_ ([**Ru-1**](PF_6_)_2_, *E*_*T*_*=* 46.5 kcal/mol^40^) in acetonitrile with 3 equiv of methyl acrylate (**17**) as an electron-deficient alkene and irradiation from a blue LED over 2 h. This resulted in the complete recovery of the starting material (entry 1), indicating that the photocatalyst did not have a high enough triplet energy (*E*_*T*_) to sensitize the cyclopropene **16**. The same result was obtained when the experiment was repeated with [Ir(ppy)_2_(dtbbpy)]PF_6_ ([**Ir-1**]PF_6_, *E*_*T*_*=* 49.2 kcal/mol^40^) in place of [Ru(bpy)_3_](PF_6_)_2_ (entry 2).

**Table 1:**
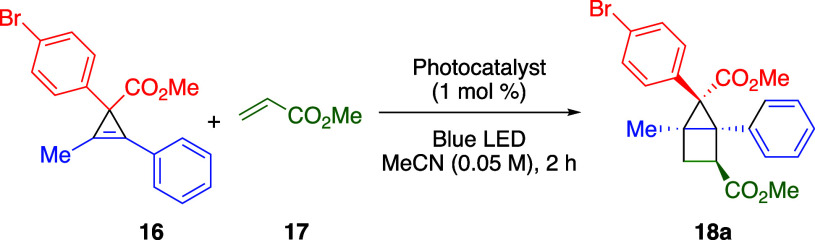
Optimization of the [2 + 2] Cycloaddition^a^

Entry	Photocatalyst	Temp (°C)	Equiv **17**	Yield (%)^b^	*E*_*T*_ (kcal/mol)
1	**[Ru-1]**(PF_6_)_2_	35	3	0	46.5
2	**[Ir-1]**PF_6_	35	3	0	49.2
3	**[Ir-2]**PF_6_	35	3	33	60.1
4	**[Ir-3]**	35	3	37	61.1
5	**[Ir-2]**PF_6_	0	3	51	60.1
6	**[Ir-2]**PF_6_	–40	3	64	60.1
7	**[Ir-3]**	–40	3	51	61.1
8	**[Ir-2]**PF_6_	**–40**	**5**	**71** (**65**)	**60.1**

a
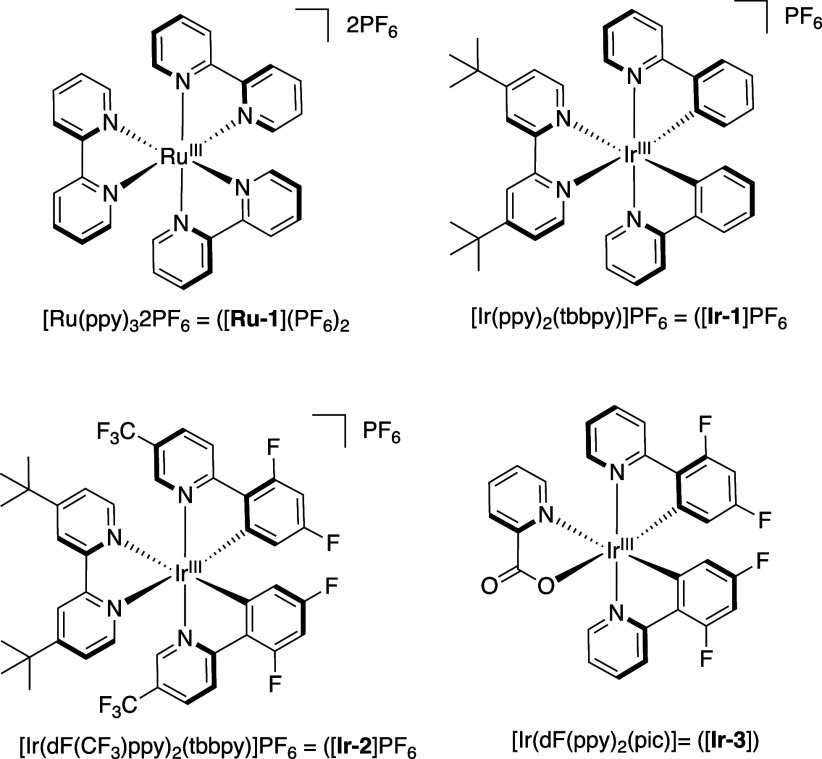

b Determined by ^1^H NMR analysis of the crude reaction mixture using 1,3,5-trimethoxybenzene.

Attempting to identify an effective sensitizer for the cyclopropene, we looked to two Ir(III) photocatalysts with higher triplet energies, [Ir(dF(CF_3_)ppy)_2_(dtbbpy)]PF_6_ ([**Ir-2**]PF_6_, *E*_*T*_*=* 60.1 kcal/mol,^40^ and Ir(dFppy)_2_(pic) ([**Ir-3**], *E*_*T*_*=* 61.1 kcal/mol^41^). This resulted in the formation of the desired product **18a** in 33% and 37% yield, respectively (entries 3 and 4), as indicated by quantitative NMR analysis. In addition, a significant amount of methyl acrylate polymerization was observed. The NMR yield of **18a**, including isomers, accounted for approximately half of the mass balance despite full consumption of the starting material. We hypothesized that the photocatalytic conditions may be detrimental to the product, leading to degradation of the product after formation. However, a control experiment revealed that **18a** was stable under the reaction conditions. An alternative cause of the low yield could be the propensity of the cyclopropene diradical intermediate to undergo deleterious reaction pathways. In an attempt to attenuate the apparent high reactivity of the cyclopropene diradical, the reaction mixture was cooled to 0 °C. This resulted in an improved yield of 51% (entry 5). The reaction was then repeated at −40 °C, close to the melting point of the solvent, which further improved the yield to 64% (entry 6). We also tested the reaction at low temperature with the other successful photocatalyst ([**Ir-3**]), which led to a slightly lower yield of 51% (entry 7). A further advantage of running the reaction at −40 °C was the lack of any methyl acrylate polymerization, which was no longer observed by NMR. We also examined increasing the methyl acrylate loading to 5 equiv, and under these conditions, the yield of **18a** was 71% (entry 8).

Having established the optimal conditions for the [2 + 2] cycloaddition, we then analyzed the generated isomeric mixture **18a**–**d**. In principle, this reaction could generate eight isomeric products, including two regioisomeric sets of four diastereomers. However, only one set of regioisomers was observed in the reaction mixture, those with the ester of the alkene located adjacent to the aryl of the cyclopropene. The four possible diastereomers (**18a**–**d**) of this set are illustrated in Figure 1. The reaction is quite diastereoselective with one isomer (**18a**) strongly predominating and only two of the other three diastereomers (**18b** and **18c**) being observed. The diastereomeric ratio significantly improved upon lowering the reaction temperature, increasing from 73:17:10 at 35 °C to 86:9:5 at −40 °C. The structure of the major diastereomer was confirmed by X-ray crystallography to be **18a**, wherein the ester at the bridge position of the cyclopropane is in the *anti* position and the ester from the methyl acrylate is in the *endo* position. The next most favored diastereomer (**18b**) has the ester at the bridge position of the cyclopropane *syn* and the methyl acrylate ester remaining *endo*. The third diastereomer (**18c**) is related to **18a** except the ester from the methyl acrylate is in the *exo* position. The corresponding *exo* isomer to **18b** (**18d**) is not observed. These results indicate that the [2 + 2] cycloaddition gives exclusively one regioisomer. Further, methyl acrylate approaching on the side of the bridging aryl group is favored by a factor of about 10:1 and the *endo* isomer is favored by about 20:1. The major *endo* diastereomer **18a** was generally easy to separate from diastereomer **18b** by silica gel column chromatography, but separation from **18c** was more challenging. Nevertheless, it was possible to isolate **18a** in 65% yield with >95:5 d.r. (62% yield and >95:5 d.r. on a 1 mmol scale). Due to the dense functionality present in the housanes, the NMR analysis was complicated by considerable signal broadening due to hindered rotation.

**Figure 1:**
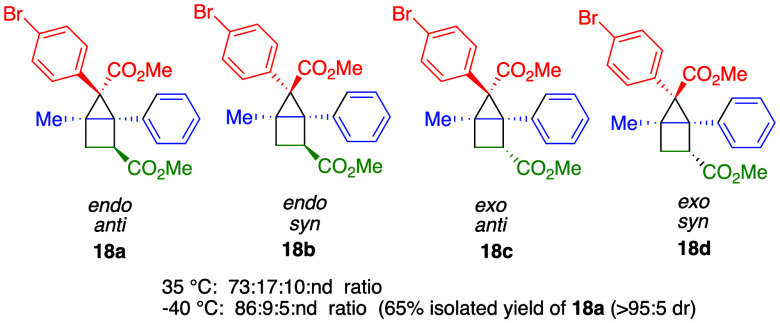
Diastereoselectivity of the [2 + 2] cycloaddition.

With these results in hand, we proceeded to explore the scope of the [2 + 2] cycloadditions with a variety of cyclopropene functionalities as well as electron-deficient alkenes (Tables 2 and 3). For each entry, a crude diastereomeric ratio is given, followed by the isolated yield and purity of the major diastereomer. In most instances, the drawn major diastereomer (**18a**, **21a**–**l**, and **23a**–**d**) could be isolated in >95:5 dr. The reaction of methyl acrylate (**17**) can be conducted with cyclopropenes derived from various aryldiazoacetates as illustrated in the formation of **18a**, **21a**–**c**, and **23a**. The reaction is also applicable to a range of cyclopropenes generated from various alkynes, as illustrated in the formation of **21d**–**f** and **23b**–**d**. The reaction can also be extended to other electron-deficient alkenes **20a**–**f**, as illustrated in the formation of **21g**–**l**.

**Table 2:**
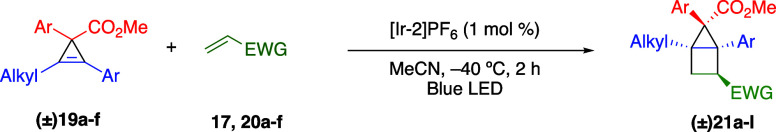

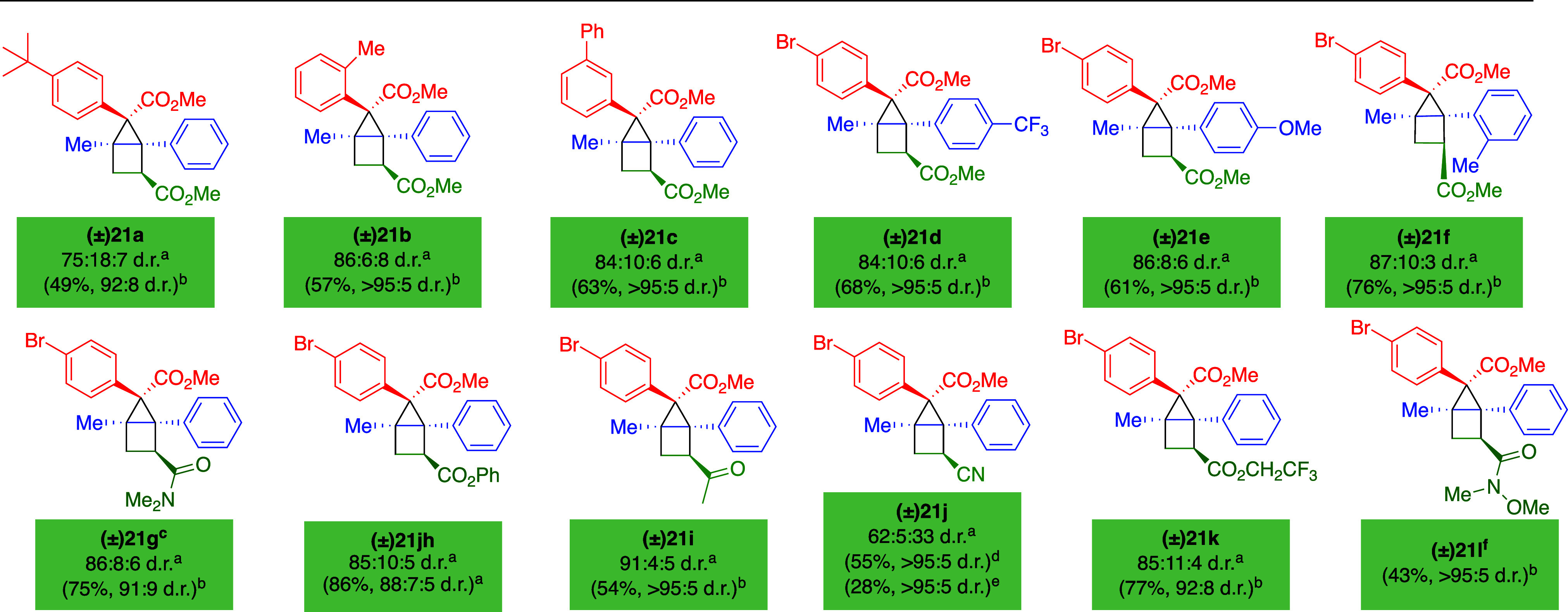
Scope of the [2 + 2] Cycloaddition^g^

a Diastereomeric ratio in the crude reaction mixture between the three stereoisomers analogous to **18a**:**18b**:**18c**.

b Isolated yield of a mixture and diastereomer ratio analogous to **18a**:**18c** after purification.

c Reaction run at −20 °C.

d Isolated major diastereomer.

e Isolated minor diastereomer

f Diastereomeric ratio could not be determined.

g Conditions: **19a**–**f** (1.0 equiv), **17**, **20a**–**f** (5.0 equiv), [Ir(dF(CF_3_)ppy)_2_(tbbpy)]PF_6_ (1.0 mol %) in acetonitrile (0.05 M) at −40 °C, 2 h.

**Table 3:**
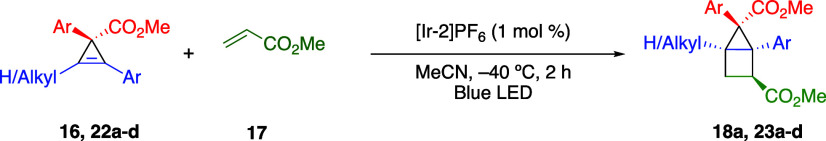

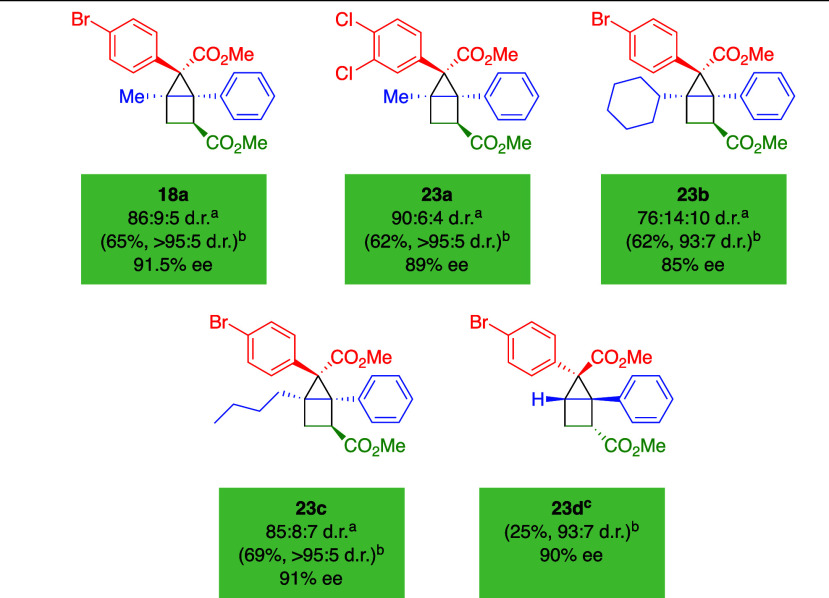
Generation of Enantioenriched Housanes^d^

a Diastereomeric ratio in the crude reaction mixture between the three stereoisomers analogous to **18a**:**18b**:**18c**.

b Isolated yield of a mixture and diastereomer ratio analogous to **18a**:**18c** after purification.

c Diastereomeric ratio could not be determined.

d Conditions: **16**, **22a–d** (1.0 equiv), **17** (5.0 equiv), [Ir(dF(CF3)ppy)_2_(tbbpy)]PF_6_ (1.0 mol %) in acetonitrile (0.05 M) at −40 °C, 2 h.

As enantioselective methods are available for generating the cyclopropenes, this approach can be applied to form enantioenriched housanes, as illustrated in Scheme 2 and Table 3. The published gold-catalyzed reactions generated the cyclopropenes **16** and **22a**–**c** in 88–91% ee as published,^24^ although it was challenging to reproduce the published yields. The rhodium-catalyzed cyclopropenation^22^ is best suited for monosubstituted alkynes, and **22d** was formed in 65% yield and 92% ee. The [2 + 2] cycloaddition proceeded without racemization as illustrated in the formation of the housanes **18a** and **23a**–**d**. The [2 + 2] cycloaddition of the less substituted cyclopropene **22d** was not as effective, as seen in the formation of the tetrasubstituted housane **23d**, generated in only 25% yield.

**Scheme 2:**
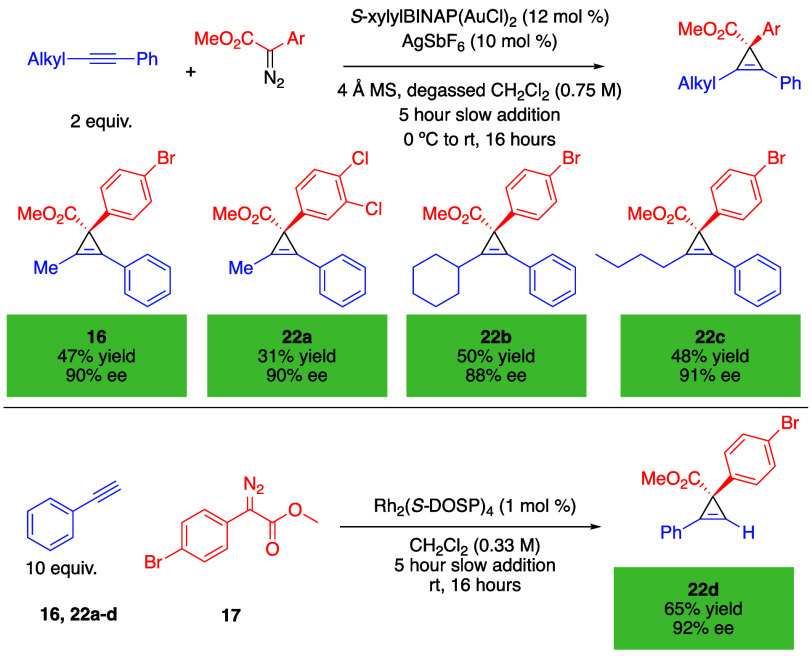
Enantioselective Cyclopropenation

In order to streamline the overall synthetic process, we also explored the possibility of telescoping the [2 + 1] and [2 + 2] cycloaddition reactions (Scheme 3). The optimal solvents for these two reactions are different. Hence, it is necessary to pass the first reaction through a plug of Celite followed by a solvent switch before conducting the second reaction. Under these conditions, the housane **18a** can be obtained in 51% yield in >95:5 ratio favoring a single diastereomer and regioisomer over two steps.

**Scheme 3:**
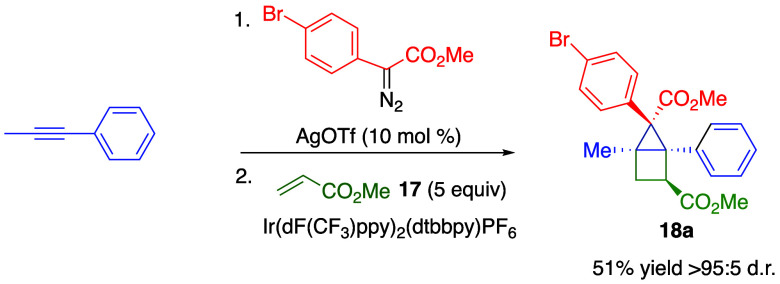
Telescoped [2 + 1]/[2 + 2] Cycloadditions

We propose that the [2 + 2] cycloaddition proceeds via the mechanism depicted in Scheme 4. Blue light irradiation results in excited-state Ir(III), which then sensitizes the cyclopropene through an energy transfer event to form the 1,2-triplet diradical cyclopropene. This then undergoes an intermolecular C–C bond formation with the terminal alkene, forming the 1,4-triplet diradical, which then forms the 1,4-singlet diradical after intersystem crossing. The 1,4-diradical can then ring close through an intramolecular C–C bond formation, resulting in the bicyclo[2.1.0]pentane (housane) product. We verified that there was no cyclopropane ring opening event at any intermediate by measuring the enantioretention of the reaction when the cyclopropene was synthesized using an asymmetric method. We measured complete enantioretention from the starting material to the bicyclo[2.1.0]pentane. The regioselectivity of the reaction was thought to be a result of the benzylic radical’s higher stability, leading to the alkyl radical reacting faster with the alkene. This could explain the increase in yield when the benzylic radical was adjacent to an electron-deficient arene over the electron-rich arene and the large decrease in yield when the alkyl group was replaced with a hydrogen.

**Scheme 4:**
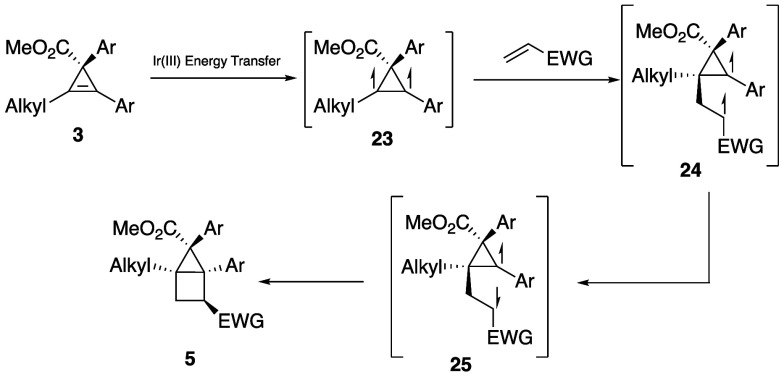
Proposed Mechanism for the [2 + 2] Cycloaddition

In conclusion, we have developed an effective two-step process for the preparation of highly substituted housanes, involving cyclopropenation of alkynes followed by visible-light-sensitized [2 + 2] cycloaddition. The reactions proceed with good regio- and diastereocontrol. When the cyclopropenes are generated by asymmetric cyclopropenation, the enantioselectivity of the process can be controlled as well. The reaction proceeds with a relatively broad scope, providing access to a range of highly substituted housanes in a stereodefined manner.

## Data Availability

The data underlying this study are available in the published article and its Supporting Information.

## Supporting Information Available

Complete experimental procedures and compound characterization (PDF)
