# Supplementary material for: Stereoselective Synthesis of Highly Functionalized Bicyclo[2.1.0]pentanes by Sequential [2 + 1] and [2 + 2] Cycloadditions
Source: Org Lett. 2025 Feb 10;27(7):1673–8. doi: 10.1021/acs.orglett.5c00054 (PMC11852226; doi:10.1021/acs.orglett.5c00054)
Supplement: Supplementary file 1 — ol5c00054_si_001.pdf [file ol5c00054_si_001.pdf]

Stereoselective Synthesis of Highly Functionalized Bicyclo[2.1.0]pentanes by Sequential [2+1] and [2+2] Cycloadditions.

Brockton Keen,[a] Christina Cong,[b] Alberto Castanedo,[b] Geraint H. M. Davies,[c,*] Robert R. Knowles[b,*] and Huw M. L. Davies.[a,*]

[a]Department of Chemistry, Emory University, 1515 Dickey Drive, Atlanta, Georgia 30322

email: hmdavie@emory.edu

[b]Department of Chemistry, Princeton University, Princeton, New Jersey 08544

email: rknowles@princeton.edu

[c]PostEra, One Broadway; 14th Floor; Cambridge, MA 02142

email: geraint.davies@postera.ai

Supporting Information

Table of Contents

I. General Considerations S-3
   a. General Information S-3
   b. Caution about Diazo Compounds S-3
   c. Low Temperature Irradiation Setup S-3
II. General Procedure S-5
   a. General Procedure A S-5
   b. General Procedure B S-5
   c. General Procedure C S-5
   d. General Procedure D S-5
III. Preparation of Starting Materials S-6
   a. General Procedure for Known Diazo Compound Synthesis S-6
   b. Novel Diazo Compound Synthesis S-6
   c. Internal Alkyne Synthesis S-8
   d. Racemic Cyclopropene Synthesis S-10
   e. Chiral Cyclopropene Synthesis S-13
IV. Preparation of Products from Substate Table S-16
V. Diastereomer NMR S-25
   a. Evidence for Diastereomer Stereochemistry Assignment S-26
   b. Crude and Purified Spectra and Diastereomeric Ratios S-65
VI. Failed Reactions S-100
VII. References S-101
VIII. NMR Spectra S-102
   a. NMR Spectra of Starting Materials S-102
   b. NMR of Bicyclo[2.1.0]pentane Products S-122
IX. Chiral SFC S-142
X. X-ray Crystallography Data S-163

I. General Considerations

Ia. General Information

Unless otherwise noted, all reagents and solvents were purchased from commercial sources (e.g. Sigma Aldrich, Strem, TCI Chemicals, Oakwood Chemicals, Ambeed) and used without further purification. All electron deficient alkenes were distilled prior to use to remove any stabilizer and store below 0 ºC. All known diazo compounds were synthesized using previously reported procedures. (*S*)-xylylBINAP AuCl was synthesized following a known literature procedure.[1] 4-acetamidobenzenesulfonyl azide (*p*ABSA) was synthesized following a known literature procedure.[2] All reactions were performed in oven-dried glassware or glassware flame-dried while under reduced pressure and carried out under a nitrogen or argon atmosphere while stirring. Thin layer chromatography (TLC) analysis was performed with aluminum-backed silica gel plates and visualized with ultraviolet light (254 nm), cerium aluminum molybdate (CAM) or potassium permanganate ($KMnO_4$). Preparative thin-layer chromatography was performed using 20 x 20 cm, 1000 μm glass-backed SiliaPlate preparative TLC plates (SiliCycle®, Cat. No. TLG-R10011B-341) with a silica gel adsorbent and F254 fluorescent indicator. Silica gel chromatography was performed manually using a Chemglass column apparatus filled with SiliaFlash® P60 or SiliCycle® F60. NMR were obtained in a solution using $CDCl_3$, toluene-d8, or CD_3CN on a 400 MHz (^{13}C at 101 MHz, ^{19}F at 376 MHz) Bruker or Varian, 500 MHz (^{13}C at 126 MHz) Bruker or 600 MHz (^{13}C at 151 MHz, ^{19}F at 565 MHz) INOVA 600 or Bruker 600, or 800 MHz Bruker (^{13}C at 201 MHz) NMR spectrometer. Residual solvent was used as the internal standard (7.26 ppm for ^1H and 77.16 ppm for ^{13}C in $CDCl_3$, 2.08 ppm for ^1H in toluene-d8, and 1.94 ppm for ^1H in CD_3CN) with multiplicities (*s* = singlet, br. *s* = broad singlet, *d* = doublet, *t* = triplet, *q* = quartet, *p* = pentet, *m* = multiplet, *dd* = doublet of doublet etc.) Coupling constants were obtained from the spectra, reported in Hz, and are uncorrected. All ^{13}C spectra are proton decoupled. All NMR spectra were obtained at room temperature unless otherwise denoted. Structural assignments were made with additional information from gCOSY, gHSQC, and gHMBC experiments. Mass spectrometry was taken on a Thermo Finnigan LTQ-FTMS spectrometer with APCI or ESI and on an Agilent 6230 TOF LC/MS.Enantiomeric excess data was obtained using a Waters SFC eluting with supercritical CO_2 and either a 1:1 mixtures of HPLC grade methanol:isopropanol with 0.2% formic acid, 1:1 mixture of HPLC grade ethanol:isopropanol with 0.2% formic acid, or 1:1 ethanol:acetonitrile with 0.2% formic acid. SFC conditions were determined by separating a racemic standard synthesized using silver(I) triflate or a 1:1 mixture of Rh_2(*R*-DOSP)$_4$: Rh_2(*S*-DOSP)$_4$. Optical rotation was measured using a Jasco P-2000 polarimeter.

Ib. Caution About Diazo Compounds

Caution! This project includes the use of diazo compounds. Diazo compounds are high energy and possibly explosive. Although we experienced no unexpected explosive decomposition in this work, safety precautions should always be followed when working with diazo compounds. See the recent review by Bull *et. al* for a more complete analysis of potential risks.[3]

Ic. Low Temperature Irradiation Setup

The screw-cap test tubes were placed on a custom-built aluminum block and submerged up to the solvent level in an acetone bath inside of a Thermo/Neslab CB80 Cryocool. The temperature of the reaction was controlled by the thermocouple in contact with the aluminum block placed in adjacent to the reaction and verified using a low temperature alcohol thermometer. A Chanzon High Power 100W LED 440 nm chip (part number 1DGL-JC-100W-440) was mounted to a ASHATA 40x40 mm water-cooling aluminum block using GENELL G109 thermally conductive glue and powered by a Chanzon 3000 mA constant current LED Driver (Model QH-100LC6-10x10). The LED and cooling block were mounted using a lab clamp and suspended above the reaction at approximately 5 centimeters above the reaction. Ice water was pumped through the aluminum block using an aquarium pump. Ice was replenished during the duration of the reaction as necessary.

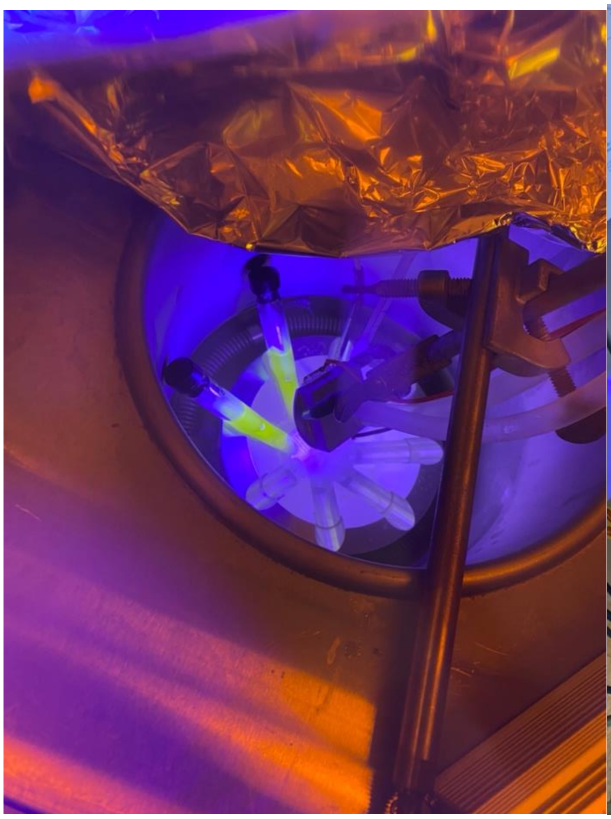
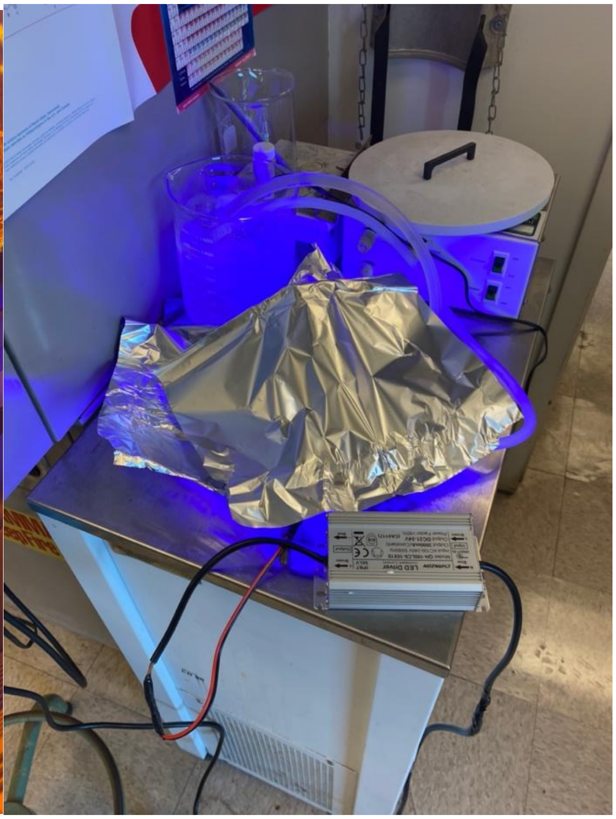

II. General Procedures

IIa. General Procedure A

To a flame-dried round-bottom flask was added an oven-dried stir bar and silver(I) triflate (10 mol %). The flask was wrapped with aluminum foil to exclude light and sealed with a rubber septum. The atmosphere of the flask was exchanged by applying vacuum and backfilling with nitrogen (this was done three times). To this was then added the internal alkyne (2 equiv) and CH_2Cl_2 (20% total volume, 0.25 M). The diazo compound (1 equiv) was dissolved in CH_2Cl_2 (80% total volume, 0.0625 M, 0.05 M overall) and added slowly *via* syringe pump over 2 hours. After addition, the resulting solution was allowed to stir for 1-14 additional hours. Upon competition, the solution was filtered through a celite pad and concentrated *in vacuo*. The resulting crude residue was then purified on silica gel to afford the product.

IIb. General Procedure B

To a flame-dried round-bottom flask was added an oven-dried stir bar, 3 g of activated 4 Å molecular sieves, and (*S*)-xylylBINAP$(AuCl)_2$ (12 mol %). The reaction was moved into a glovebox, and silver(I) hexafluoroantimonate(V) (10 mol %) was added. The reaction flask was then sealed with a rubber septum and transferred out of the glovebox. The reaction was wrapped with aluminum foil to exclude light. To this was then added the internal alkyne (5 equiv) and CH_2Cl_2 (20% total volume, 0.25 M). This was allowed to stir at room temperature for 20 minutes. The reaction was then placed in an ice bath and cooled to 0 ºC. The diazo compound (1 equiv) was placed in a vial and sealed with a cap and PTFE septum. The atmosphere of the vial was then exchanged by applying vacuum and backfilling with nitrogen (this was done three times). The diazo was dissolved in CH_2Cl_2 (80% total volume, 0.0625 M, 0.05 M overall) and added slowly *via* syringe pump over 3 hours. After addition, the resulting solution was allowed to stir for 14 additional hours. Upon competition, the reaction was filtered through a celite pad and concentrated *in vacuo.* The resulting crude residue was then purified on silica gel to afford the product.

IIc. General Procedure C

To a flame-dried round-bottom flask was added an oven-dried stir bar, 3 g of activated 4 Å molecular sieves, (*S*)-xylylBINAP$(AuCl)_2$ (12 mol %), and silver(I) hexafluoroantimonate(V) (10 mol %). The reaction flask was then sealed with a rubber septum and wrapped with aluminum foil to exclude light. The atmosphere of the flask was exchanged by applying vacuum and backfilling with nitrogen (this was done three times).To this was then added the internal alkyne (5 equiv) and CH_2Cl_2 (20% total volume, 0.25 M). This was allowed to stir at room temperature for 20 minutes. The reaction was then placed in an ice bath and cooled to 0 ºC. The diazo compound (1 equiv) was placed in a vial and sealed with a cap and PTFE septum. The atmosphere of the vial was then exchanged by applying vacuum and backfilling with nitrogen (this was done three times). The diazo was dissolved in CH_2Cl_2 (80% total volume, 0.0625 M, 0.05 M overall) and added slowly *via* syringe pump over 3 hours. After addition, the resulting solution was allowed to stir for 14 additional hours. Upon competition, the solution was filtered through a celite pad and concentrated *in vacuo.* The resulting crude residue was then purified on silica gel to afford the product.

IId. General Procedure D

To an oven-dried screwcap test tube was added an oven-dried stir bar, $Ir(dF(CF_3)ppy)_2(tbbpy)PF_6$ (1 mol %), and the cyclopropene (1 equiv). This was sealed with a cap and PTFE/silicone septum. The perimeter of the cap was then wrapped with electrical tape. The atmosphere of the test tube was then exchanged by applying vacuum and backfilling with nitrogen (this was done three times). Under the nitrogen atmosphere was added degassed MeCN (0.05 M), and the alkene (5 equiv). The top of the test tube was then quickly sealed with electrical tape, and to the perimeter was added another layer of electrical tape. The resulting mixture was then cooled and irradiated for 2 h with blue LEDs. The reaction was then concentrated *in vacuo*, and the residue purified on silica gel.

III. Preparation of Starting Materials

IIIa. General Procedure for Known Diazo Synthesis

To a 50 mL round bottom flask was added 2-phenyl acetic acid (2 grams) and a stir bar. To this was added MeOH (20 mL) and 6 drops of conc. H_2SO_4. This was sealed with a rubber septum and a needle attached to an argon balloon was placed through the septum. This stirred overnight. The reaction was quenched by adding sat. $NaHCO_3$. This was added to a separatory funnel and extracted three times with CH_2Cl_2. The organic layers were combined and washed with a brine solution, then dried over Na_2SO_4 and concentrated *in vacuo.* This material was then used without further purification. To a 100 mL round bottom flask was added a stir bar and 4-acetamidobenzenesulfonyl azide (*p*ABSA) (1.5 equiv) and the methyl 2-aryl acetate as a neat solution (1 equiv). To this was added MeCN (0.25 M). This was sealed with a rubber septum and the atmosphere purged by inserting a needle attached to a large argon balloon and an exit needle and allowing the balloon to fully deflate. The exit needle was removed, and the balloon was refilled with argon and placed back through the rubber septum. The reaction was placed into an ice bath and allowed to cool. 1,8-Diazabicyclo[5.4.0]undec-7-ene (DBU) (2 equiv) was added dropwise to the reaction using a needle and syringe. After addition, the reaction was allowed to warm and stirred at room temperature for 16 h. The reaction was quenched with saturated aqueous NH_4Cl and diluted with deionized water. The reaction was extracted with CH_2Cl_2 three times, washed with brine, and dried over Na_2SO_4, concentrated *in vacuo*, and purified on silica gel to afford the product.

IIIb. Novel Diazo Synthesis

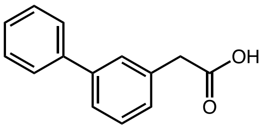

2-([1,1'-biphenyl]-3-yl)acetic acid (S1): Into a flame dried round-bottom flask was added a stir bar, phenylboronic acid (1.34 g, 11.0 mmol, 1.1 equiv), 2-(3-bromophenyl)acetic acid (2.15 g, 10.0 mmol, 1 equiv), sodium carbonate (1.17 g, 11.0 mmol, 1.1 equiv), and tetrakis(triphenylphosphine)palladium(0) (116 mg, 0.10 mmol, 1 mol %). A findensor was placed atop this and the findensor capped with a rubber septum. The reaction atmosphere was then exchanged by applying vacuum and backfilling with nitrogen (this was conducted three total times). 1,2-Dimethoxyethane (25 mL) was added via syringe. An argon balloon was then placed atop the reaction. The reaction flask was wrapped in foil around the heating block on a hot plate and the heat set to 100 ºC and refluxed for 16 h. The reaction was filtered through a celite plug using CH_2Cl_2, washed three times with 3 M hydrochloric acid, followed by brine, and dried over Na_2SO_4. The reaction was concentrated *in vacuo* to give a white powder. NMR analysis showed a mixture of starting material and product. The crude powder was then subjected to the same conditions as above, with 2-(3-bromophenyl)acetic acid (2.15 g, 10.0 mmol, 1 equiv), sodium carbonate (1.17 g, 11.0 mmol, 1.1 equiv), and tetrakis(triphenylphosphine)palladium(0) (116 mg, 0.10 mmol, 1 mol %) in 1,2-Dimethoxyethane (25 mL) at reflux for 48 h. The reaction was filtered through a celite plug using CH_2Cl_2, washed three times with 3 M hydrochloric acid, followed by brine, and dried over Na_2SO_4. The reaction was concentrated *in vacuo.* NMR analysis showed complete consumption of starting material. The reaction was then moved forward to esterification without further characterization or purification.

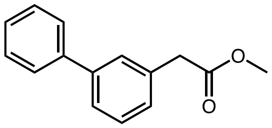

Methyl 2-([1,1'-biphenyl]-3-yl)acetate (S2): Into the round-bottom flask containing the crude 2-([1,1'-biphenyl]-3-yl)acetic acid was added a stir bar and methanol (25 mL). To this was added 8 drops of concentrated sulfuric acid. The flask was capped with a rubber septum, an argon balloon was placed atop the reaction, and the reaction allowed to stir at room temperature for 16 h. The reaction was quenched with saturated aqueous $NaHCO_3$, extracted with CH_2Cl_2 three total times, washed with brine, and dried over Na_2SO_4. The reaction was concentrated *in vacuo*, and purified on silica gel (10% ethyl ether in pentane) resulting in an oil. (1.23 g, 5.45 mmol, 54% over two steps).

^{1}H NMR (600 MHz, $CDCl_3$) δ 7.59 – 7.56 (m, 2H), 7.49 (dd, *J* = 6.5, 1.4 Hz, 2H), 7.42 (dd, *J* = 8.4, 7.0 Hz, 2H), 7.39 (dd, *J* = 8.4, 7.5 Hz, 1H), 7.33 (tt, *J* = 7.3, 1.2 Hz, 1H), 7.26 (dt, *J* = 6.5, 1.1 Hz, 1H), 3.70 (s, 3H), 3.68 (s, 2H).
^{1}H NMR spectrum is consistent with reported values[4].

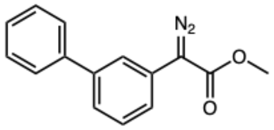

Methyl 2-([1,1'-biphenyl]-3-yl)-2-diazoacetate (S3): Into a flame-dried round-bottom flask was added oven dried stir bar, 3-((4-acetoxyphenyl)sulfonyl)-2l4-triaza-1,2-dien-1-ide (*p*-ABSA) (1.81 g, 7.54 mmol, 1.5 equiv), and methyl 2-([1,1'-biphenyl]-3-yl)acetate (1.14 g, 5.025 mmol, 1.0 equiv). MeCN (20 mL) was added, and the reaction vessel capped with a rubber septum. The reaction was cooled to 0 ºC using an ice bath while stirring. 1,8-Diazabicyclo[5.4.0]undec-7-ene (DBU) (1.50 mL, 10.05 mmol, 2 equiv) was added dropwise via syringe. After addition, the reaction was allowed to warm and stirred at room temperature for 16 h. The reaction was quenched with saturated aqueous NH_4Cl and diluted with deionized water. The reaction was extracted with CH_2Cl_2 three times, washed with brine, and dried over Na_2SO_4, concentrated *in vacuo*, and purified on silica gel (5% ethyl ether in pentane) to afford the product as an orange solid. (708 mg, 56%).
^{1}H NMR (400 MHz, $CDCl_3$) δ 7.72 (dt, *J* = 2.5, 1.1 Hz, 1H), 7.61 – 7.57 (m, 2H), 7.48 – 7.39 (m, 5H), 7.36 (tt, *J* = 7.4, 1.3 Hz, 1H), 3.89 (s, 3H).
^{13}C NMR (101 MHz, $CDCl_3$) δ 165.6, 142.1, 140.8, 129.4, 128.8, 127.6, 127.2, 126.1, 124.8, 122.8, 122.8, 52.1. (C=N_2 signal is too weak and not observed)
HRMS (ESI) m/z: $[M+Na]^+$ calc'd for $C_{15}H_{12}O_2N_2{}^{23}Na$, 275.0791, found 275.0792

IIIc. Internal Alkyne Synthesis

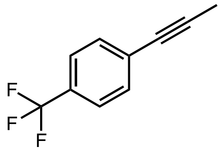

1-(prop-1-yn-1-yl)-4-(trifluoromethyl)benzene (S4): To a flame-dried round-bottom flask was added a flame dried stir bar. The flask was sealed with a rubber septum and flame dried under vacuum. The reaction atmosphere was then exchanged by applying vacuum and backfilling with nitrogen (this was conducted three total times). To this flask was added 1-ethynyl-4-(trifluoromethyl)benzene (2.5 mL, 15 mmol. 1 equiv), and THF (30 mL). The nitrogen line was removed, and an argon balloon was placed atop the reaction. The reaction vessel was cooled to -78 ºC using a dry ice/acetone bath and allowed to stir for 5 minutes. To this was then added 2.5 M n-butyllithium (6.3 mL, 15.75 mmol, 1.05 equiv), dropwise. This was allowed to stir for 30 minutes. After 30 minutes, iodomethane (1.03 mL, 16.50 mmol, 1.1 equiv) was added in slowly. The flask was removed from the dry ice/acetone bath, allowed to warm to room temperature and stirred for 16 h. After 16 h, the reaction was cooled to -78 ºC and quenched with 2-propanol, then water. The reaction was worked up by diluting with water and extracting with CH_2Cl_2. The aqueous layer was then extracted two additional times with CH_2Cl_2. The organic layers were combined and washed with brine, then dried over Na_2SO_4, and the product concentrated *in vacuo.* The crude material was purified on silica gel (100% pentane) to afford the product as a clear oil. (2.428 g, 88%).
^{1}H NMR (600 MHz, $CDCl_3$) δ 7.52 – 7.50 (m, 2H), 7.45 (d, *J* = 8.1 Hz, 2H), 2.05 (s, 3H).
^{1}H NMR spectrum is consistent with reported values[5].

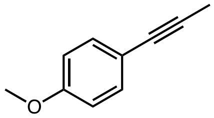

1-methoxy-4-(prop-1-yn-1-yl)benzene (S5): To a flame-dried round-bottom flask was added a flame dried stir bar. The flask was sealed with a rubber septum and flame dried under vacuum. The reaction atmosphere was then exchanged by applying vacuum and backfilling with nitrogen (this was conducted three total times). To this flask was added 1-ethynyl-4-methoxybenzene (0.97 mL, 7.5 mmol. 1 equiv), and THF (15 mL). The nitrogen line was removed, and an argon balloon was placed atop the reaction. The reaction vessel was cooled to -78 ºC using a dry ice/acetone bath and allowed to stir for 5 minutes. To this was then added 2.5 M n-butyllithium (3.2 mL, 7.9 mmol, 1.05 equiv), dropwise. This was allowed to stir for 30 minutes. After 30 minutes, iodomethane (0.51 mL, 8.3 mmol, 1.1 equiv) was added in slowly. The flask was removed from the dry ice/acetone bath, allowed to warm to room temperature and stirred for 16 h. After 16 h, the reaction was cooled to -78 ºC and quenched with 2-propanol, then water. The reaction was worked up by diluting with water and extracting with CH_2Cl_2. The aqueous layer was then extracted two additional times with CH_2Cl_2. The organic layers were combined and washed with brine, then dried over Na_2SO_4, and the product concentrated *in vacuo.* The crude material was purified on silica gel (1% ethyl ether in pentane) to afford the product as a clear oil. (1.1 g, >99%).
^{1}H NMR (600 MHz, $CDCl_3$) δ 7.32 (d, *J* = 8.8 Hz, 2H), 6.81 (d, *J* = 8.9 Hz, 2H), 3.80 (s, 3H), 2.03 (s, 3H).
^{1}H NMR spectrum is consistent with reported values[6]

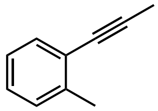

1-methyl-2-(prop-1-yn-1-yl)benzene (S6): To an oven-dried test tube was added an oven-dried stir bar. This was sealed with a cap and a PTFE septum. The vial atmosphere was then exchanged by applying vacuum and backfilling with nitrogen (this was conducted three total times). An argon balloon was then placed atop the reaction vial. To the vial was then added 1-ethynyl-2-methylbenzene (0.63 mL, 5.0 mmol, 1 equiv). THF (10 mL) was added via syringe. The reaction was cooled in a dry ice/acetone bath while stirring. To this was then added 2.5 M n-butyllithium (2.1 mL, 5.25 mmol, 1.05 equiv), dropwise. The reaction was allowed to react for 30 minutes and was then allowed to warm to room temperature. Iodomethane (0.34 mL, 5.5 mmol, 1.1 equiv) was added using a syringe and dropwise addition. The

reaction was stirred at room temperature for 16 h. Upon completion, the reaction was cooled to -78 ºC and quenched with 2-propanol, then water. The reaction was then extracted three times using CH_2Cl_2, the organic layers combined and washed with brine, then dried over Na_2SO_4. This was then concentrated *in vacuo*. The crude material was purified on silica gel (100% pentane) to afford the product as a clear oil. (295 mg, 45%).
^{1}H NMR (600 MHz, $CDCl_3$) δ 7.36 (d, *J* = 7.5 Hz, 1H), 7.19 – 7.15 (m, 2H), 7.13 – 7.07 (m, 1H), 2.42 (s, 3H), 2.10 (s, 3H).
^{1}H NMR spectrum is consistent with reported values[7].

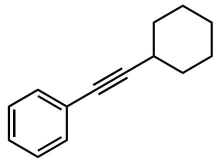

(Cyclohexylethynyl)benzene (S7): To a flame-dried round-bottom flask was added a stir bar and bis(triphenylphosphine)palladium(II) dichloride (526 mg, 0.750 mmol, 5 mol %) and triethylamine (60 mL). The round-bottom flask was sealed with a rubber septum and then degassed by sparging with argon for 5 minutes while stirring. An argon balloon was placed atop the reaction. To this was then added added iodobenzene (1.67 mL, 15 mmol, 1.0 equiv) and ethynylcyclohexane (2.35 mL, 18 mmol, 1.2 equiv) using a syringe through the septum. This was allowed to stir for 5 minutes. Copper(I) iodide (526 mg, 0.11 mmol, 10 mol %) was added through the top before quickly resealing. The reaction was allowed to stir at room temperature for 16 h. The crude material was concentrated *in vacuo* and then suspended in pentane. The pentane solution was then passed through a silica gel plug. The product was used without further purification.

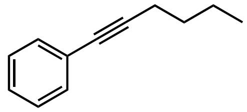

Hex-1-yn-1-ylbenzene (S8): To a flame-dried round-bottom flask was added a stir bar and bis(triphenylphosphine)palladium(II) dichloride (154 mg, 0.220 mmol, 2.2 mol %). The catalyst was then dissolved in triethylamine (100 mL). The round-bottom flask was sealed with a rubber septum and then degassed by sparging with argon for 5 minutes while stirring. An argon balloon was placed atop the reaction. To this was then added iodobenzene (1.34 mL, 12 mmol, 1.2 equiv) and hex-1-yne (1.1 mL, 10 mmol, 1.0 equiv) using a syringe through the septum. This was allowed to stir for 5 minutes. Copper(I) iodide (21 mg, 0.11 mmol, 1.1 mol %) was added through the top before quickly resealing. The reaction was allowed to stir at room temperature for 16 h. The reaction was worked up by filtering through a celite pad while rinsing with CH_2Cl_2. The reaction was extracted with CH_2Cl_2 and the aqueous layer extracted three times. The organic layers were combined, washed with brine, dried over Na_2SO_4, and concentrated *in vacuo.* The resulting crude was purified on silica gel (100% pentane) to afford the product as a colorless oil. (1.21 g, 76%).
^{1}H NMR (600 MHz, $CDCl_3$) δ 7.39 (dd, *J* = 7.8, 1.8 Hz, 2H), 7.30 – 7.23 (m, 3H), 2.41 (t, *J* = 7.1 Hz, 2H), 1.59 (dt, *J* = 15.1, 6.9 Hz, 2H), 1.49 (dt, *J* = 15.2, 7.3 Hz, 2H), 0.95 (t, *J* = 7.3 Hz, 3H).
^{1}H NMR spectrum is consistent with reported values[8].

IIId. Racemic cyclopropene synthesis

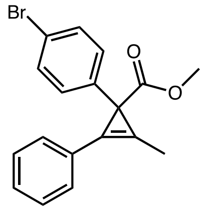

(±)-Methyl 1-(4-bromophenyl)-2-methyl-3-phenylcycloprop-2-ene-1-carboxylate (±16): General procedure A was followed using silver(I) triflate (77 mg, 0.30 mmol, 10 mol %), prop-1-yn-1-ylbenzene (0.92 mL, 6 mmol, 2 equiv) and CH_2Cl_2 (48 mL) added to a 250 mL round-bottom flask. Methyl 2-(4-bromophenyl)-2-diazoacetate (765 mg, 3.0 mmol, 1 equiv) in CH_2Cl_2 (12 mL) was added slowly *via* syringe pump over 2 hours and allowed to stir overnight. The reaction mixture was worked up as described in general procedure A and purified on silica gel (15% ethyl ether in pentane) to afford a white solid. The product was triturated using pentane and the pentane decanted to afford the product as a white solid (757 mg, 74%).
^{1}H NMR (600 MHz, $CDCl_3$) δ 7.51 (dd, *J* = 7.2, 1.2 Hz, 2H), 7.41 (td, *J* = 7.3, 1.1 Hz, 2H), 7.40 – 7.32 (m, 3H), 7.24 (dd, *J* = 8.6, 0.9 Hz, 2H), 3.69 (s, 3H), 2.37 (s, 3H).
^{1}H NMR spectrum is consistent with reported values[9].

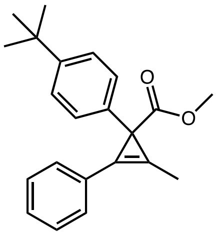

(±)-Methyl 1-(4-(*tert*-butyl)phenyl)-2-methyl-3-phenylcycloprop-2-ene-1-carboxylate (±19a): General procedure A was followed using silver(I) triflate (26 mg, 0.10 mmol, 10 mol %), prop-1-yn-1-ylbenzene (0.24 mL, 2.0 mmol, 2 equiv) and CH_2Cl_2 (4 mL) added to a 50 mL round-bottom flask. Methyl 2-(4-(tert-butyl)phenyl)-2-diazoacetate (232 mg, 1.0 mmol, 1 equiv) in CH_2Cl_2 (6 mL) was added slowly *via* syringe pump over 2 hours and allowed to stir an additional 14 h. The reaction mixture was worked up as described in general procedure A and purified on silica gel (10% ethyl ether in pentane) to afford the product as a yellow oil. The product was triturated using pentane and the pentane decanted to afford the product as an off-white solid (183 mg, 57%).
^{1}H NMR (600 MHz, $CDCl_3$) δ 7.56 (dt, *J* = 7.0, 1.4 Hz, 2H), 7.42 (tt, *J* = 7.3, 1.5 Hz, 2H), 7.35 (tt, *J* = 7.5, 1.9 Hz, 1H), 7.29 (s, 4H), 3.70 (s, 3H), 2.39 (s, 3H), 1.29 (s, 9H).
^{1}H NMR spectrum is consistent with reported values[10].

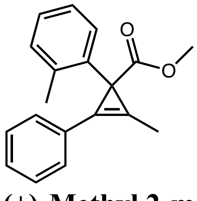

(±)-Methyl 2-methyl-3-phenyl-1-(*o*-tolyl)cycloprop-2-ene-1-carboxylate (±19b): General procedure A was followed using silver(I) triflate (26 mg, 0.10 mmol, 10 mol %), prop-1-yn-1-ylbenzene (0.24 mL, 2.0 mmol, 2 equiv) and CH_2Cl_2 (4 mL) added to a 50 mL round-bottom flask. Methyl 2-diazo-2-(*o*-tolyl)acetate (190 mg, 1.0 mmol, 1 equiv) in CH_2Cl_2 (6 mL) was added slowly *via* syringe pump over 2 hours and allowed to stir an additional 6 h. The reaction mixture was worked up as described in general procedure A and purified on silica gel (10% ethyl ether in pentane) to afford the product as a yellow oil. The product was triturated using pentane and the pentane decanted to afford the product as a white solid (164 mg, 59%).

^{1}H NMR (600 MHz, $CDCl_3$) δ 7.62 – 7.57 (m, 2H), 7.48 – 7.39 (m, 2H), 7.37 (tt, *J* = 7.5, 1.1 Hz, 1H), 7.20 (dd, *J* = 7.6, 1.3 Hz, 1H), 7.18 – 7.10 (m, 2H), 7.04 (dddd, *J* = 7.6, 6.9, 1.9, 0.7 Hz, 1H), 3.70 (s, 3H), 2.42z (s, 3H), 2.39 (s, 3H).
^{1}H NMR spectrum is consistent with reported values[10].

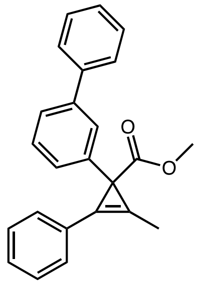

(±)-Methyl 1-([1,1'-biphenyl]-3-yl)-2-methyl-3-phenylcycloprop-2-ene-1-carboxylate (±19c): General procedure A was followed using silver(I) triflate (39 mg, 0.15 mmol, 10 mol %), prop-1-yn-1-ylbenzene (348 mg, 3.0 mmol, 2.0 equiv) and CH_2Cl_2 (6 mL) added to a 100 mL round-bottom flask. Methyl 2-([1,1'-biphenyl]-3-yl)-2-diazoacetate (378 mg, 1.5 mmol, 1 equiv) in CH_2Cl_2 (24 mL) was added slowly *via* syringe pump over 2 hours and allowed to stir overnight. The reaction mixture was worked up as described in general procedure A and purified on silica gel (10% ethyl ether in pentane) to afford a white solid. (319 mg, 63%).
^{1}H NMR (600 MHz, $CDCl_3$) δ 7.61 – 7.59 (m, 1H), 7.58 – 7.53 (m, 4H), 7.44 – 7.38 (m, 5H), 7.36 – 7.30 (m, 4H), 3.71 (s, 3H), 2.42 (s, 3H).
^{1}H NMR spectrum is consistent with reported values[10].

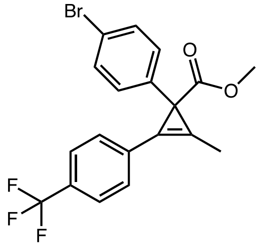

(±)-Methyl 1-(4-bromophenyl)-2-methyl-3-(4-(trifluoromethyl)phenyl)cycloprop-2-ene-1-carboxylate (±19d): General procedure A was followed using silver(I) triflate (39 mg, 0.15 mmol, 10 mol %), 1-(prop-1-yn-1-yl)-4-(trifluoromethyl)benzene (552 mg, 3.0 mmol, 2.0 equiv) and CH_2Cl_2 (6 mL) added to a round-bottom flask. Methyl 2-(4-bromophenyl)-2-diazoacetate (383 mg, 1.5 mmol, 1.0 equiv) in CH_2Cl_2 (24 mL) was added slowly *via* syringe pump over 2 hours and allowed to stir an additional 90 min. The reaction mixture was worked up as described in general procedure A and purified on silica gel (15% ethyl ether in pentane) to afford a yellow oil (227 mg, 37%).
^{1}H NMR (400 MHz, $CDCl_3$) δ 7.67 (d, *J* = 8.2 Hz, 1H), 7.61 (d, *J* = 8.2 Hz, 1H), 7.39 (dt, *J* = 8.6, 2.6 Hz, 1H), 7.21 (dt, *J* = 8.6, 2.6 Hz, 1H), 3.70 (s, 1H), 2.41 (s, 1H).
^{13}C NMR (151 MHz, $CDCl_3$) δ 174.3, 139.6, 131.4, 130.9 (q, *J* = 32.6 Hz), 129.9, 129.5, 126.0 (q, *J* = 3.8 Hz), 124.0 (q, *J* = 272.0 Hz), 120.6, 114.5, 107.5, 52.3, 35.1, 9.9.
^{19}F NMR (565 MHz, $CDCl_3$) δ -62.78.
HRMS (APCI) m/z: $[M+H]^+$ calc'd for $C_{19}H_{15}O_2{}^{79}BrF_3$, 411.0202, found 411.0200

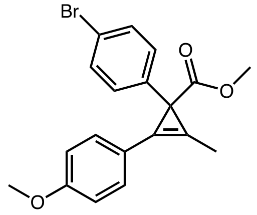

(±)-Methyl 1-(4-bromophenyl)-2-(4-methoxyphenyl)-3-methylcycloprop-2-ene-1-carboxylate (±19e): General procedure A was followed using silver(I) triflate (26 mg, 0.10 mmol, 10 mol %), 1-methoxy-4-(prop-1-yn-1-yl)benzene (0.30 g, 2.1 mmol, 2.1 equiv) and CH_2Cl_2 (4 mL) added to a 50 mL round-bottom flask. Methyl 2-(4-bromophenyl)-2-diazoacetate (257 mg, 1.01 mmol, 1 equiv) in CH_2Cl_2 (16 mL)

was added slowly *via* syringe pump over 2 hours and allowed to stir an additional 14 h. The reaction mixture was worked up as described in general procedure A and purified on silica gel (15% ethyl ether in pentane) to afford a yellow viscous oil. (152 mg, 40%).

^{1}H NMR (400 MHz, $CDCl_3$) δ 7.47 (d, *J* = 8.8 Hz, 2H), 7.39 (d, *J* = 8.6 Hz, 2H), 7.25 (d, *J* = 8.5 Hz, 2H), 6.96 (d, *J* = 8.8 Hz, 2H), 3.85 (s, 3H), 3.71 (s, 3H), 2.36 (s, 3H).

^{13}C NMR (101 MHz, $CDCl_3$) δ 174.9, 160.2, 140.4, 131.1, 130.7, 129.9, 120.0, 118.7, 114.5, 107.9, 107.4, 55.4, 52.0, 34.7, 9.4.

HRMS (APCI) m/z: $[M+H]^+$ calc'd for $C_{19}H_{18}O_3{}^{79}Br$, 373.0434, found 373.0434

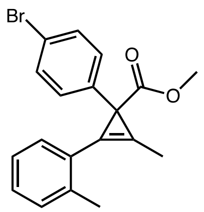

(±)-Methyl 1-(4-bromophenyl)-2-methyl-3-(*o*-tolyl)cycloprop-2-ene-1-carboxylate (±19f): General procedure A was followed using silver(I) triflate (51 mg, 0.10 mmol, 10 mol %), 1-methyl-2-(prop-1-yn-1-yl)benzene (521 mg, 4.0 mmol, 2.0 equiv) and CH_2Cl_2 (8 mL) added to a 100 mL round-bottom flask. Methyl 2-(4-bromophenyl)-2-diazoacetate (510 mg, 2.0 mmol, 1 equiv) in CH_2Cl_2 (40 mL) was added slowly *via* syringe pump over 2 hours and allowed to stir an additional 3 h. The reaction mixture was worked up as described in general procedure A and purified on silica gel (10% ethyl ether in pentane) to afford an off-white solid. The product was triturated using pentane and the pentane decanted to afford the product as a white solid. (398 mg, 56%).

^{1}H NMR (600 MHz, $CDCl_3$) δ 7.35 (d, *J* = 8.6 Hz, 2H), 7.32 (dd, *J* = 7.3, 1.1 Hz, 1H), 7.25 (dd, *J* = 5.2, 1.2 Hz, 2H), 7.23 – 7.17 (m, 3H), 3.67 (s, 3H), 2.49 (s, 3H), 2.37 (s, 3H).

^{13}C NMR (101 MHz, $CDCl_3$) δ 174.8, 140.2, 138.4, 131.1, 130.2, 129.9, 129.6, 129.3, 126.2, 125.5, 120.0, 111.7, 106.9, 52.0, 33.8, 20.9, 10.0.

HRMS (APCI) m/z: $[M+H]^+$ calc'd for $C_{19}H_{18}O_2{}^{79}Br$, 357.0485, found 357.0483

IIIe. Chiral Cyclopropene Synthesis

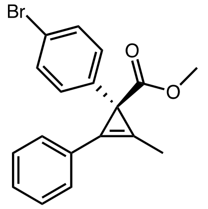

Methyl (*S*)-1-(4-bromophenyl)-2-methyl-3-phenylcycloprop-2-ene-1-carboxylate (16): General procedure B was followed using (*S*)-xylylBINAP(AuCl)$_2$ (216 mg, 0.18 mmol, 12 mol %), silver(I) hexafluoroantimonate(V) (52 mg, 0.10 mmol, 10 mol %), prop-1-yn-1-ylbenzene (0.92 mL, 7.5 mmol, 5.0 equiv) and CH_2Cl_2 (4 mL) added to a 50 mL round-bottom flask. Methyl 2-(4-bromophenyl)-2-diazoacetate (383 mg, 1.50 mmol, 1 equiv) in CH_2Cl_2 (16 mL) was added slowly *via* syringe pump over 2 hours and allowed to stir an additional 1 h. The reaction mixture was worked up as described in general procedure B and purified on silica gel (10% ethyl ether in pentane) to afford an off-white solid. (240 mg, 47%).

^{1}H NMR (600 MHz, $CDCl_3$) δ 7.51 (dd, *J* = 7.2, 1.2 Hz, 2H), 7.41 (td, *J* = 7.3, 1.1 Hz, 2H), 7.40 – 7.32 (m, 3H), 7.24 (dd, *J* = 8.6, 0.9 Hz, 2H), 3.69 (s, 3H), 2.37 (s, 3H).

^{1}H NMR spectrum is consistent with reported values.[9]

Chiral SFC: OJ-3 2% MeOH/iPrOH 0.2% formic acid 2.5 mL/min, λ=210 nm, Retention time (min) = 2.33 (major) and 4.46 (minor). 94.91:5.09 e.r.

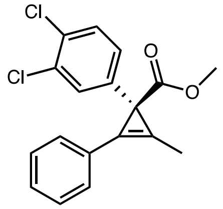

Methyl (*S*)-1-(3,4-dichlorophenyl)-2-methyl-3-phenylcycloprop-2-ene-1-carboxylate (22a): General procedure C was followed using (*S*)-xylylBINAP(AuCl)$_2$ (252 mg, 0.21 mmol, 12 mol %), silver(I) hexafluoroantimonate(V) (60 mg, 0.18 mmol, 10 mol %), prop-1-yn-1-ylbenzene (1.10 mL, 8.75 mmol, 5.0 equiv) and CH_2Cl_2 (4.8 mL) added to a 50 mL round-bottom flask. Methyl 2-diazo-2-(3,4-dichlorophenyl)acetate (429 mg, 1.75 mmol, 1 equiv) in CH_2Cl_2 (18 mL) was added slowly *via* syringe pump over 5 hours and allowed to stir an additional 11 h. The reaction mixture was worked up as described in general procedure C and purified on silica gel (15% ethyl ether in pentane) to afford a yellow oil (180 mg, 31%).

^{1}H NMR (600 MHz, $CDCl_3$) δ 7.50 (d, *J* = 7.0 Hz, 2H), 7.46 (d, *J* = 2.1 Hz, 1H), 7.42 (t, *J* = 7.4 Hz, 2H), 7.37 (t, *J* = 7.4 Hz, 1H), 7.31 (dd, *J* = 8.3, 0.7 Hz, 1H), 7.19 (dd, *J* = 8.4, 2.1 Hz, 1H), 3.70 (s, 3H), 2.37 (s, 3H).

^{1}H NMR spectrum is consistent with reported values.[9]

Chiral SFC: OJ-3 2% MeOH/iPrOH 0.2% formic acid 2.5 mL/min, λ=210 nm, Retention time (min) = 2.15 (major) and 3.87 (minor). 94.90:5.10 e.r.

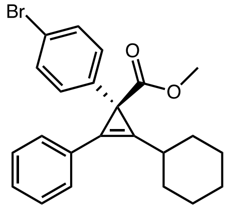

Methyl (*S*)-1-(4-bromophenyl)-2-cyclohexyl-3-phenylcycloprop-2-ene-1-carboxylate (22b): General procedure B was followed using (*S*)-xylylBINAP(AuCl)$_2$ (252 mg, 0.21 mmol, 12 mol %), silver(I) hexafluoroantimonate(V) (60 mg, 0.10 mmol, 10 mol %), (cyclohexylethynyl)benzene (1.61 g, 8.75 mmol, 5.00 equiv) and CH_2Cl_2 (4 mL) added to a 50 mL round-bottom flask. Methyl 2-(4-bromophenyl)-2-diazoacetate (446 mg, 1.75 mmol, 1 equiv) in CH_2Cl_2 (20 mL) was added slowly *via* syringe pump over

3 hours and allowed to stir an additional 13 h. The reaction mixture was worked up as described in general procedure B and purified on silica gel (10% ethyl ether in pentane) to afford a yellow oil. (360 mg, 50%).
^{1}H NMR (400 MHz, $CDCl_3$) δ 7.52 (d, *J* = 6.8 Hz, 2H), 7.45 – 7.38 (m, 2H), 7.39 – 7.31 (m, 3H), 7.24 (d, *J* = 8.6 Hz, 2H), 3.68 (s, 3H), 2.78 (tt, *J* = 10.6, 3.7 Hz, 1H), 2.06 – 1.93 (m, 2H), 1.82 – 1.59 (m, 3H), 1.55 – 1.19 (m, 5H).
^{13}C NMR (101 MHz, $CDCl_3$) δ 175.1, 140.7, 131.1, 130.1, 129.6, 129.1, 129.1, 126.3, 120.0, 119.1, 105.9, 52.1, 35.2, 34.9, 31.4, 31.1, 26.0, 25.7.
HRMS (APCI) m/z: $[M+H]^+$ calc'd for $C_{23}H_{23}O_2{}^{79}Br$, 411.0954, found 411.0955.
Chiral SFC: CEL1 2% MeOH/iPrOH 0.2% formic acid 2.5 mL/min, λ=210 nm, Retention time = 4.05 and 4.39.
$[\alpha]_D^{22}$ = -76.7 (*c* = 0.20, $CHCl_3$)

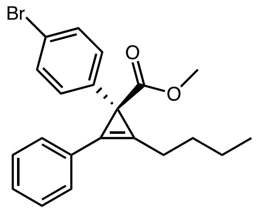

Methyl (*S*)-1-(4-bromophenyl)-2-butyl-3-phenylcycloprop-2-ene-1-carboxylate (22c): General procedure B was followed using (*S*)-xylylBINAP$(AuCl)_2$ (288 mg, 0.24 mmol, 12 mol %), silver(I) hexafluoroantimonate(V) (69 mg, 0.20 mmol, 10 mol %), hex-1-yn-1-ylbenzene (1.58 g, 10 mmol, 5 equiv) and CH_2Cl_2 (8 mL) added to a 100 mL round-bottom flask. Methyl 2-(4-bromophenyl)-2-diazoacetate (510 mg, 2.0 mmol, 1 equiv) in CH_2Cl_2 (32 mL) was added slowly *via* syringe pump over 3 hours and allowed to stir an additional 14 h. The reaction mixture was worked up as described in general procedure B and purified on silica gel (10% ethyl ether in pentane) to afford an off-white solid. (368 mg, 48%).
^{1}H NMR (600 MHz, $CDCl_3$) δ 7.50 (d, *J* = 6.9 Hz, 2H), 7.44 – 7.38 (m, 2H), 7.39 – 7.32 (m, 3H), 7.24 (d, *J* = 8.7 Hz, 2H), 3.68 (s, 3H), 2.71 (td, *J* = 7.4, 2.4 Hz, 2H), 1.72 (p, *J* = 7.4 Hz, 2H), 1.43 (dd, *J* = 7.2, 5.7 Hz, 1H), 1.41 (dd, *J* = 7.4, 5.6 Hz, 1H), 0.93 (t, *J* = 7.4 Hz, 2H).
^{13}C NMR (101 MHz, $CDCl_3$) δ 174.8, 140.4, 131.1, 129.9, 129.3, 129.0, 129.0, 126.2, 120.0, 115.2, 107.2, 52.0, 34.8, 29.6, 24.5, 22.5, 13.7.
HRMS (APCI) m/z: $[M+H]^+$ calc'd for $C_{21}H_{22}O_2{}^{79}Br$, 385.0798, found 385.0797
Chiral SFC: OJ-3 2% MeOH/iPrOH 0.2% formic acid 2.5 mL/min, λ=210 nm, Retention time = 1.58 and 3.37.
$[\alpha]_D^{22}$ = -62.9 (*c* = 0.80, $CHCl_3$)

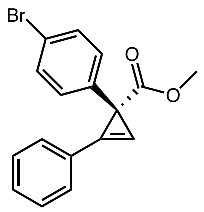

Methyl (*S*)-1-(4-bromophenyl)-2-phenylcycloprop-2-ene-1-carboxylate (22d): Into an oven dried round bottom flask was added an oven dried stir bar, Rh_2(*S*-DOSP)$_4$ (38 mg, 0.02 mmol, 1 mol %), and 1 gram of activated 4 Å molecular sieves. This was then sealed with a rubber septum, and the atmosphere exchanged by drawing a vacuum and backfilling with nitrogen three times. To this was then added hexanes (ACS grade) (40 mL), and phenylacetylene (2.2 mL, 20 mmol, 10 equiv). Methyl 2-(4-bromophenyl)-2-diazoacetate in hexanes (24 mL) was added slowly *via* syringe pump over 5 hours and was allowed to stir overnight. The reaction was worked up by filtering through a celite plug and concentrated under reduced pressure. The crude residue was then purified on silica gel (15% ethyl ether in pentane) as a white solid. (384 mg, 58%).
^{1}H NMR (400 MHz, $CDCl_3$) δ 7.62 – 7.56 (m, 2H), 7.48 – 7.37 (m, 5H), 7.28 (d, *J* = 2.0 Hz, 2H), 7.17 (s, 1H), 3.71 (s, 3H).
^{1}H NMR spectrum is consistent with reported values[11]

Chiral SFC: OZ-3 3% EtOH/MeCN 0.2% formic acid 2.5 mL/min λ=210 nm, Retention time = 3.74 (minor) and 3.97 (major), 96.1:3.9 e.r. (Note: This reflects the e.r. of the solid substrate, which appears to be enantioenriched compared to a solution made from the mixture of enantiomers).

IV. Preparation of Products from Substate Table

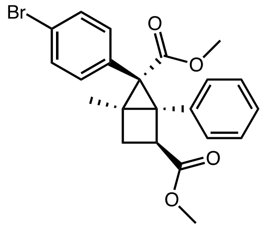

Dimethyl (1*R*,2*S*,4*R*,5*S*)-5-(4-bromophenyl)-4-methyl-1-phenylbicyclo[2.1.0]pentane-2,5-dicarboxylate (18a): General procedure D was followed using methyl (*S*)-1-(4-bromophenyl)-2-methyl-3-phenylcycloprop-2-ene-1-carboxylate (172 mg, 0.500 mmol, 1 equiv), Ir(dF(CF_3)ppy)$_2$(tbbpy)PF_6 (5.6 mg, 0.50 mmol, 1 mol %), and methyl acrylate (0.23 mL, 2.5 mmol, 5.0 equiv) and MeCN (10 mL) added to a 15 mL screw-top test tube. The reaction was cooled to -40 ºC and irradiated with a blue LED. The reaction mixture was worked up as described in general procedure D and purified on silica gel (17% ethyl ether in pentane) to afford the product as an amorphous white solid. (140 mg, 65%).

1 mmol (±18a): General procedure D was followed using methyl 1-(4-bromophenyl)-2-methyl-3-phenylcycloprop-2-ene-1-carboxylate (343 mg, 1.00 mmol, 1 equiv), Ir(dF(CF_3)ppy)$_2$(tbbpy)PF_6 (11.2 mg, 0.010 mmol, 1 mol %), methyl acrylate (0.45 mL, 5.0 mmol, 5.0 equiv) and MeCN (20 mL) using a 25 mL screw cap test tube. The reaction was cooled to -40 ºC and irradiated with a blue LED. The reaction mixture was worked up as described in general procedure D and purified on silica gel (17% ethyl ether in pentane) to afford the product as an amorphous white solid. (265 mg, 62%).

Telescoped Reaction (±18a): To a 25 mL flame dried round bottom flask was added an oven dried stir bar and silver(I) triflate (13 mg, 0.050 mmol, 10 mol %). The round bottom flask was then wrapped in aluminum foil to exclude light. The atmosphere of the flask was exchanged with nitrogen *via* vacuum followed by backfilling with nitrogen. This was done three times. To this was added CH_2Cl_2 (2 mL) and prop-1-yn-1-ylbenzene (125 µL, 1.00 mmol, 2 equiv). Methyl 2-(4-bromophenyl)-2-diazoacetate (128 mg, 0.500 mmol, 1 equiv) was dissolved in CH_2Cl_2 (8 mL) and added slowly *via* a syringe pump over 2 hours. This was stirred for 1 additional hour. The reaction was worked up by filtering through a celite pad, washing with CH_2Cl_2. This was concentrated *in vacuo.* The crude residue was examined by NMR in $CDCl_3$ and again concentrated *in vacuo*. To an oven dried screw cap test was added an oven dried stir bar and Ir(dF(CF_3)ppy)$_2$(tbbpy)PF_6 (5.6 mg, 0.005 mmol, 1 mol %). The crude reaction residue was dissolved in MeCN (4 mL) and transferred to the screw cap test tube. The vial containing the residue was then washed with MeCN (6 mL) and that was transferred to the screw cap test tube. This was sealed with a cap and PTFE/silicone septum. The perimeter of the cap was then wrapped with electrical tape. The reaction mixture was sparged with nitrogen for 15 minutes. After 15 minutes, methyl acrylate (0.23 mL, 3.0 mmol, 5.0 equiv) was added to the reaction mixture. The top of the cap was then sealed with electrical tape, and a second layer of electrical tape was added around the perimeter. The test tube was then placed into a -40 ºC bath and irradiated with blue LED for 2 hours. After 2 hours, the reaction was transferred to a round bottom flask and the test tube washed with ethyl acetate three times. This was concentrated *in vacuo.* The crude residue was then purified on silica gel (17% ethyl ether in pentane) to afford the product as an amorphous white solid. (110 mg, 51% over two steps).

^{1}H NMR (600 MHz, $CDCl_3$, 223 K) δ 7.54 (dd, *J* = 8.1, 2.1 Hz, 1H), 7.53 – 7.48 (m, 1H), 7.49 (d, *J* = 8.3 Hz, 2H), 7.42 (t, *J* = 7.7 Hz, 3H), 7.39 (dd, *J* = 8.3, 2.3 Hz, 1H), 7.34 (t, *J* = 7.3 Hz, 1H), 7.19 (dd, *J* = 8.2, 2.3 Hz, 1H), 3.36 (s, 5H), 3.30 (dd, *J* = 10.4, 4.3 Hz, 1H), 2.40 (dd, *J* = 12.5, 10.4 Hz, 1H), 2.05 (dd, *J* = 12.5, 4.4 Hz, 1H), 1.76 (s, 3H).

^{13}C NMR (101 MHz, $CDCl_3$, 294 K) δ 172.5, 170.2, 138.4, 132.7, 131.0, 129.1, 128.3, 127.2, 121.8, 52.1, 51.2, 47.1, 46.8, 42.5, 35.5, 30.2, 14.3. (Two carbons could not be resolved at room temperature).

HRMS (APCI) m/z: $[M+H]^+$ calc'd for $C_{22}H_{22}O_4{}^{79}Br$, 429.0696, found 429.0700.

Chiral SFC: CEL1 2% EtOH/iPrOH 0.2% formic acid 2.5 mL/min, λ=210 nm, Retention time = 4.02 (major) and 4.53 (minor), 95.75:4.25 e.r.

$[\alpha]_D^{22}$ = -17.8 (*c* = 0.73, $CHCl_3$)

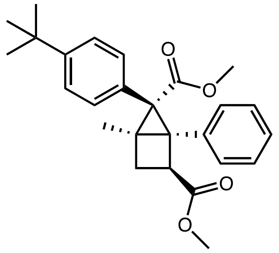

(±)-Dimethyl 5-(4-(*tert*-butyl)phenyl)-4-methyl-1-phenylbicyclo[2.1.0]pentane-2,5-dicarboxylate (±21a): General procedure D was followed using methyl 1-(4-(tert-butyl)phenyl)-2-methyl-3-phenylcycloprop-2-ene-1-carboxylate (160 mg, 0.50 mmol, 1 equiv), $Ir(dF(CF_3)ppy)_2(tbbpy)PF_6$ (5.6 mg, 0.005 mmol, 1 mol %), and methyl acrylate (0.23 mL, 2.5 mmol, 5.0 equiv) and MeCN (10 mL) added to a 15 mL screw-top test tube. The reaction was cooled to -40 ºC and irradiated with a blue LED. The reaction mixture was worked up as described in general procedure D and purified on silica gel (15% ethyl ether in pentane) to afford the product as a colorless oil. (99 mg, 49%).
^{1}H NMR (600 MHz, $CDCl_3$, 223 K) δ 7.55 (d, J = 6.9 Hz, 2H), 7.47 – 7.39 (m, 5H), 7.35 (t, J = 7.4 Hz, 1H), 7.24 (d, J = 6.6 Hz, 1H), 3.39 (s, 3H), 3.28 (dd, J = 10.3, 4.3 Hz, 1H), 3.24 (s, 3H), 2.38 (dd, J = 12.2, 10.4 Hz, 1H), 2.12 (dd, J = 12.2, 4.3 Hz, 1H), 1.79 (s, 3H), 1.35 (s, 9H).
^{13}C NMR (101 MHz, $CDCl_3$, 298 K) δ 172.7, 171.0, 150.3, 139.1, 130.4, 129.1, 128.2, 127.0, 124.7, 52.0, 51.1, 47.0, 46.8, 42.5, 35.2, 34.7, 31.5, 30.3, 14.6. (Two carbons could not be resolved at room temperature).
HRMS (APCI) m/z: $[M+H]^+$ calc'd for $C_{26}H_{31}O_4$, 407.2217, found 407.2222.

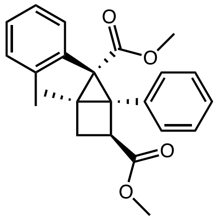

(±)-Dimethyl 4-methyl-1-phenyl-5-(*o*-tolyl)bicyclo[2.1.0]pentane-2,5-dicarboxylate (±21b): General procedure D was followed using methyl 2-methyl-3-phenyl-1-(o-tolyl)cycloprop-2-ene-1-carboxylate (139 mg, 0.50 mmol, 1 equiv), $Ir(dF(CF_3)ppy)_2(tbbpy)PF_6$ (5.6 mg, 0.005 mmol, 1 mol %), and methyl acrylate (0.23 mL, 2.5 mmol, 5.0 equiv) and MeCN (10 mL) added to a 15 mL screw-top test tube. The reaction was cooled to -40 ºC and irradiated with a blue LED. The reaction mixture was worked up as described in general procedure D and purified on silica gel (22% ethyl ether in pentane) to afford the product as an amorphous white solid. (104 mg, 57%).
^{1}H NMR (600 MHz, $CDCl_3$, 223 K) δ 7.52 (dd, *J* = 6.9, 1.2 Hz, 2H), 7.50 (d, *J* = 7.7 Hz, 1H), 7.44 (t, *J* = 7.5 Hz, 2H), 7.36 (t, *J* = 7.4 Hz, 1H), 7.33 – 7.27 (m, 2H), 7.23 (td, *J* = 7.2, 1.9 Hz, 1H), 3.46 (s, 3H), 3.38 (s, 3H), 3.33 (dd, *J* = 10.1, 4.9 Hz, 1H), 2.31 – 2.22 (m, 4H), 1.79 (s, 3H), 1.67 (dd, *J* = 12.3, 4.9 Hz, 1H).
^{13}C NMR (101 MHz, $CDCl_3$, 298 K) δ 172.9, 170.6, 138.9, 138.7, 133.6, 133.0, 130.8, 129.2, 128.3, 127.8, 127.1, 125.2, 52.1, 51.3, 46.7, 46.1, 42.5, 36.1, 29.4, 20.0, 13.9.
HRMS (APCI) m/z: $[M+H]^+$ calc'd for $C_{23}H_{25}O_4$, 365.1747, found 365.1747.

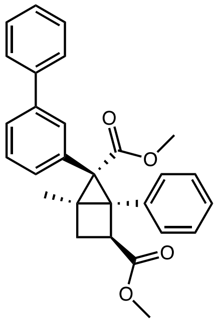

(±)-Dimethyl 5-([1,1'-biphenyl]-3-yl)-4-methyl-1-phenylbicyclo[2.1.0]pentane-2,5-dicarboxylate (±21c): General procedure D was followed using methyl 1-([1,1'-biphenyl]-3-yl)-2-methyl-3-phenylcycloprop-2-ene-1-carboxylate (170 mg, 0.50 mmol, 1 equiv), $Ir(dF(CF_3)ppy)_2(tbbpy)PF_6$ (5.6 mg,

0.005 mmol, 1 mol %), and methyl acrylate (0.23 mL, 2.5 mmol, 5.0 equiv) and MeCN (10 mL) added to a 15 mL screw-cap test tube. The reaction was cooled to -40 ºC and irradiated with a blue LED. The reaction mixture was worked up as described in general procedure D and purified on silica gel (20% ethyl ether in pentane) to afford the product as an amorphous white solid. (134 mg, 63%).
^{1}H NMR (400 MHz, $CDCl_3$, 328 K) δ 7.70 (d, *J* = 7.6 Hz, 3H), 7.59 – 7.52 (m, 3H), 7.50 – 7.42 (m, 3H), 7.42 – 7.34 (m, 4H), 7.29 (d, *J* = 7.2 Hz, 1H), 3.35 – 3.29 (m, 4H), 3.16 (br s, 3H), 2.35 (dd, *J* = 12.2, 10.4 Hz, 1H), 2.20 (dd, *J* = 12.2, 4.6 Hz, 1H), 1.85 (s, 3H).
^{13}C NMR (101 MHz, $CDCl_3$, 292 K) δ 172.7, 170.7, 138.8, 129.1, 128.9, 128.4, 128.2, 127.4, 127.3, 127.1, 126.3, 52.1, 51.2, 47.5, 47.0, 42.5, 35.4, 30.3, 14.5. (6 carbons could not be resolved at room temperature).
HRMS (APCI) m/z: $[M+H]^+$ calc'd for $C_{28}H_{27}O_4$, 427.1904, found 427.1907.

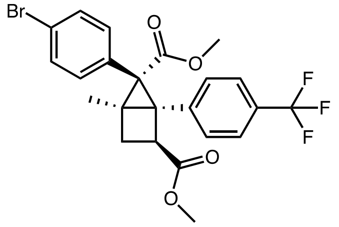

(±)-Dimethyl 5-(4-bromophenyl)-4-methyl-1-(4-(trifluoromethyl)phenyl)bicyclo[2.1.0]pentane-2,5-dicarboxylate (±21d): General procedure D was followed using methyl 1-(4-bromophenyl)-2-methyl-3-(4-(trifluoromethyl)phenyl)cycloprop-2-ene-1-carboxylate (206 mg, 0.50 mmol, 1 equiv), $Ir(dF(CF_3)ppy)_2(tbbpy)PF_6$ (5.6 mg, 0.005 mmol, 1 mol %), and methyl acrylate (0.23 mL, 2.5 mmol, 5.0 equiv) and MeCN (10 mL) added to a 15 mL screw-cap test tube. The reaction was cooled to -40 ºC and irradiated with a blue LED. The reaction mixture was worked up as described in general procedure D and purified on silica gel (17% ethyl ether in pentane) to afford the product as an amorphous off-white solid. (169 mg, 68%).
^{1}H NMR (600 MHz, $CDCl_3$, 223 K) δ 7.68 (d, *J* = 8.1 Hz, 2H), 7.63 (d, *J* = 8.1 Hz, 2H), 7.58 (dd, *J* = 8.1, 2.1 Hz, 1H), 7.54 (dd, *J* = 8.2, 2.1 Hz, 1H), 7.38 (dd, *J* = 8.2, 2.2 Hz, 1H), 7.23 (dd, *J* = 8.1, 2.3 Hz, 1H), 3.42 (s, 3H), 3.37 (s, 3H), 3.29 (dd, *J* = 10.4, 4.3 Hz, 1H), 2.46 (dd, *J* = 12.5, 10.3 Hz, 1H), 2.11 (dd, *J* = 12.5, 4.3 Hz, 1H), 1.77 (s, 3H).
^{13}C NMR (101 MHz, $CDCl_3$, 292 K) δ 172.1, 170.0, 142.5, 132.1, 131.1, 129.4, 129.2 (q, *J* = 32.5 Hz), 125.3 (q, *J* = 3.8 Hz), 124.3 (q, *J* = 272.2 Hz), 122.0, 52.3, 51.3, 46.9, 46.4, 42.1, 35.8, 29.9, 14.0. (Two carbons could not be resolved at room temperature).
^{19}F NMR (376 MHz, $CDCl_3$, 297 K) δ -62.43.
HRMS (APCI) m/z: $[M+H]^+$ calc'd for $C_{23}H_{21}O_4{}^{79}BrF_3$, 497.0570, found 497.0573.

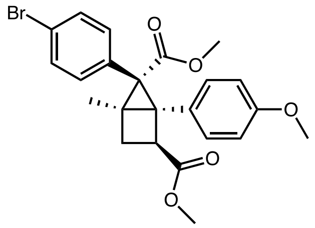

(±)-Dimethyl 5-(4-bromophenyl)-1-(4-methoxyphenyl)-4-methylbicyclo[2.1.0]pentane-2,5-dicarboxylate (±21e): General procedure D was followed using methyl 1-(4-bromophenyl)-2-(4-methoxyphenyl)-3-methylcycloprop-2-ene-1-carboxylate (191 mg, 0.51 mmol, 1 equiv), $Ir(dF(CF_3)ppy)_2(tbbpy)PF_6$ (5.7 mg, 0.005 mmol, 1 mol %), and methyl acrylate (0.23 mL, 2.6 mmol, 5.0 equiv) and MeCN (10 mL) added to a 15 mL screw-cap test tube. The reaction was cooled to -40 ºC and irradiated with a blue LED. The reaction mixture was worked up as described in general procedure D and purified on silica gel (32% ethyl ether in pentane) to afford the product as an amorphous off-white solid. (143 mg, 61%).
^{1}H NMR (600 MHz, $CDCl_3$, 253 K) δ 7.51 (d, J = 8.2 Hz, 1H), 7.49 (d, J = 8.3 Hz, 1H), 7.42 (d, J = 8.6 Hz, 2H), 7.39 (dd, J = 8.2, 2.2 Hz, 1H), 7.17 (dd, J = 8.2, 2.2 Hz, 1H), 6.92 (d, J = 8.7 Hz, 2H), 3.82 (s, 3H), 3.36 (s, 3H), 3.34 (s, 3H), 3.26 (dd, J = 10.3, 4.4 Hz, 1H), 2.35 (dd, J = 12.4, 10.4 Hz, 1H), 2.03 (dd, J = 12.4, 4.4 Hz, 1H), 1.77 (s, 3H).
^{13}C NMR (101 MHz, $CDCl_3$, 292 K) δ 172.6, 170.3, 158.6, 132.8, 130.5, 130.2, 121.7, 113.7, 55.3, 52.2, 51.2, 46.7, 46.7, 42.7, 35.4, 30.1, 14.3. (Four carbons could not be resolved at room temperature).

HRMS (APCI) m/z: $[M+H]^+$ calc'd for $C_{23}H_{24}O_5{}^{79}Br$, 459.0802, found 459.0806.

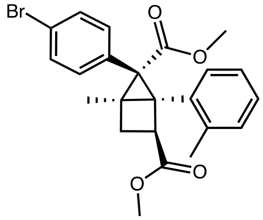

(±)-Dimethyl 5-(4-bromophenyl)-4-methyl-1-(*o*-tolyl)bicyclo[2.1.0]pentane-2,5-dicarboxylate (±21f): General procedure D was followed using methyl 1-(4-bromophenyl)-2-methyl-3-(o-tolyl)cycloprop-2-ene-1-carboxylate (179 mg, 0.50 mmol, 1 equiv), $Ir(dF(CF_3)ppy)_2(tbbpy)PF_6$ (5.6 mg, 0.005 mmol, 1 mol %), and methyl acrylate (0.23 mL, 2.5 mmol, 5.0 equiv) and MeCN (10 mL) added to a 15 mL screw-cap test tube. The reaction was cooled to -40 ºC and irradiated with a blue LED. The reaction mixture was worked up as described in general procedure D and purified on silica gel (20% ethyl ether in pentane) to afford the product as an amorphous off-white solid. (169 mg, 76%).

^{1}H NMR (600 MHz, $CDCl_3$, 253 K) δ 7.56 (m, 0.3H, minor rotamer) 7.52 (ddd, *J* = 12.3, 8.2, 2.1 Hz, 1.7H, major rotamer), 7.48 (d, *J* = 8.0 Hz, 0.3H), 7.44 (dd, *J* = 8.2, 2.2 Hz, 0.7H, major rotamer), 7.39 (dd, *J* = 7.2, 1.5 Hz, 0.7H, major rotamer), 7.29-7.27 (m, 0.3H, minor rotamer), 7.25 – 7.18 (m, 2H, mixture of rotamers), 7.25 – 7.18 (m, 3H, mixture of rotamers), 7.16 (dd, *J* = 8.1, 2.3 Hz, 1H, mixture of rotamers), 3.41 (s, 0.8H, minor rotamer), 3.39 – 3.34 (m, 2.8H, mixture of rotamers), 3.39 – 3.35 (m, 0.8H, major rotamer) 3.34 (s, 0.8H, minor rotamer), 3.33 – 3.31 (m, 0.2H, minor rotamer), 3.30 (s, 2H, major rotamer), 2.62 (s, 0.8H, minor rotamer), 2.47 (s, 2.1H, major rotamer), 2.41 (dd, *J* = 12.5, 10.3 Hz, 1H, mixture of rotamers), 2.10 (dd, *J* = 12.5, 4.1 Hz, 0.7H, major rotamer), 1.96 (dd, *J* = 12.5, 4.6 Hz, 0.3H, minor rotamer), 1.78 (s, 2.1H, major rotamer), 1.65 (s, 0.8H, minor rotamer).

^{13}C NMR (151 MHz, $CDCl_3$, 298 K) δ 172.4, 170.8, 136.9, 135.9, 135.3, 133.3, 132.9, 131.4, 131.1, 130.8, 130.2, 127.5, 126.2, 125.4, 121.8, 52.2, 51.2, 45.4, 43.8, 40.3, 39.3, 34.8, 30.5, 20.9, 19.9, 16.4, 14.1. Mixture of rotamers.

HRMS (APCI) m/z: $[M+H]^+$ calc'd for $C_{23}H_{24}O_4{}^{79}Br$, 443.0853, found 443.0847.

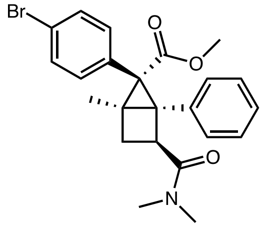

(±)-Methyl 5-(4-bromophenyl)-2-(dimethylcarbamoyl)-4-methyl-1-phenylbicyclo[2.1.0]pentane-5-carboxylate (±21g): General procedure D was followed using methyl 1-(4-bromophenyl)-2-methyl-3-phenyl-cycloprop-2-ene-1-carboxylate (172 mg, 0.50 mmol, 1 equiv), $Ir(dF(CF_3)ppy)_2(tbbpy)PF_6$ (5.6 mg, 0.005 mmol, 1 mol %), and dimethyl acrylamide (0.26 mL, 2.5 mmol, 5.0 equiv) and MeCN (10 mL) added to a 15 mL screw-cap test tube. The reaction was cooled to -20 ºC and irradiated with a blue LED. The reaction mixture was worked up as described in general procedure D and purified on silica gel (20% ethyl ether in hexanes) to afford the major diastereomer product as an amorphous white solid. (221 mg, 75%).

^{1}H NMR (500 MHz, $CDCl_3$, 238 K) δ 7.70 (d, *J* = 7.4 Hz, 1H), 7.55 (d, *J* = 6.9 Hz, 2H), 7.47 (t, *J* = 6.9 Hz, 2H), 7.37 (t, *J* = 7.5 Hz, 2H), 7.33 – 7.24 (m, 1H), 7.14 (d, *J* = 7.6 Hz, 1H), 3.43 (dd, *J* = 10.4, 4.9 Hz, 1H), 3.36 (s, 3H), 2.54 (s, 3H), 2.51 – 2.43 (m, 3H), 2.46 (s, 3H), 2.08 (dd, *J* = 11.8, 5.0 Hz, 1H), 1.79 (s, 3H).

^{13}C NMR (126 MHz, $CDCl_3$, 298 K) δ 170.9, 170.7, 138.8, 133.1, 130.5, 129.9, 128.1, 127.1, 121.4, 52.1, 48.1, 46.6, 43.8, 36.7, 35.3, 33.2, 14.1. (Three carbons could not be resolved at room temperature).

HRMS (ESI) m/z: [M+H]+ calc'd for $C_{23}H_{25}{}^{79}BrNO_3$, 442.1012, found 442.1016.

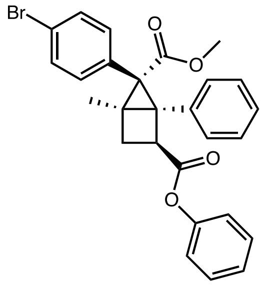

(±)-5-methyl 2-phenyl 5-(4-bromophenyl)-4-methyl-1-phenylbicyclo[2.1.0]pentane-2,5-dicarboxylate (±21h): General procedure D was followed using methyl 1-(4-bromophenyl)-2-methyl-3-phenylcycloprop-2-ene-1-carboxylate (±16) (171.7 mg, 0.500 mmol, 1 equiv.), Ir(dF(CF$_3$)ppy)$_2$(tbbpy)PF$_6$ (5.6 mg, 0.005 mmol, 1 mol %), and phenyl acrylate (0.350 mL, 2.50 mmol, 5.0 equiv.) and MeCN (10 mL) added to a 10 mL screw-top test tube. The reaction was cooled to -40 ºC and irradiated with a blue LED chip. The reaction mixture was worked up as described in general procedure D and purified on silica gel (0% to 20% ether/hexanes) to afford the product as an amorphous white solid (191 mg, 86%).
^{1}H NMR (500 MHz, CDCl$_3$, 238 K) δ 7.59 (dd, *J* = 8.2, 2.1 Hz, 1H), 7.57 – 7.51 (m, 2H), 7.49 (dd, *J* = 8.2, 2.2 Hz, 1H), 7.42 – 7.35 (m, 3H), 7.36 – 7.29 (m, 4H), 7.19 (t, *J* = 7.5 Hz, 1H), 6.62 – 6.58 (m, 2H), 3.60 (dd, *J* = 10.5, 4.4 Hz, 1H), 3.35 (s, 3H), 2.54 (dd, *J* = 12.5, 10.5 Hz, 1H), 2.23 (dd, *J* = 12.4, 4.5 Hz, 1H), 1.84 (s, 3H).
^{13}C NMR (126 MHz, CDCl$_3$, 298 K) δ 170.5, 170.2, 150.2, 138.3, 132.6, 131.5, 129.3, 129.2, 128.3, 127.3, 125.8, 122.2, 121.3, 52.2, 47.1, 47.0, 42.8, 35.5, 30.8, 14.3. (Two carbons could not be resolved at room temperature).
HRMS (ESI) m/z: [M+H]$^+$ calc'd for $C_{27}H_{24}{}^{79}BrO_4$, 491.0852, found 491.0854.

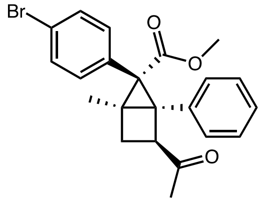

(±)-Methyl 2-acetyl-5-(4-bromophenyl)-4-methyl-1-phenylbicyclo[2.1.0]pentane-5-carboxylate (±21i): General procedure D was followed using methyl 1-(4-bromophenyl)-2-methyl-3-phenylcycloprop-2-ene-1-carboxylate (±16) (171.7 mg, 0.500 mmol, 1 equiv.), Ir(dF(CF$_3$)ppy)$_2$(tbbpy)PF$_6$ (5.6 mg, 0.005 mmol, 1 mol %), and methyl vinyl ketone (0.210 mL, 2.50 mmol, 5.0 equiv.) and MeCN (10 mL) added to a 10 mL screw-top test tube. The reaction was cooled to -40 ºC and irradiated with a blue LED chip. The reaction mixture was worked up as described in general procedure D and purified on silica gel (0% to 35% ether/hexanes) to afford the product as an amorphous white solid (112 mg, 54%).
^{1}H NMR (500 MHz, CDCl$_3$, 238 K) δ 7.53 – 7.48 (m, 2H), 7.47 (d, *J* = 7.6 Hz, 2H), 7.40 (t, *J* = 7.5 Hz, 2H), 7.35 – 7.29 (m, 2H), 7.17 (dd, *J* = 8.1, 2.3 Hz, 1H), 3.36 (s, 3H), 3.16 (dd, *J* = 10.1, 4.5 Hz, 1H), 2.37 (dd, *J* = 12.3, 10.2 Hz, 1H), 2.08 (dd, *J* = 12.3, 4.5 Hz, 1H), 1.73 (s, 3H), 1.52 (s, 3H).
^{13}C NMR (126 MHz, CDCl$_3$, 298 K) δ 208.7, 170.3, 138.3, 133.4, 131.2, 129.4, 128.5, 127.3, 121.9, 52.2, 51.1, 49.1, 46.6, 34.9, 30.6, 28.0, 14.2. (Two carbons could not be resolved at room temperature).
HRMS (ESI) m/z: [M+H]$^+$ calc'd for $C_{22}H_{22}{}^{79}BrO_3$ 413.0747, found 413.0760.

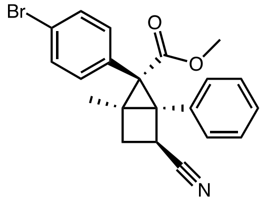

(±)-Methyl 5-(4-bromophenyl)-2-cyano-4-methyl-1-phenylbicyclo[2.1.0]pentane-5-carboxylate (±21j): General procedure D was followed using methyl 1-(4-bromophenyl)-2-methyl-3-phenyl-cycloprop-2-ene-1-carboxylate (172 mg, 0.50 mmol, 1 equiv), Ir(dF(CF$_3$)ppy)$_2$(tbbpy)PF$_6$ (5.6 mg, 0.005 mmol, 1 mol %), and acrylonitrile (0.165 mL, 2.5 mmol, 5.0 equiv) and MeCN (10 mL) added to a 15 mL screw-cap test tube. The reaction was cooled to -20 ºC and irradiated with a blue LED. The reaction

mixture was worked up as described in general procedure D and purified on silica gel (20% ethyl ether in hexanes) to afford the major diastereomer product as an amorphous white solid (108 mg, 55%) and the minor diastereomer as an amorphous white solid (56 mg, 28%).

Major Diastereomer

^{1}H NMR (500 MHz, $CDCl_3$, 238 K) δ 7.71 (dd, J = 8.3, 2.2 Hz, 1H), 7.65 (dd, J = 8.2, 2.1 Hz, 1H), 7.56 (dd, J = 8.2, 2.1 Hz, 1H), 7.46 – 7.32 (m, 5H), 7.18 (dd, J = 8.2, 2.3 Hz, 1H), 3.45 (dd, J = 10.3, 4.6 Hz, 1H), 3.37 (s, 3H), 2.51 (dd, J = 12.3, 10.4 Hz, 1H), 2.00 (dd, J = 12.3, 4.6 Hz, 1H), 1.80 (s, 3H).

^{13}C NMR (126 MHz, $CDCl_3$, 298 K) δ 169.5, 137.1, 131.6, 130.4, 128.8, 128.2, 128.0, 122.8, 117.9, 52.4, 47.1, 43.9, 38.2, 31.2, 27.3, 14.2. (Two carbons could not be resolved at room temperature).

HRMS (ESI): m/z $[M+H]^+$ calc'd for $C_{21}H_{19}{}^{79}BrNO_2$, 396.0594, found 396.0600

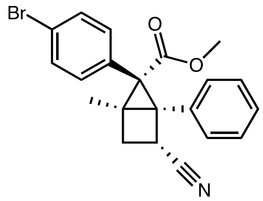

Minor Diastereomer

^{1}H NMR (500 MHz, $CDCl_3$, 238 K) δ 7.65 (dd, J = 8.1, 2.1 Hz, 1H), 7.61 (dd, J = 8.1, 2.2 Hz, 1H), 7.48 – 7.36 (m, 6H), 7.20 (dd, J = 8.2, 2.2 Hz, 1H), 3.40 (s, 3H), 2.65 (dd, J = 5.9, 4.3 Hz, 1H), 2.45 (dd, J = 12.2, 4.4 Hz, 1H), 1.93 (dd, J = 12.2, 6.0 Hz, 1H), 1.87 (s, 3H).

^{13}C NMR (126 MHz, $CDCl_3$, 298 K) δ 169.3, 133.9, 133.1, 132.2, 131.9, 129.4, 128.6, 128.4, 122.4, 120.1, 52.4, 46.7, 45.9, 39.8, 32.9, 28.4, 14.6.

HRMS (ESI): m/z $[M+H]^+$ calc'd for $C_{21}H_{19}{}^{79}BrNO_2$, 396.0594, found 396.0608

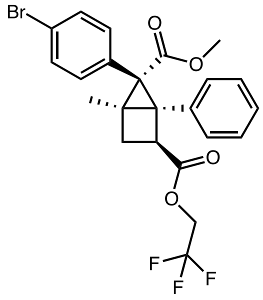

(±)-5-methyl 2-(2,2,2-trifluoroethyl) 5-(4-bromophenyl)-4-methyl-1-phenylbicyclo[2.1.0]pentane-2,5-dicarboxylate (±21k): General procedure D was followed using methyl 1-(4-bromophenyl)-2-methyl-3-phenylcycloprop-2-ene-1-carboxylate (±16) (171.7 mg, 0.500 mmol, 1 equiv.), $Ir(dF(CF_3)ppy)_2(tbbpy)PF_6$ (5.6 mg, 0.005 mmol, 1 mol %), and 2,2,2-trifluoroethyl acrylate (0.320 mL, 2.50 mmol, 5.0 equiv.) and MeCN (10 mL) added to a 10 mL screw-top test tube. The reaction was cooled to -40 °C and irradiated with a blue LED chip. The reaction mixture was worked up as described in general procedure D and purified on silica gel (0% to 25% ether/hexanes) to afford the product as an amorphous white solid (192 mg, 77%).

^{1}H NMR (500 MHz, $CDCl_3$, 238 K) δ 7.54 (d, J = 8.2 Hz, 1H), 7.49 – 7.44 (m, 3H), 7.44 – 7.37 (m, 3H), 7.33 (t, J = 7.4 Hz, 1H), 7.20 (d, J = 8.1 Hz, 1H), 4.19 (dd, J = 12.4, 8.0 Hz, 1H), 3.83 (dd, J = 12.4, 8.0 Hz, 1H), 3.39 – 3.32 (m, 4H), 2.44 (dd, J = 12.1, 10.4 Hz, 1H), 2.11 (dd, J = 12.5, 4.2 Hz, 1H), 1.78 (s, 3H).

^{13}C NMR (126 MHz, $CDCl_3$, 238 K) δ 170.6, 170.0, 137.5, 134.5, 133.2, 132.1, 131.6, 130.6, 128.9, 128.4, 127.5, 126.7, 122.9 (q, J = 276.7 Hz), 122.1, 60.6 (q, J = 36.1 Hz), 52.7, 47.3, 46.1, 41.9, 35.7, 29.6, 14.0.

^{19}F NMR (376 MHz, $CDCl_3$) δ -73.30 (t, J = 8.6 Hz).

HRMS (ESI): m/z $[M+H]^+$ calc'd for $C_{23}H_{20}{}^{79}BrF_3O_4$, 497.0570, found 497.0577.

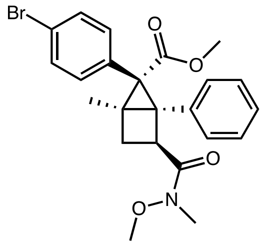

(±)-Methyl 5-(4-bromophenyl)-2-(methoxy(methyl)carbamoyl)-4-methyl-1-phenylbicyclo[2.1.0]pentane-5-carboxylate (±21l): General procedure D was followed using methyl 1-(4-bromophenyl)-2-methyl-3-phenylcycloprop-2-ene-1-carboxylate (±16) (171.7 mg, 0.500 mmol, 1 equiv.), Ir(dF(CF$_3$)ppy)$_2$(tbbpy)PF$_6$ (5.6 mg, 0.005 mmol, 1 mol %), and *N*-methoxy-*N*-methylacrylamide (0.320 mL, 2.50 mmol, 5.0 equiv.) and MeCN (10 mL) added to a 10 mL screw-top test tube. The reaction was cooled to -40 ºC and irradiated with a blue LED chip. The reaction mixture was worked up as described in general procedure D. The crude material was purified using a combination of silica gel flash column chromatography (0% to 25% acetone/hexanes), preparative thin-layer chromatography (10% acetone/hexanes), and trituration in hexanes to afford the product as an amorphous white solid (99 mg, 43%).

^{1}H NMR (500 MHz, CDCl$_3$, 238 K) δ 7.53 (dd, *J* = 8.2, 1.9 Hz, 1H), 7.50 – 7.42 (m, 4H), 7.38 (t, *J* = 7.5 Hz, 2H), 7.30 (d, *J* = 7.5 Hz, 1H), 7.21 (dd, *J* = 8.2, 2.0 Hz, 1H), 3.42 (s, 3H), 3.43 – 3.38 (m, 1H), 2.88 (s, 3H), 2.84 (s, 3H), 2.42 (dd, *J* = 12.5, 10.3 Hz, 1H), 2.18 (dd, *J* = 12.6, 4.6 Hz, 1H), 1.68 (s, 3H).

^{13}C NMR (126 MHz, CDCl$_3$, 238 K) δ 171.4, 170.8, 138.0, 134.6, 133.4, 133.0, 131.1, 130.5, 130.1, 127.8, 126.9, 121.5, 59.9, 52.5, 48.7, 45.5, 43.8, 35.5, 32.3, 30.1, 13.8.

HRMS (ESI): m/z [M+H]$^+$ calc'd for $C_{23}H_{25}{}^{79}BrNO_4$, 458.0961, found 458.0964.

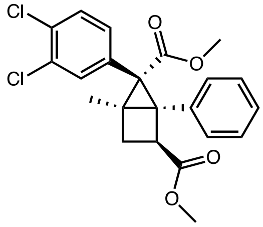

Dimethyl (1*R*,2*S*,4*R*,5*S*)-5-(3,4-dichlorophenyl)-4-methyl-1-phenylbicyclo[2.1.0]pentane-2,5-dicarboxylate (23a): General procedure D was followed using methyl 1-(3,4-dichlorophenyl)-2-methyl-3-phenylcycloprop-2-ene-1-carboxylate (63 mg, 0.19 mmol, 1 equiv), Ir(dF(CF$_3$)ppy)$_2$(tbbpy)PF$_6$ (2.1 mg, 0.19 mmol, 1 mol %), and methyl acrylate (86 µL, 0.95 mmol, 5.0 equiv) and MeCN (3.8 mL) added to a 4 mL vial. The reaction was cooled to -40 ºC and irradiated with blue LEDs. The reaction mixture was worked up as described in general procedure D and purified on silica gel (10% ethyl ether in pentane) to afford the product as an amorphous white solid. (144 mg, 62%).

^{1}H NMR (600 MHz, CDCl$_3$, 223 K) δ 7.57 (d, *J* = 1.9 Hz, 0.67H, major rotamer), 7.49 (d, *J* = 8.1 Hz, 0.67H, major rotamer), 7.47 – 7.39 (m, 4.45H, mixture of rotamers), 7.37 – 7.32 (m, 1.79H, mixture of rotamers), 7.15 (dd, *J* = 8.2, 2.0 Hz, 0.67H, major rotamer), 3.50 (s, 1.93H, major rotamer), 3.37 (s, 5H, mixture of rotamers) 3.36 (s, 1.04H, minor rotamer), 3.33 (ddd, *J* = 10.4, 4.5, 2.7 Hz, 1H, mixture of rotamers), 2.42 (td, *J* = 12.3, 10.4 Hz, 1H, mixture of rotamers), 2.01 (dd, *J* = 12.6, 4.4 Hz, 1H, mixture of rotamers), 1.76 (s, 1.09H, minor rotamer), 1.75 (s, 1.91H, major rotamer).

^{13}C NMR (101 MHz, CDCl$_3$, 292 K) δ 172.4, 169.8, 138.0, 134.1, 131.9, 129.1, 128.3, 127.3, 52.2, 51.4, 47.3, 46.5, 42.4, 35.7, 30.1, 14.1. (Four carbons could not be resolved at room temperature).

HRMS (APCI) m/z: [M+H]$^+$ calc'd for $C_{22}H_{21}O_4{}^{35}Cl_2$, 419.0811, found 419.0812.

Chiral SFC: CEL-1 3% EtOH/MeCN 0.2% formic acid 2.5 mL/min, λ=210 nm, Retention time = 2.54 (minor) and 2.74 (major). 94.3:5.7 e.r.

$[\alpha]_D^{22}$ = -51.8 (*c* = 0.34, CHCl$_3$)

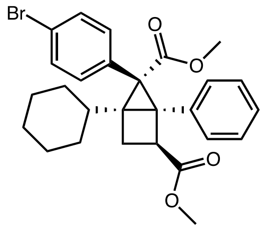

Dimethyl (1*R*,2*S*,4*S*,5*S*)-5-(4-bromophenyl)-4-cyclohexyl-1-phenylbicyclo[2.1.0]pentane-2,5-dicarboxylate (23b): General procedure D was followed using methyl 1-(4-bromophenyl)-2-cyclohexyl-3-phenylcycloprop-2-ene-1-carboxylate (298 mg, 0.72 mmol, 1 equiv), Ir(dF(CF_3)ppy)$_2$(tbbpy)PF_6 (8.1 mg, 0.0072 mmol, 1 mol %), and methyl acrylate (0.33 mL, 3.6 mmol, 5.0 equiv) and MeCN (14.5 mL) added to a 15 mL screw-cap test tube. The reaction was cooled to -40 ºC and irradiated with a blue LED. The reaction mixture was worked up as described in general procedure D and purified on silica gel (12% ethyl ether in pentane) to afford the product as an amorphous white solid. (225 mg, 62%).
^{1}H NMR (600 MHz, $CDCl_3$, 223 K) δ 7.50 – 7.43 (m, 4H), 7.38 (t, *J* = 7.5 Hz, 2H), 7.34 (dd, *J* = 8.2, 2.2 Hz, 1H), 7.31 – 7.27 (m, 1H), 7.16 (dd, *J* = 8.1, 2.2 Hz, 1H), 3.34 (s, 3H), 3.30 (s, 3H), 3.21 (dd, *J* = 10.3, 4.4 Hz, 1H), 2.58 (dd, *J* = 12.5, 10.4 Hz, 1H), 2.05 (q, *J* = 13.1 Hz, 2H), 1.80 (d, *J* = 12.9 Hz, 1H), 1.74 (dq, *J* = 10.2, 5.6 Hz, 2H), 1.66 (d, *J* = 12.2 Hz, 1H), 1.59 (d, *J* = 12.1 Hz, 1H), 1.50 – 1.41 (m, 1H), 1.36 – 1.27 (m, 2H), 1.26 – 1.10 (m, 3H).
^{13}C NMR (101 MHz, $CDCl_3$, 298 K) δ 172.6, 170.3, 138.6, 132.7, 129.1, 128.2, 127.0, 121.8, 52.2, 51.3, 48.9, 47.7, 45.6, 42.5, 34.1, 31.7, 26.6, 26.4, 26.4, 23.5. (Four carbons could not be resolved at room temperature).
HRMS (APCI) m/z: [M+H]$^+$ calc'd for $C_{27}H_{30}O_4{}^{79}Br$, 497.1322, found 497.1324.
Chiral SFC: OZ-3 5% EtOH/MeCN 0.2% formic acid 2.5 mL/min, λ=210 nm, Retention time = 2.91 (minor) and 3.37 (major). 92.5:7.6 e.r.
$[\alpha]_D^{22}$ = -102.2 (*c* = 0.40, $CHCl_3$)

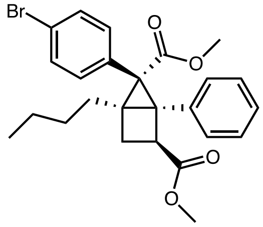

Dimethyl 5-(4-bromophenyl)-4-butyl-1-phenylbicyclo[2.1.0]pentane-2,5-dicarboxylate (23c): General procedure D was followed using methyl 1-(4-bromophenyl)-2-butyl-3-phenylcycloprop-2-ene-1-carboxylate (191 mg, 0.50 mmol, 1 equiv), Ir(dF(CF_3)ppy)$_2$(tbbpy)PF_6 (5.6 mg, 0.0056 mmol, 1 mol %), and methyl acrylate (0.22 mL, 1.0 mmol, 5.0 equiv) and MeCN (10 mL) added to a 15 mL screw-cap test tube. The reaction was cooled to -40 ºC and irradiated with a blue LED. The reaction mixture was worked up as described in general procedure D and purified on silica gel (15% ethyl ether in pentane) to afford the product as an amorphous white solid. (160 mg, 69%)
^{1}H NMR (600 MHz, $CDCl_3$, 223 K) δ 7.51 (dd, *J* = 8.2, 2.1 Hz, 1H), 7.48 – 7.44 (m, 3H), 7.42 – 7.35 (m, 3H), 7.31 (t, *J* = 7.4 Hz, 1H), 7.13 (dd, *J* = 8.2, 2.3 Hz, 1H), 3.33 (s, 6H), 3.26 (dd, *J* = 10.3, 4.3 Hz, 1H), 2.34 (dd, *J* = 12.4, 10.3 Hz, 1H), 2.12 (td, *J* = 12.9, 4.8 Hz, 1H), 2.00 (dd, *J* = 12.4, 4.4 Hz, 1H), 1.98 – 1.91 (m, 1H), 1.64 – 1.52 (m, 1H), 1.38 – 1.31 (m, 2H), 1.24 (tq, *J* = 12.5, 5.8 Hz, 1H), 0.90 (t, *J* = 7.3 Hz, 3H).
^{13}C NMR (101 MHz, $CDCl_3$, 298 K) δ 172.5, 170.2, 138.5, 132.7, 129.2, 128.3, 127.2, 121.8, 52.1, 51.2, 47.8, 46.9, 42.5, 40.3, 30.0, 29.0, 27.8, 23.1, 14.2. (Four carbons could not be resolved at room temperature).
HRMS (APCI) m/z: [M+H]$^+$ calc'd for $C_{25}H_{28}O_4{}^{79}Br$, 471.1166, found 471.1163.
Chiral SFC: *S,S*-WHELK 5% EtOH/MeCN 0.2% formic acid 2.5 mL/min, λ=210 nm, Retention time = 3.19 (major) and 4.49 (minor). 95.6:4.4 e.r.
$[\alpha]_D^{22}$ = -83.3 (*c* = 0.55, $CHCl_3$)

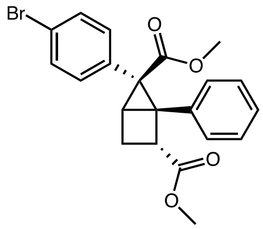

Dimethyl (1*S*,2*R*,5*S*)-5-(4-bromophenyl)-1-phenylbicyclo[2.1.0]pentane-2,5-dicarboxylate (23d): General procedure D was followed using methyl (*S*)-1-(4-bromophenyl)-2-phenylcycloprop-2-ene-1-carboxylate (165 mg, 0.50 mmol, 1 equiv), Ir(dF(CF_3)ppy)$_2$(tbbpy)PF_6 (5.6 mg, 0.005 mmol, 1 mol %), and methyl acrylate (0.23 mL, 2.5 mmol, 5.0 equiv) and MeCN (10 mL) added to a 15 mL screw-cap test tube. The reaction was cooled to -40 ºC and irradiated with a blue LED. The reaction mixture was worked up as described in general procedure D and purified on silica gel (17% ethyl ether in pentane) to afford the product as an amorphous white solid. (51 mg, 25%).
^{1}H NMR (800 MHz, $CDCl_3$, 253 K) δ 7.57 – 7.51 (m, 5H), 7.37 (t, *J* = 7.7 Hz, 2H), 7.30 (t, *J* = 7.3 Hz, 1H), 7.18 (d, *J* = 7.3 Hz, 1H), 3.40 (dd, *J* = 10.3, 4.3 Hz, 1H), 3.35 (s, 3H), 3.24 (s, 3H), 3.05 (d, *J* = 4.8 Hz, 1H), 2.59 (ddd, *J* = 12.5, 10.3, 4.9 Hz, 1H), 1.80 (dd, *J* = 12.5, 4.3 Hz, 1H).
^{13}C NMR (201 MHz, $CDCl_3$, 300 K) δ 172.3, 170.3, 137.4, 131.2, 130.9, 129.4, 128.3, 127.6, 122.2, 52.3, 51.3, 45.5, 45.3, 43.4, 26.9, 21.9. (Two carbons could not be resolved at room temperature).
HRMS (APCI) m/z: $[M–H]^-$: calc'd for $C_{21}H_{18}O_4{}^{79}Br$, 413.0394, found 413.0390.
Chiral SFC: OJ-3 4% EtOH/MeCN 0.2% formic acid 2.0 mL/min, λ=210 nm, Retention time = 1.74 (major) and 3.11 (minor). 95.0:5.0 e.r.
$[\alpha]_D^{22}$ = -6.1 (*c* = 0.21, $CHCl_3$)

V. Diastereomer NMR

Va. Evidence for Diastereomer Stereochemistry Assignment

18a *endo anti* ^{1}H NMR major diastereomer ambient temperature probe

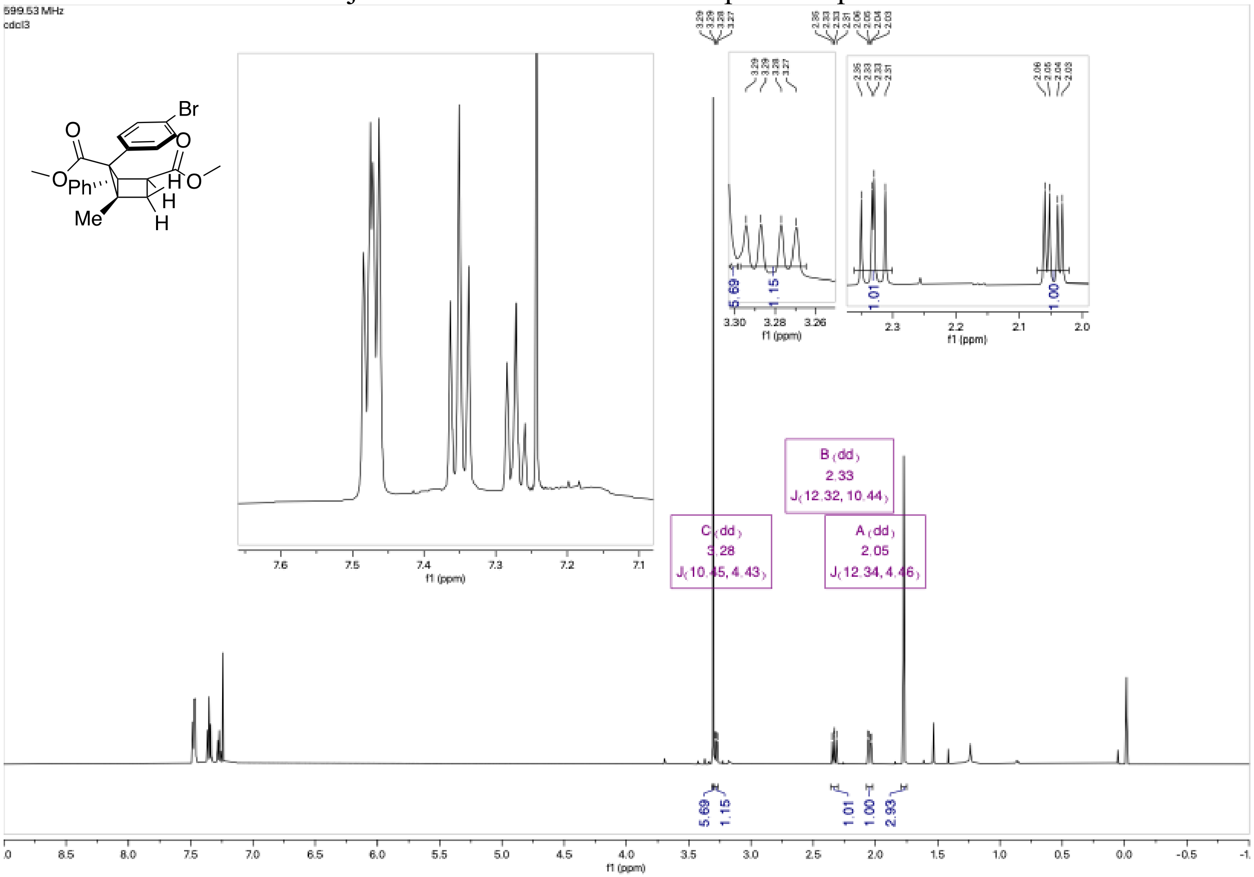

18a *endo anti* ^{1}H NMR major diastereomer variable temperature probe

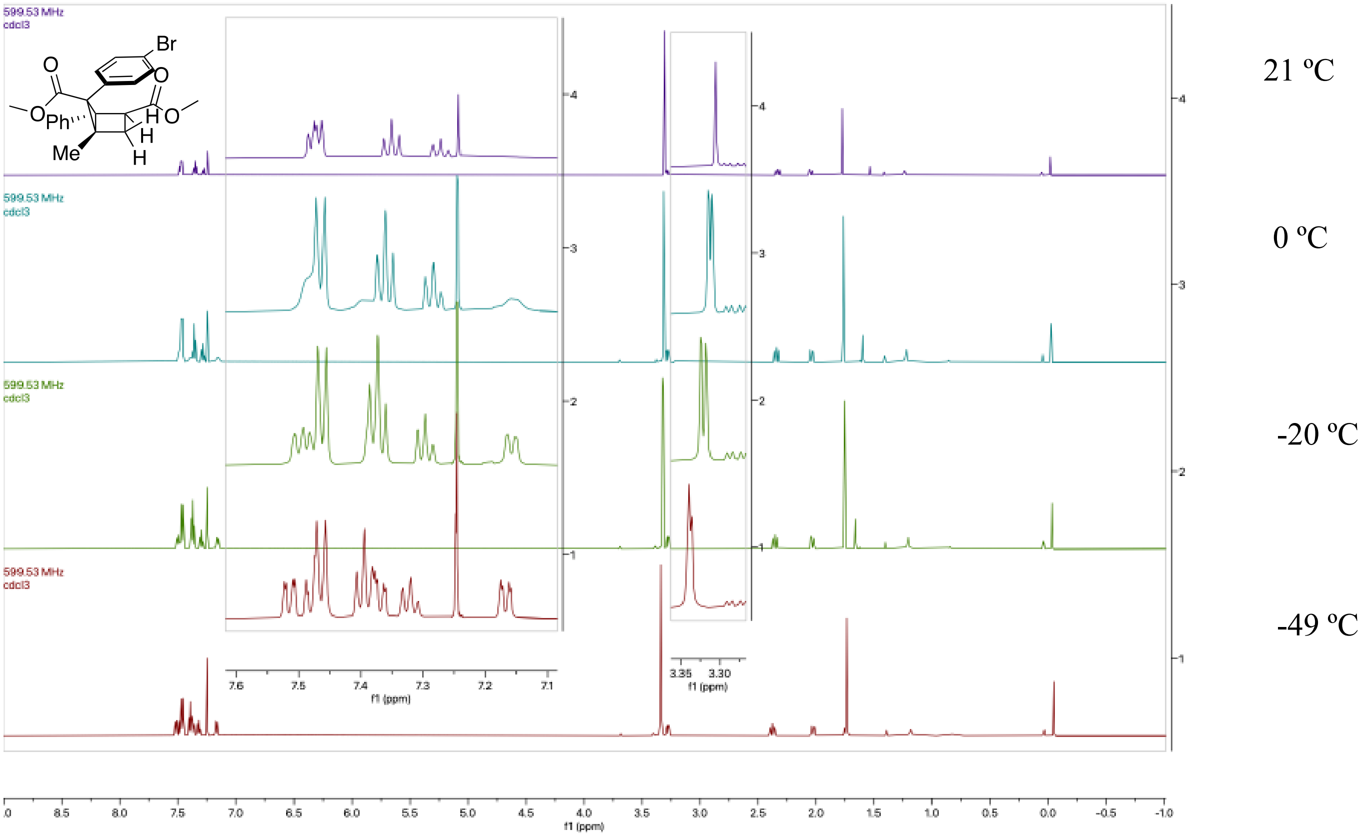

18a *endo anti* ^{1}H NMR major diastereomer -49 ºC temperature probe

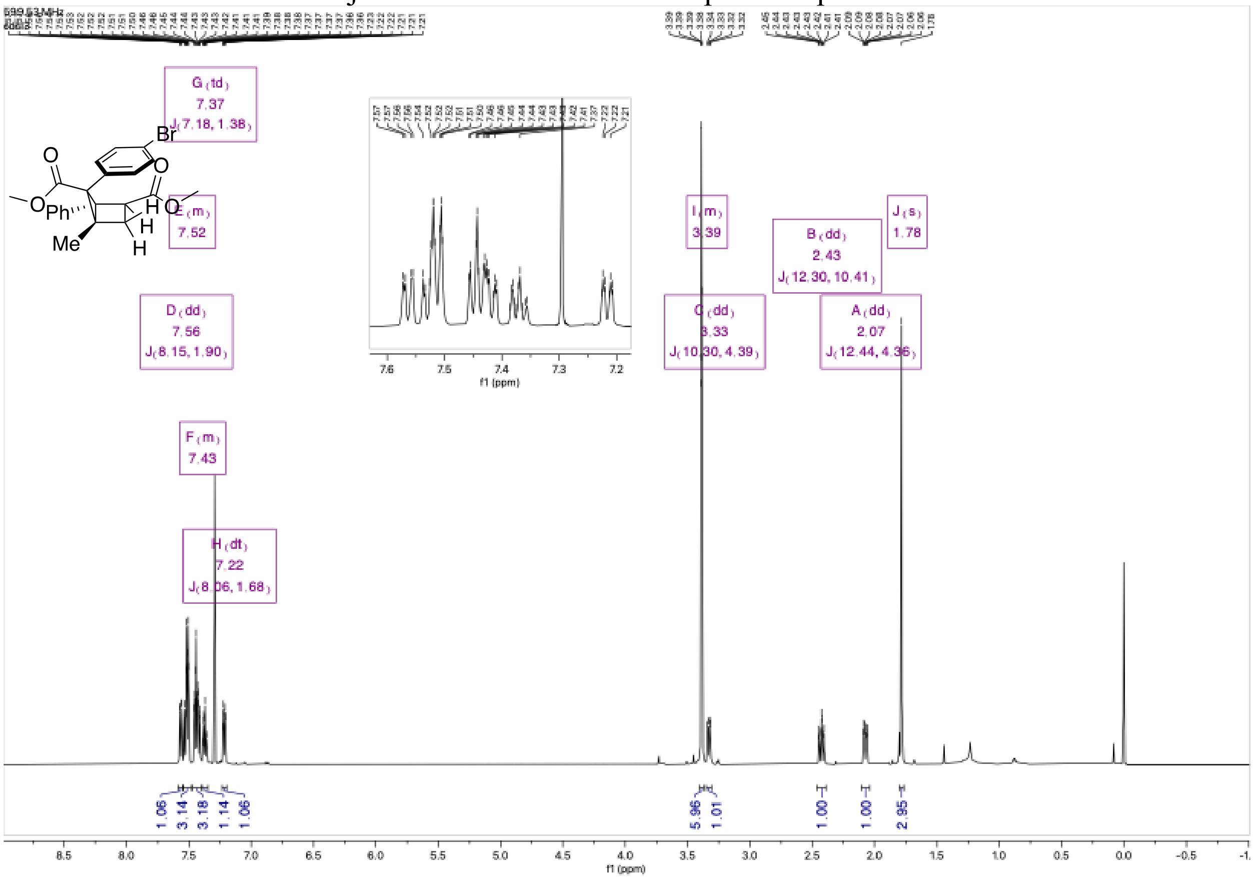

18a *Endo anti* ^{13}C NMR major diastereomer ambient temperature probe

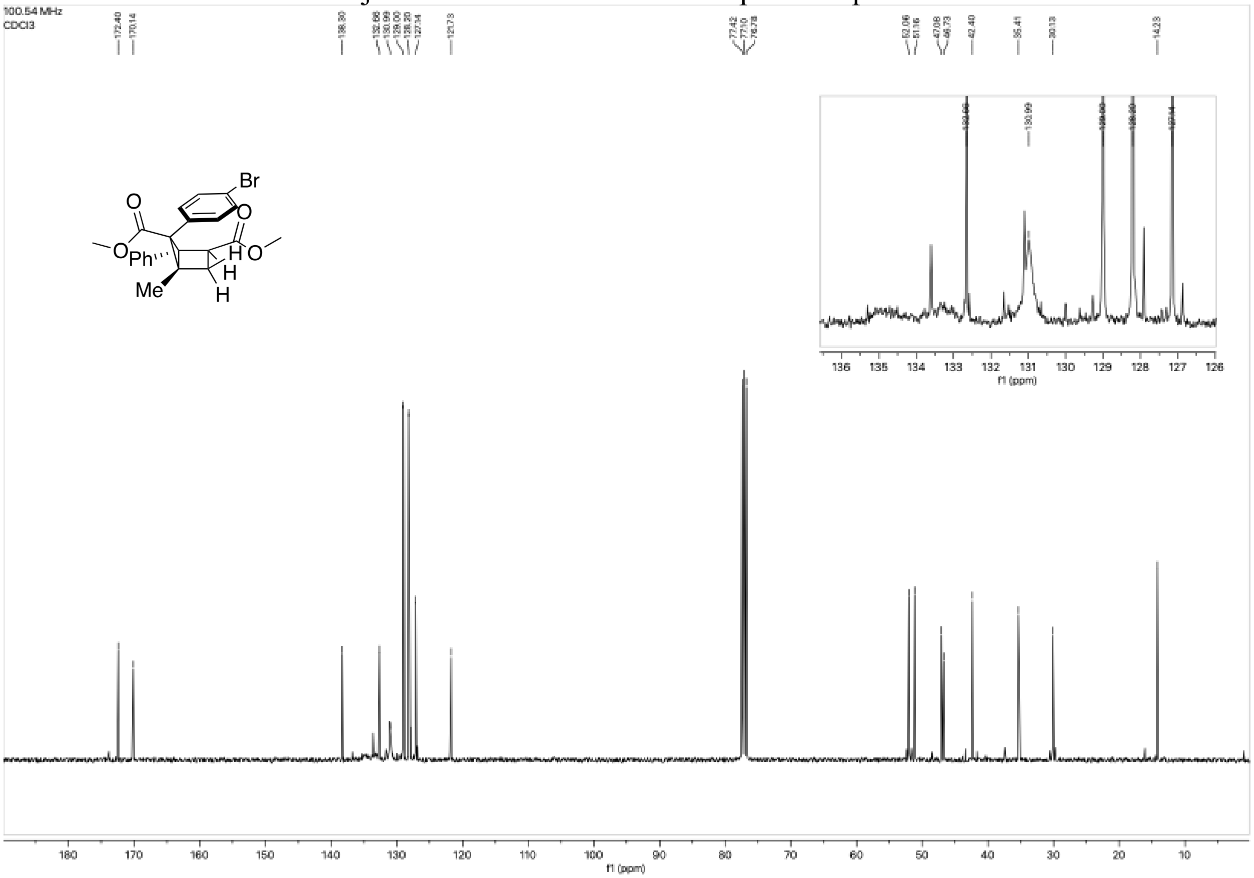

18a *endo anti* major diastereomer COSY

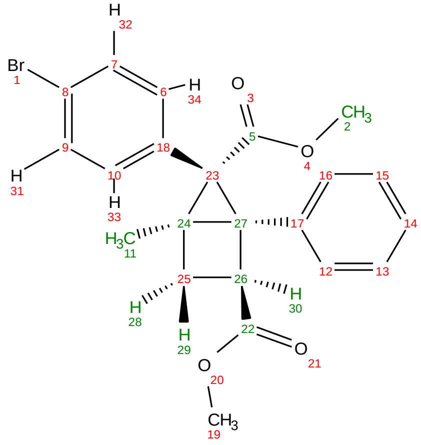

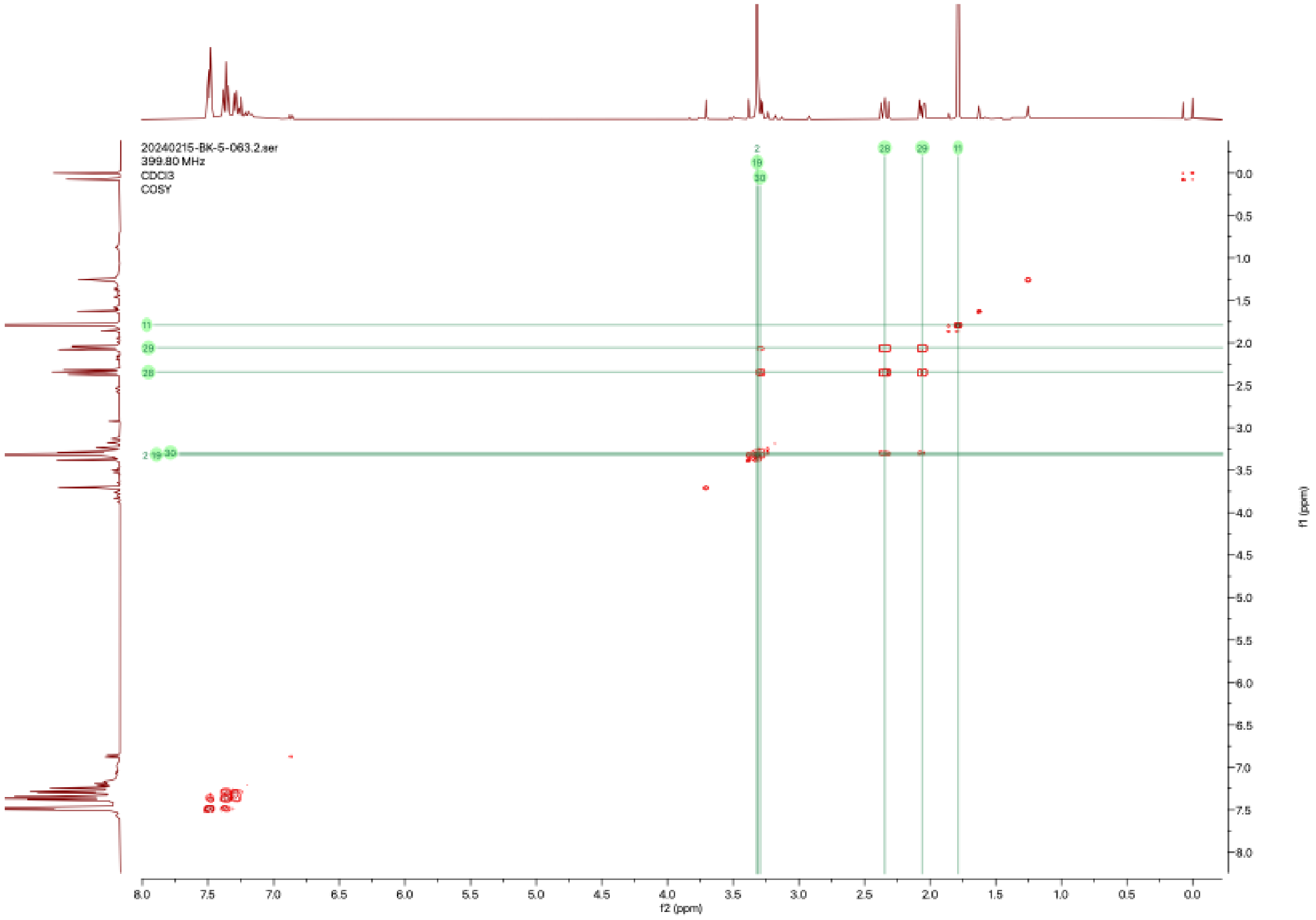

18a *endo anti* major diastereomer HSQC

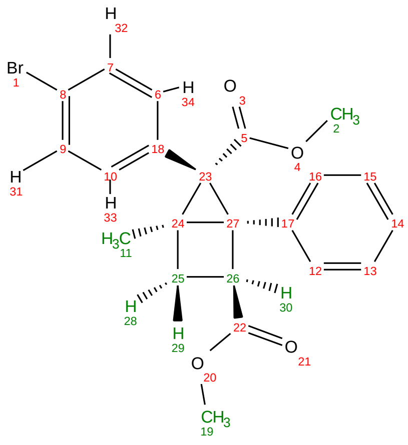

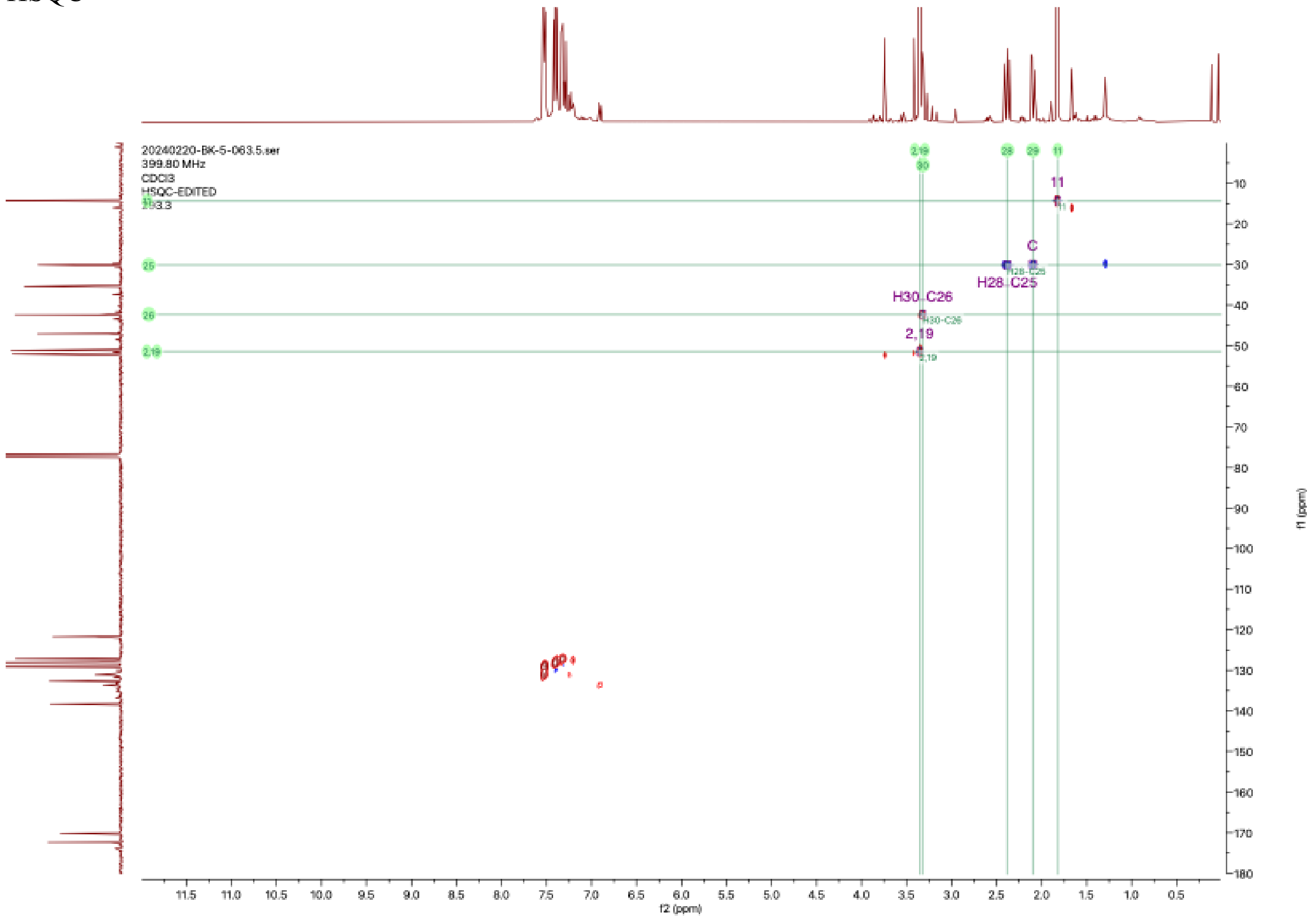

18a *endo anti* major diastereomer HSQC

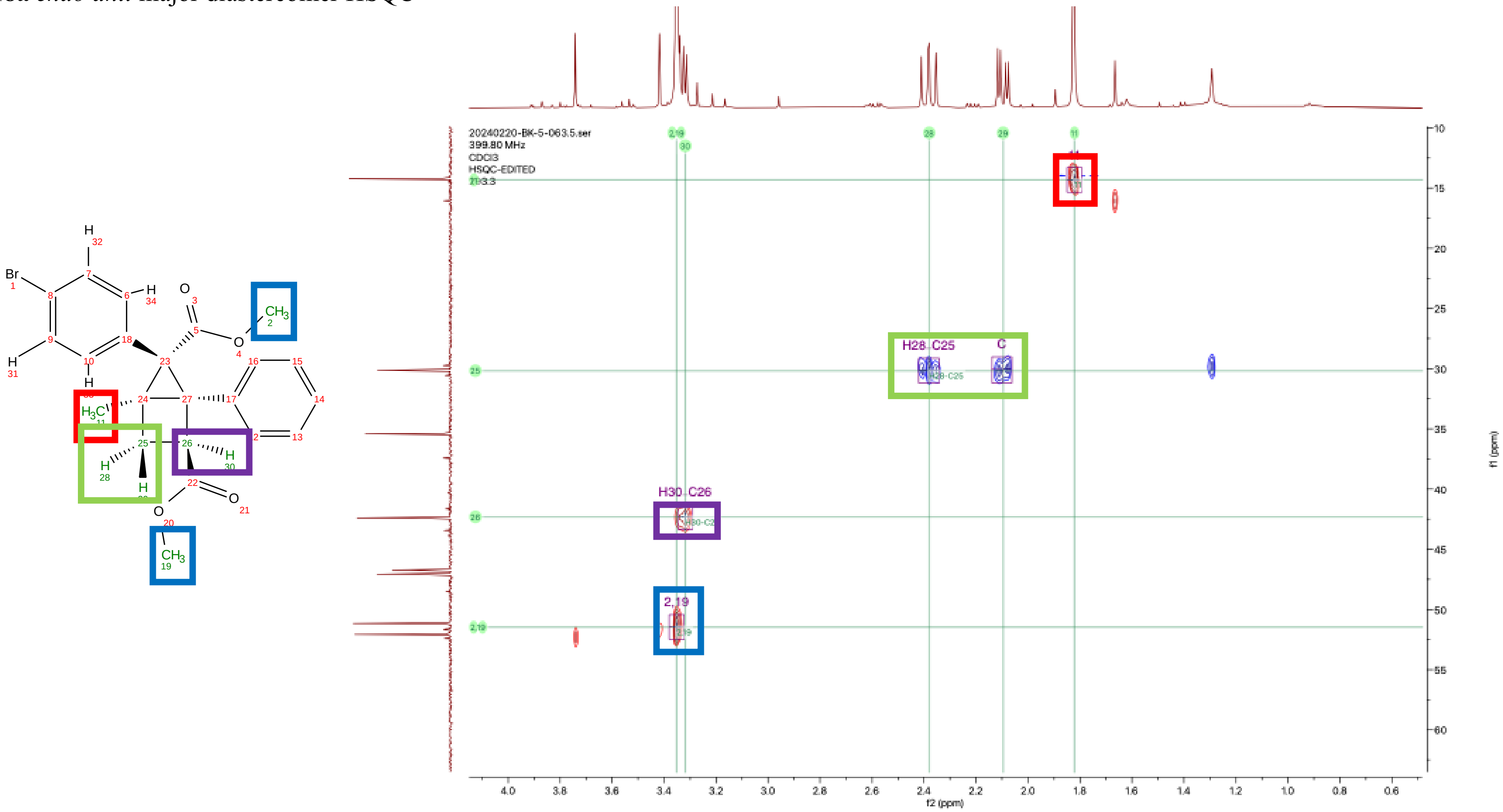

18a *endo anti* major diastereomer HMBC

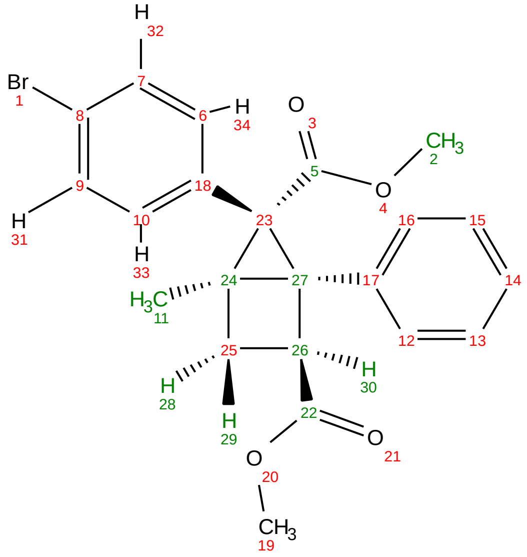

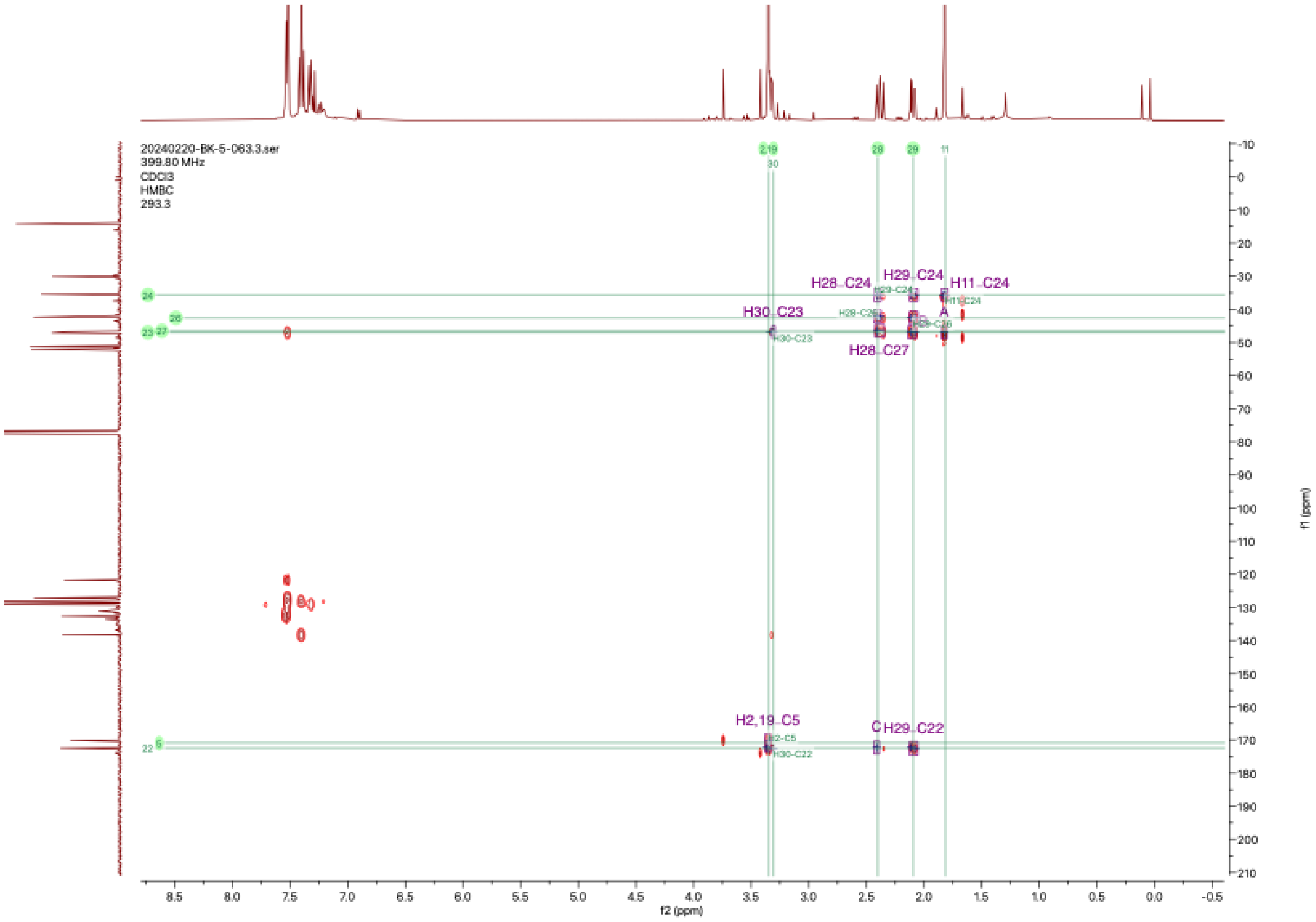

18a *endo anti* major diastereomer HMBC

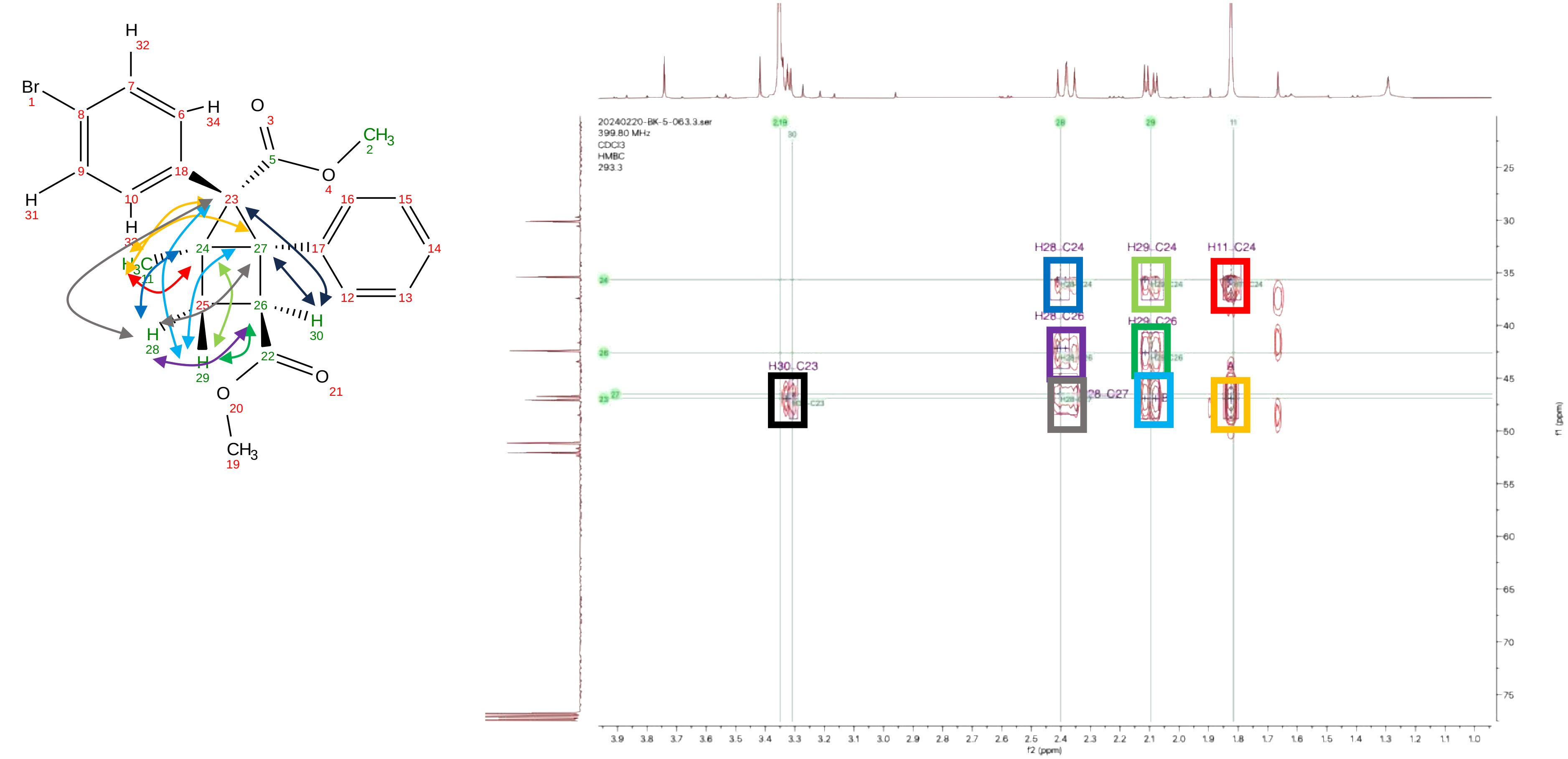

18a *endo anti* major diastereomer HMBC

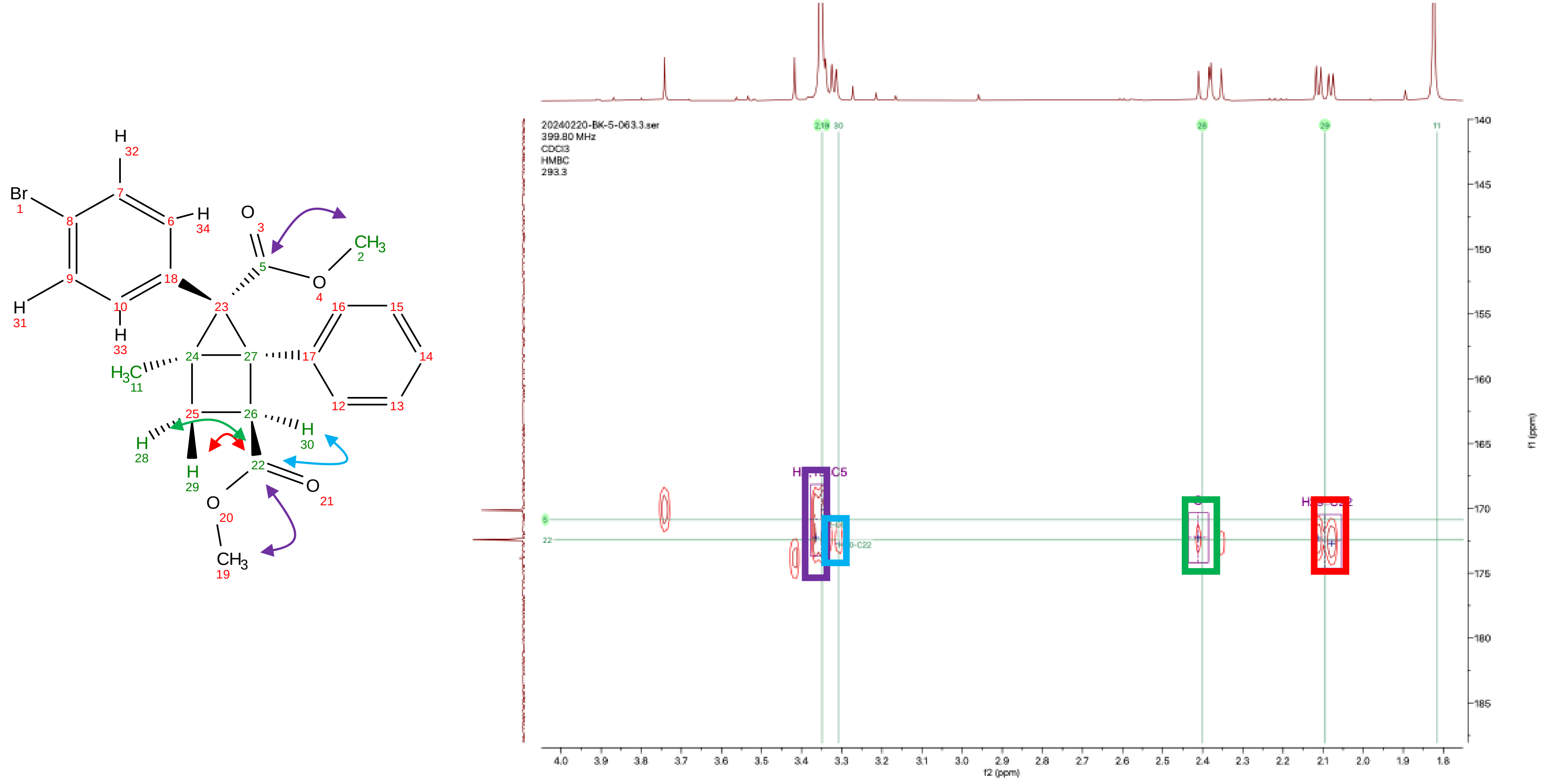

18a *endo anti* major diastereomer NOESY at -30 ºC

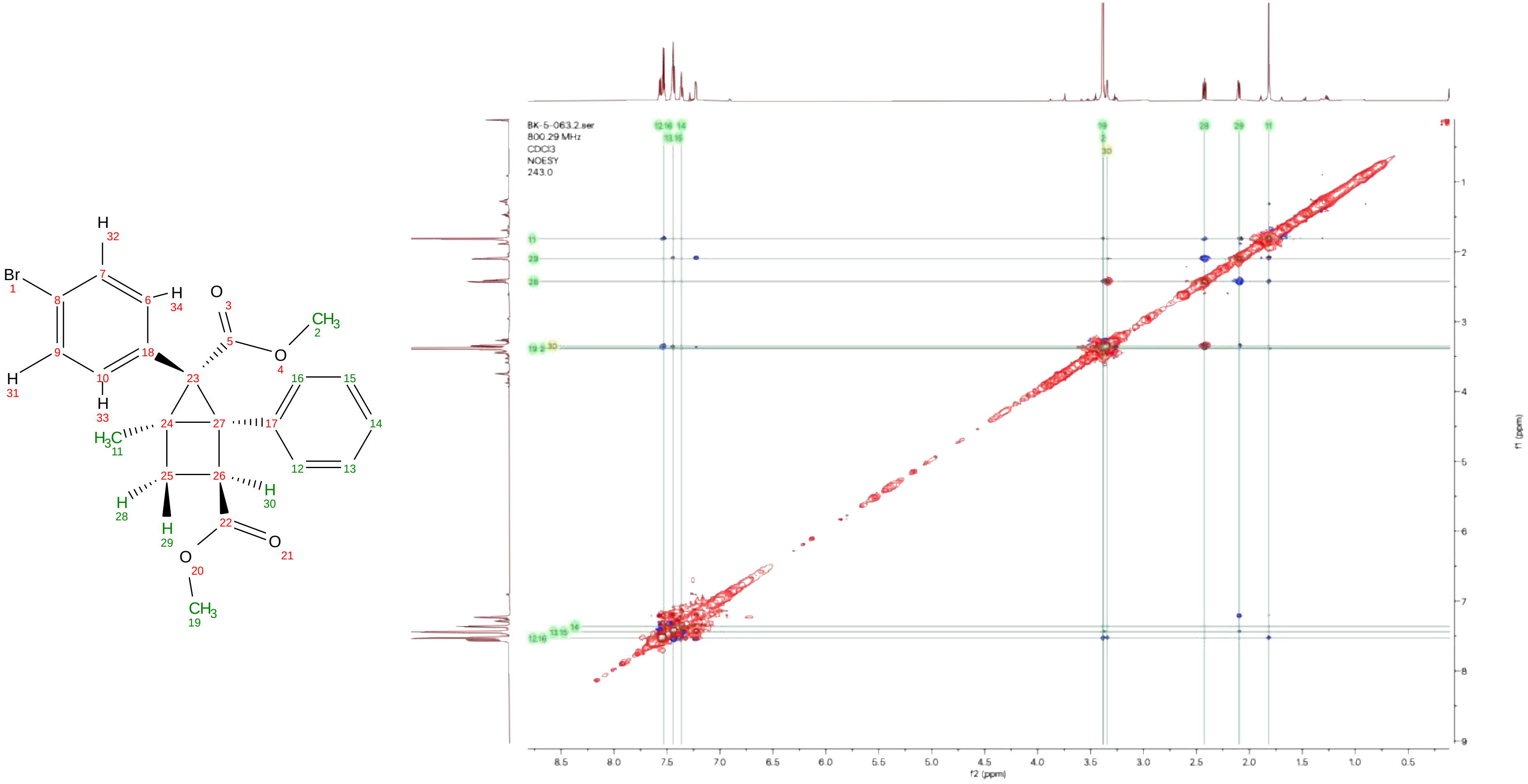

18a *endo anti* major diastereomer NOESY at -30 ºC

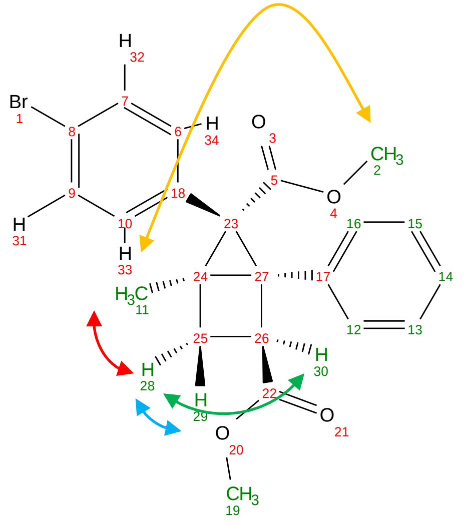

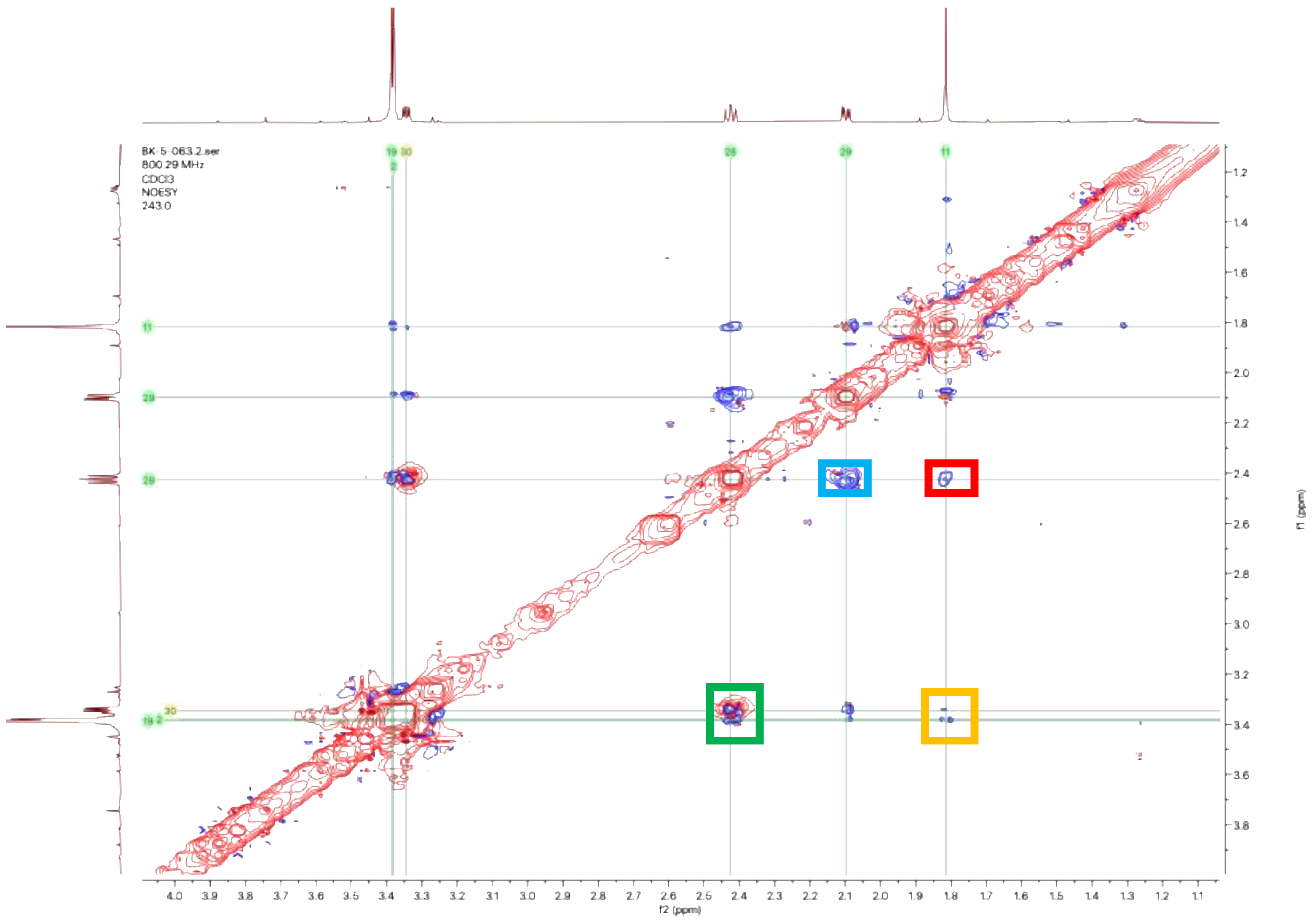

18a *endo anti* major diastereomer NOESY at -30 ºC

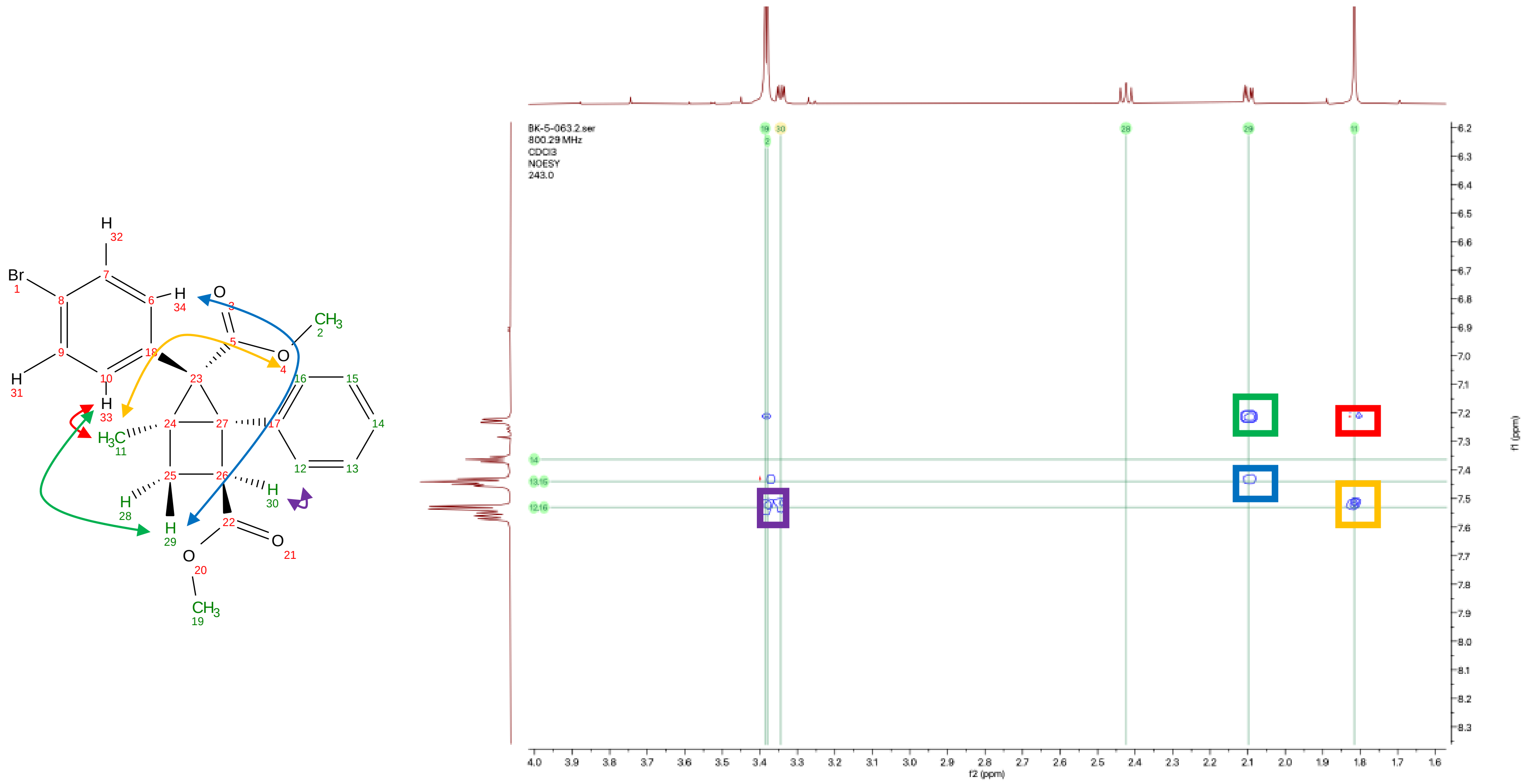

18a *endo anti* major diastereomer NOESY at -30 ºC

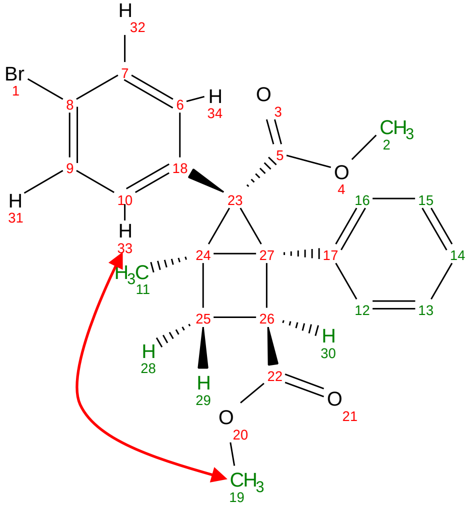
Br
CH3
H3C
CH3

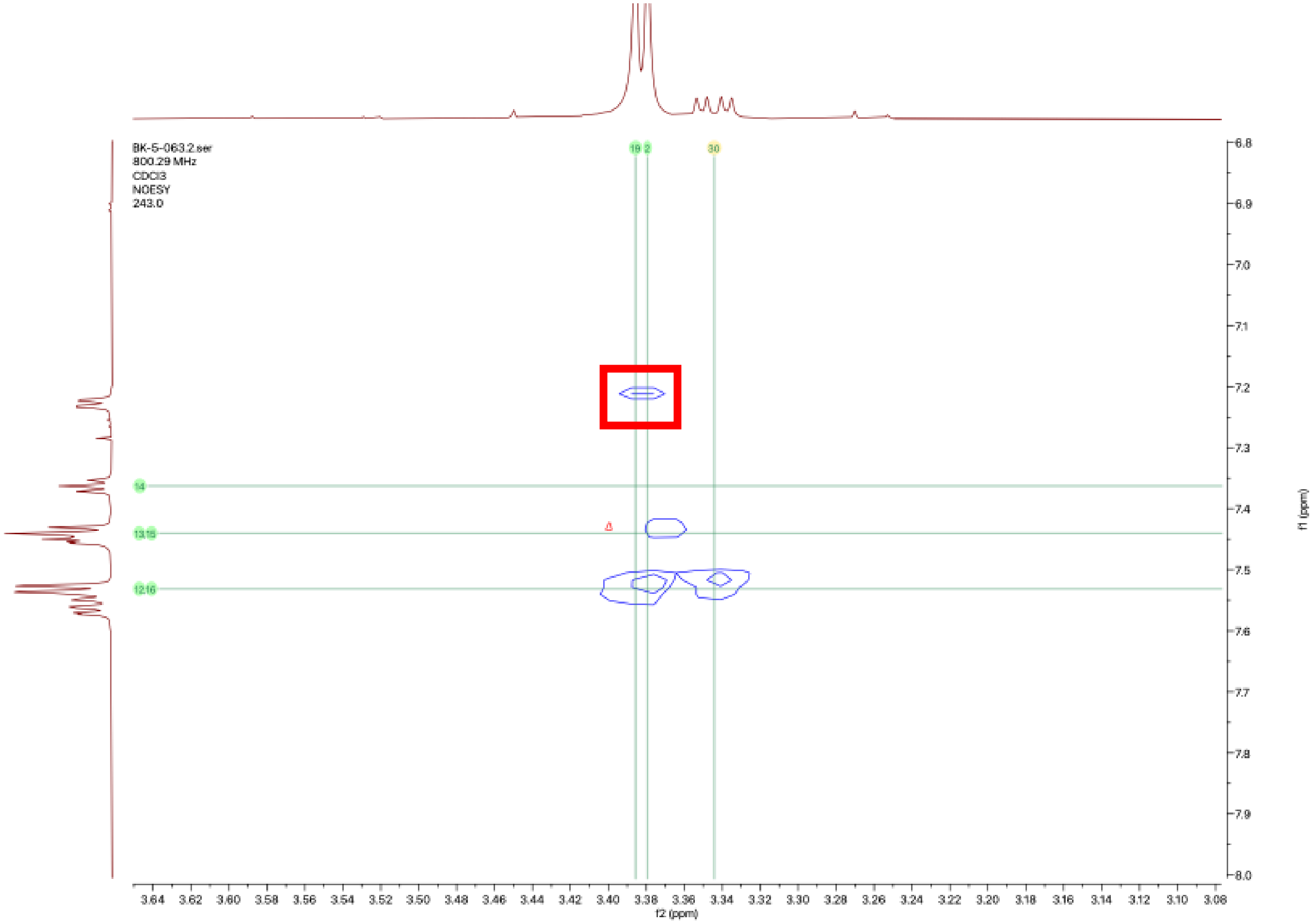
BK-5-063.2.ser
800.29 MHz
CDCl3
NOESY
243.0
f2 (ppm)
f1 (ppm)

18b *endo syn* ^{1}H NMR minor diastereomer ambient temperature probe

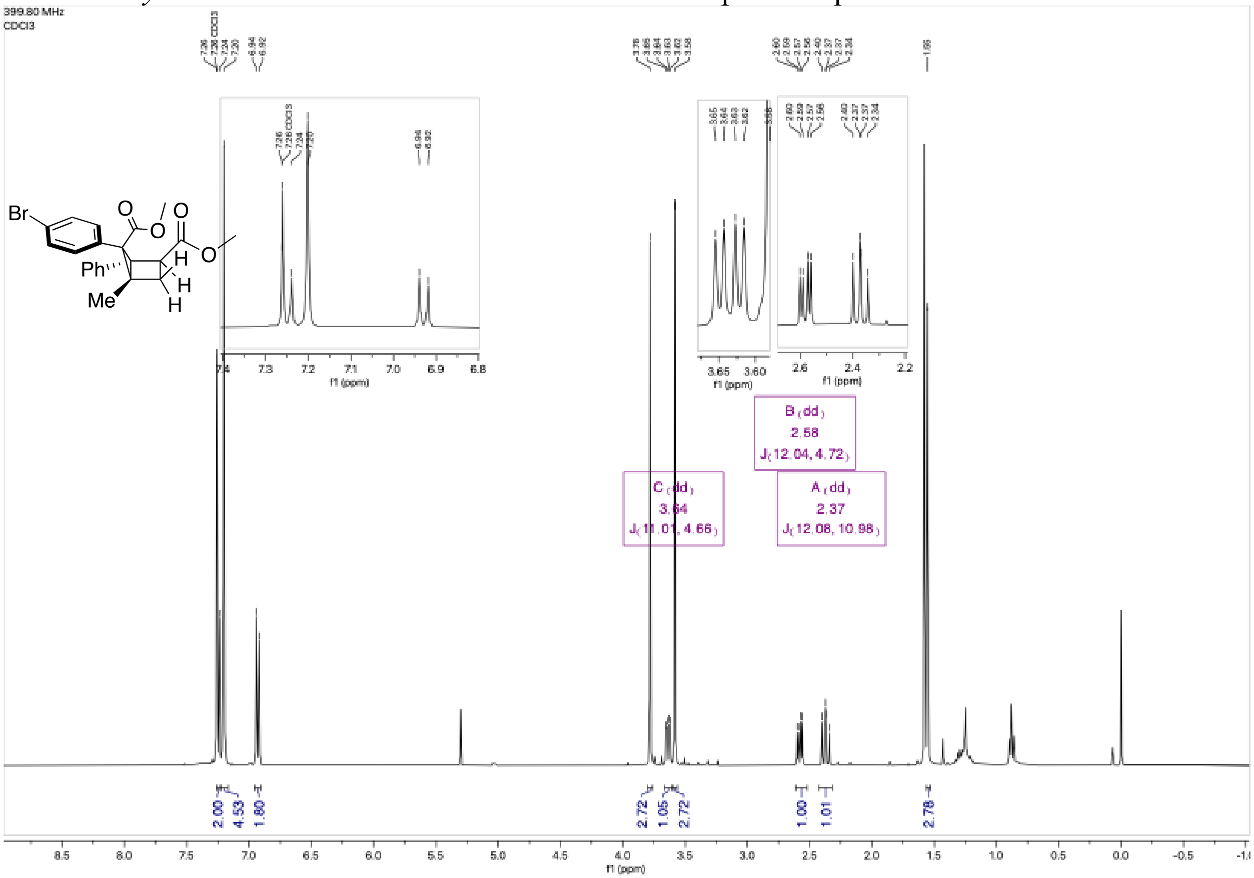

18b *endo syn* ^{13}C NMR minor diastereomer ambient temperature probe

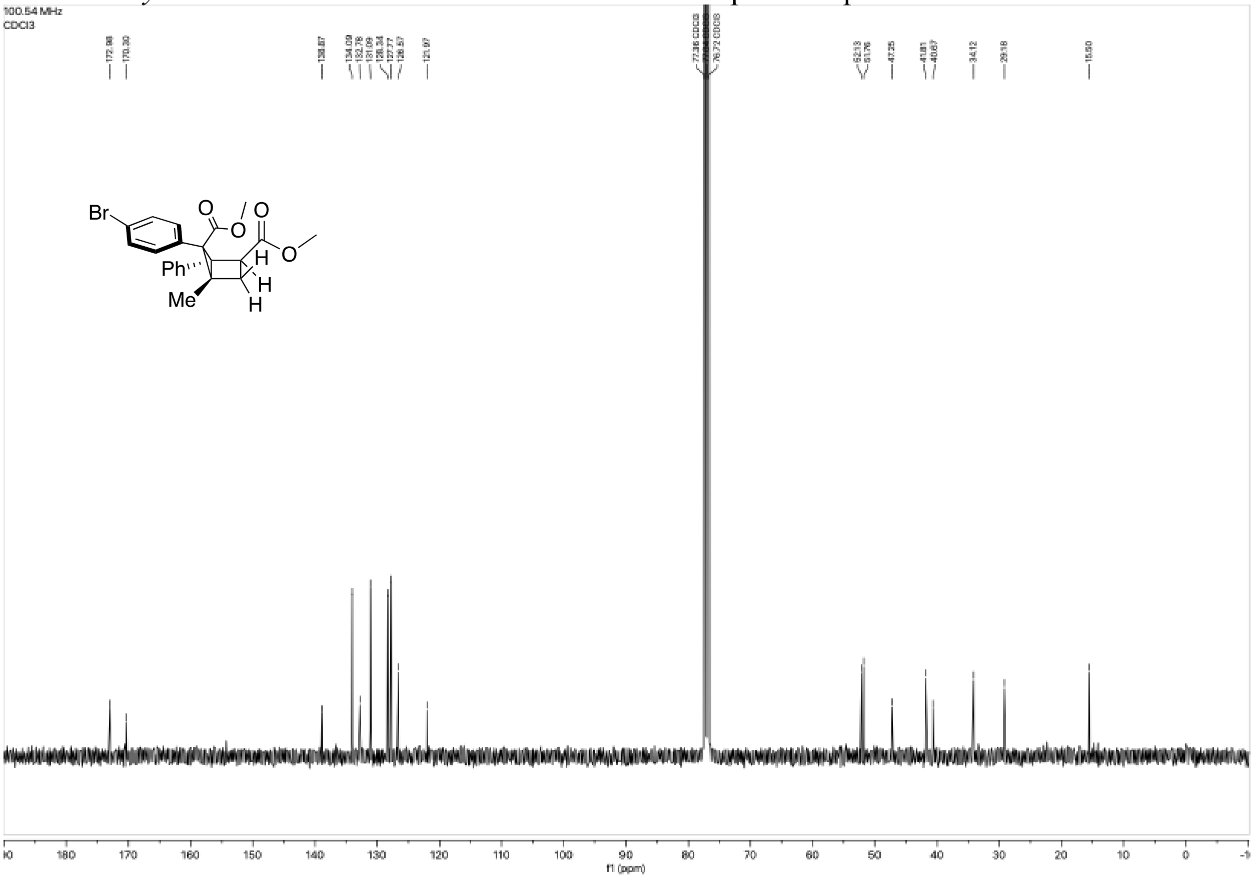

18b *endo syn* minor diastereomer HSQC

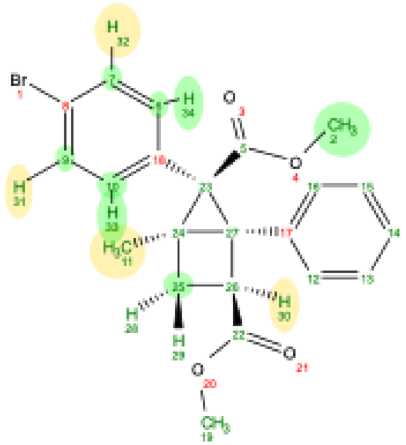

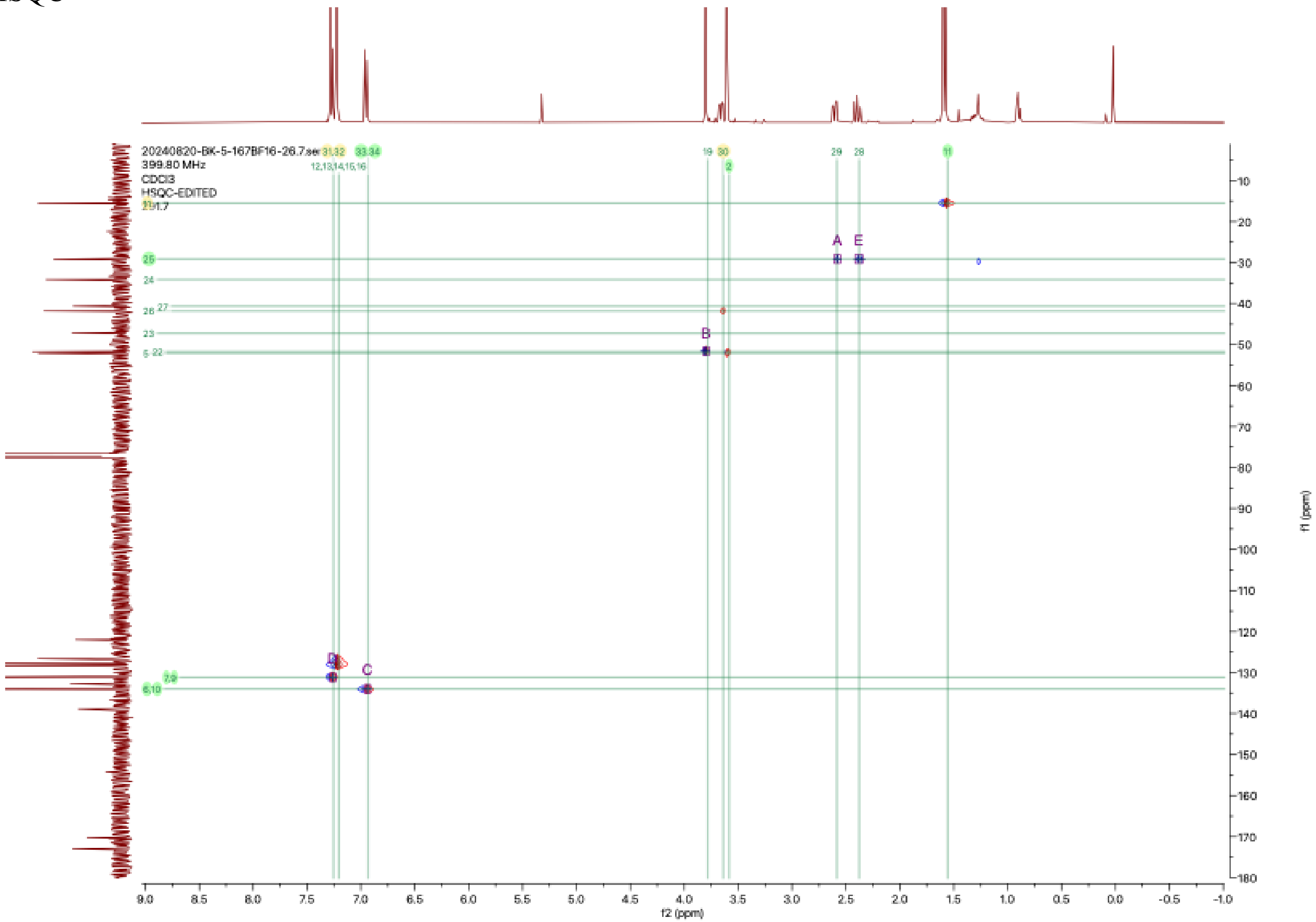

18b *endo syn* minor diastereomer HSQC

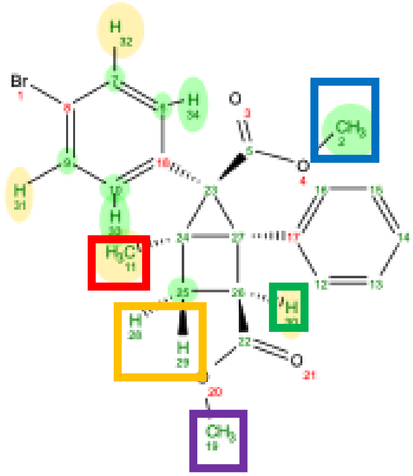

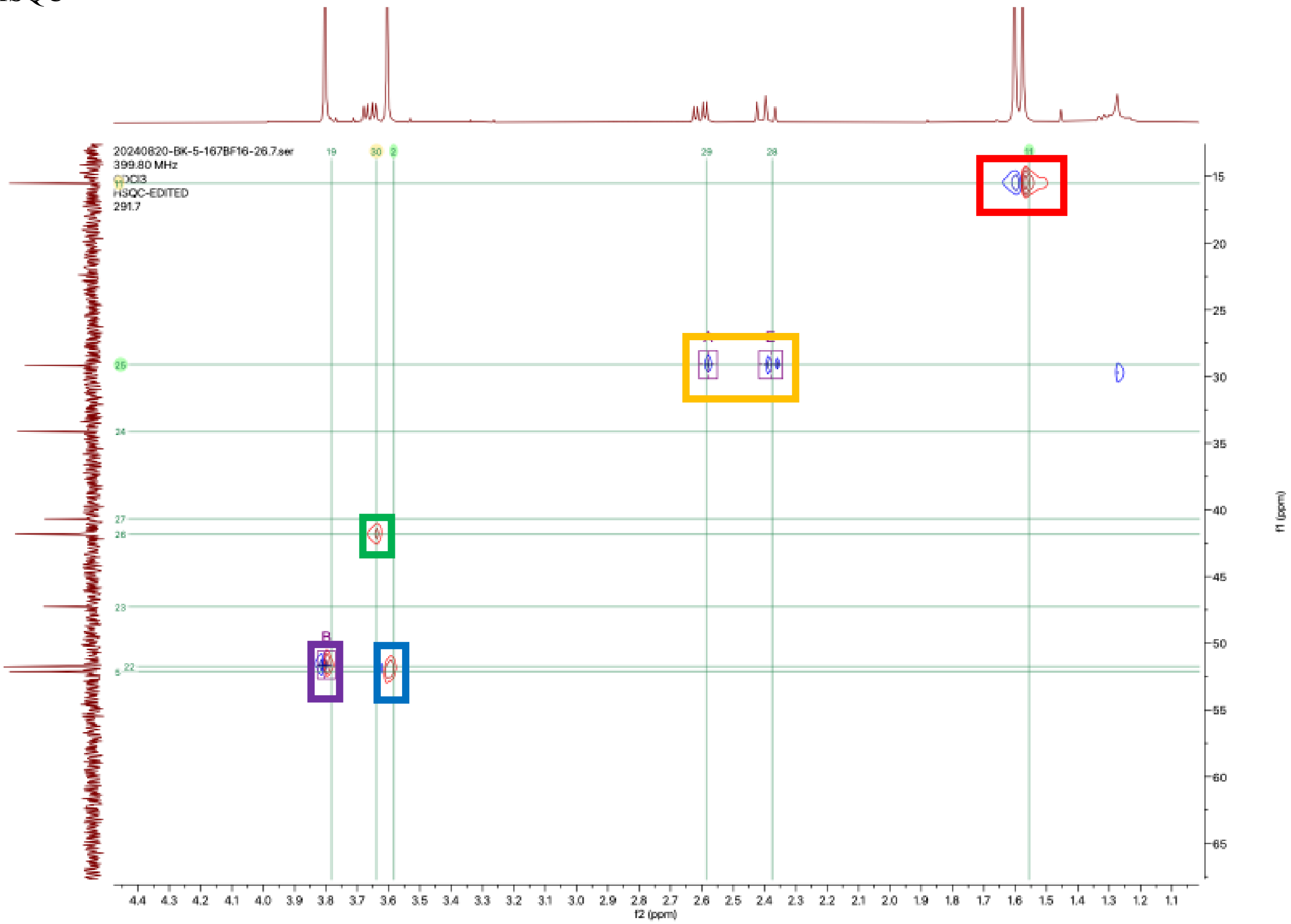

18b *endo syn* minor diastereomer HSQC

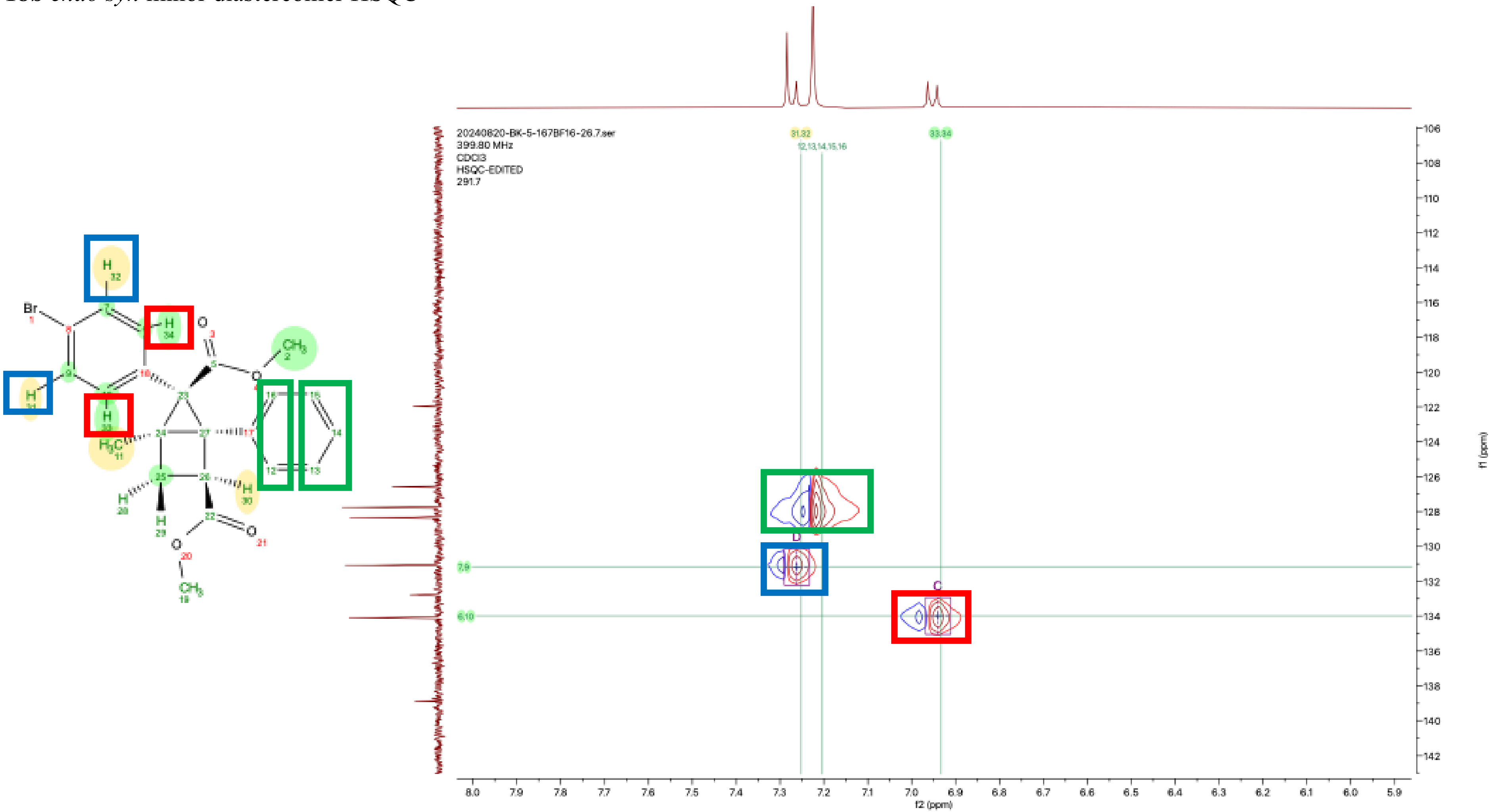

18b *endo syn* minor diastereomer HMBC

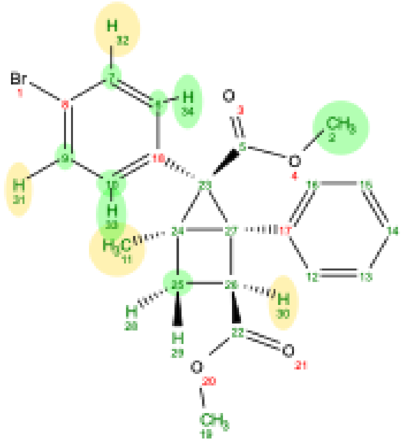

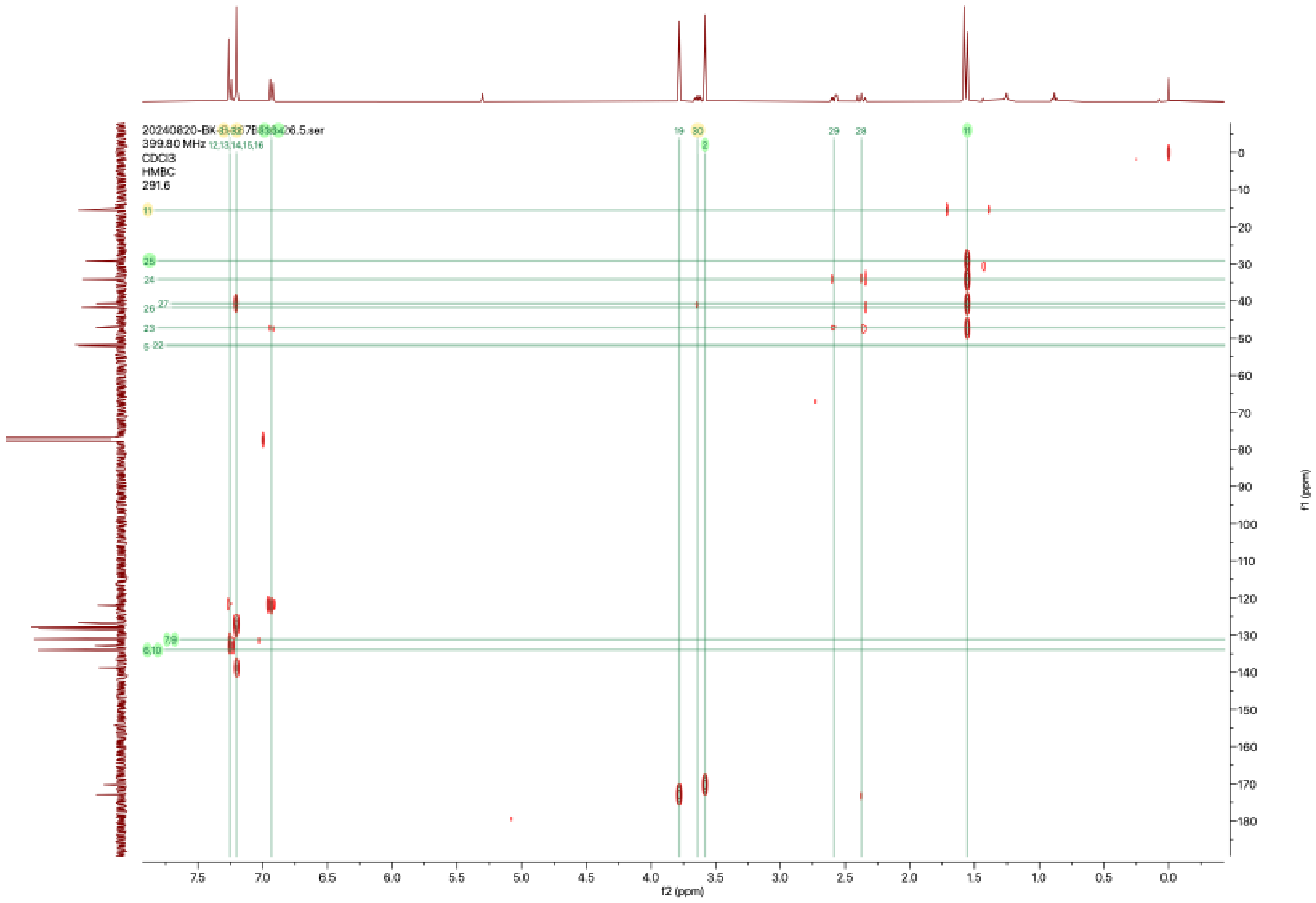

18b *endo syn* minor diastereomer HMBC

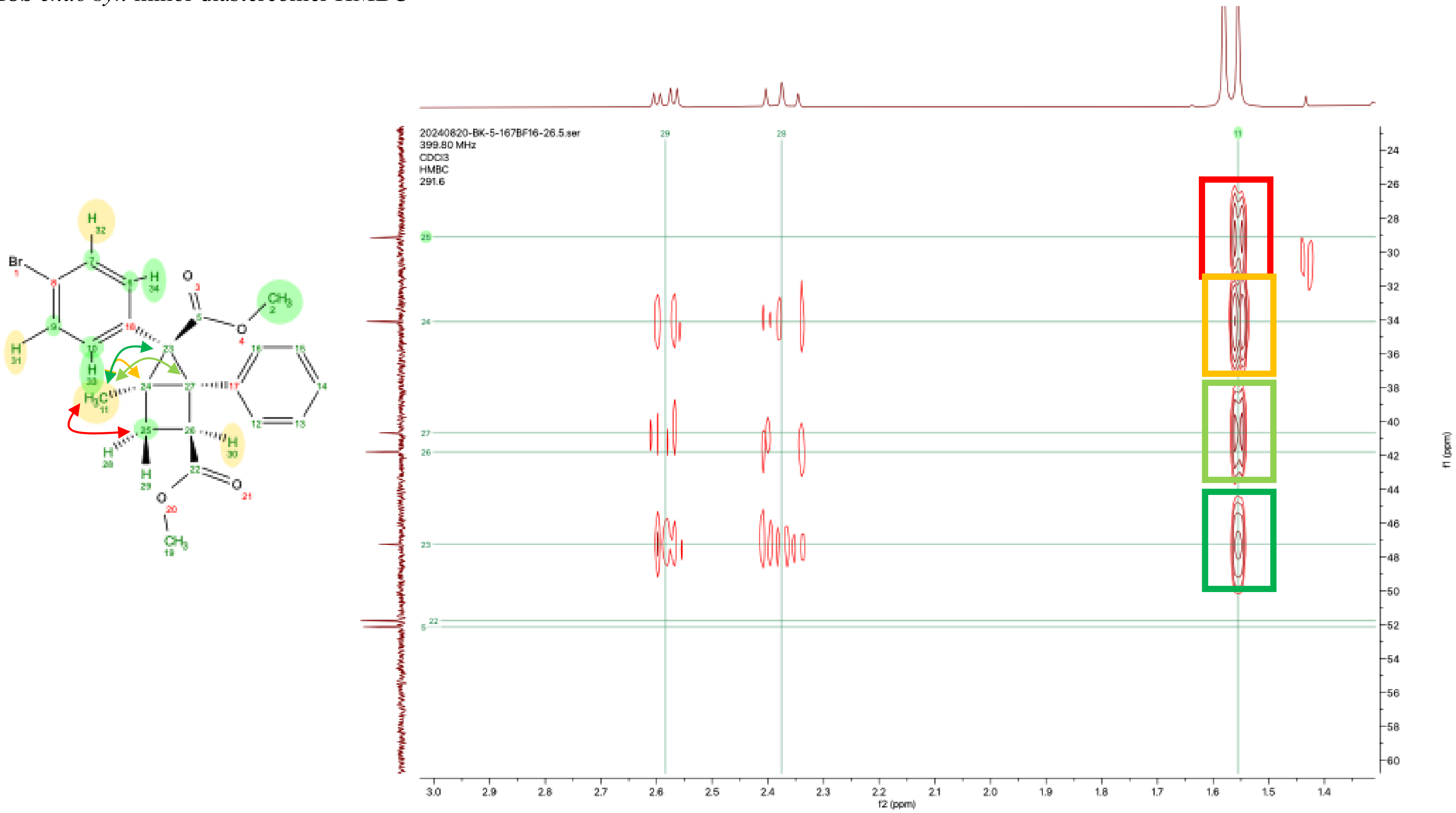

18b *endo syn* minor diastereomer HMBC

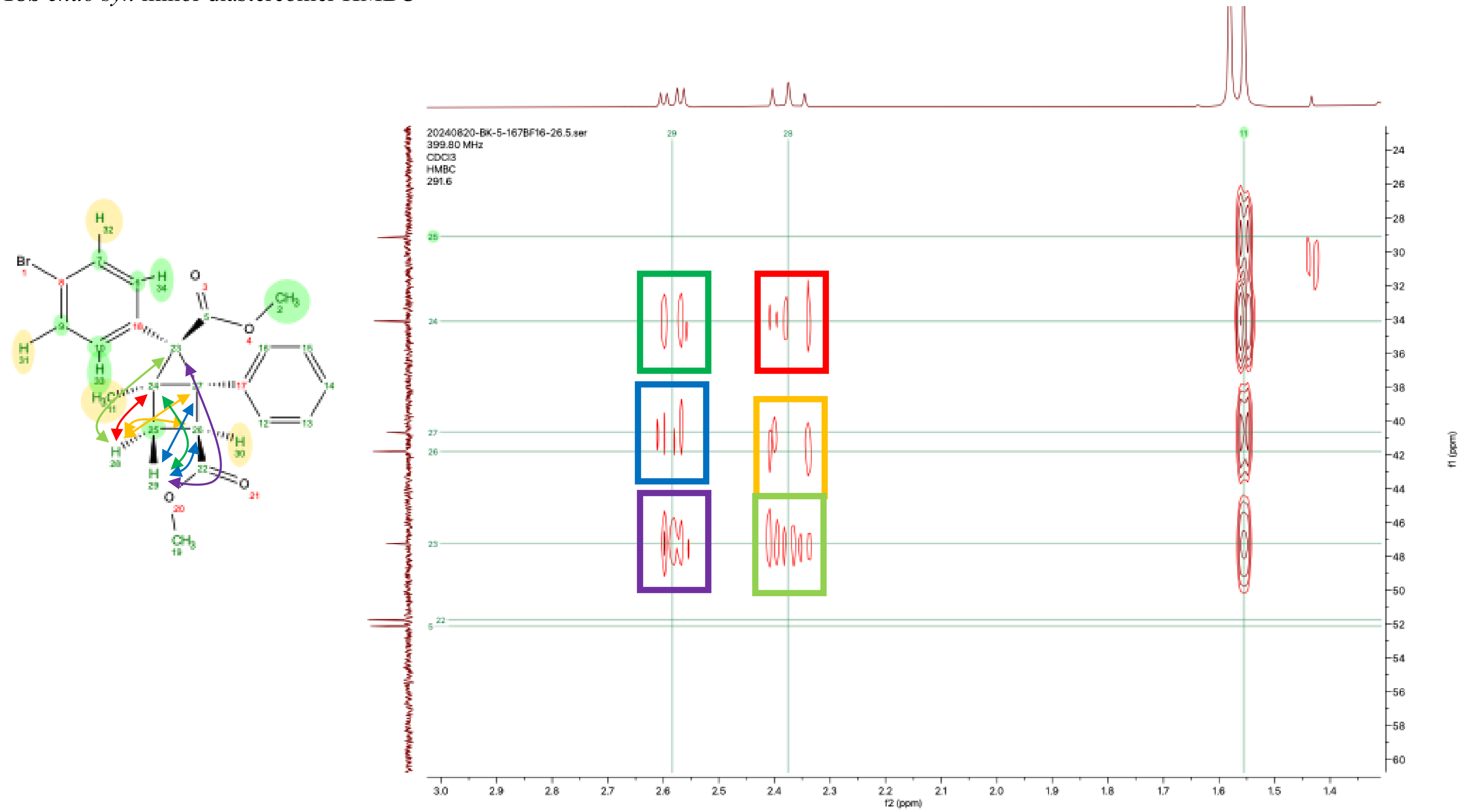

18b *endo syn* minor diastereomer HMBC

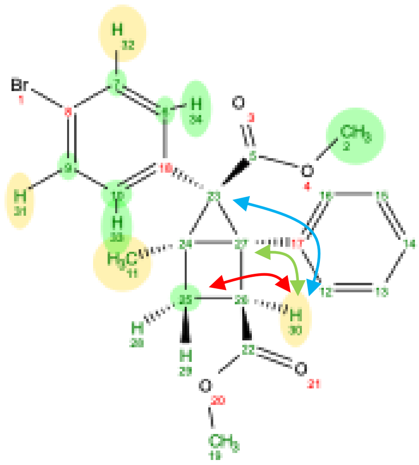

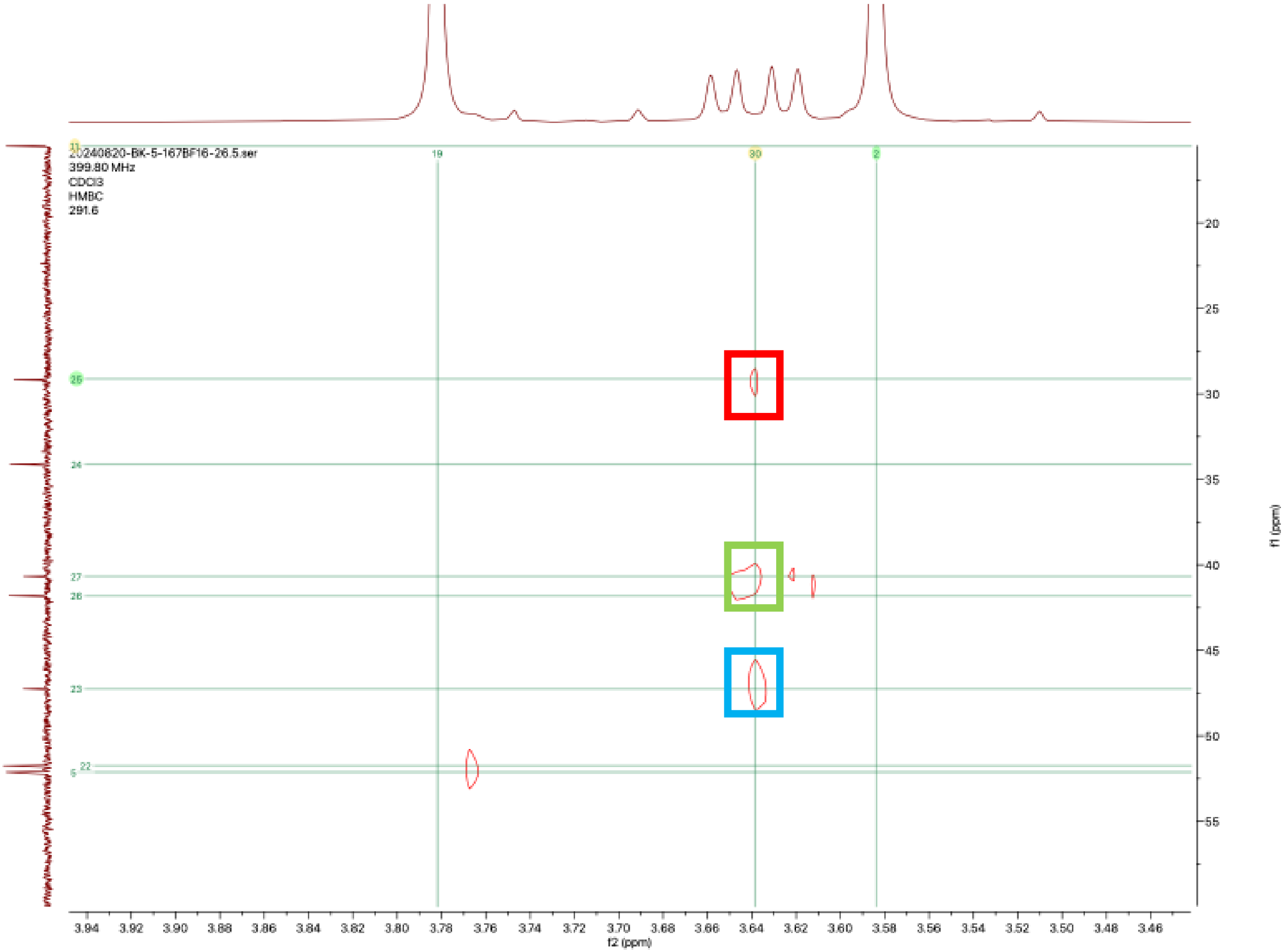

18b *endo syn* minor diastereomer HMBC

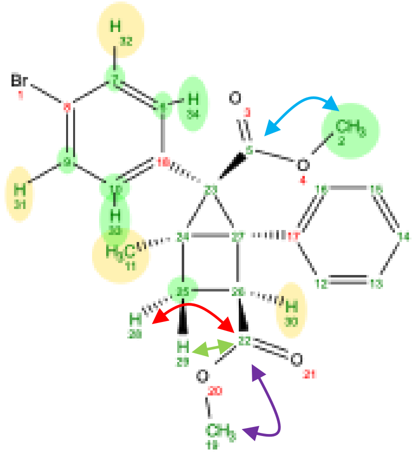

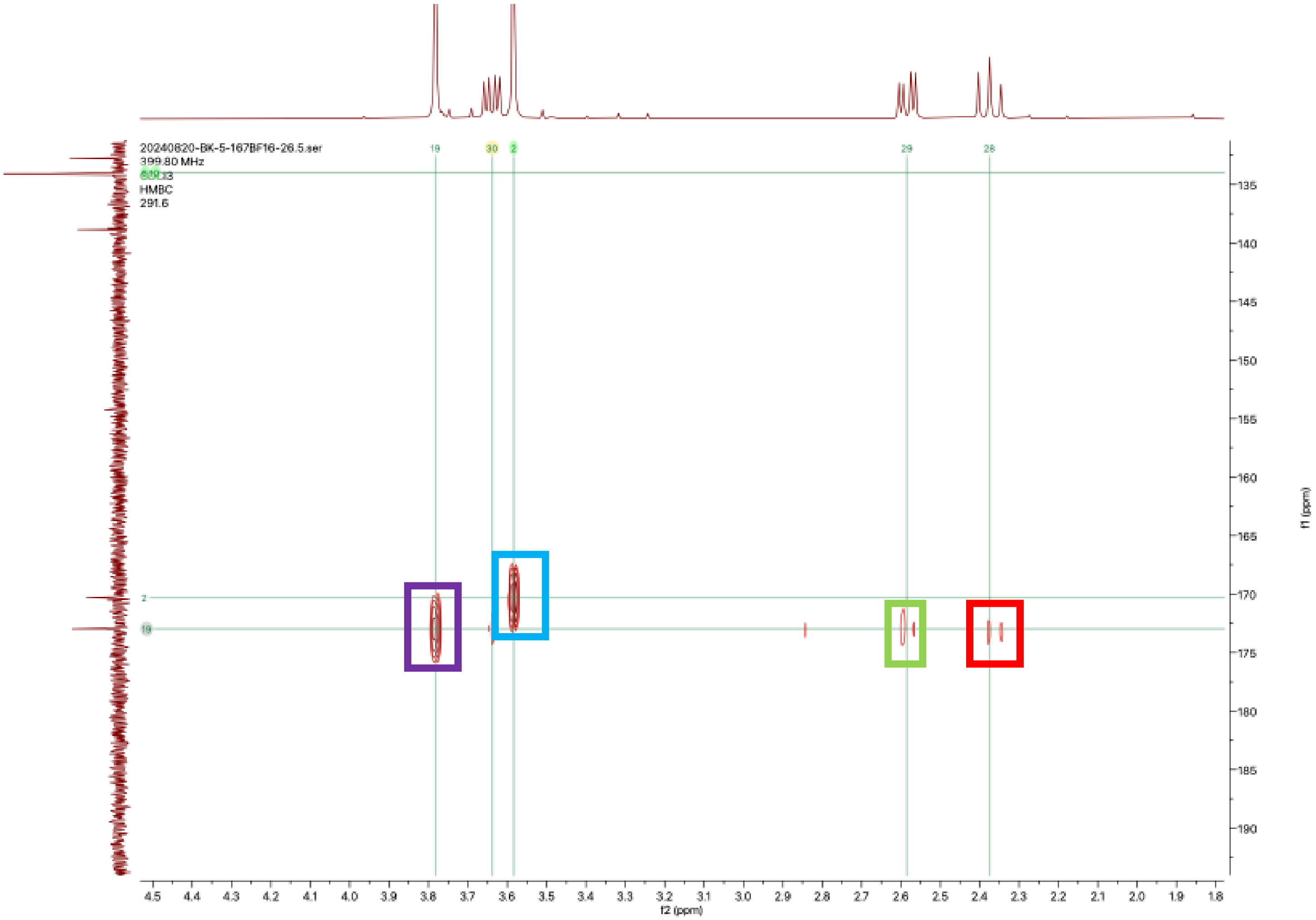

18b *endo syn* minor diastereomer HMBC

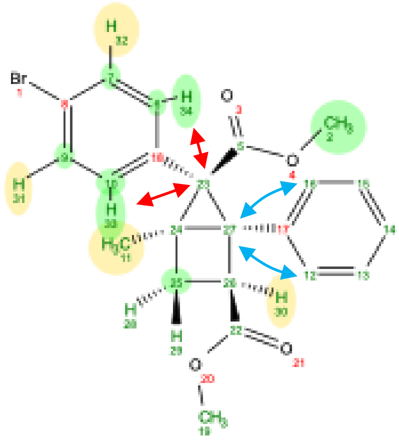

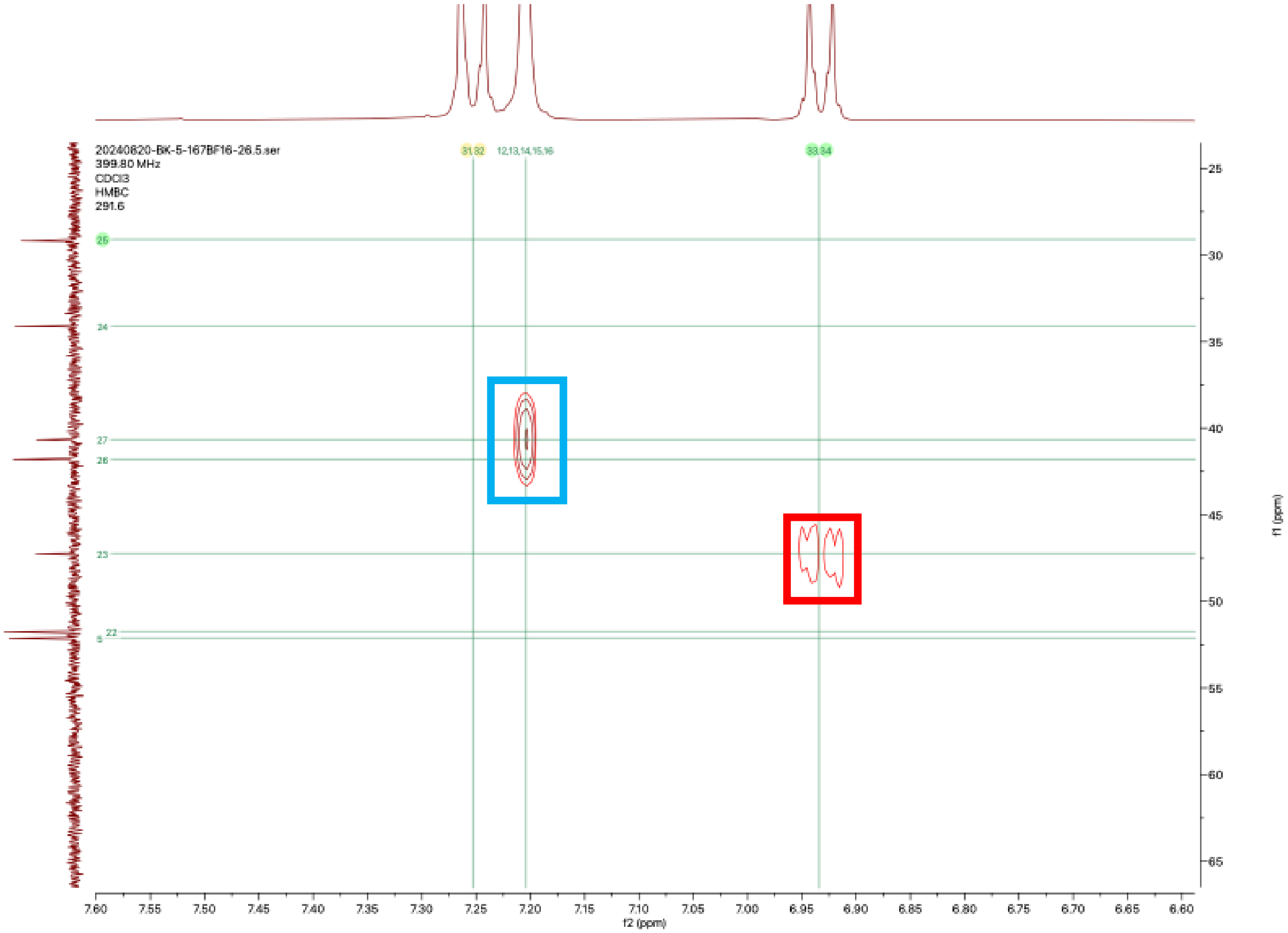

18b *endo syn* minor diastereomer NOESY

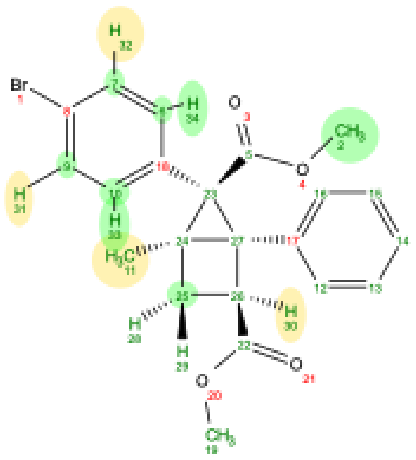

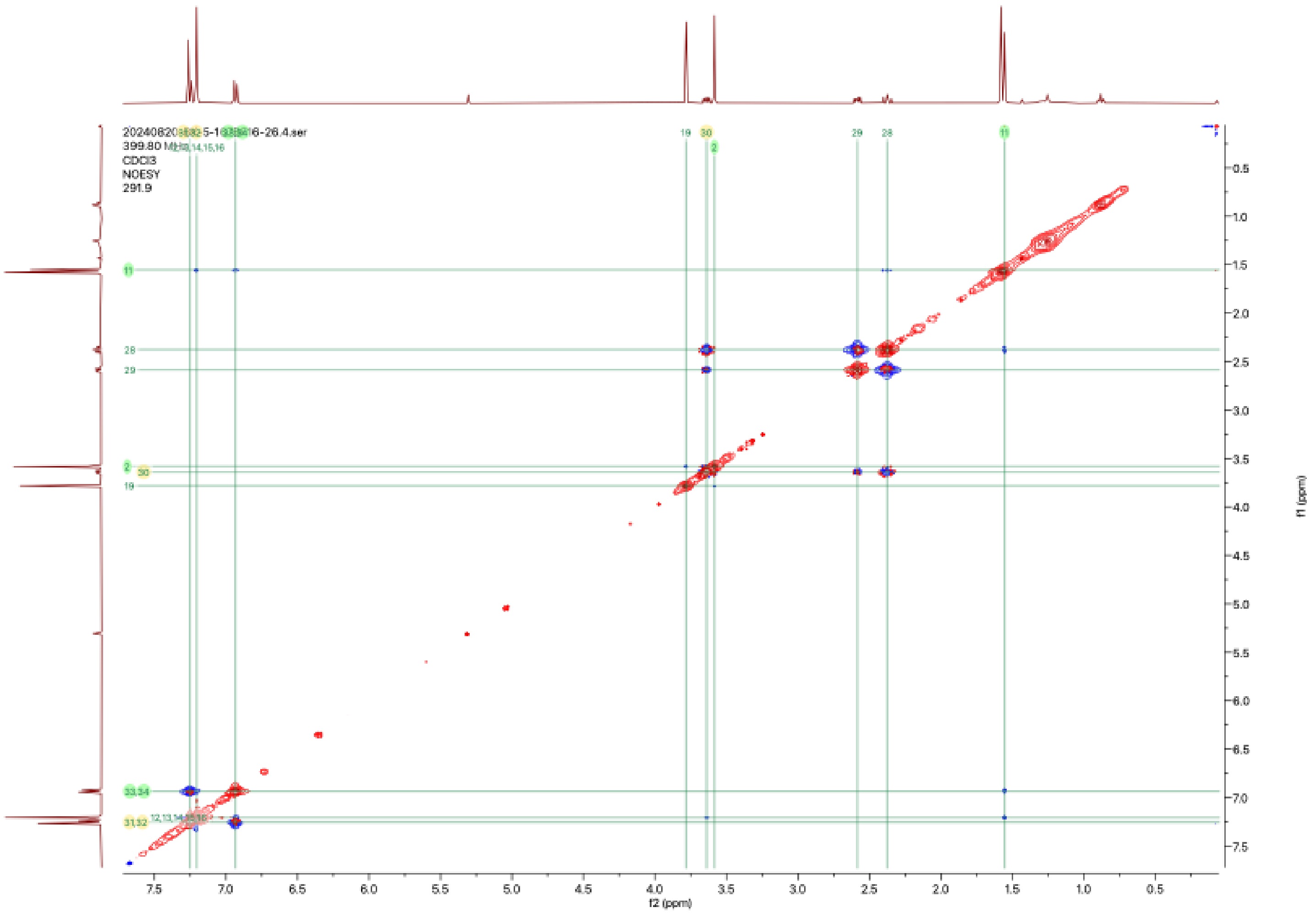

18b *endo syn* minor diastereomer NOESY

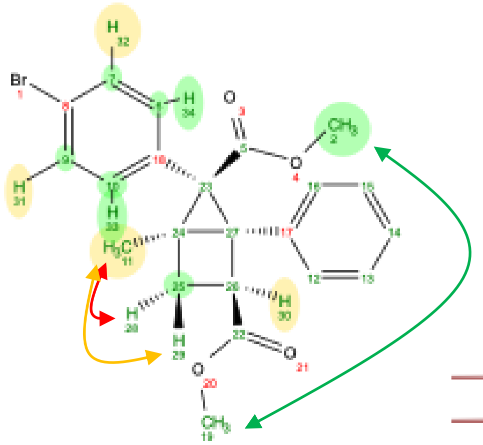

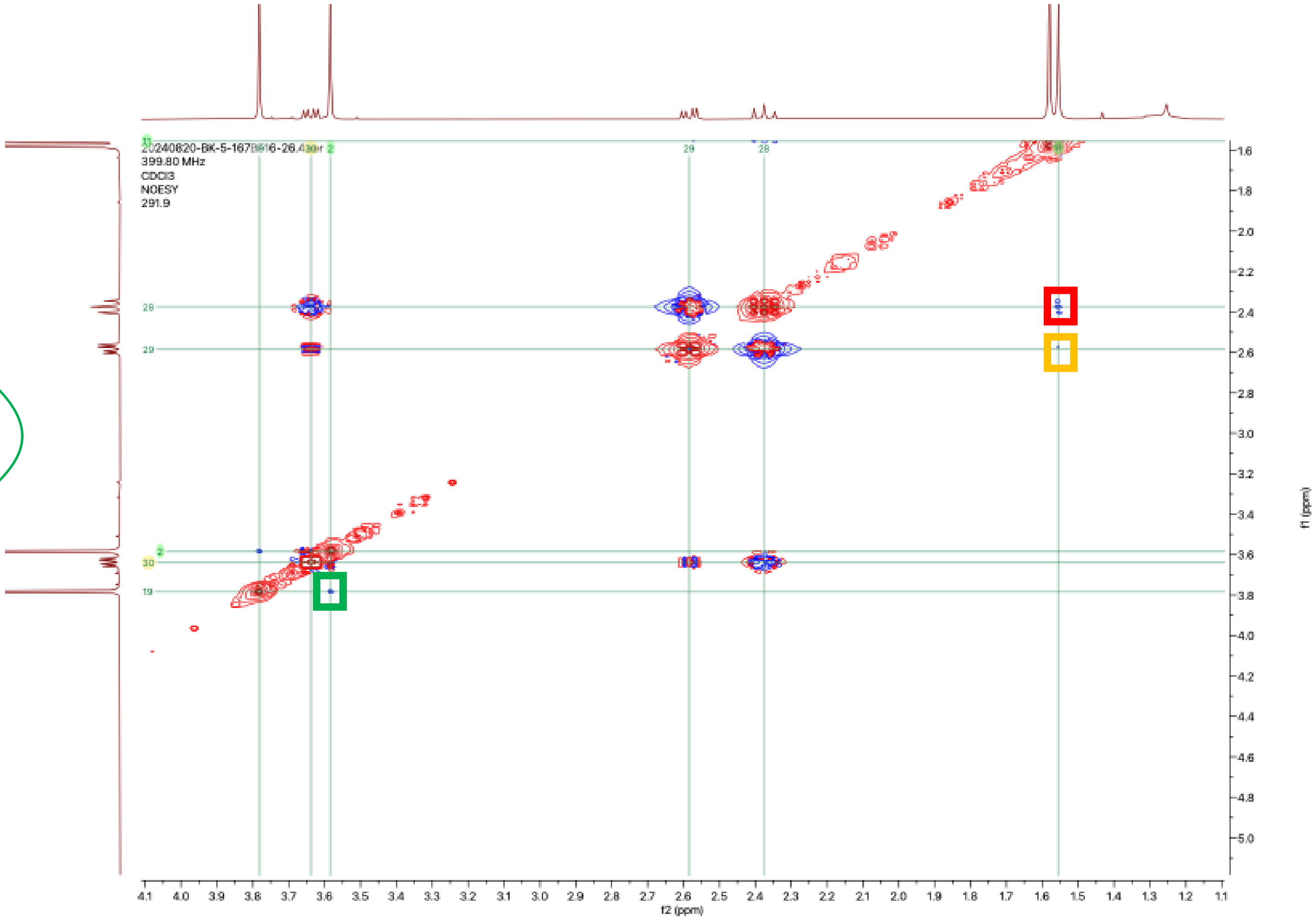

18b *endo syn* minor diastereomer NOESY

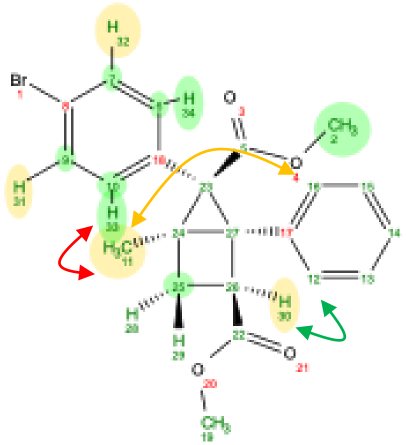

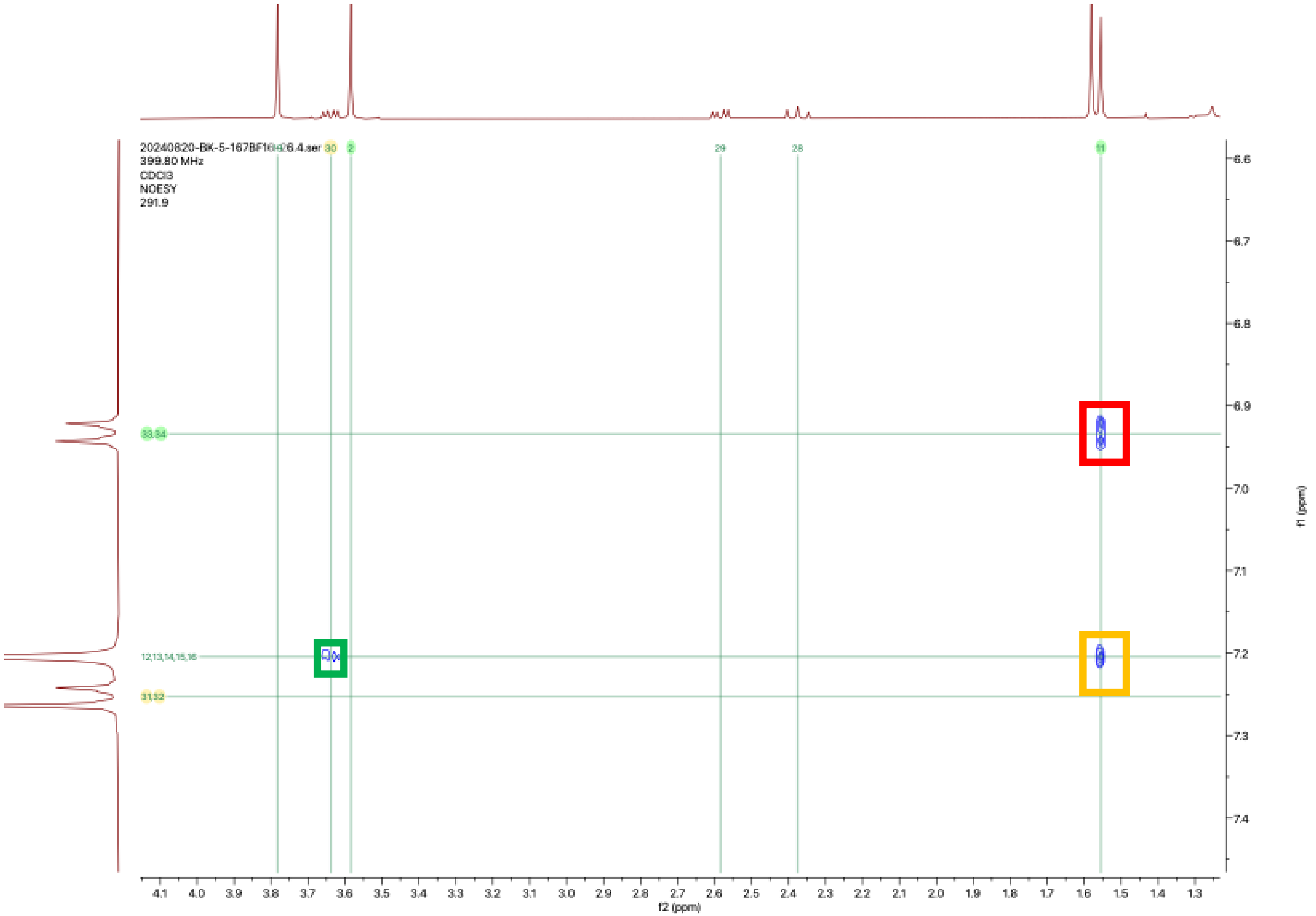

18c *exo anti* ^{1}H NMR minor diastereomer ambient temperature probe

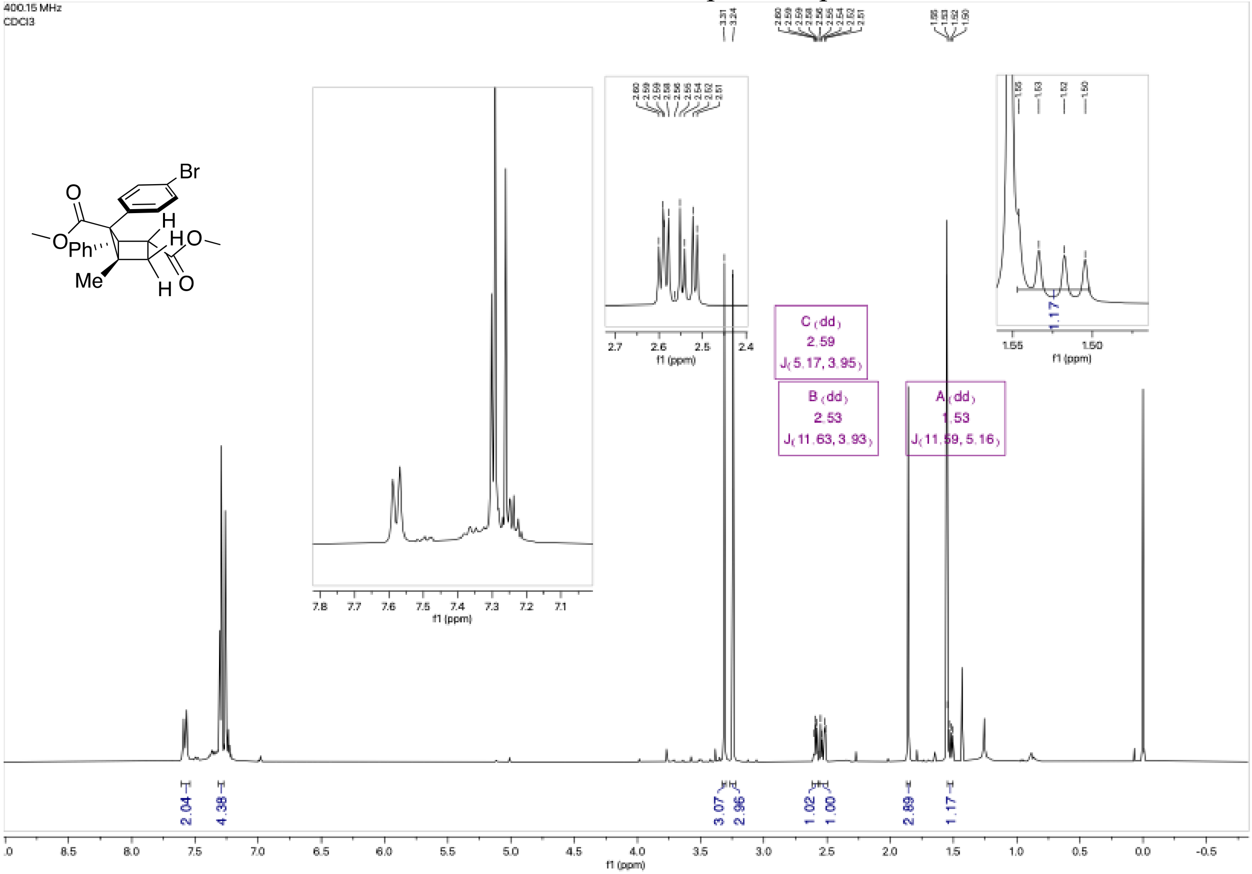

18c *exo anti* ^{1}H NMR minor diastereomer -25 ºC temperature probe

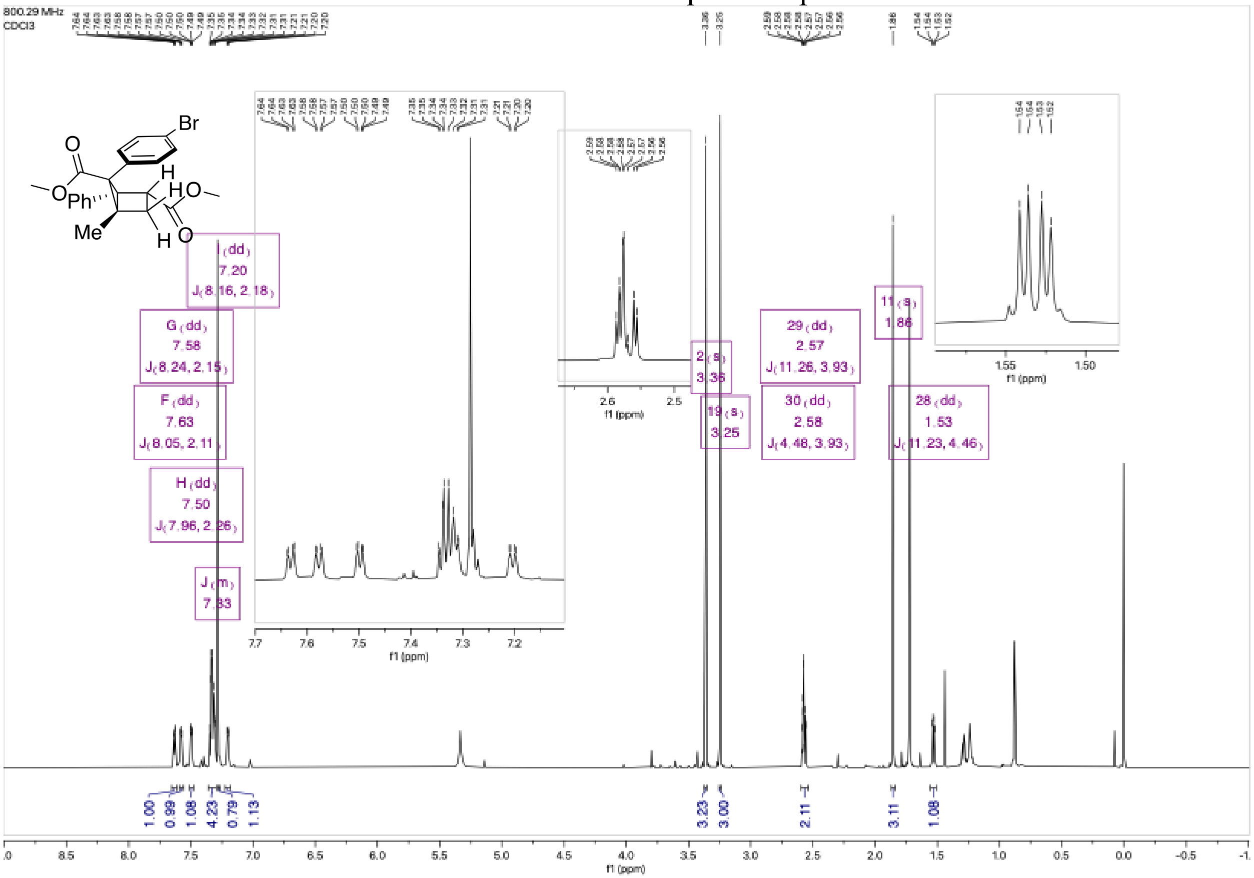

18c *exo anti* ^{13}C NMR minor diastereomer ambient temperature probe

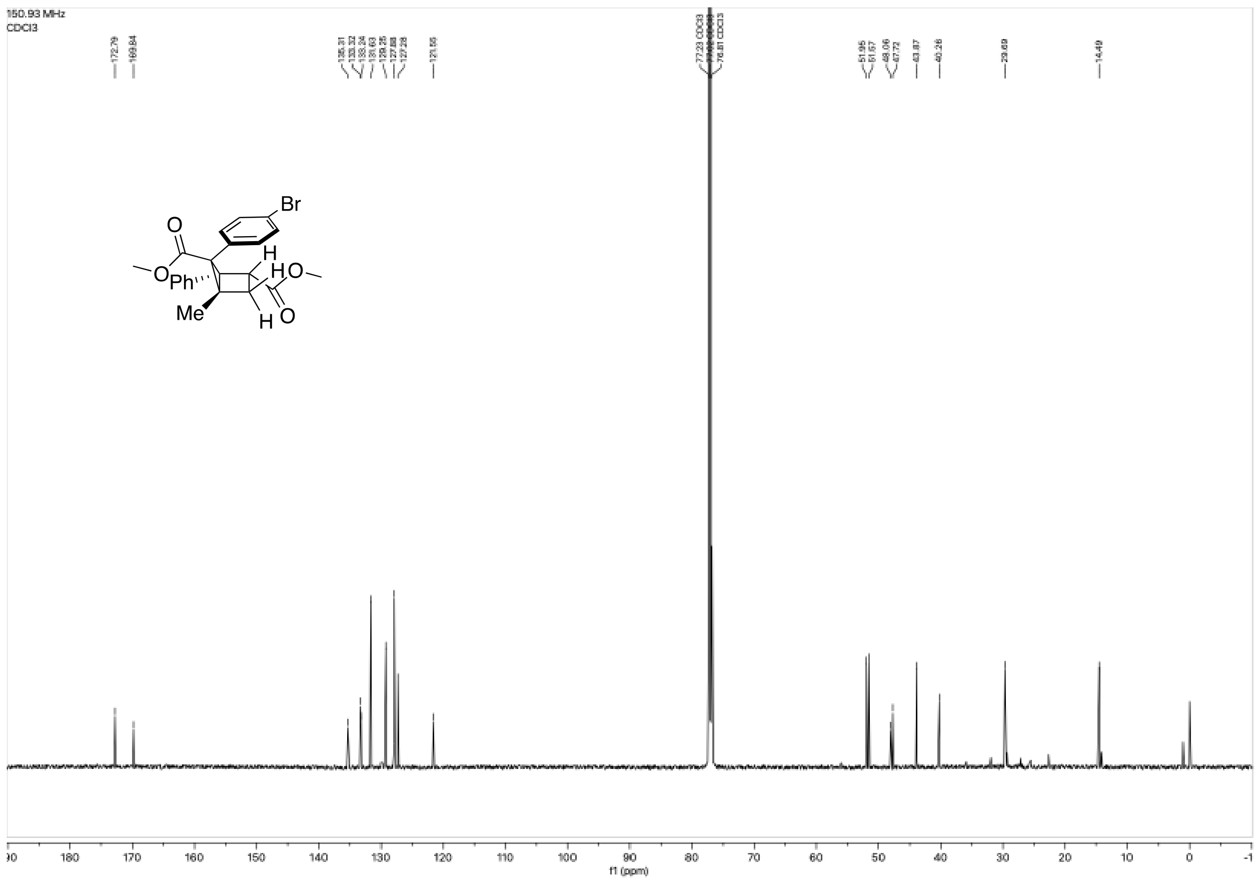

18c *exo anti* minor diastereomer HSQC

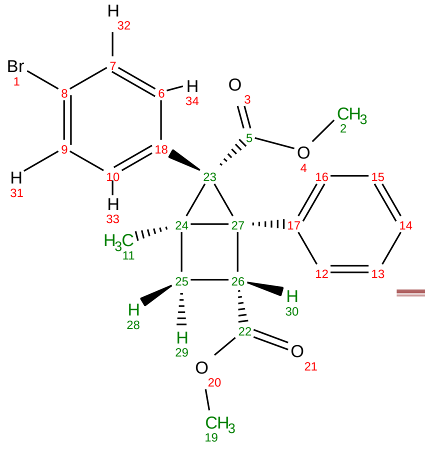

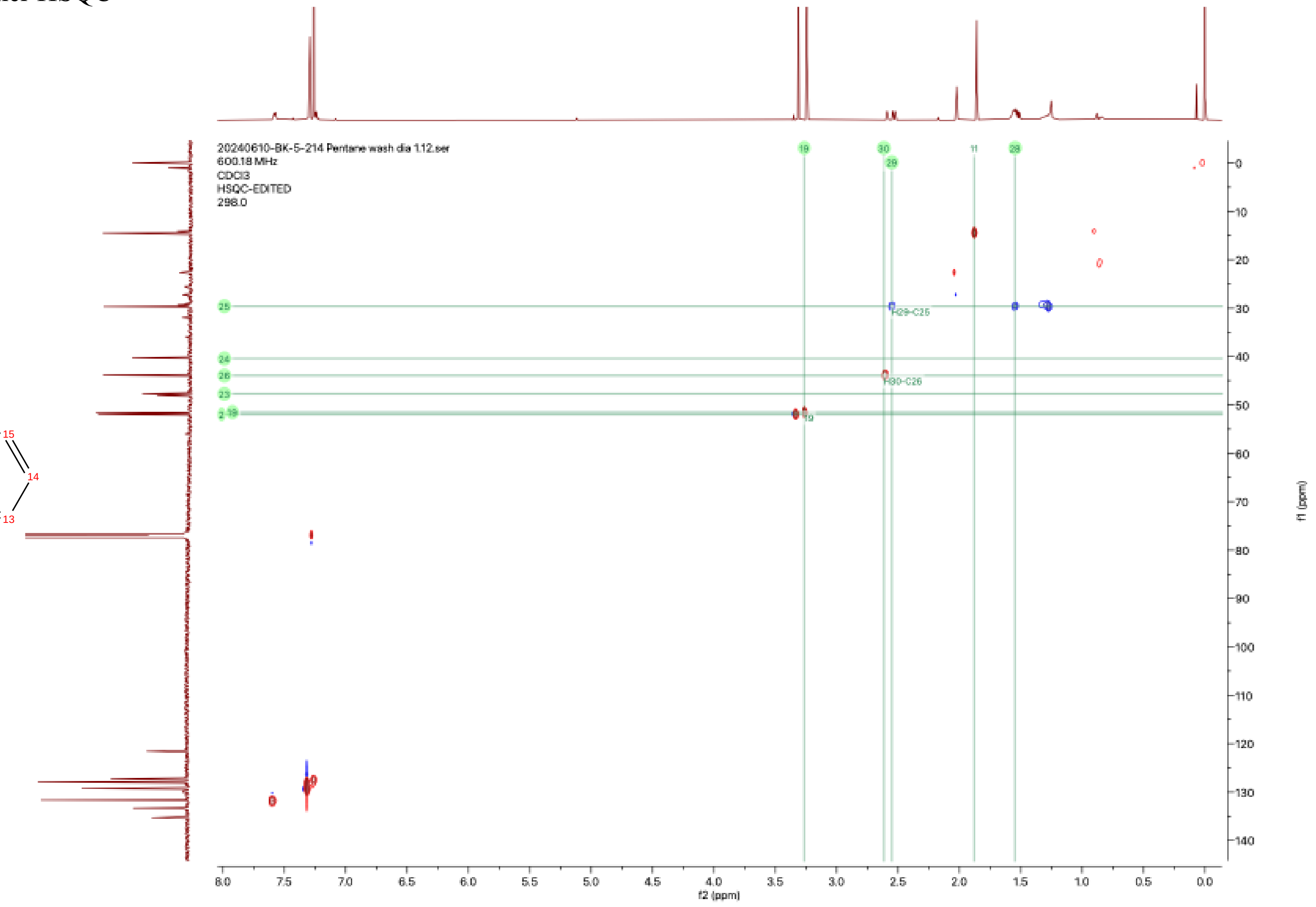

18c *exo anti* minor diastereomer HSQC

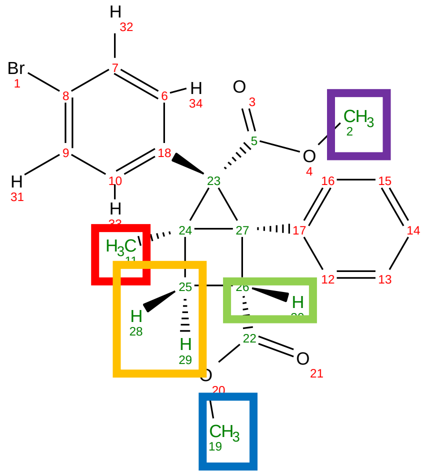

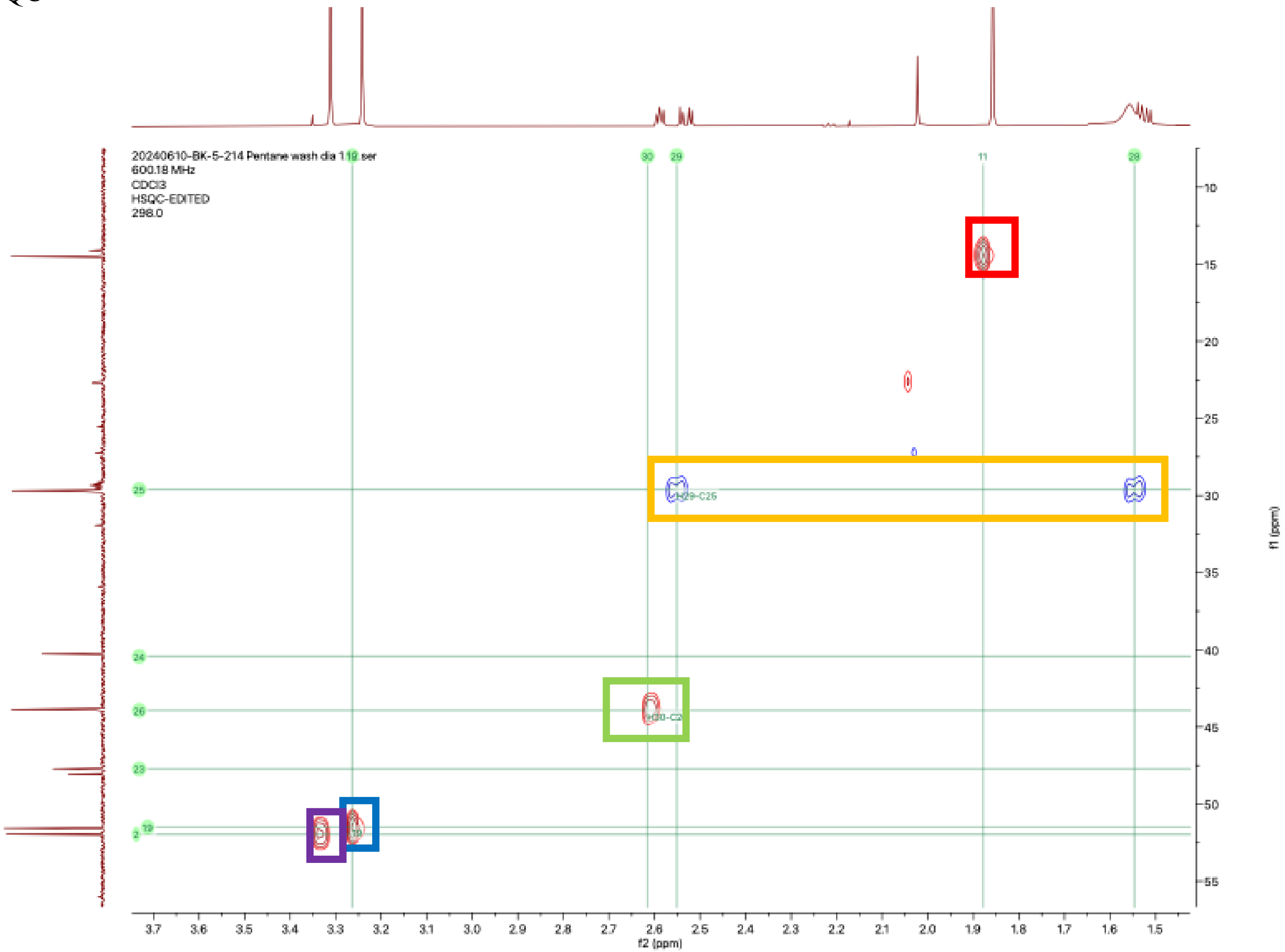

18c *exo anti* minor diastereomer HMBC

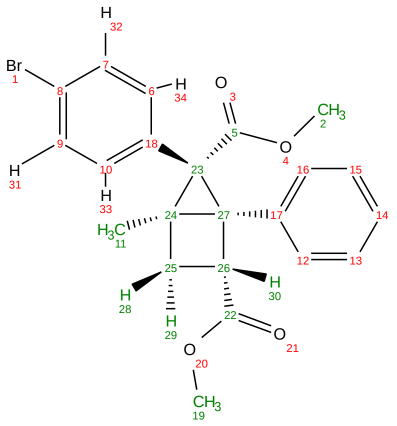

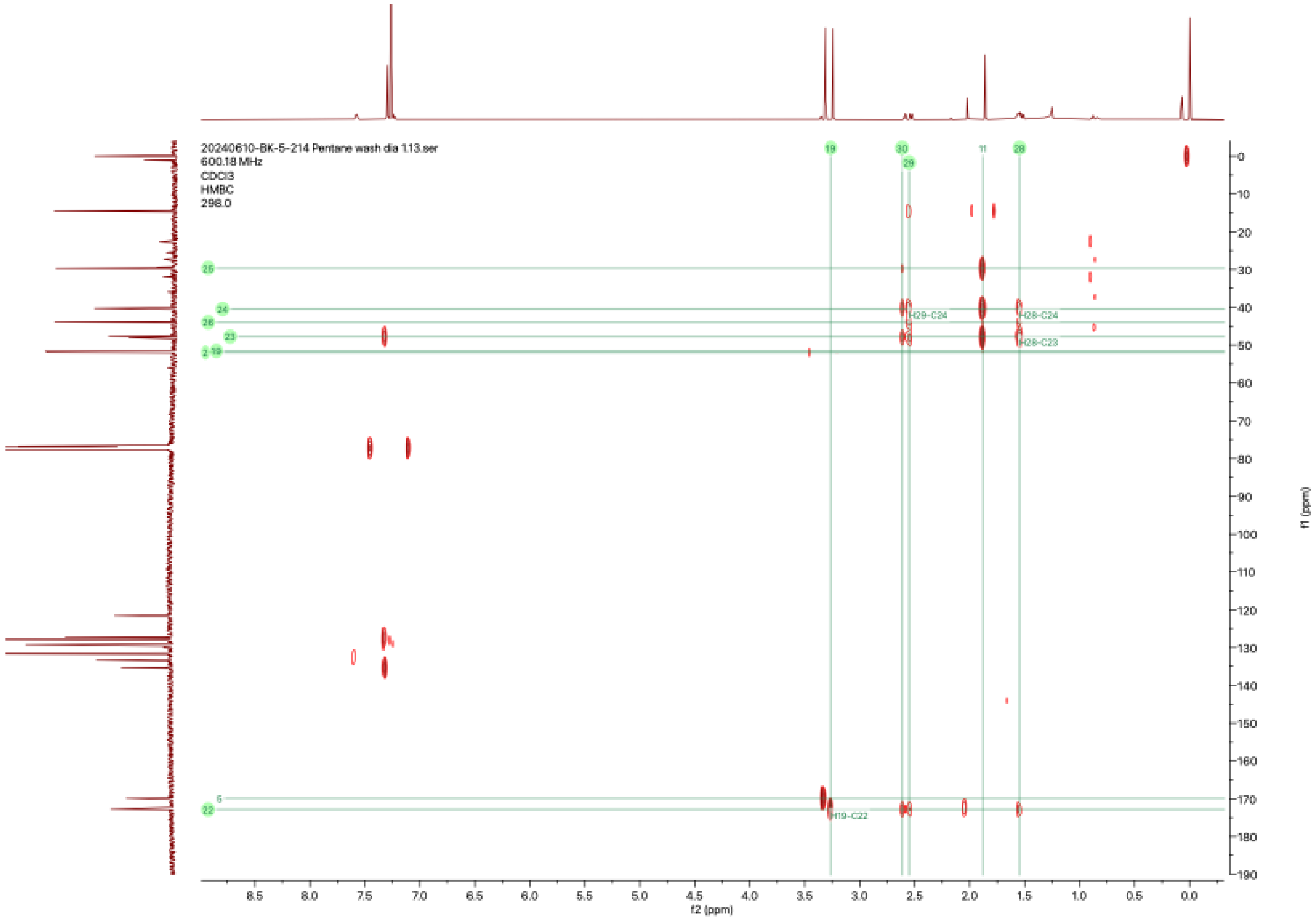

18c *exo anti* minor diastereomer HMBC

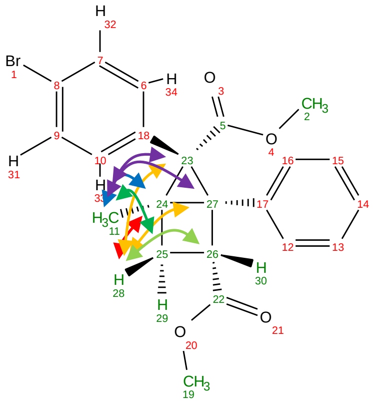

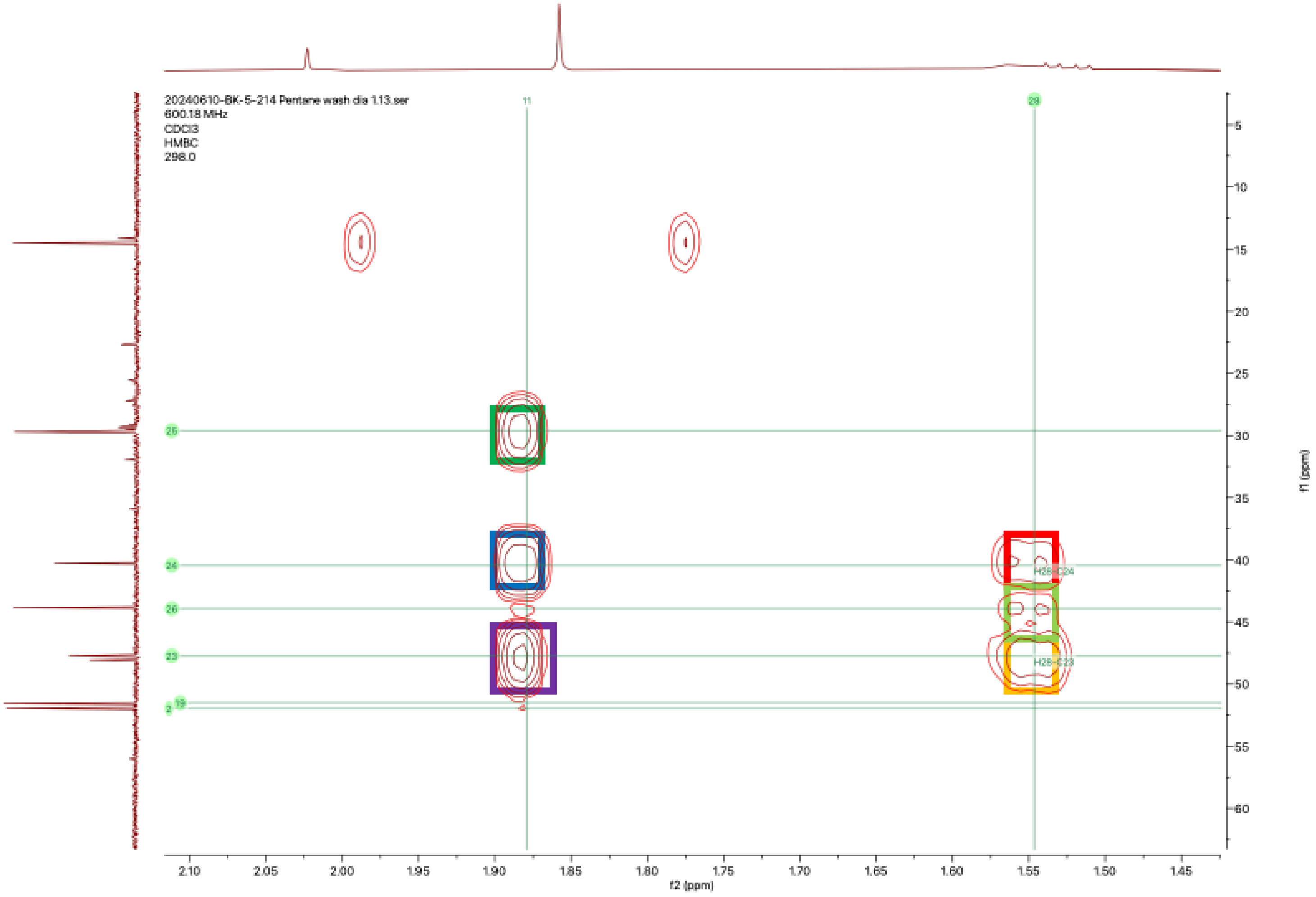

18c *exo anti* minor diastereomer HMBC

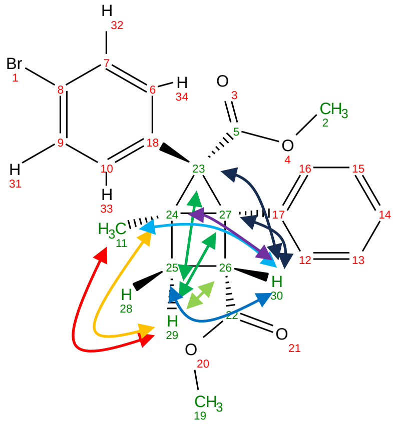

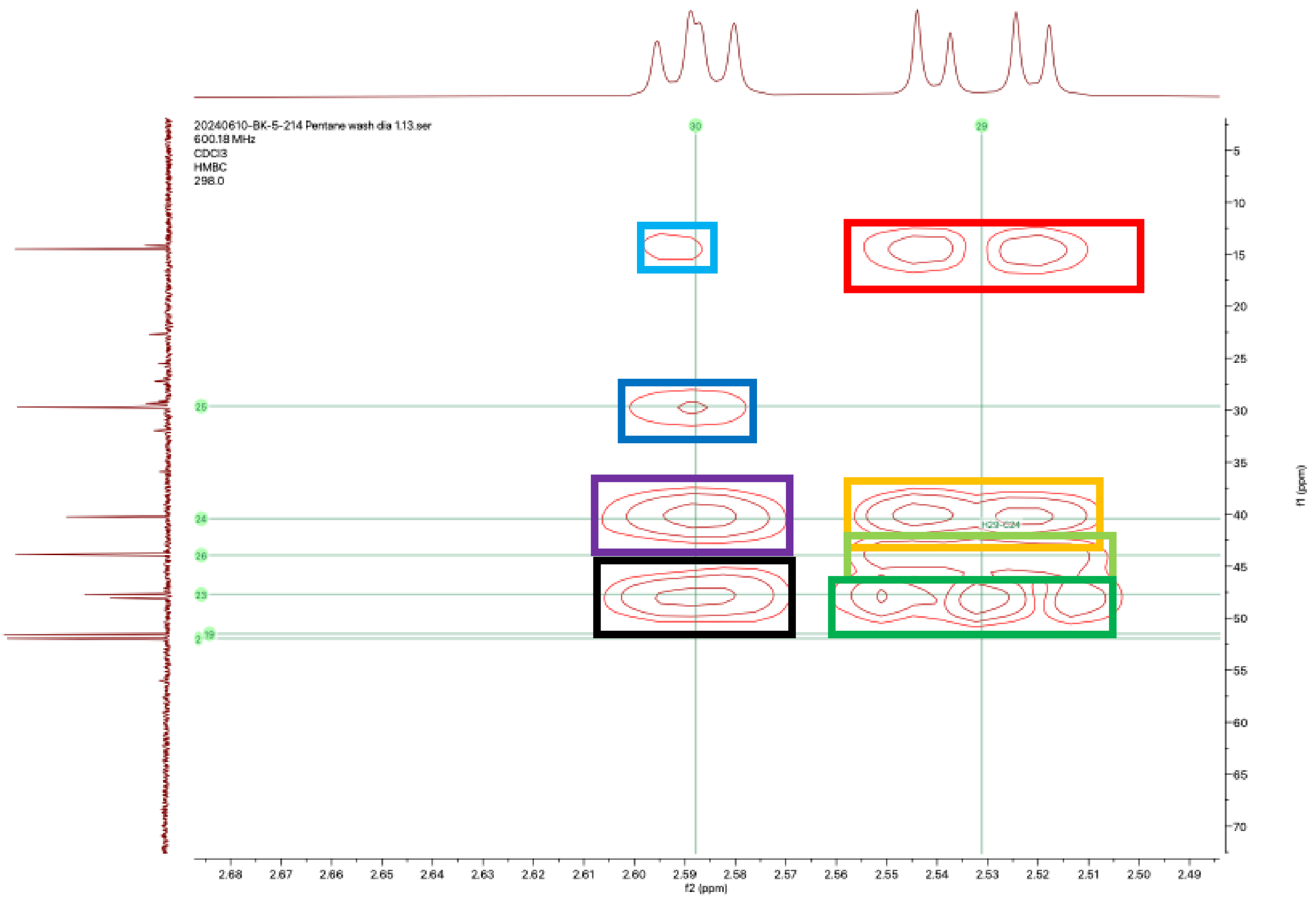

18c *exo anti* minor diastereomer HMBC

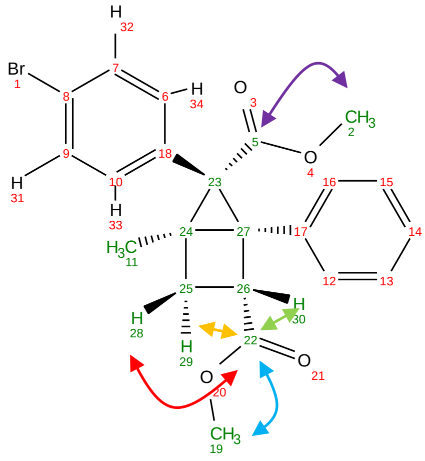

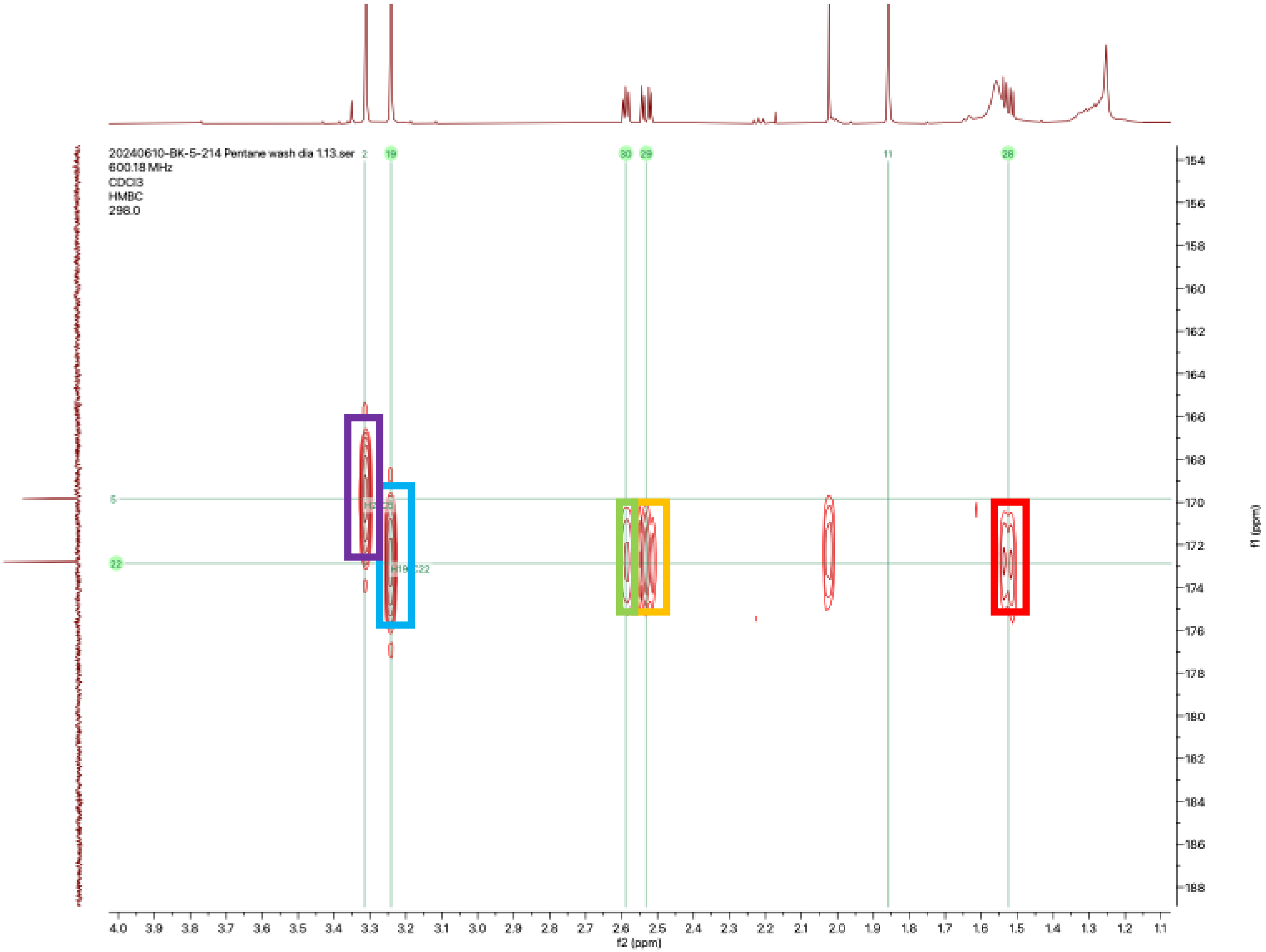

18c *exo anti* minor diastereomer NOESY at -25 ºC

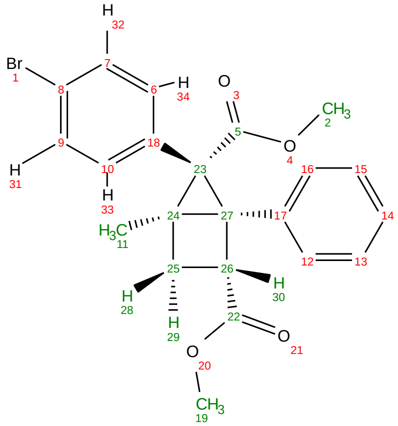

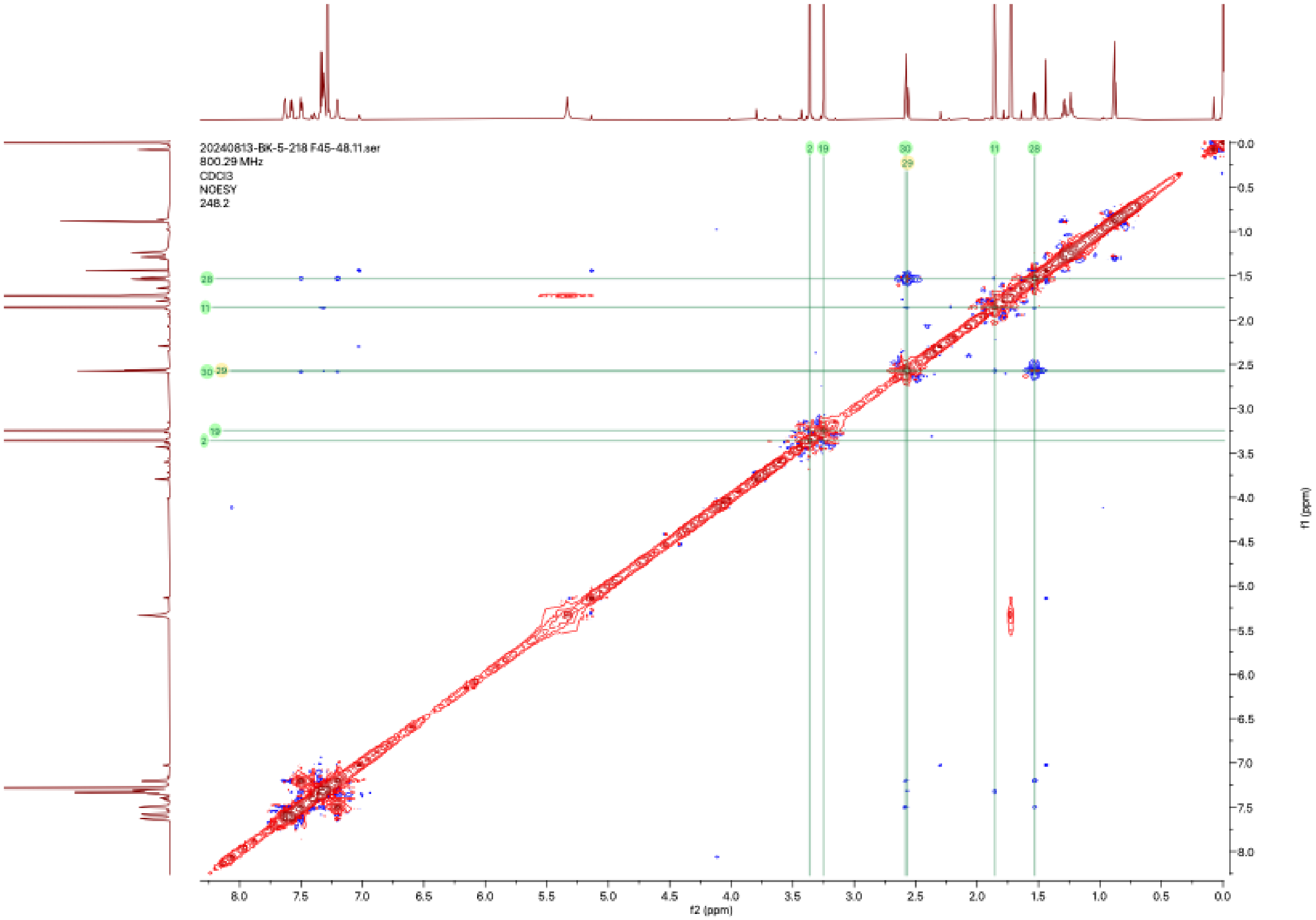

18c *exo anti* minor diastereomer NOESY at -25 ºC

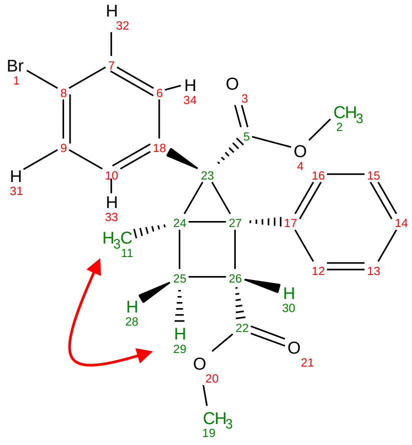

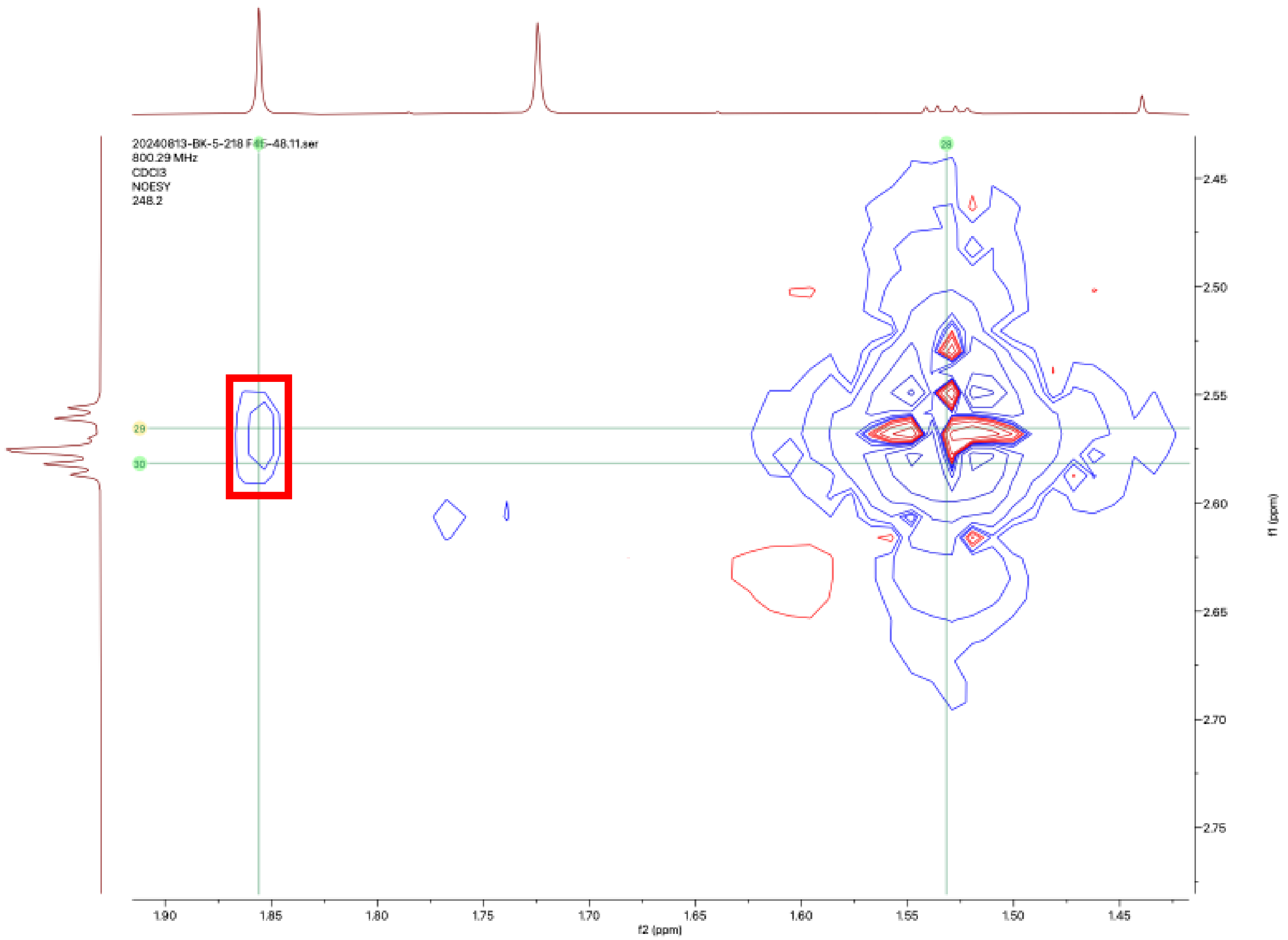

18c *exo anti* minor diastereomer NOESY at -25 ºC

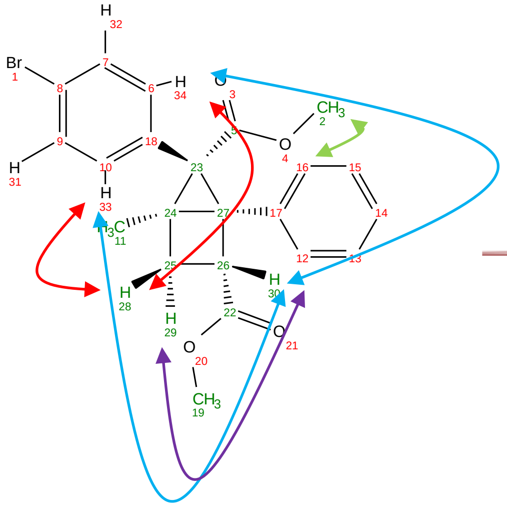

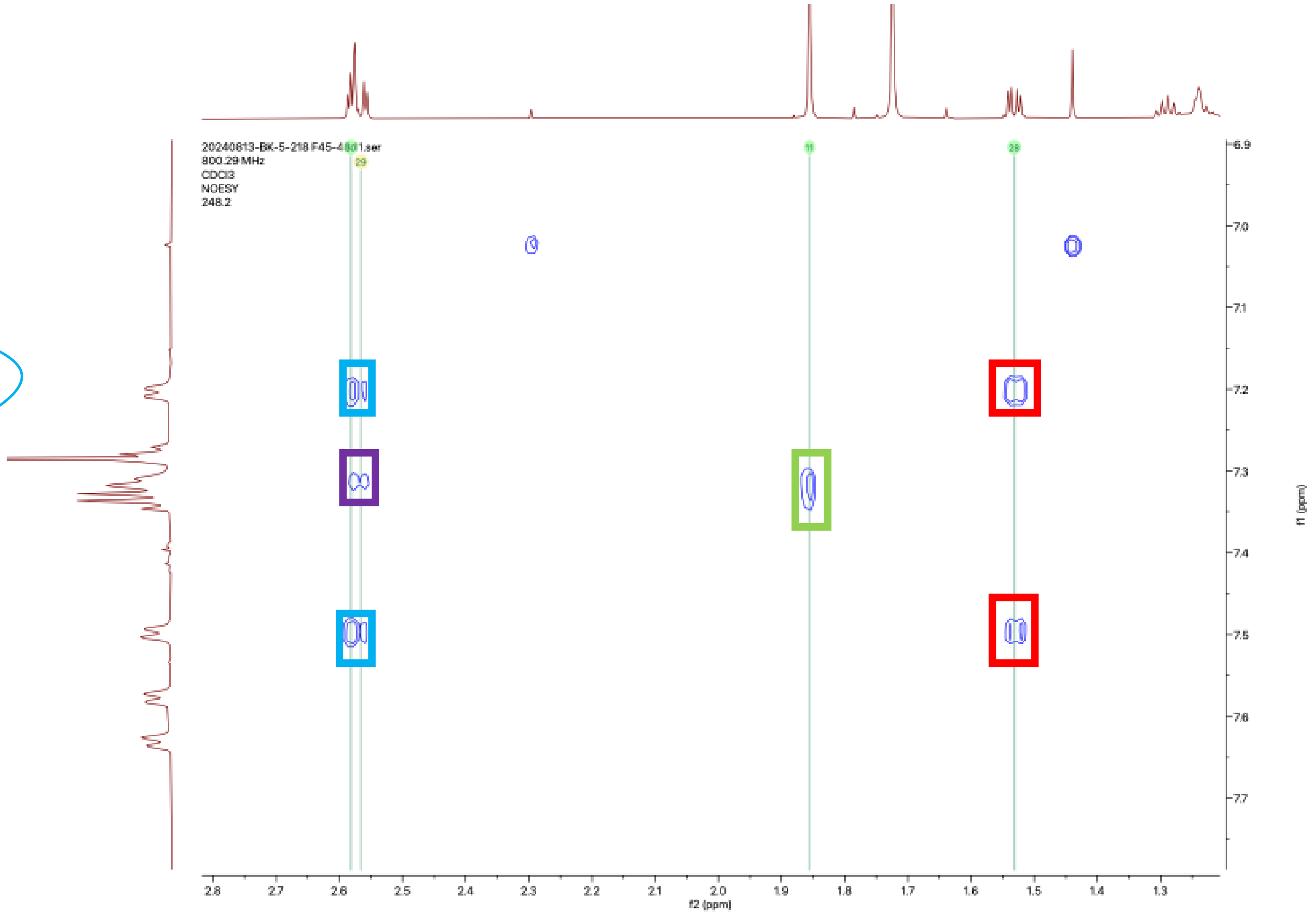

Stacked NMR spectra of **18a**, **18b**, and **18c**

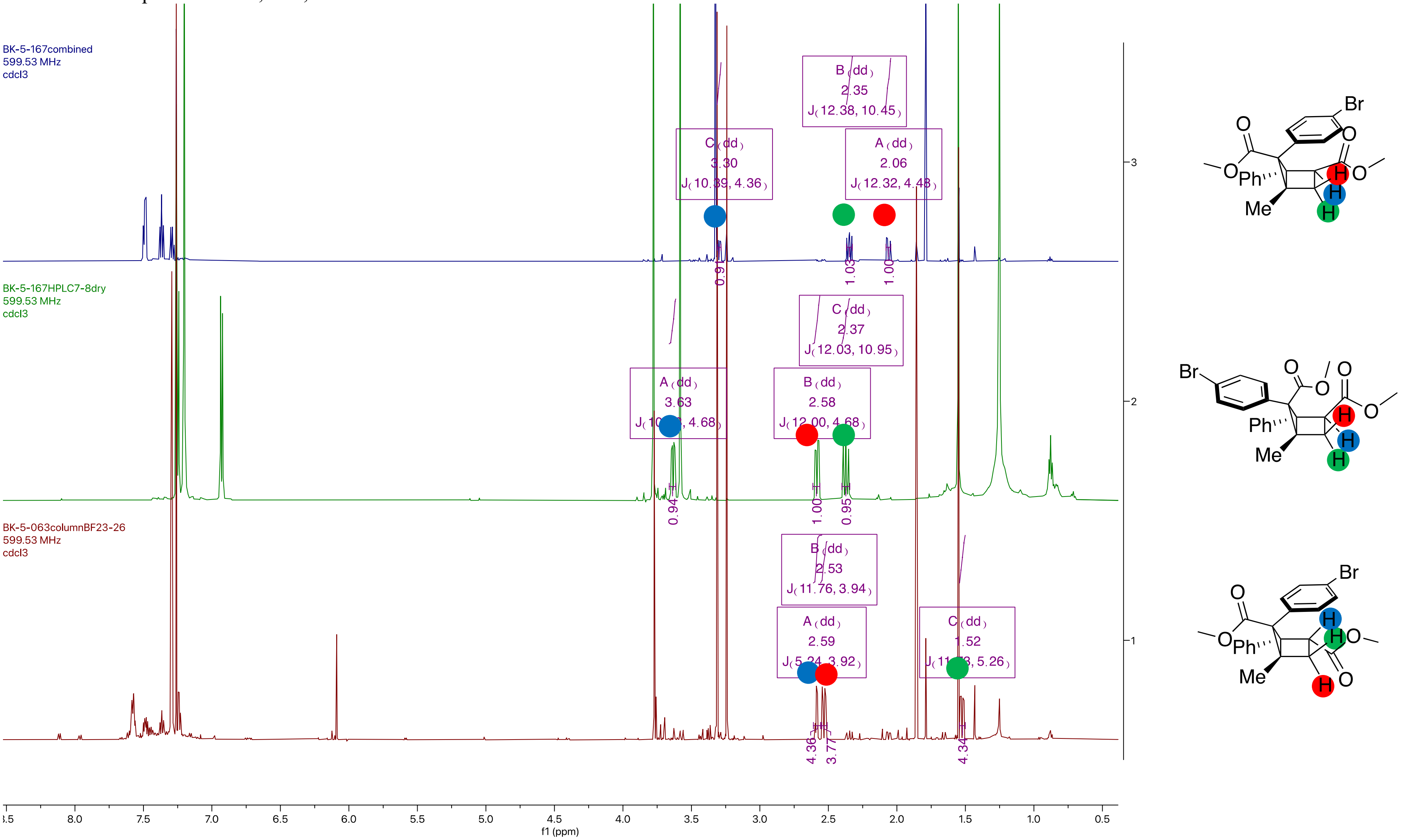

Stacked NMR spectra of **18a**, **18b**, and **18c**

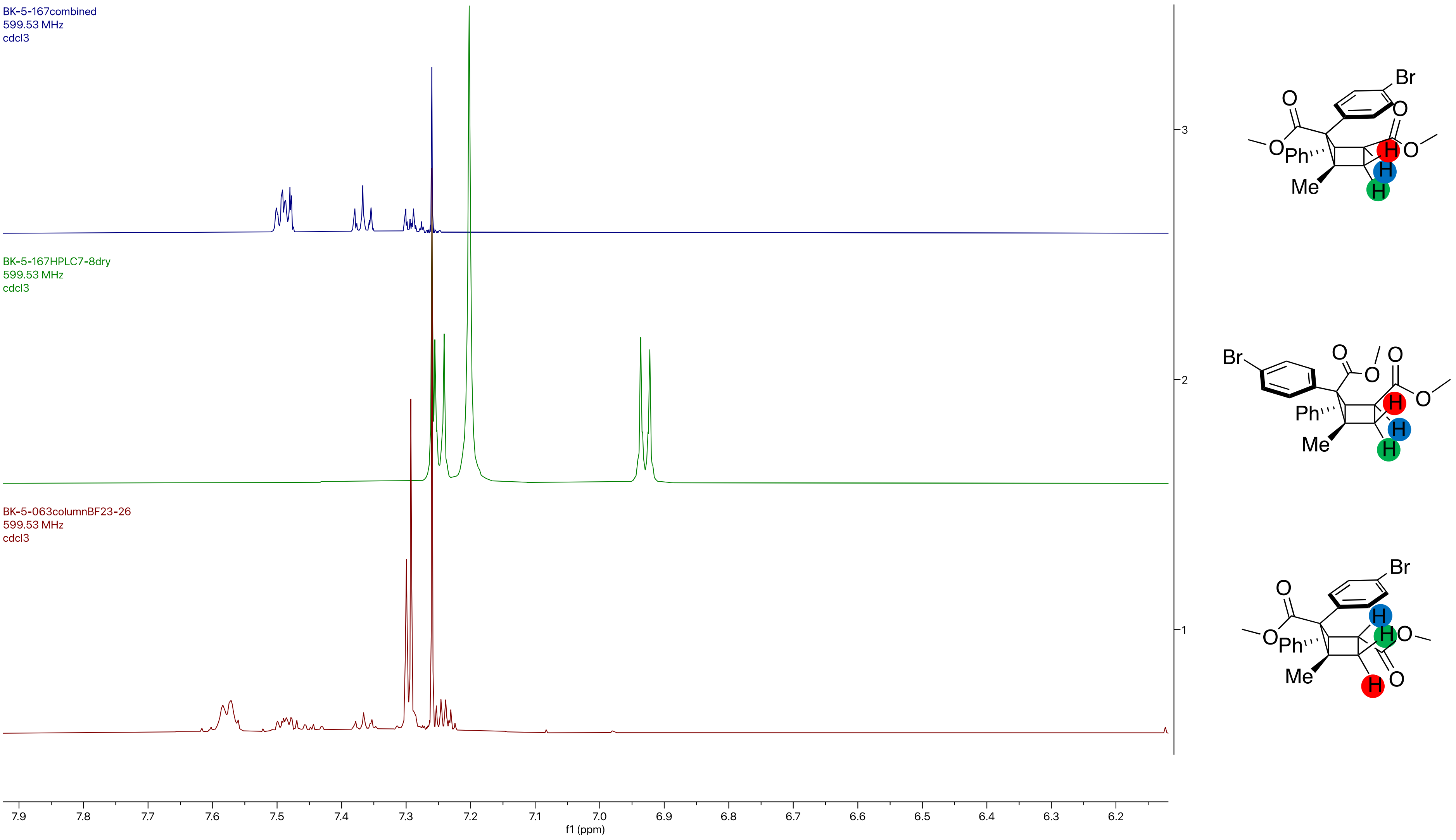

Stacked NMR spectra of **18a**, **18b**, and **18c**

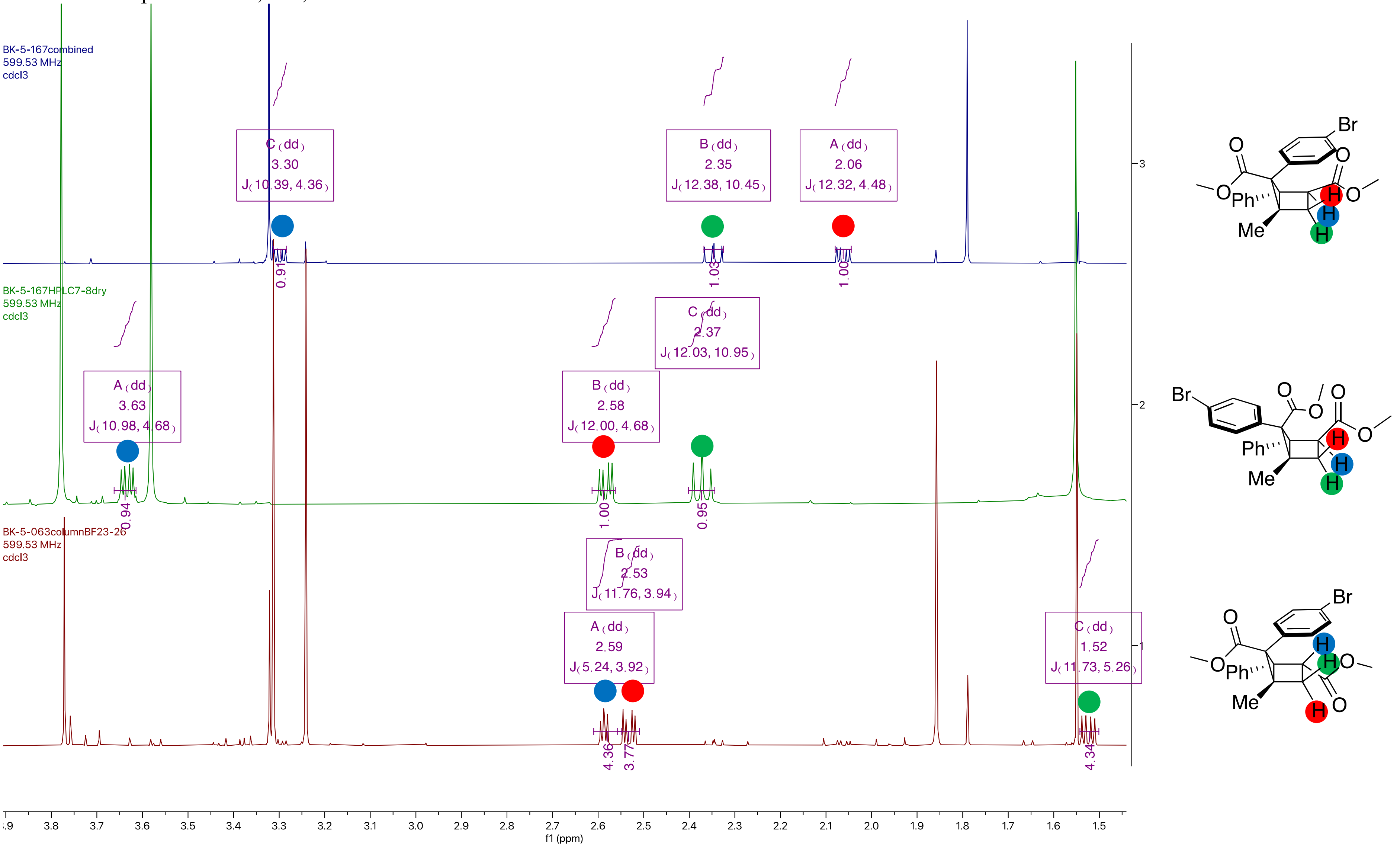

Vb. Crude and Purified Spectra and Diastereomeric Ratios

Crude reaction forming **18a:18b:18c; 86:9:5 d.r.**

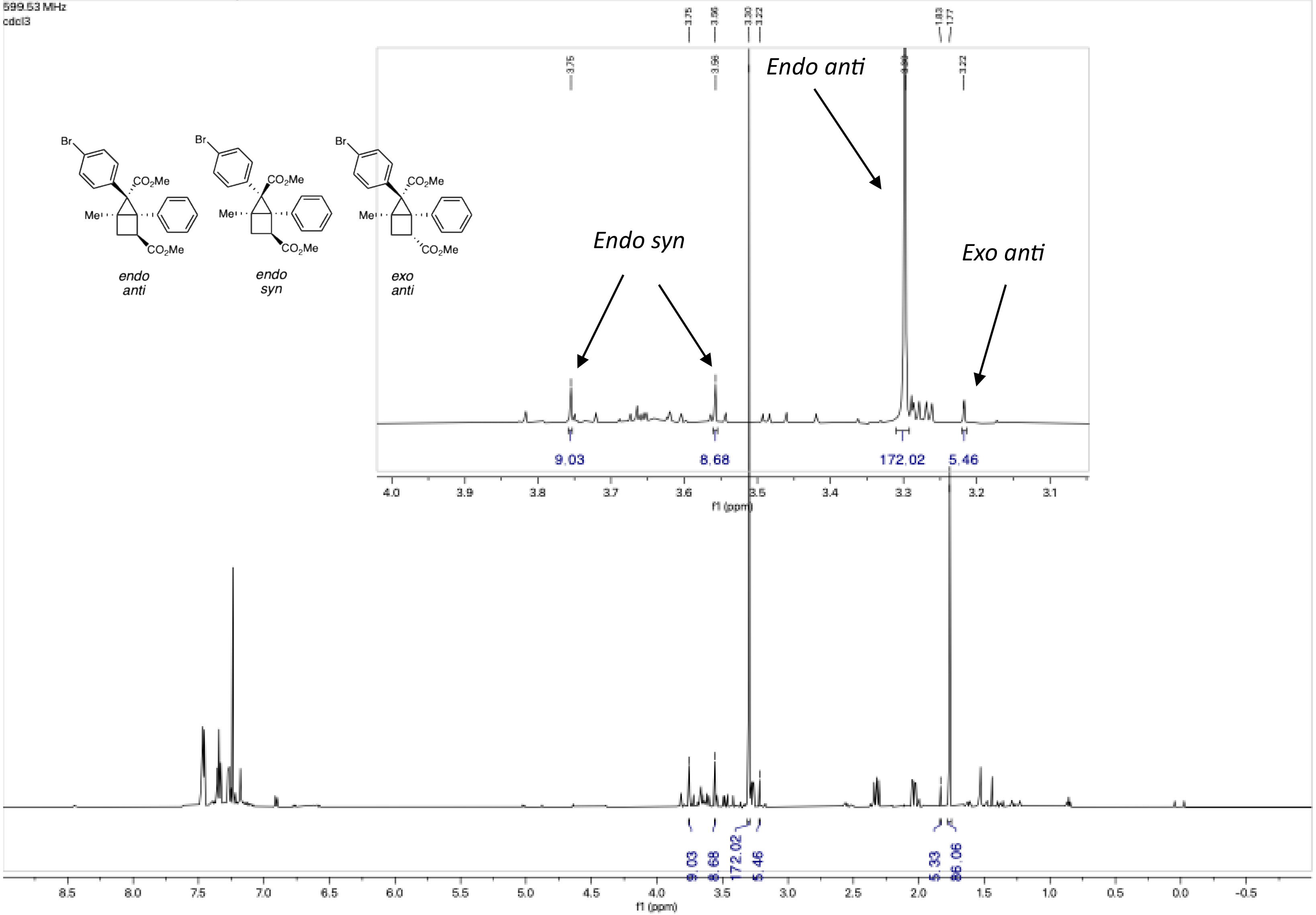

Purified yield **18a:18c >95:5 d.r.**

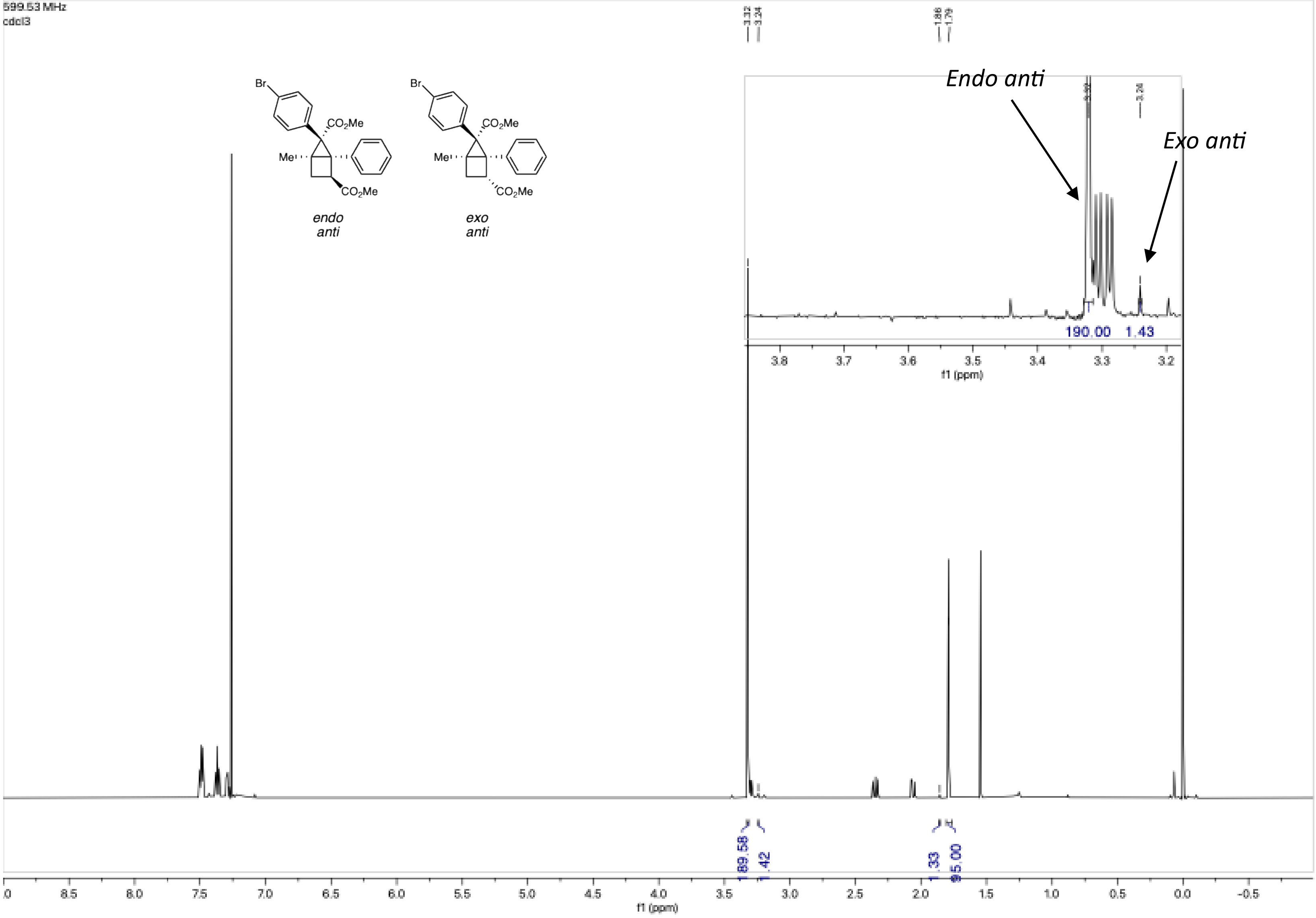

Crude reaction forming **21a** as a mixture of diastereomers *endo anti:endo syn:exo anti* **75:18:7 d.r.**

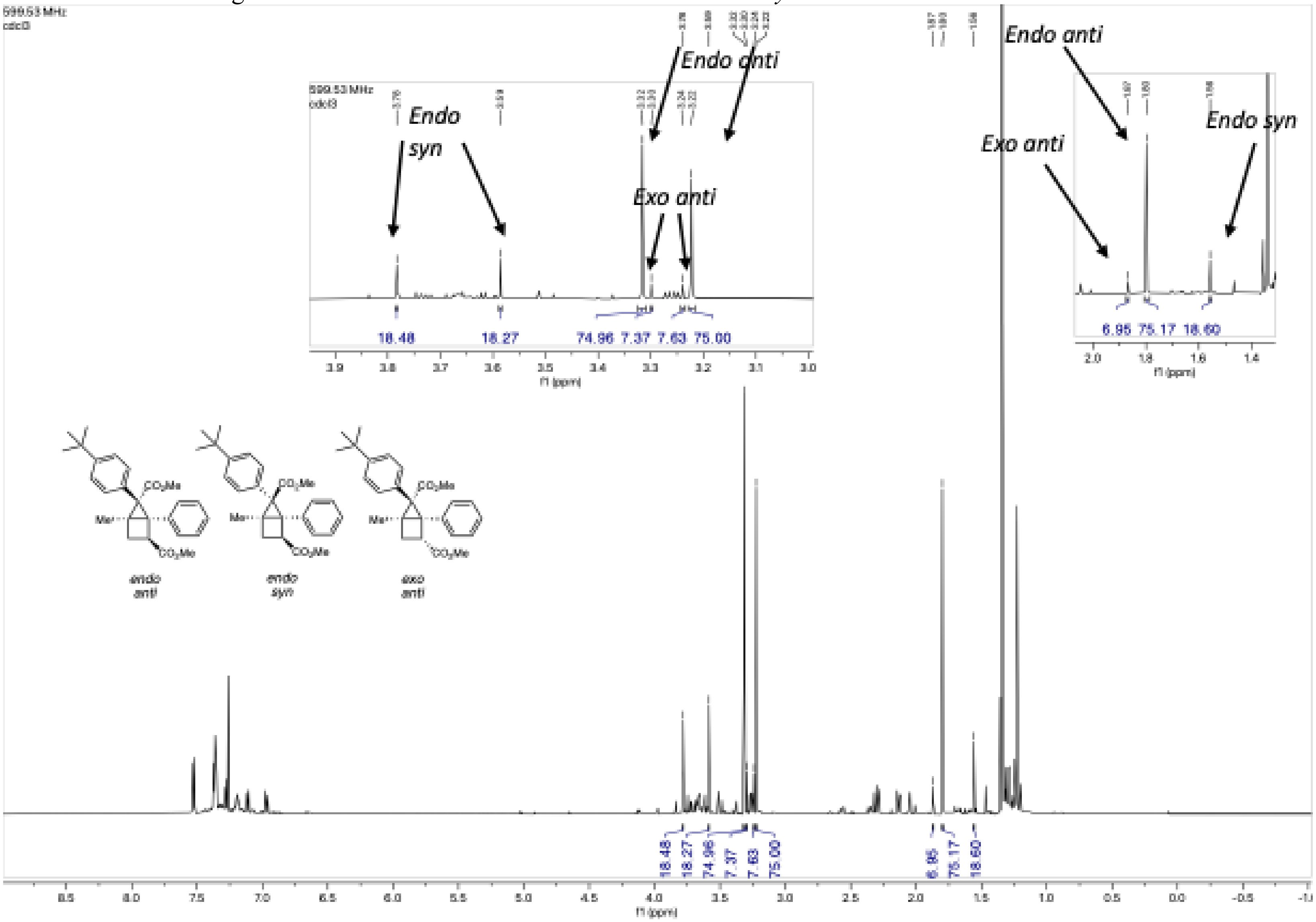

Purified yield of **21a** as a mixture of diastereomers *endo anti:exo anti* **92:8 d.r.**

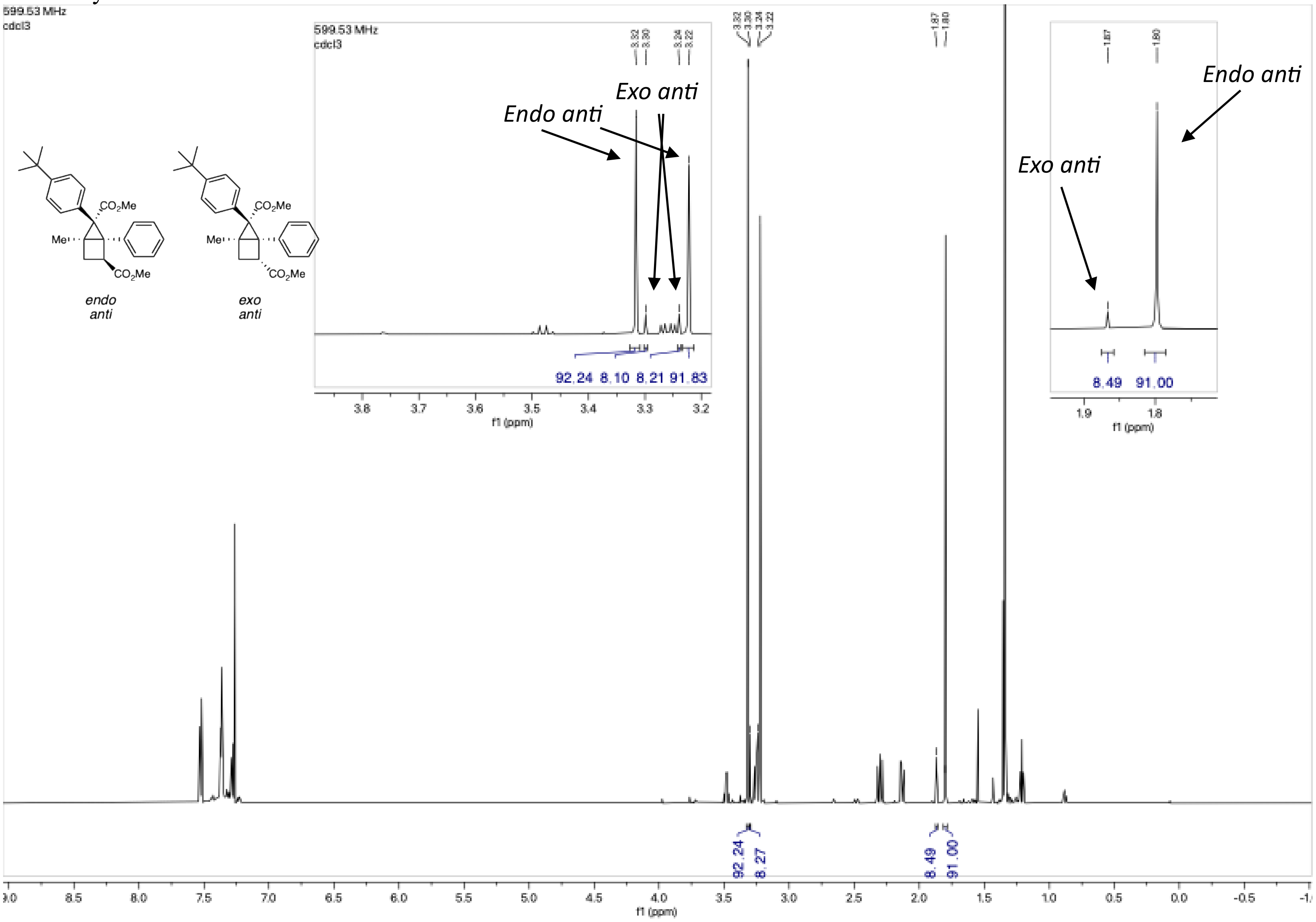

Crude reaction forming **21b** as a mixture of diastereomers *endo anti:endo syn:exo anti* **86:6:8 d.r.**

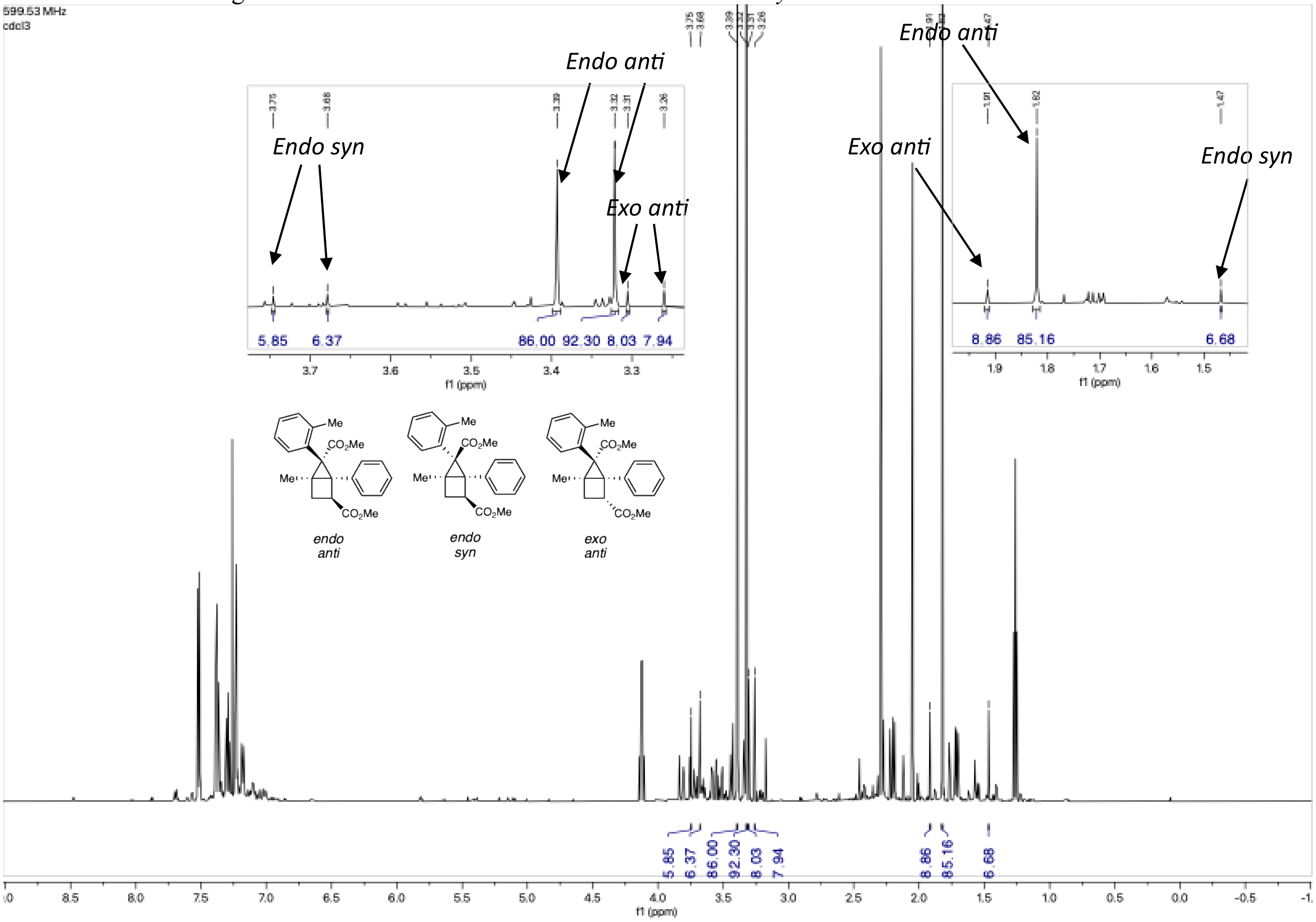

Purified yield of **21b** as a mixture of diastereomers *endo anti:exo anti* >**95:5 d.r.**

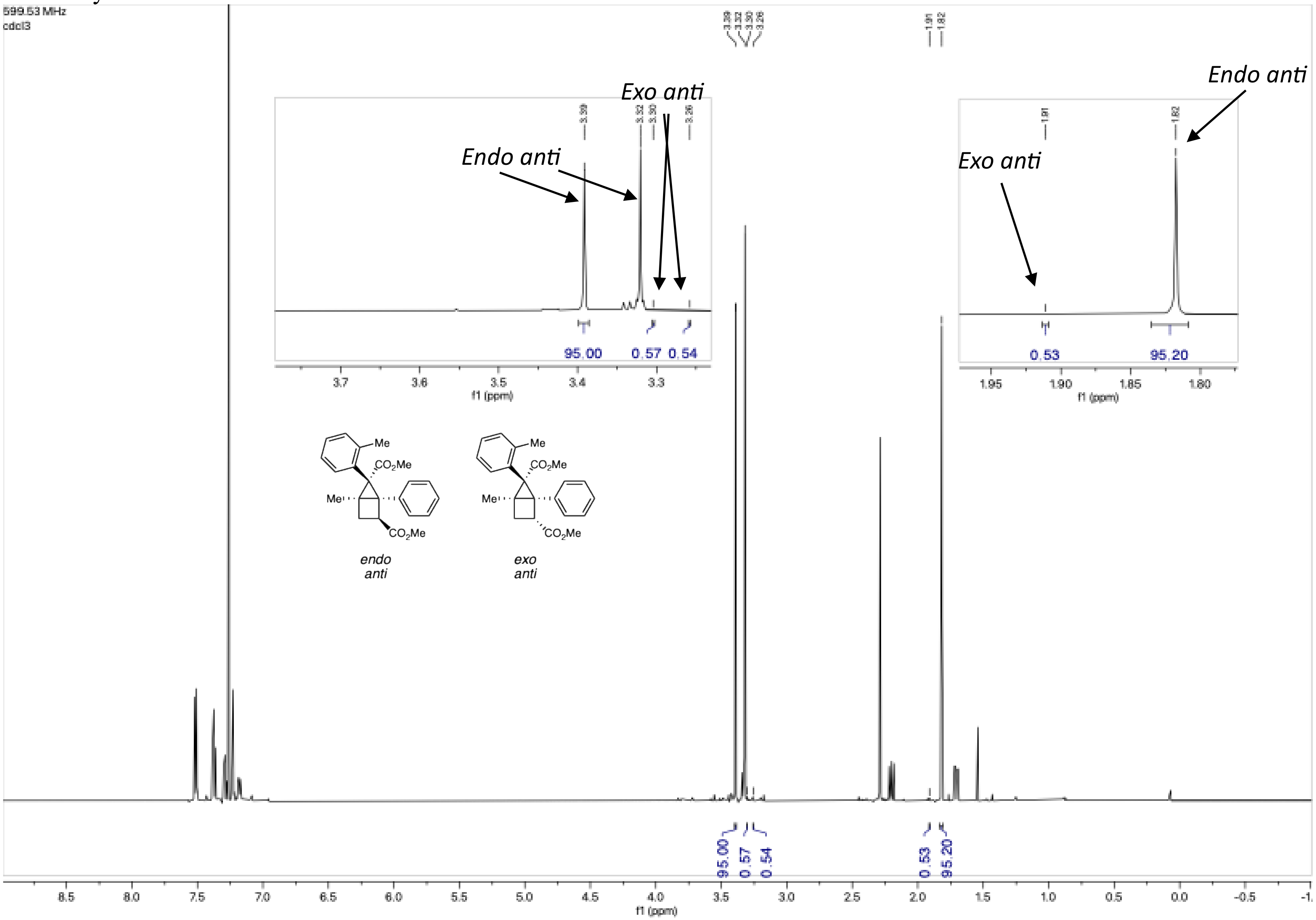

Crude reaction forming **21c** as a mixture of diastereomers *endo anti:endo syn:exo anti* **84:10:6 d.r.**

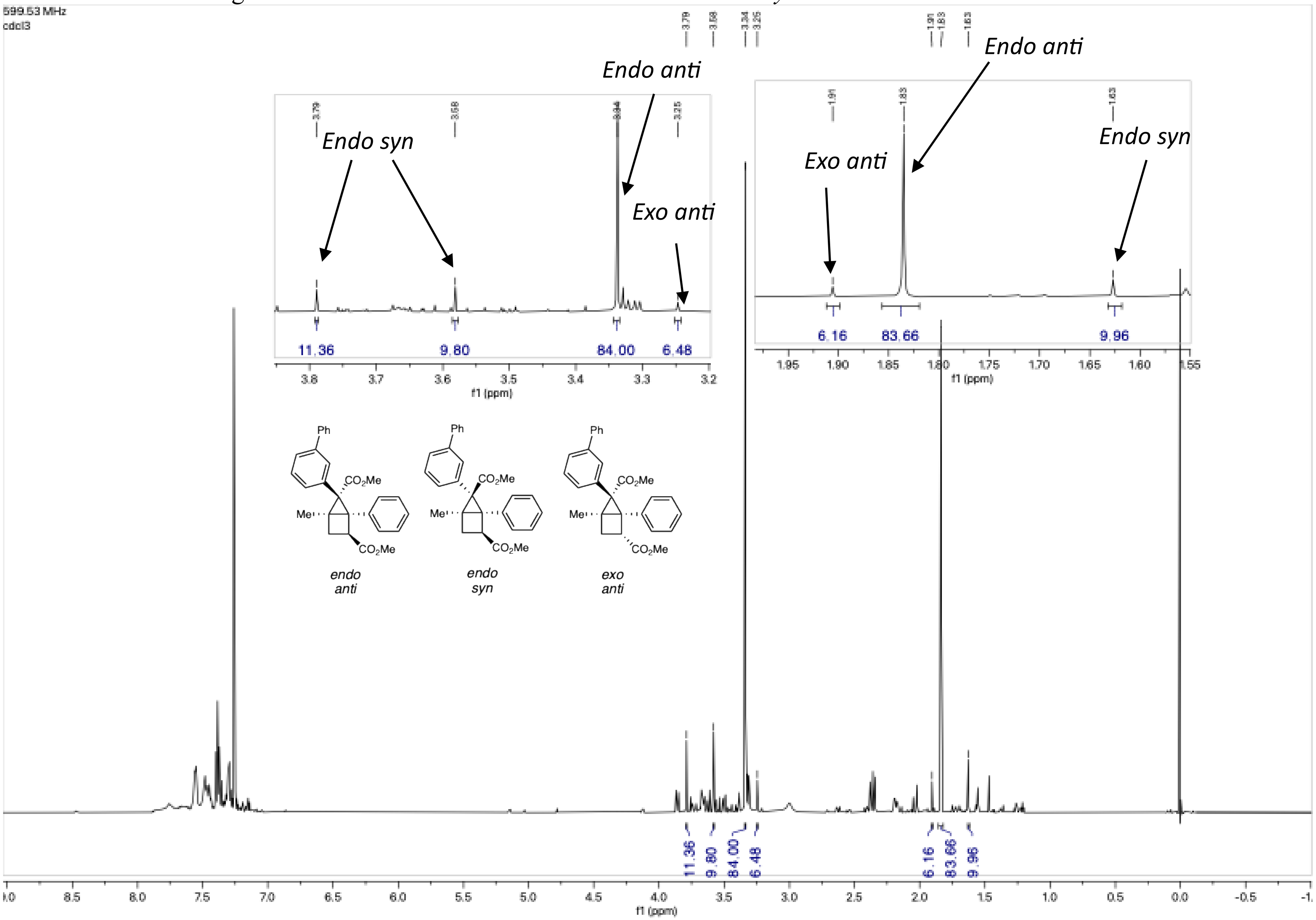

Purified yield of **21c** as a mixture of diastereomers *endo anti:exo anti* **>95:5 d.r.**

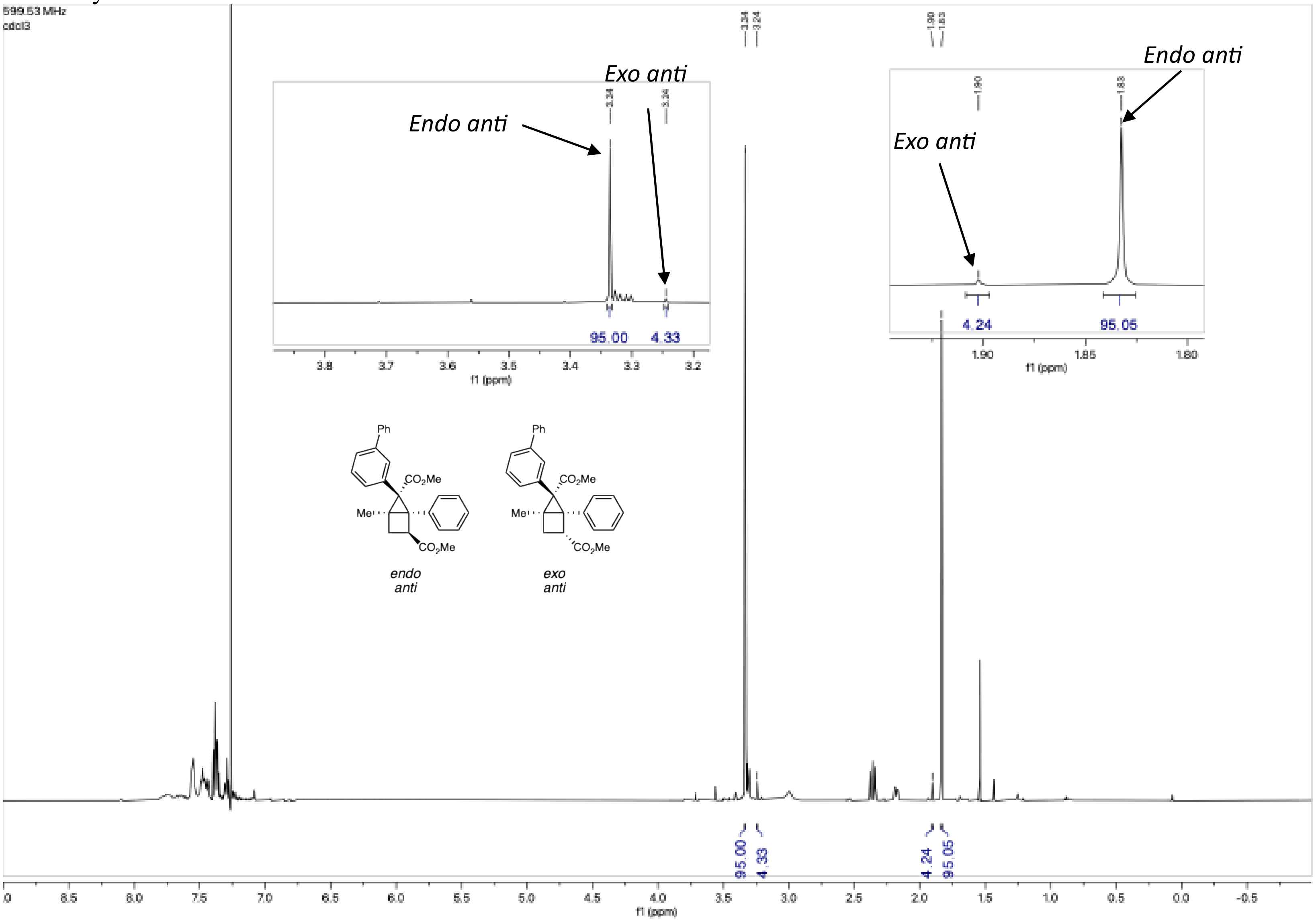

Crude reaction forming **21d** as a mixture of diastereomers *endo anti:endo syn:exo anti* **84:10:6 d.r.**

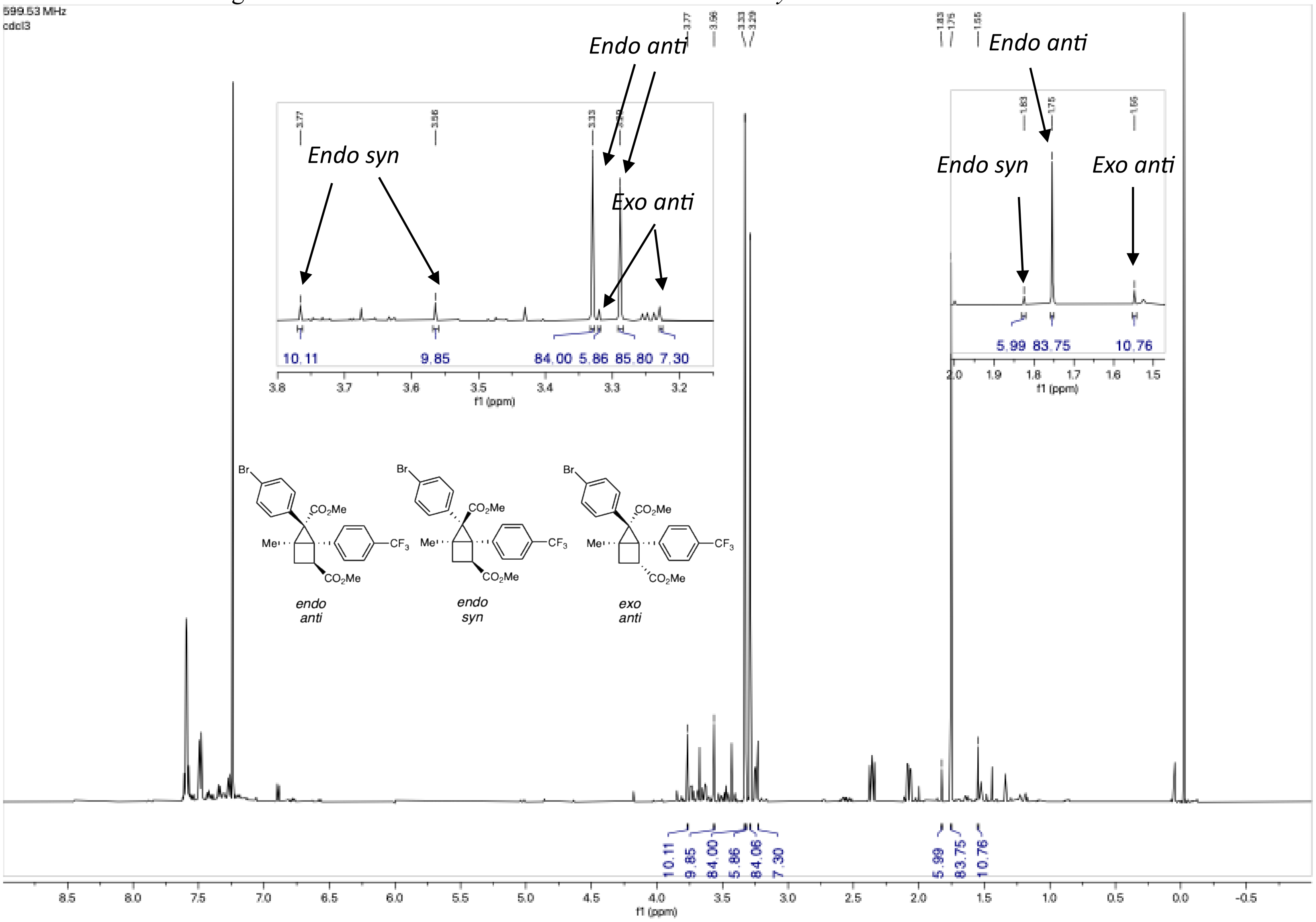

Purified yield of **21d** as a mixture of diastereomers *endo anti:exo anti* **>95:5 d.r.**

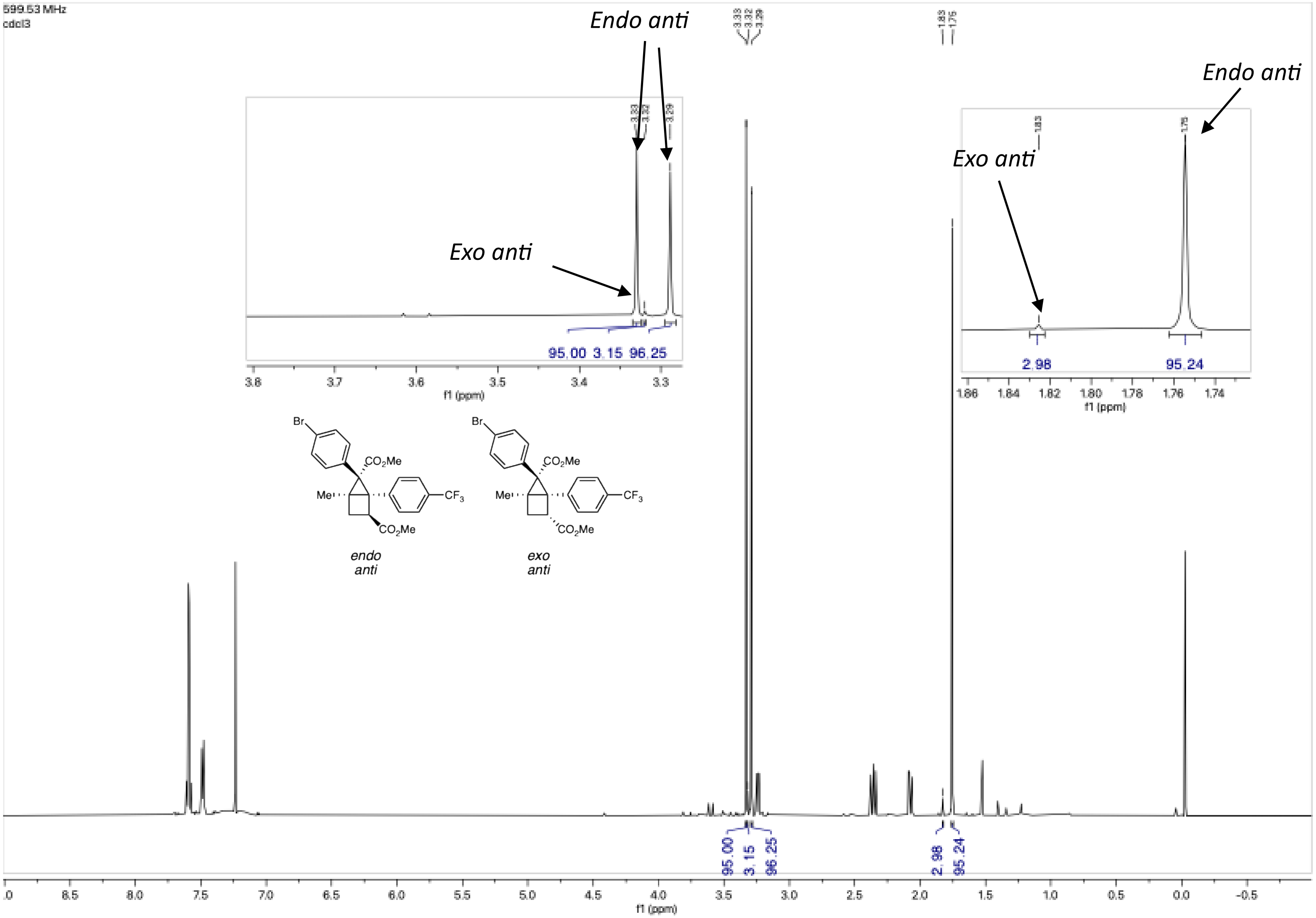

Crude reaction forming **21e** as a mixture of diastereomers *endo anti:endo syn:exo anti* **86:8:6 d.r.**

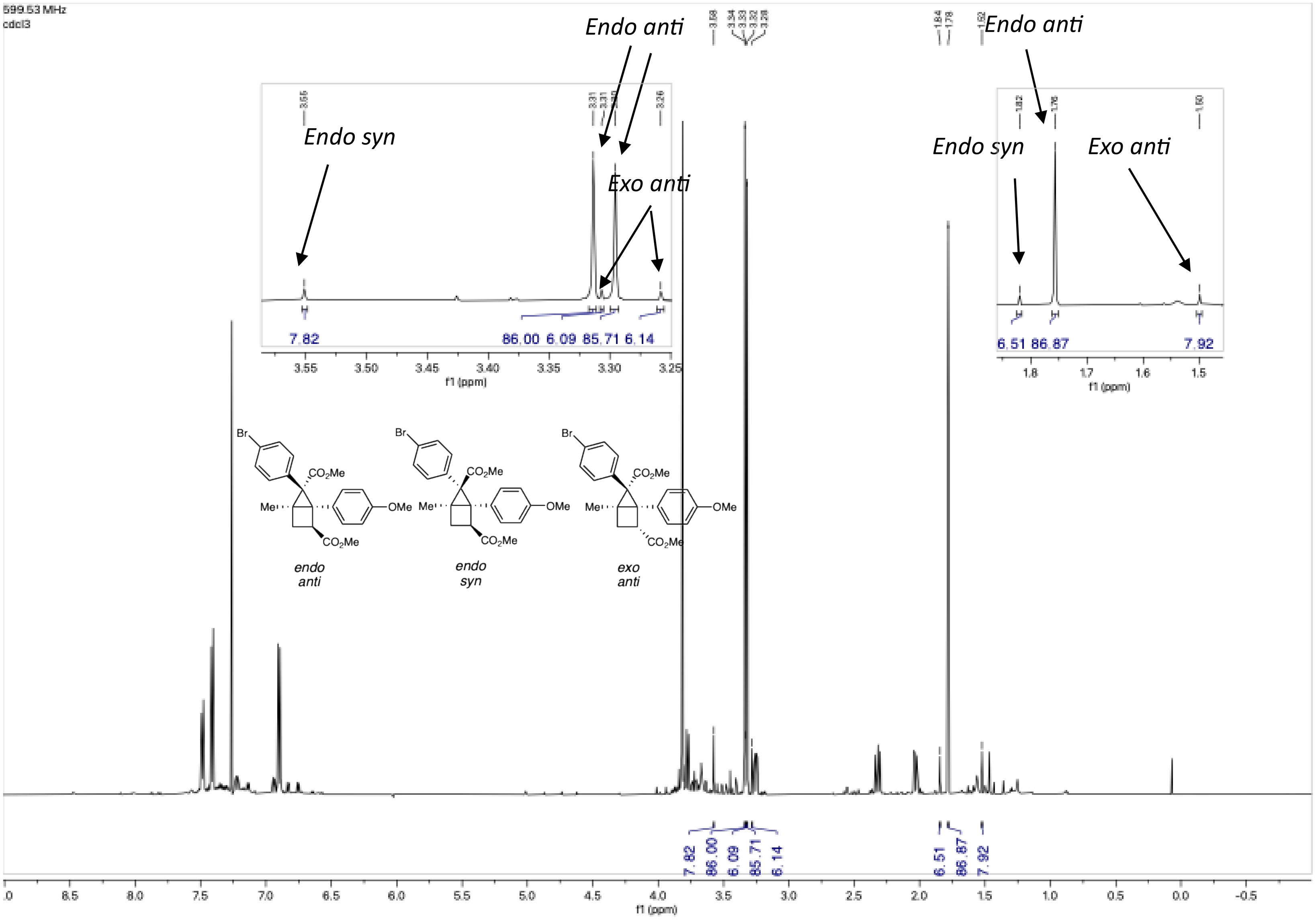

Purified yield of **21e** as a mixture of diastereomers *endo anti:exo anti* **>95:5 d.r.**

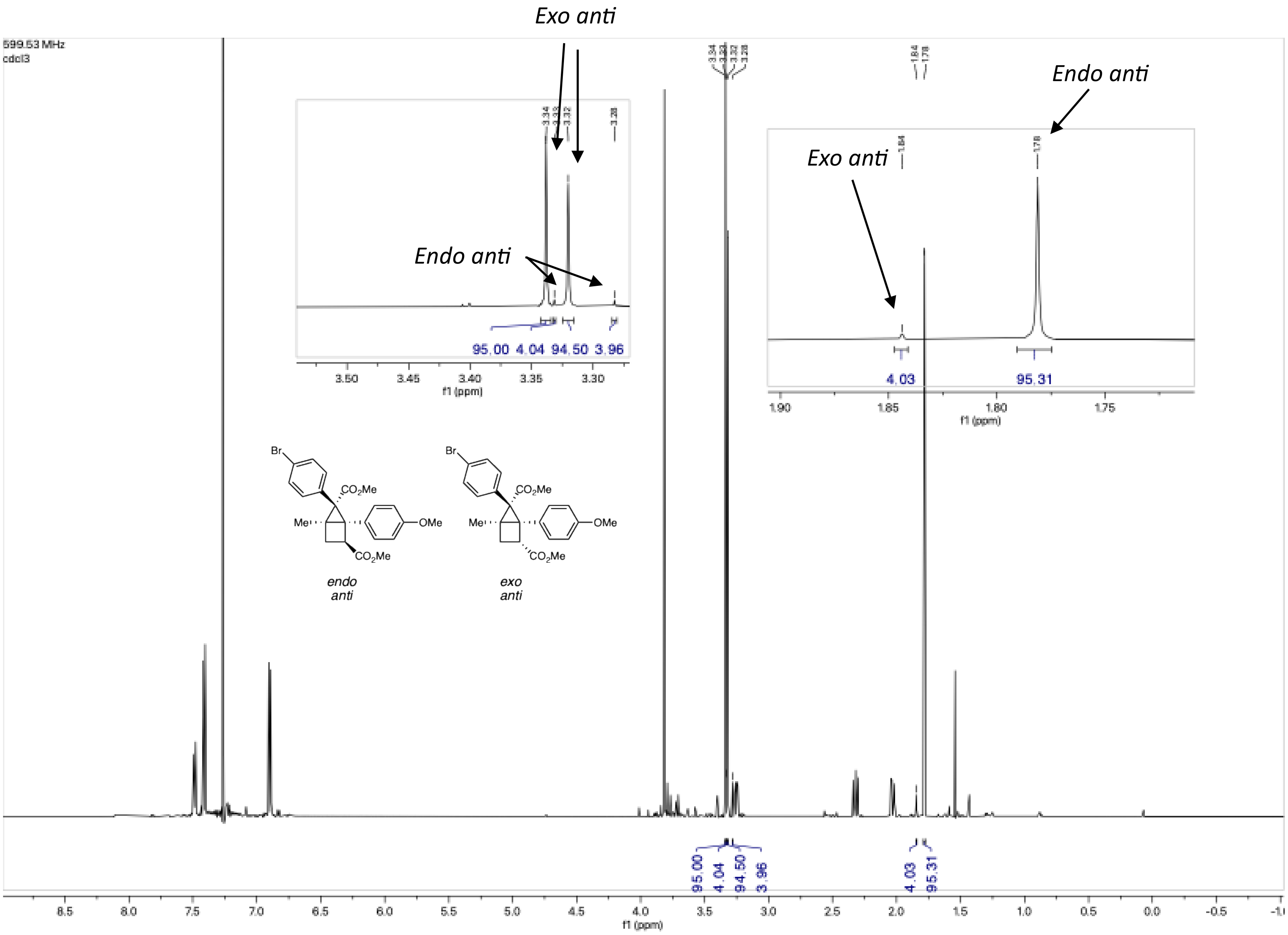

Crude reaction forming **21f** as a mixture of diastereomers *endo anti:endo syn:exo anti* **87:10:3 d.r.** (85 ºC)

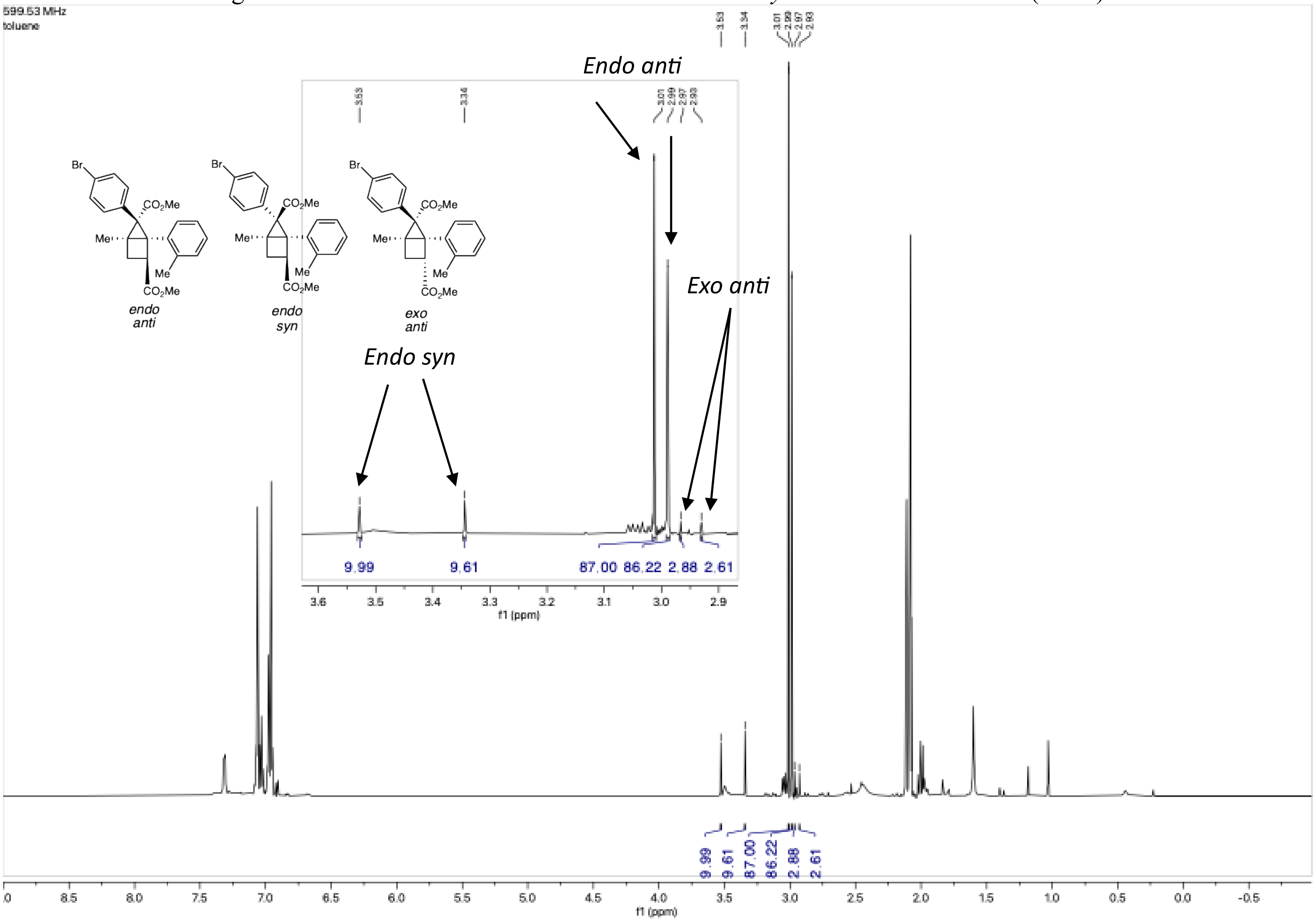

Purified yield of **21f** as a mixture of diastereomers *endo anti:exo anti* >**95:5 d.r.** (85 ºC)

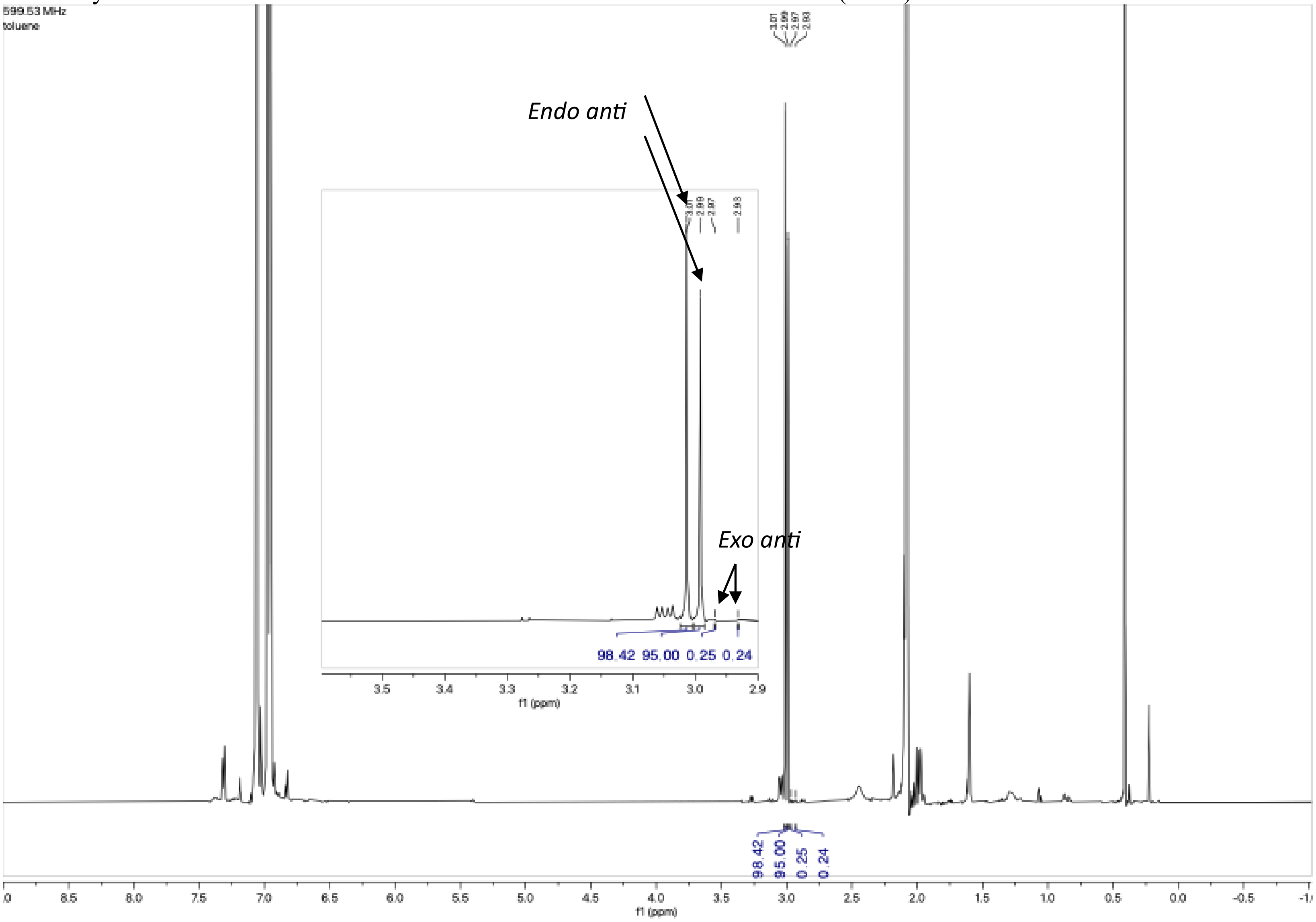

Crude reaction forming **21g** as a mixture of diastereomers *endo anti:endo syn:exo anti* **86:8:6 d.r.**

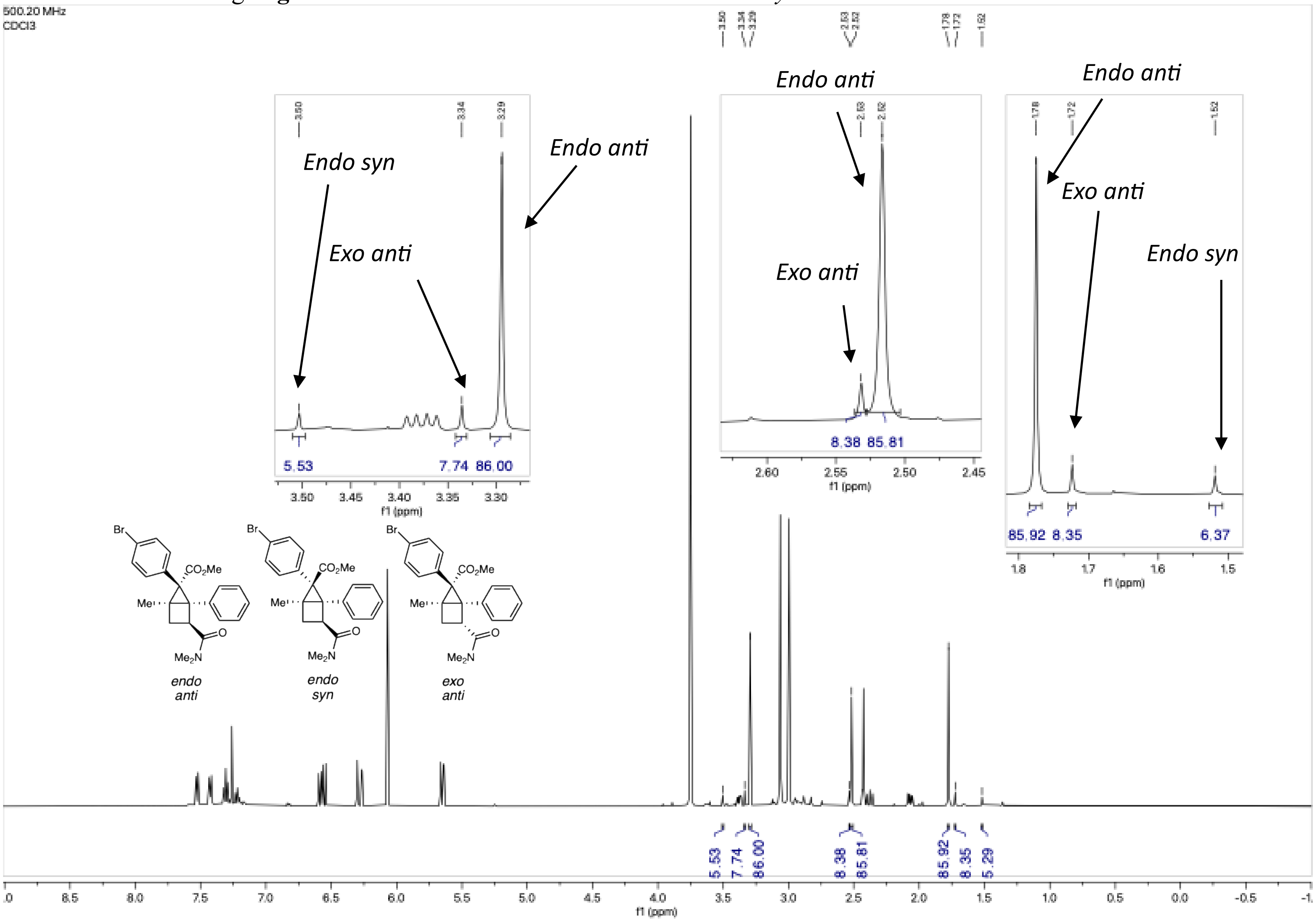

Purified yield of **21g** as a mixture of diastereomers *endo anti:exo anti* **91:9 d.r.**

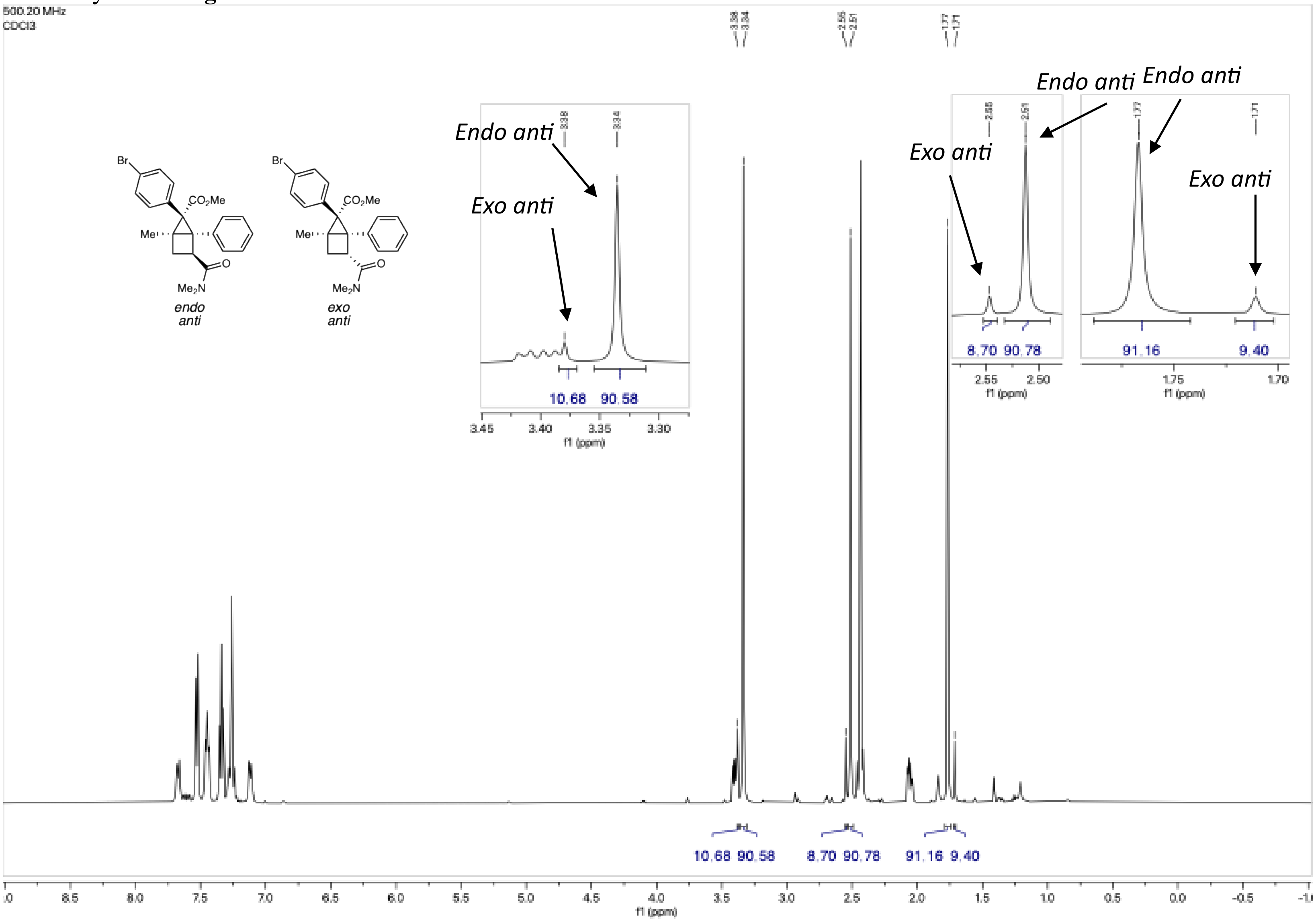

Crude reaction forming **21h** as a mixture of diastereomers *endo anti:endo syn:exo anti* **85:10:5 d.r.**

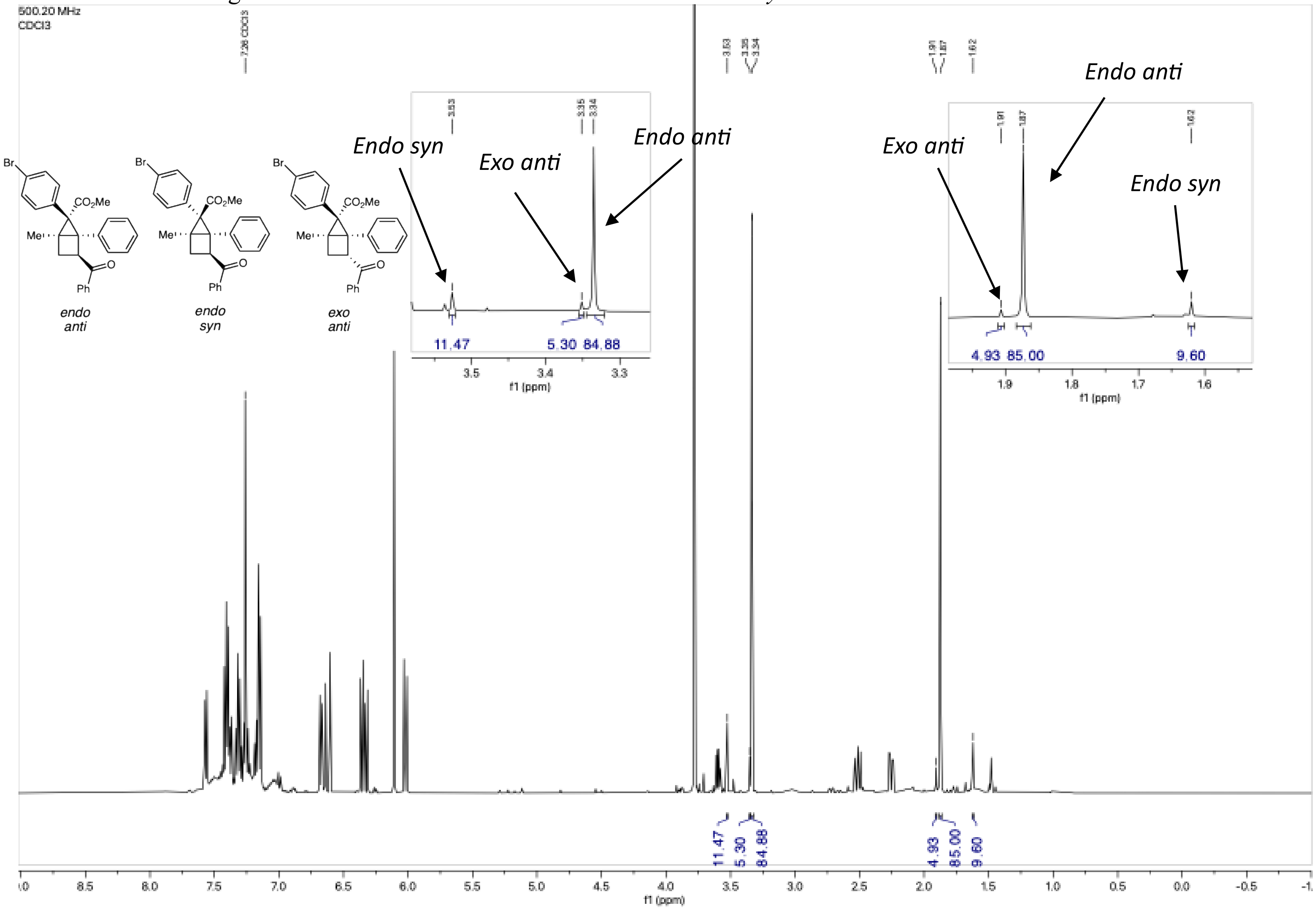

Purified yield of **21h** as a mixture of diastereomers *endo anti:endo syn:exo anti* **88:7:5 d.r.**

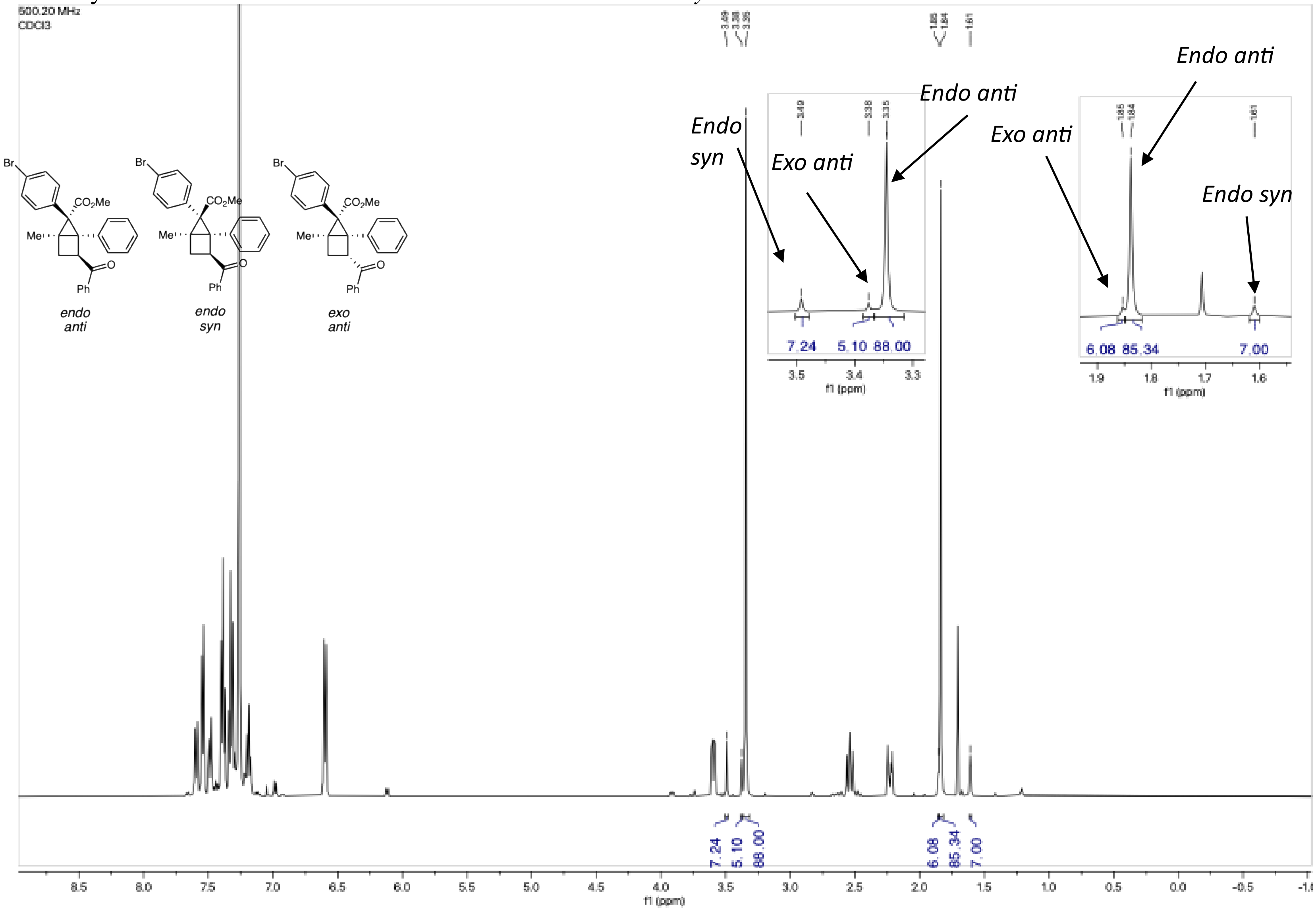

Crude reaction forming **21i** as a mixture of diastereomers *endo anti:endo syn:exo anti* **91:4:5 d.r.**

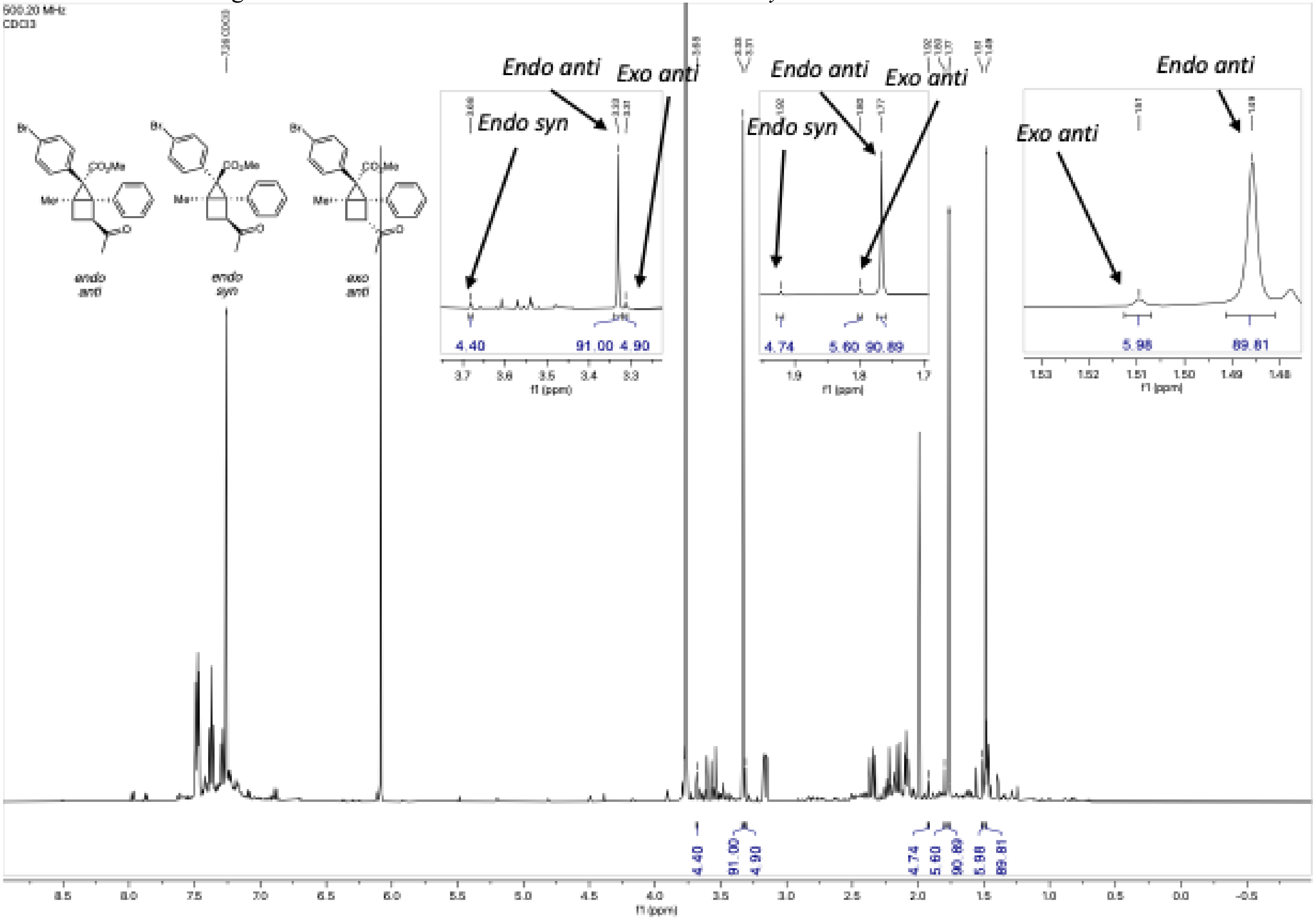

Purified yield of **21i** as a mixture of diastereomers *endo anti:endo syn* **>95:5 d.r.**

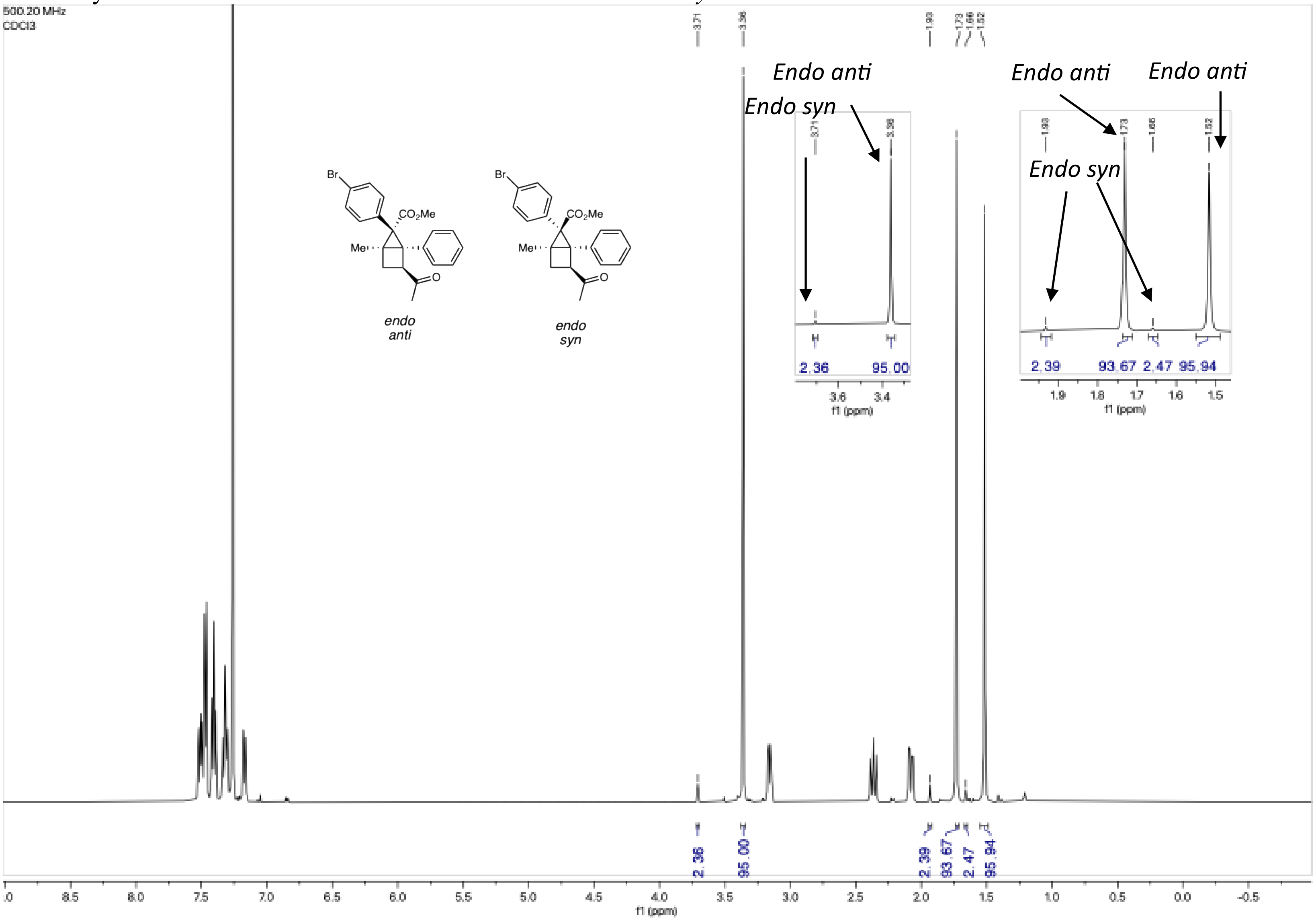

Crude reaction forming **21j** as a mixture of diastereomers *endo anti:endo syn:exo anti* **62:5:33 d.r.**

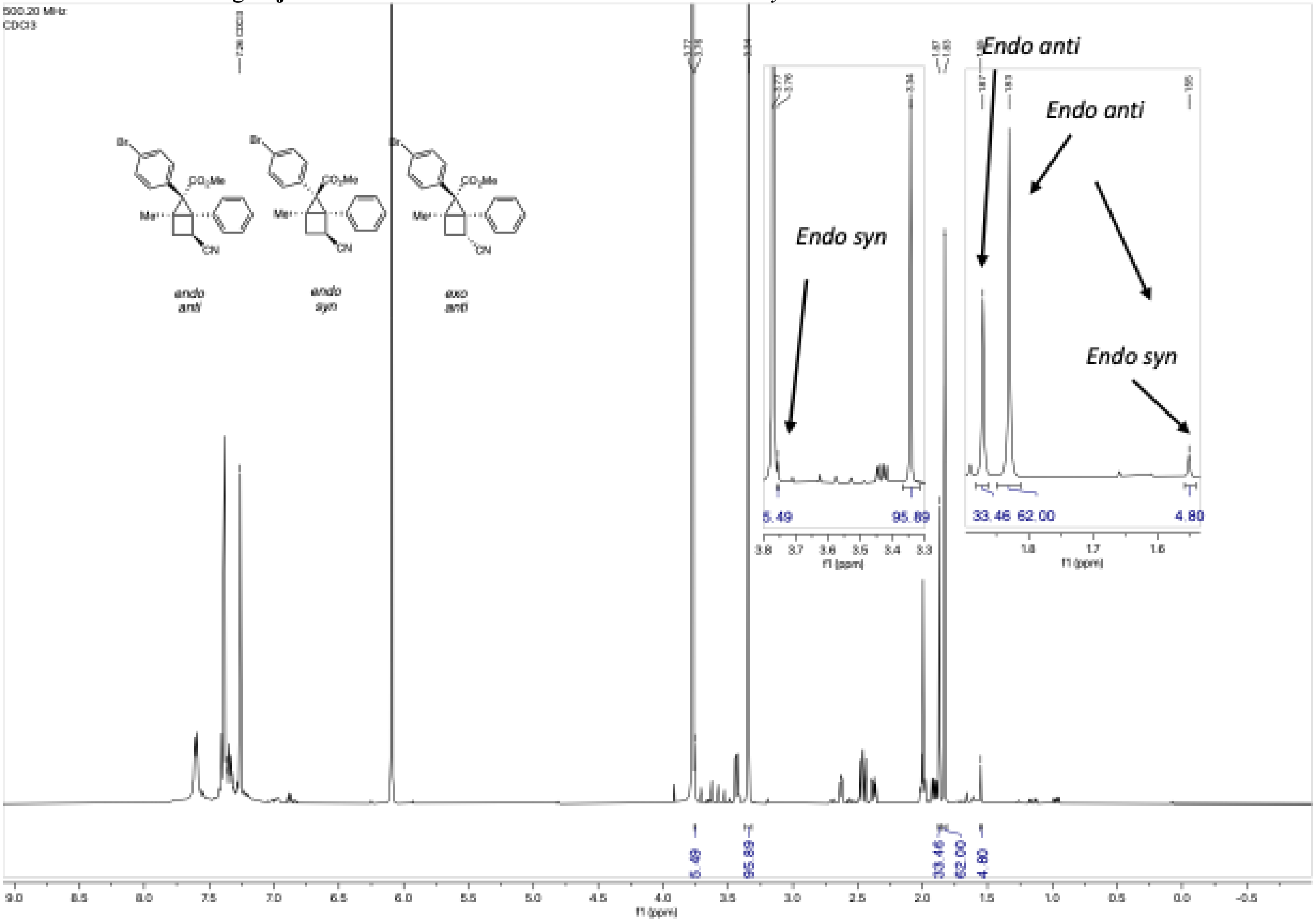

Purified yield of **21j** major diastereomer *endo anti:exo anti* >**95:5 d.r.**

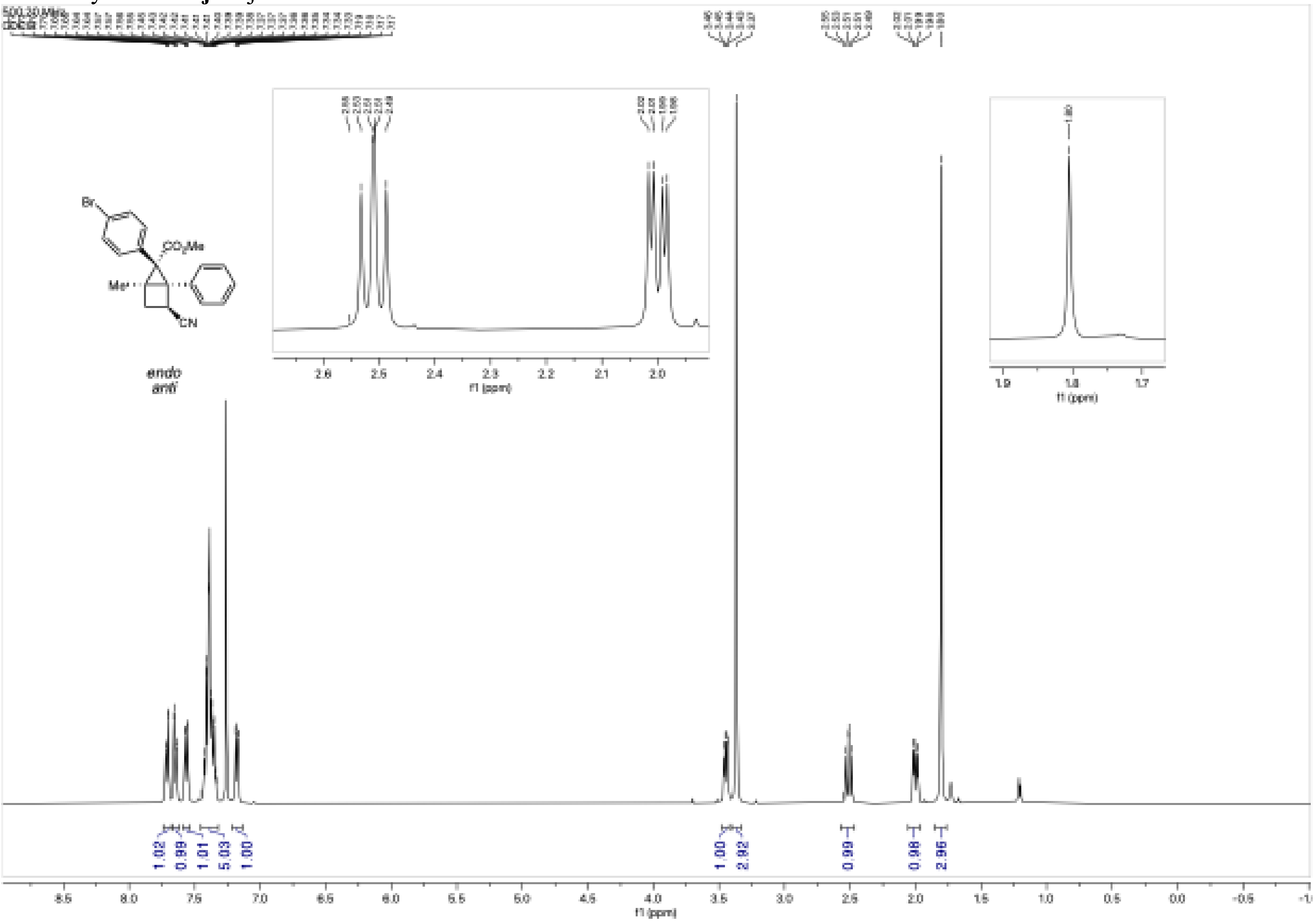

Purified yield of **21j** minor diastereomer *exo anti*:*endo anti* >**95:5 d.r.**

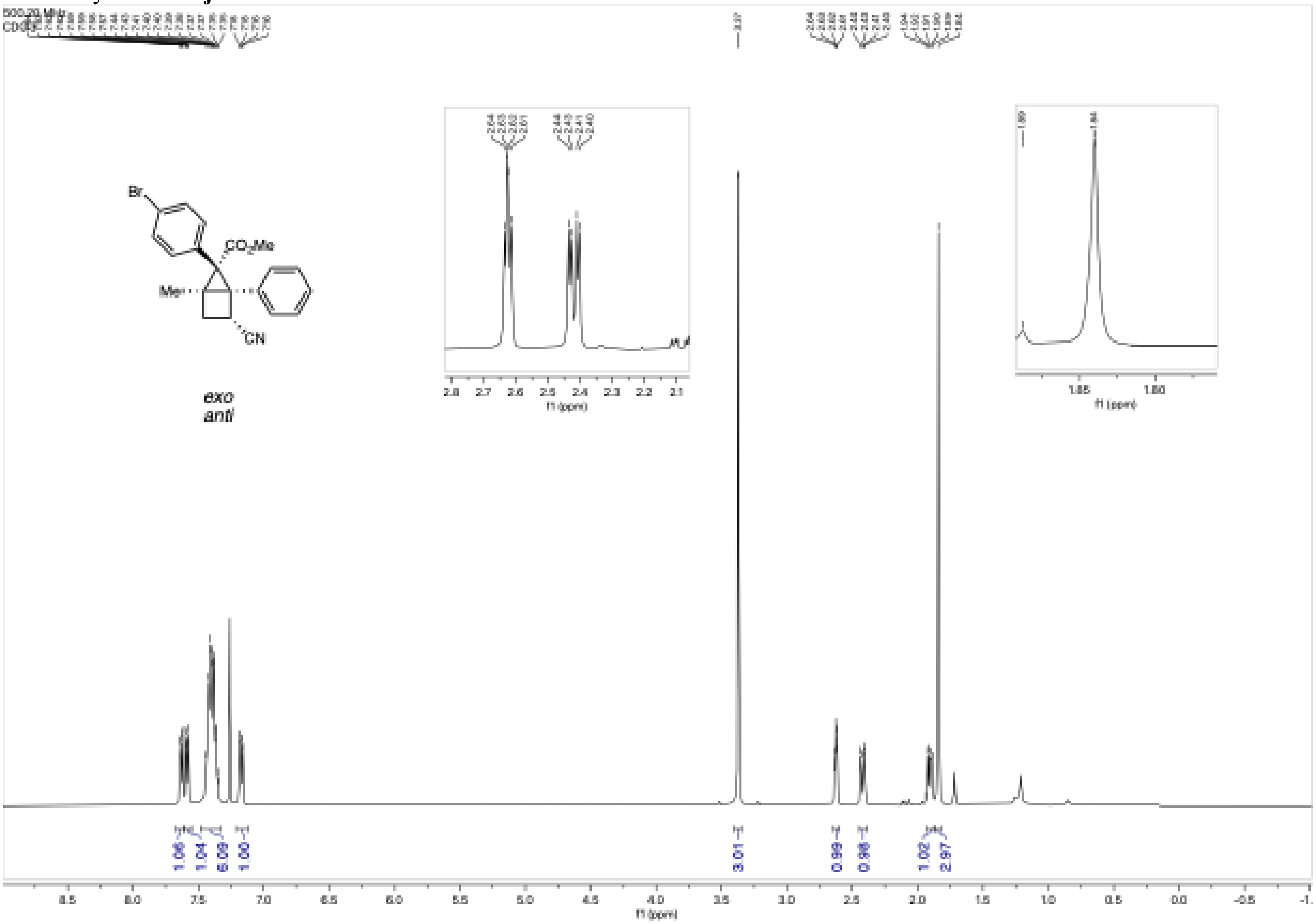

Crude reaction in CD_3CN forming **21k** as a mixture of diastereomers *endo anti:endo syn:exo anti* **85:11:4 d.r.**

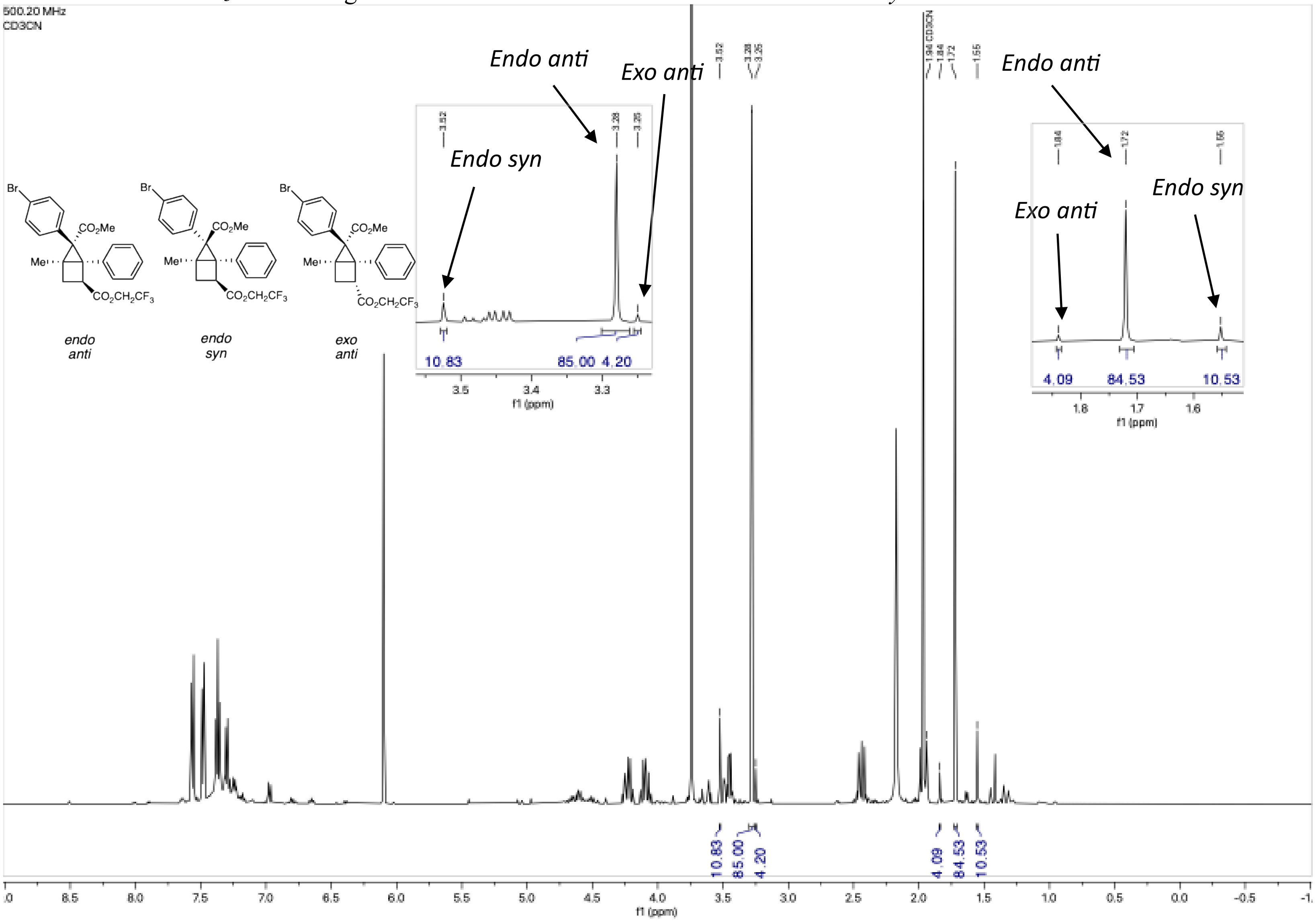

Purified yield of **21k** as a mixture of diastereomers *endo anti:endo syn* **92:8 d.r.**

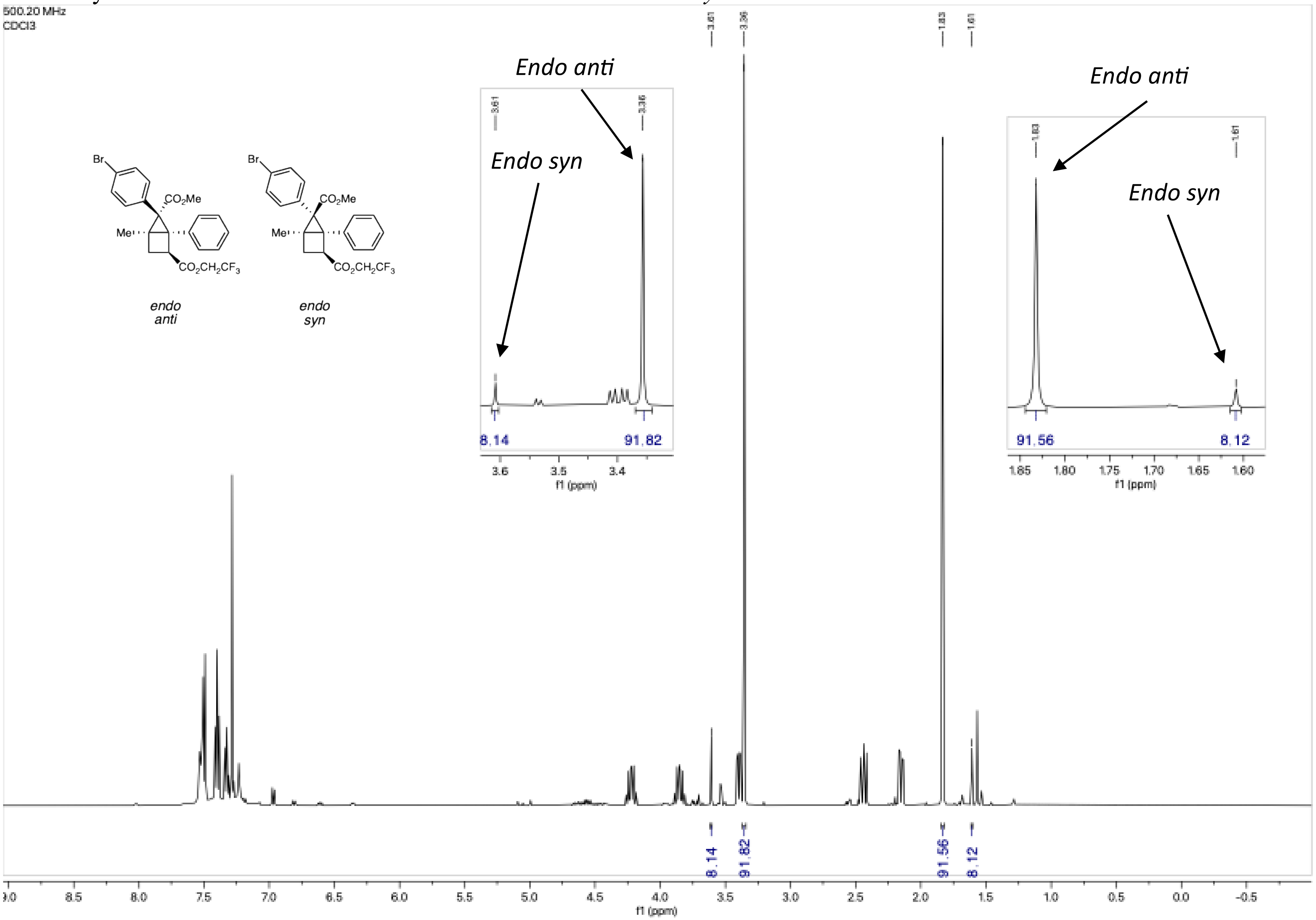

Only Observed Diastereomer of **21l** (Purified)

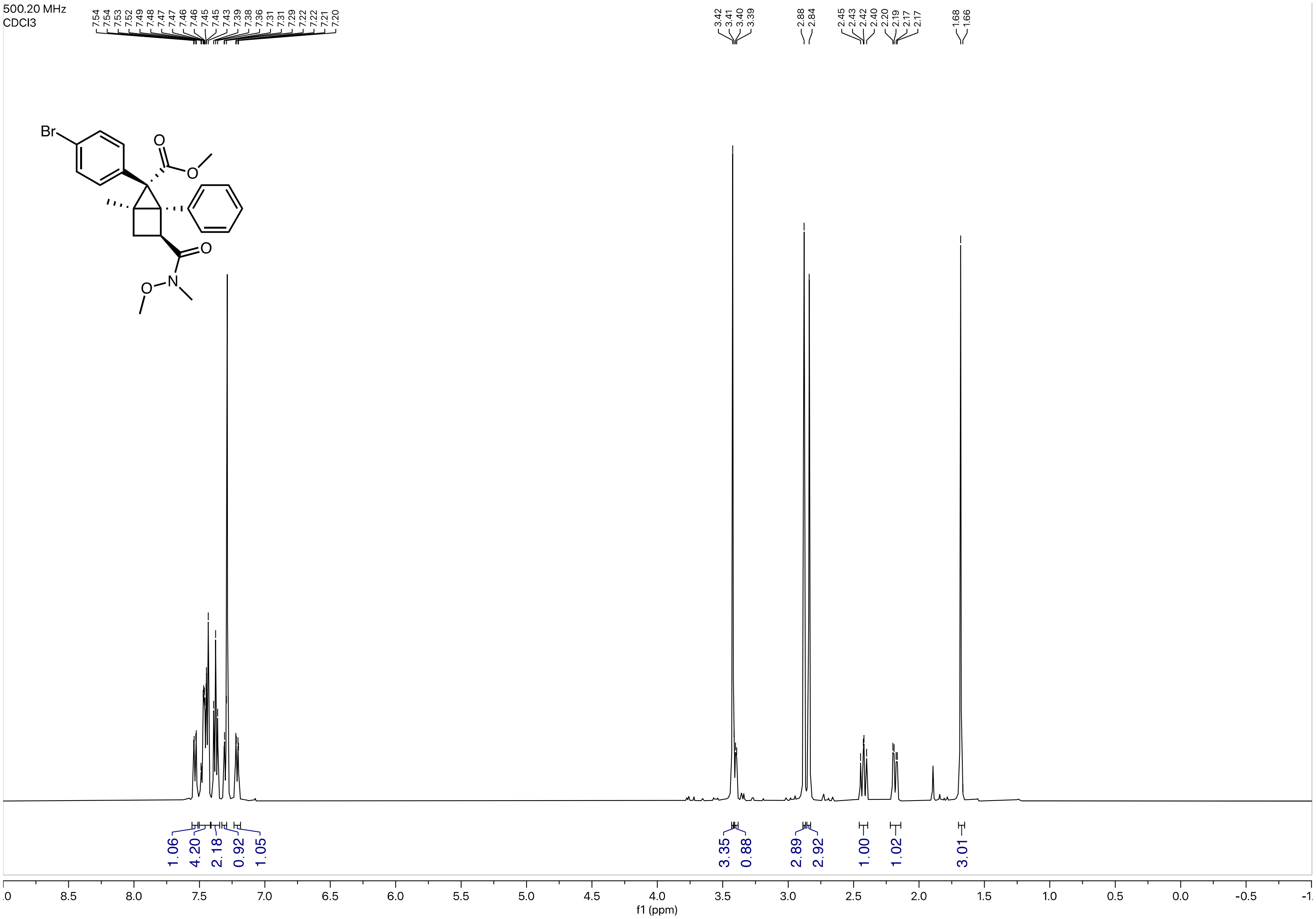
500.20 MHz
CDCl3
7.54
7.54
7.53
7.52
7.49
7.48
7.47
7.47
7.46
7.46
7.45
7.45
7.43
7.39
7.38
7.36
7.31
7.31
7.29
7.22
7.22
7.21
7.20
3.42
3.41
3.40
3.39
2.88
2.84
2.45
2.43
2.42
2.40
2.20
2.19
2.17
2.17
1.68
1.66
Br
O
O
O
N
1.06
4.20
2.18
0.92
1.05
3.35
0.88
2.89
2.92
1.00
1.02
3.01
f1 (ppm)

Crude reaction forming **23a** as a mixture of diastereomers *endo anti:endo syn:exo anti* **90:6:4 d.r.**

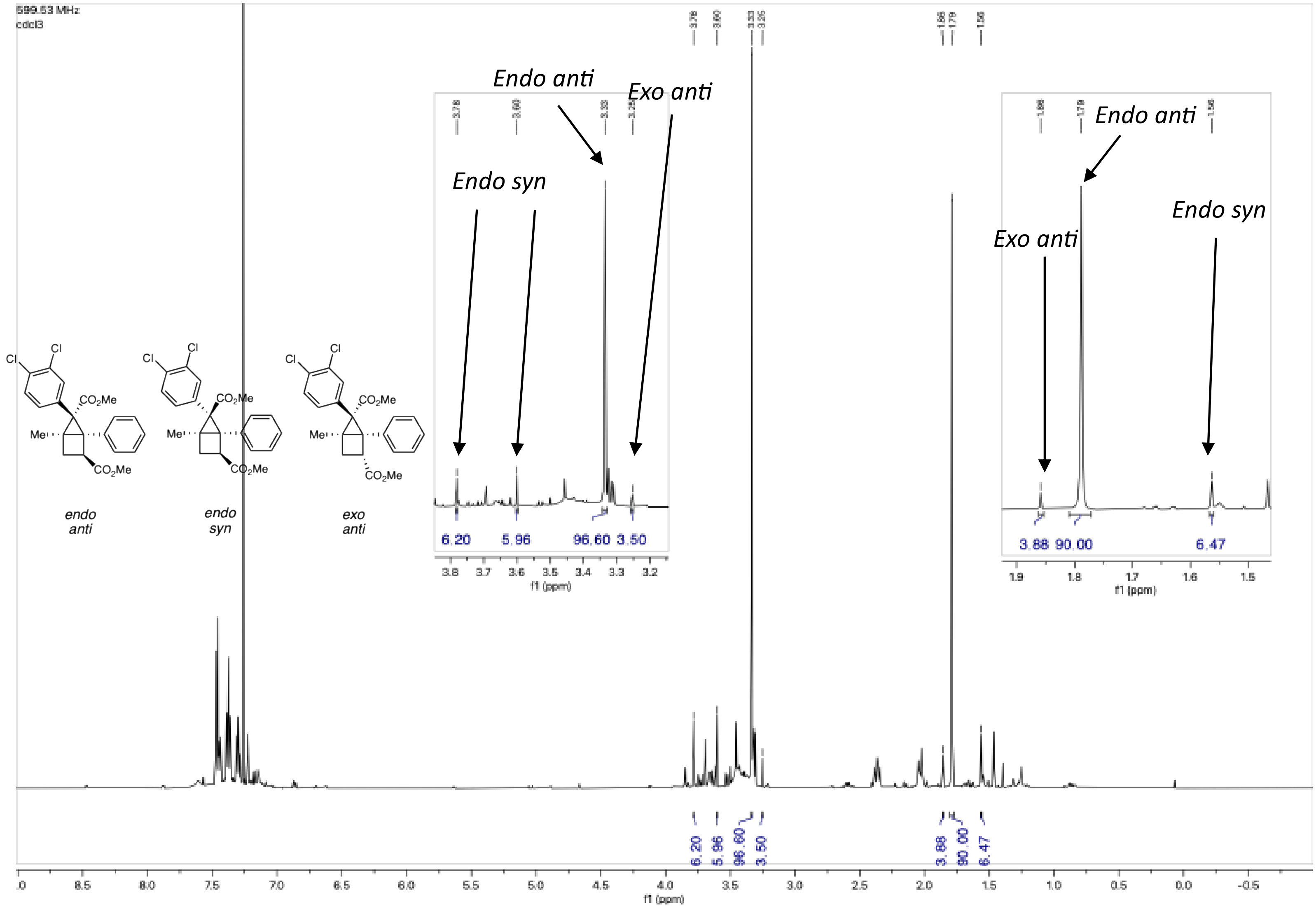

Purified yield of **23a** as a mixture of diastereomers *endo anti:exo anti* **>95:5 d.r.**

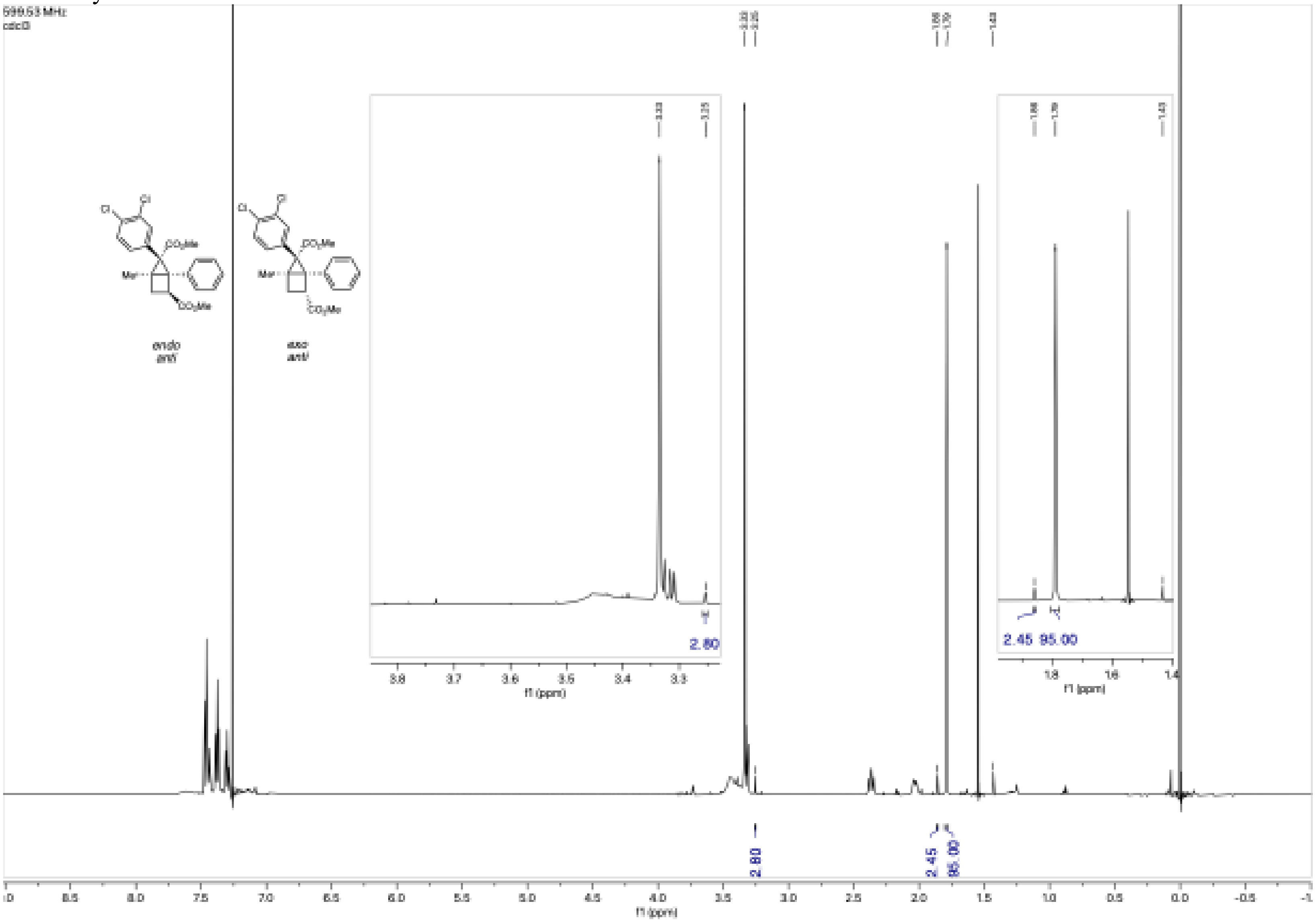

Crude reaction forming **23b** as a mixture of diastereomers *endo anti:endo syn:exo anti* **76:14:10 d.r.**

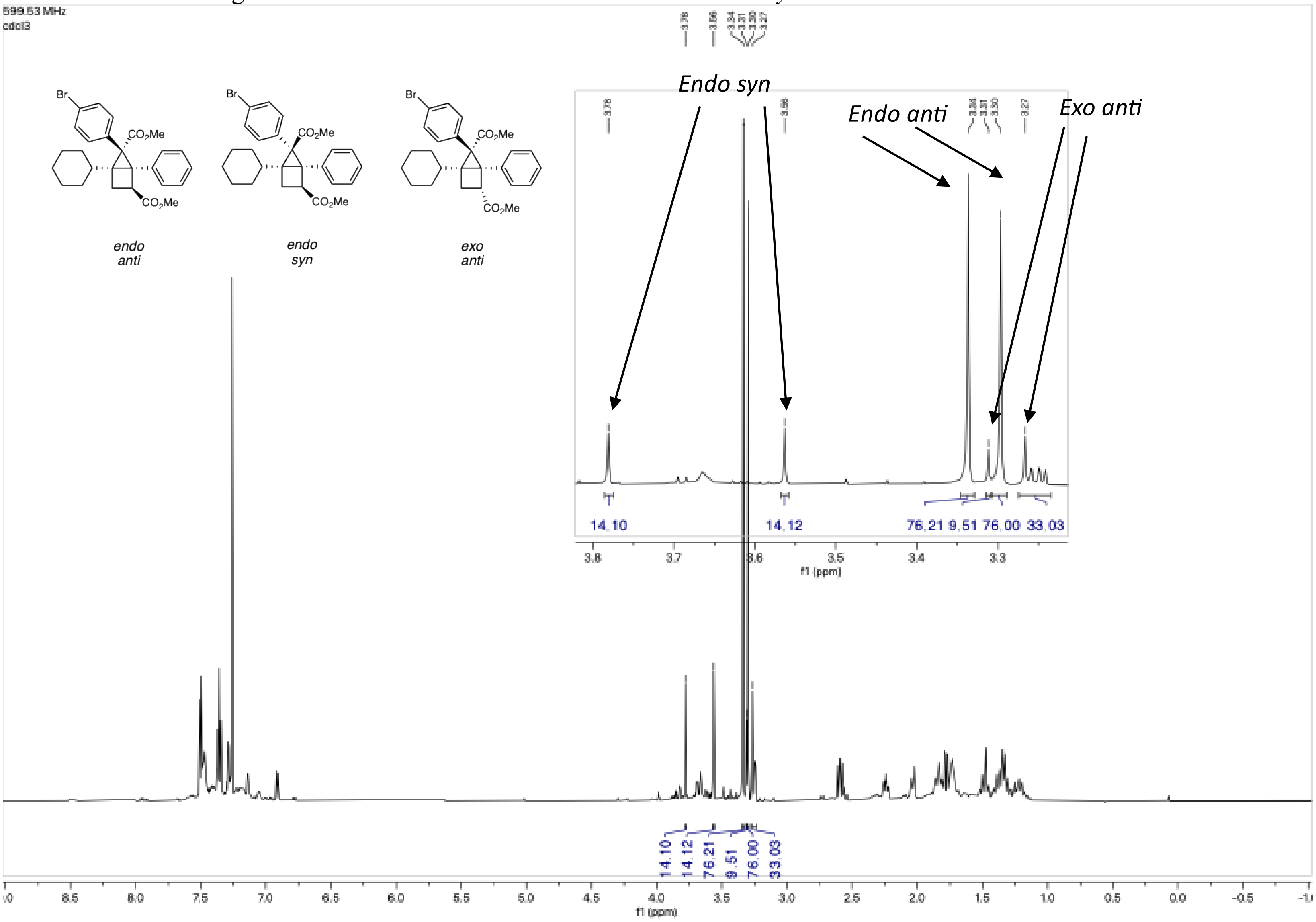

Purified yield of **23b** as a mixture of diastereomers *endo anti:exo anti* **93:7 d.r.**

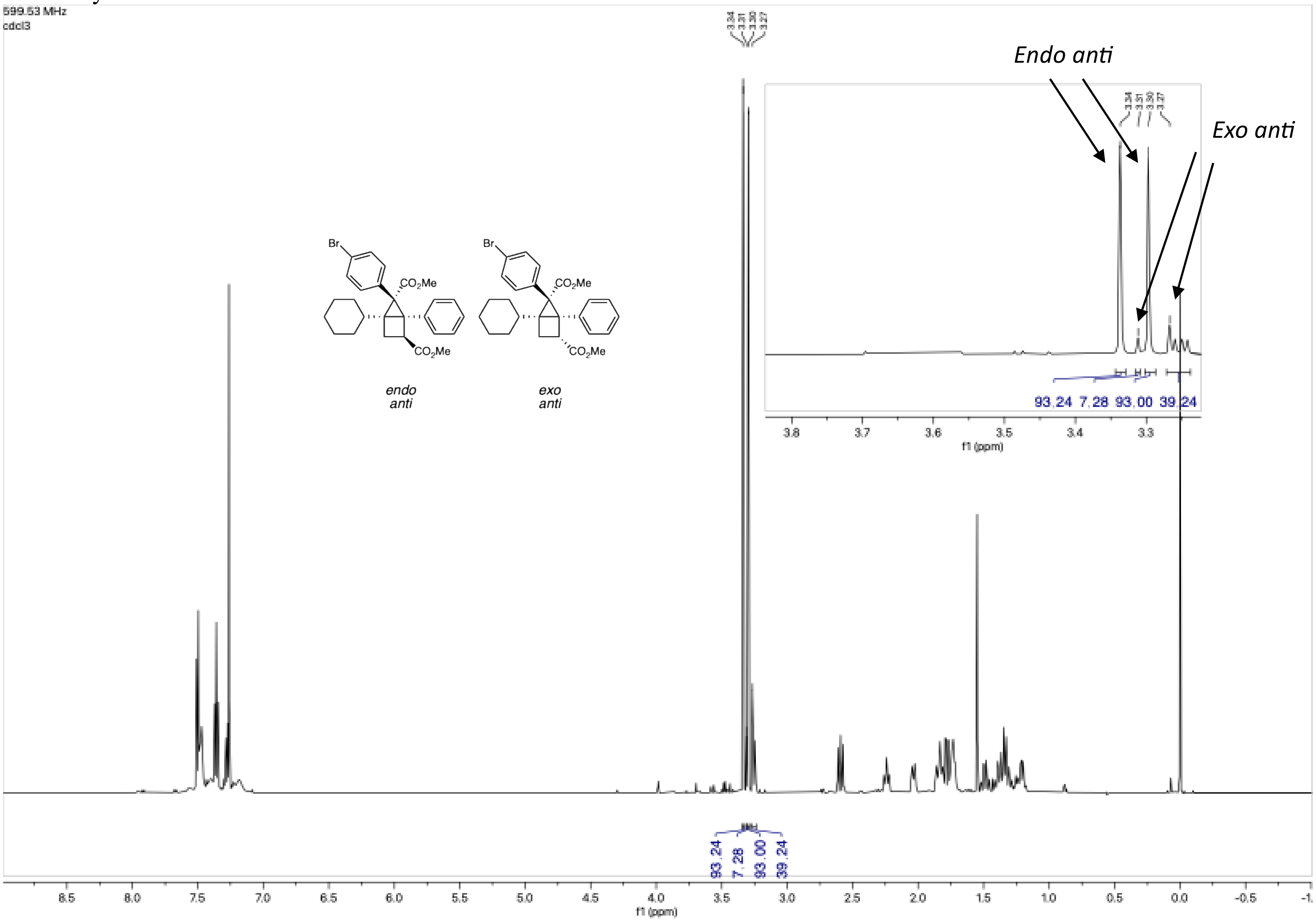

Crude reaction forming **23c** as a mixture of diastereomers *endo anti:endo syn:exo anti* **85:8:7 d.r.**

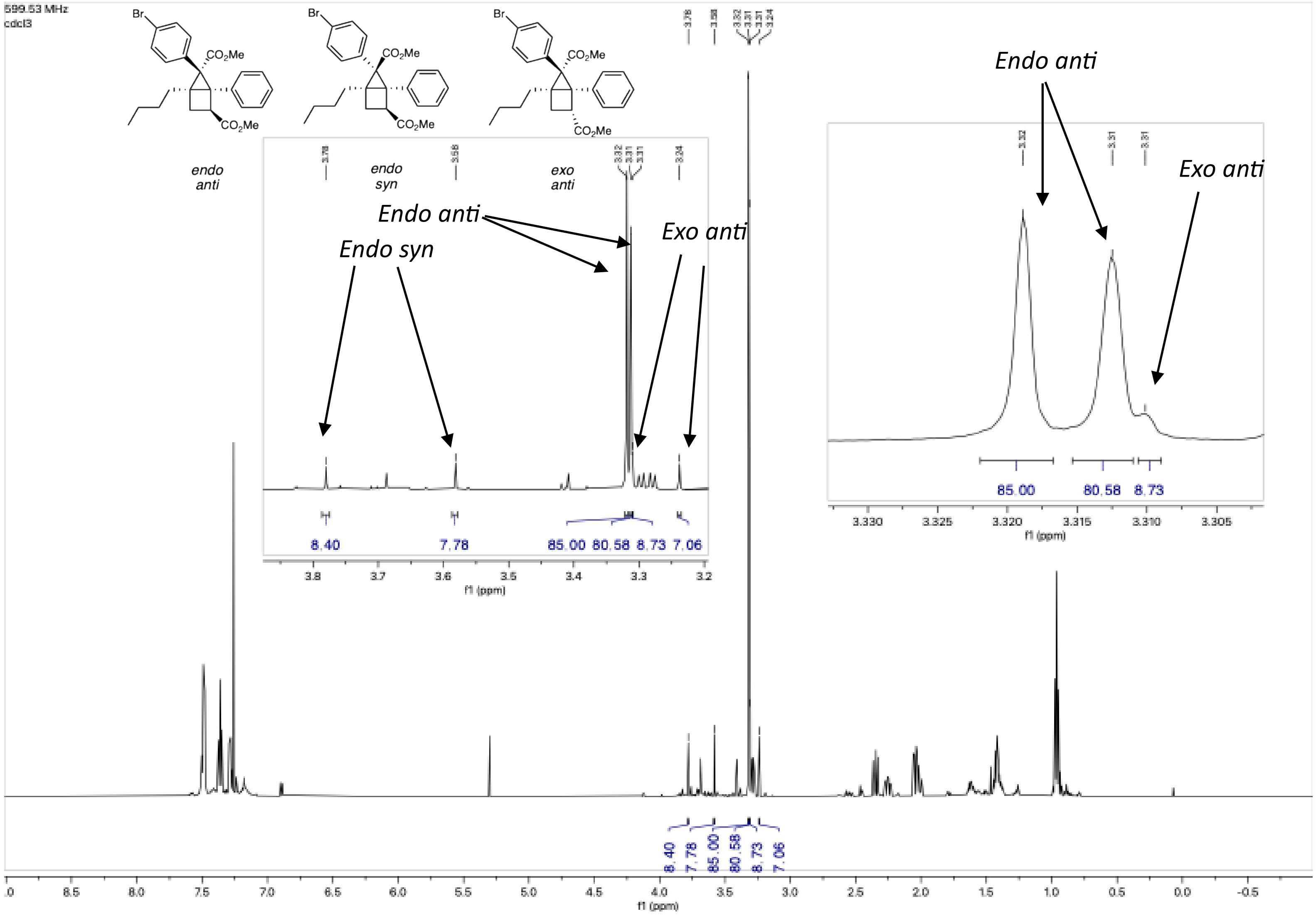

Purified yield of **23c** as a mixture of diastereomers *endo anti:exo anti* **>95:5 d.r.**

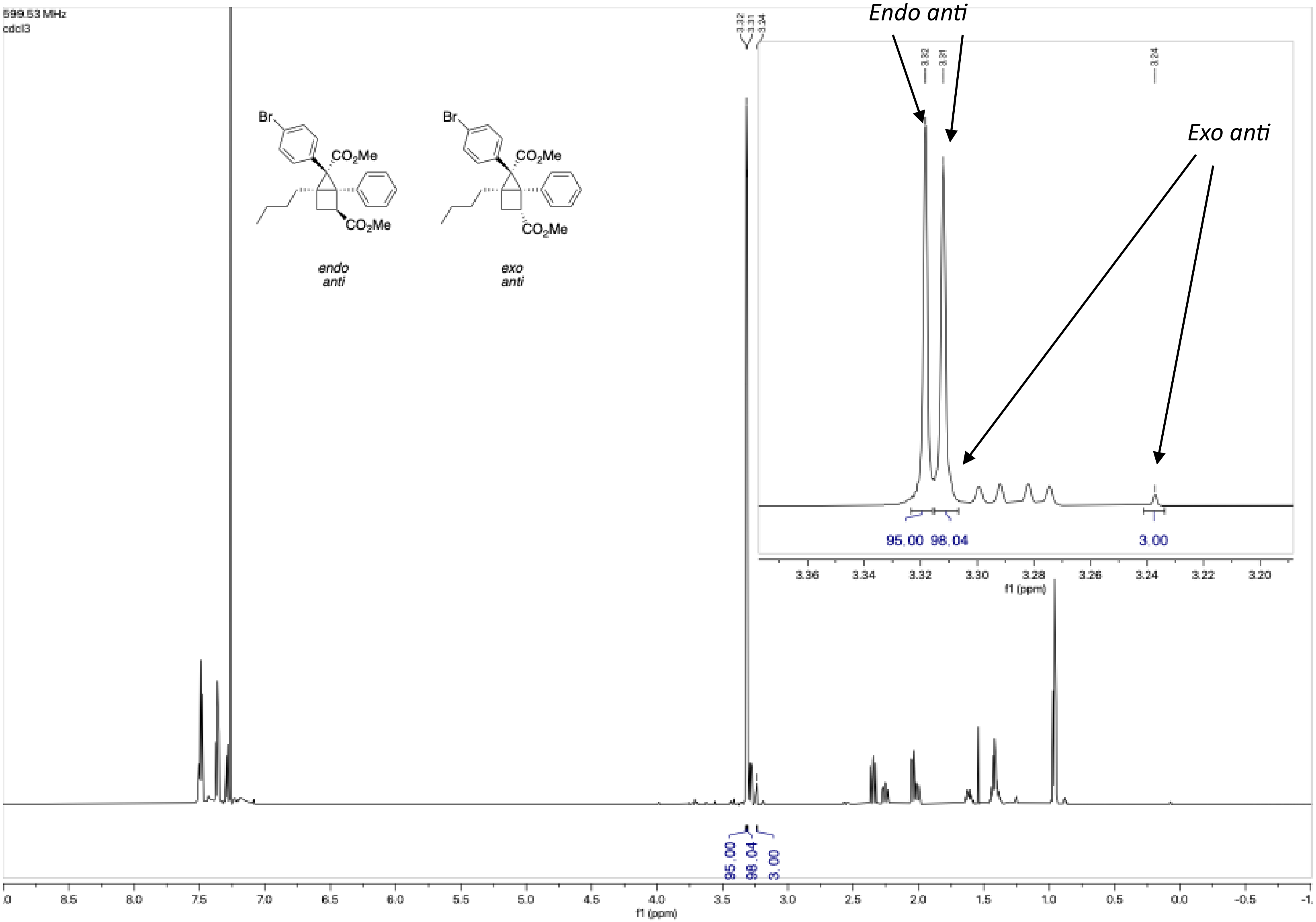

Purified yield of **23d** as a mixture of diastereomers *endo anti:exo anti* **90:10 d.r.** (-25 ºC)

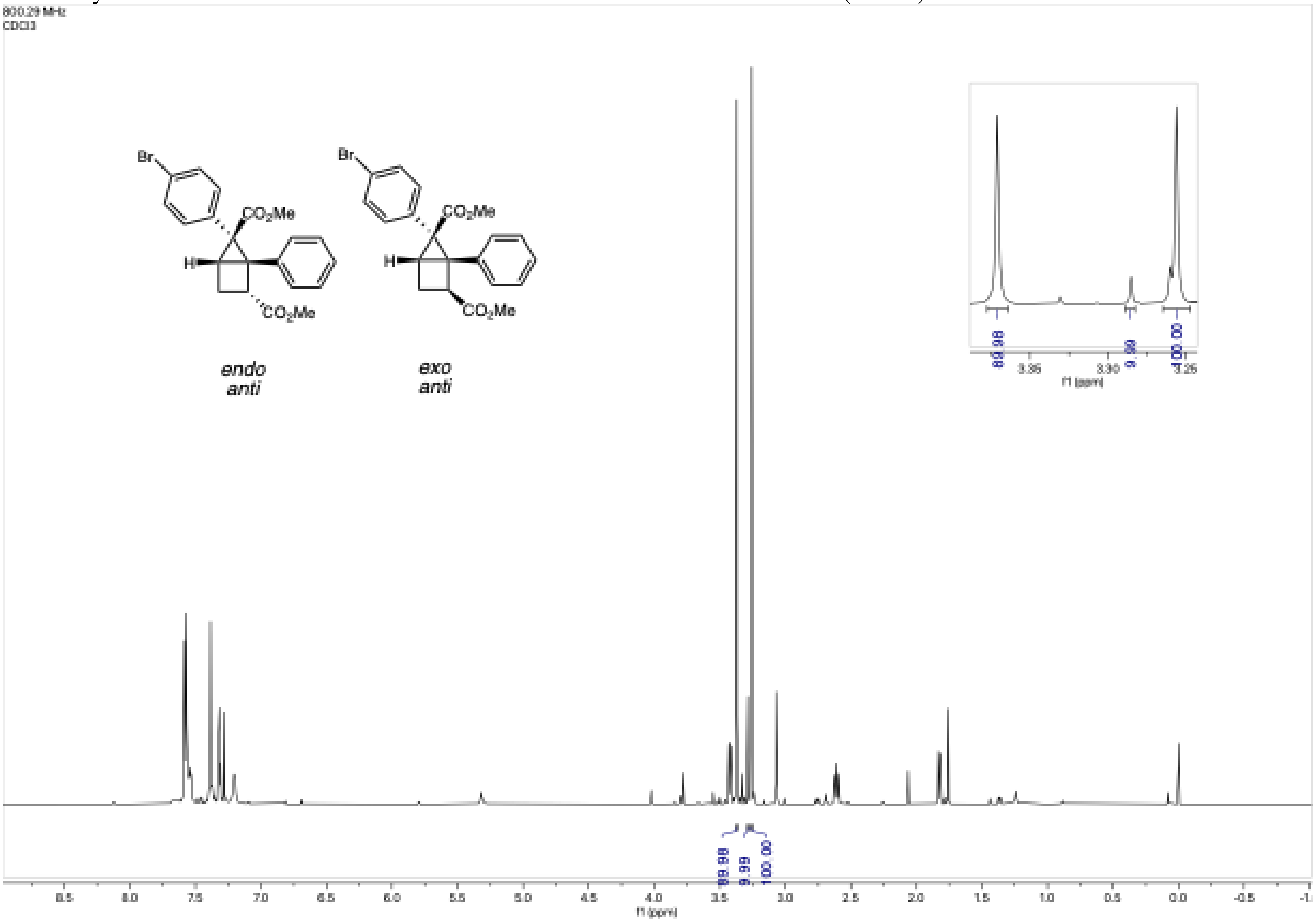

Purified yield of **23d** as a mixture of diastereomers *endo anti:exo anti* **integration of major diastereomer** (-25 ºC)

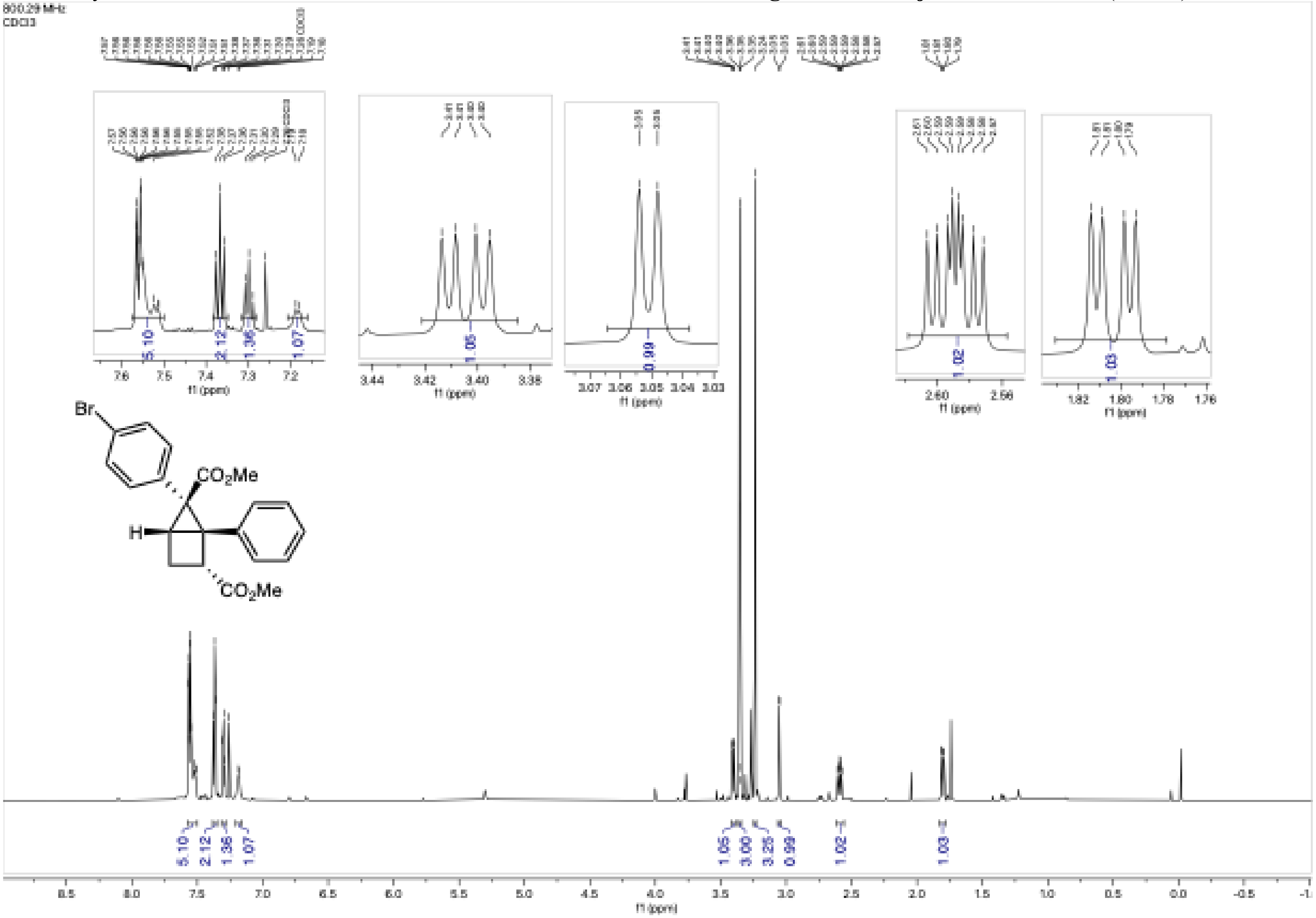

Purified yield of **23d** as a mixture of diastereomers *endo anti:exo anti* **integration of minor diastereomer** (-25 ºC)

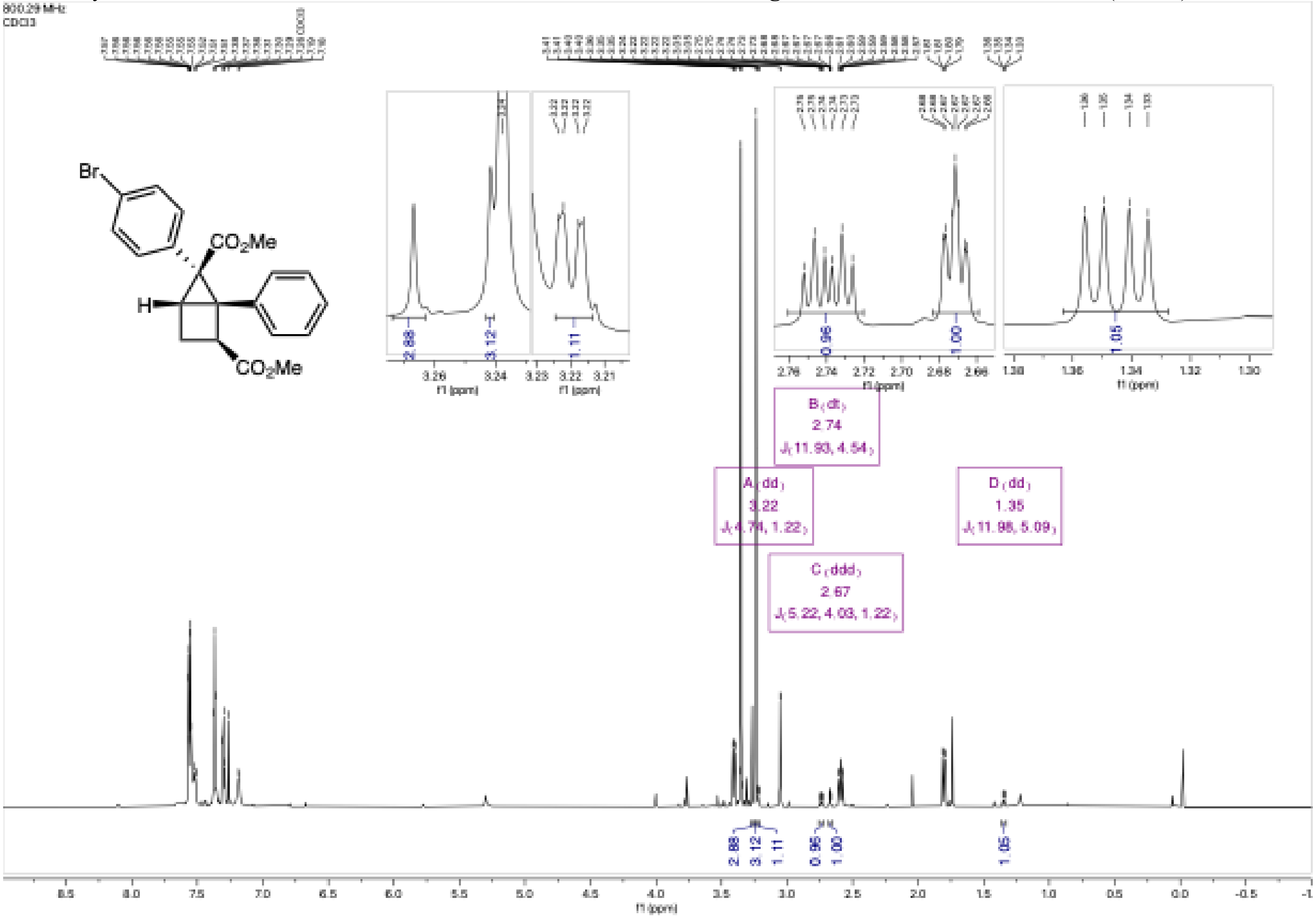

VI. Failed Reactions

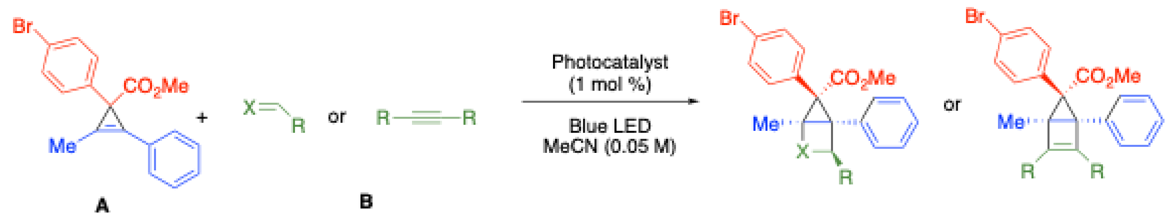

Entry	B	Equiv B	Time	Temp.	Yield (NMR)	d.r.
1.	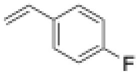	5	26 h	-40 °C	17%	>20:1
2.	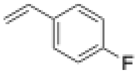	5	16 h	-20 °C	21%	>20:1
3.	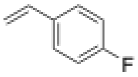	20	22 h	-20 °C	19%	>20:1
4.	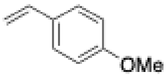	5	14 h	-20 °C	<20%	n.d.
5.	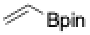	5	1.5 h	-20 °C	25%	12.3:3.3:1
6.	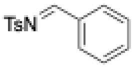	3	1.5 h	-20 °C	Trace	n.d.
7.	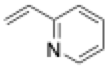	5	98.5 h	-40 °C	30%	3.3:1.3:1:1[a]
8.	$MeO_2C-C\equiv C-CO_2Me$	5	4 h	-20 °C	29%	1.6:1
9.	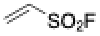	4	15 h	-20 °C	N.R.	n/a

[a] Reaction mixture may contain regioisomer, assignments could not be determined. NMR yield determined using 1,3,5-trimethoxybenzene.

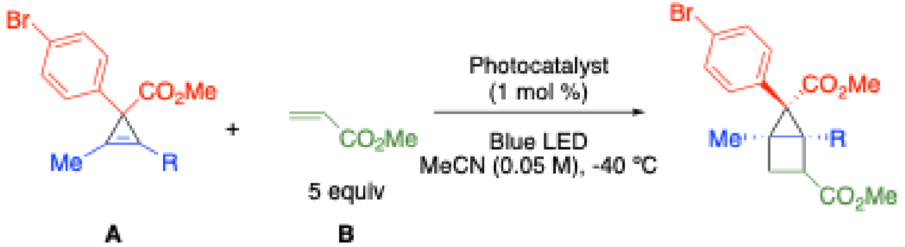

Entry	R	Time	Yield (NMR)
1.	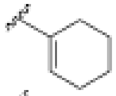	2 h	20%
2.	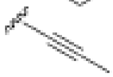	6 h	14%

Reactions were purified on silica gel in an attempt to isolate, but a clean reaction product could not be isolated. To the isolated material was added 1 equiv of 1,3,5-trimethoxybeneze to determine yield. Diastereomeric ratio could not be determined.

VII. REFERENCE

1. Muñoz, M. P.; Adrio, J.; Carretero, J. C.; Echavarren, A. M., Ligand Effects in Gold- and Platinum-Catalyzed Cyclization of Enynes: Chiral Gold Complexes for Enantioselective Alkoxycyclization. *Organometallics* **2005,** *24* (6), 1293-1300.
2. Keipour, H.; Ollevier, T., Iron-Catalyzed Carbene Insertion Reactions of α-Diazoesters into Si–H Bonds. *Org. Lett.* **2017,** *19* (21), 5736-5739.
3. Green, S. P.; Wheelhouse, K. M.; Payne, A. D.; Hallett, J. P.; Miller, P. W.; Bull, J. A., Thermal Stability and Explosive Hazard Assessment of Diazo Compounds and Diazo Transfer Reagents. *Organic Process Research & Development* **2020,** *24* (1), 67-84.
4. JR, H. D. G. COMPOSITIONS AND METHODS OF TREATING CELL PROLIFERATION DISORDER. US2006/0160800, 2006.
5. Cruz, F. A.; Dong, V. M., Stereodivergent Coupling of Aldehydes and Alkynes via Synergistic Catalysis Using Rh and Jacobsen's Amine. *J. Am. Chem. Soc.* **2017,** *139* (3), 1029-1032.
6. Davis-Gilbert, Z. W.; Wen, X.; Goodpaster, J. D.; Tonks, I. A., Mechanism of Ti-Catalyzed Oxidative Nitrene Transfer in [2 + 2 + 1] Pyrrole Synthesis from Alkynes and Azobenzene. *J. Am. Chem. Soc.* **2018,** *140* (23), 7267-7281.
7. Chiu, H.-C.; Tonks, I. A., Trimethylsilyl-Protected Alkynes as Selective Cross-Coupling Partners in Titanium-Catalyzed [2+2+1] Pyrrole Synthesis. *Angew. Chem. Int. Ed.* **2018,** *57* (21), 6090-6094.
8. Lei, Z.; Liu, H.; Cai, M., A phosphine-free, atom-efficient cross-coupling of aryl iodides with triarylindiums or trialkynylindiums catalyzed by immobilization of palladium (0) in MCM-41. *J. Organomet. Chem.* **2017,** *852*, 54-63.
9. Briones, J. F.; Davies, H. M. L., Silver Triflate-Catalyzed Cyclopropenation of Internal Alkynes with Donor-/Acceptor-Substituted Diazo Compounds. *Org. Lett.* **2011,** *13* (15), 3984-3987.
10. Briones, J. F.; Davies, H. M. L., Gold(I)-Catalyzed Asymmetric Cyclopropenation of Internal Alkynes. *J. Am. Chem. Soc.* **2012,** *134* (29), 11916-11919.
11. Davies, H. M. L.; Lee, G. H., Dirhodium(II) Tetra(N-(dodecylbenzenesulfonyl)prolinate) Catalyzed Enantioselective Cyclopropenation of Alkynes. *Org. Lett.* **2004,** *6* (8), 1233-1236.

VIII. NMR Spectra

VIIIa. NMR Spectra of Starting Materials

^{1}H NMR of **S2**

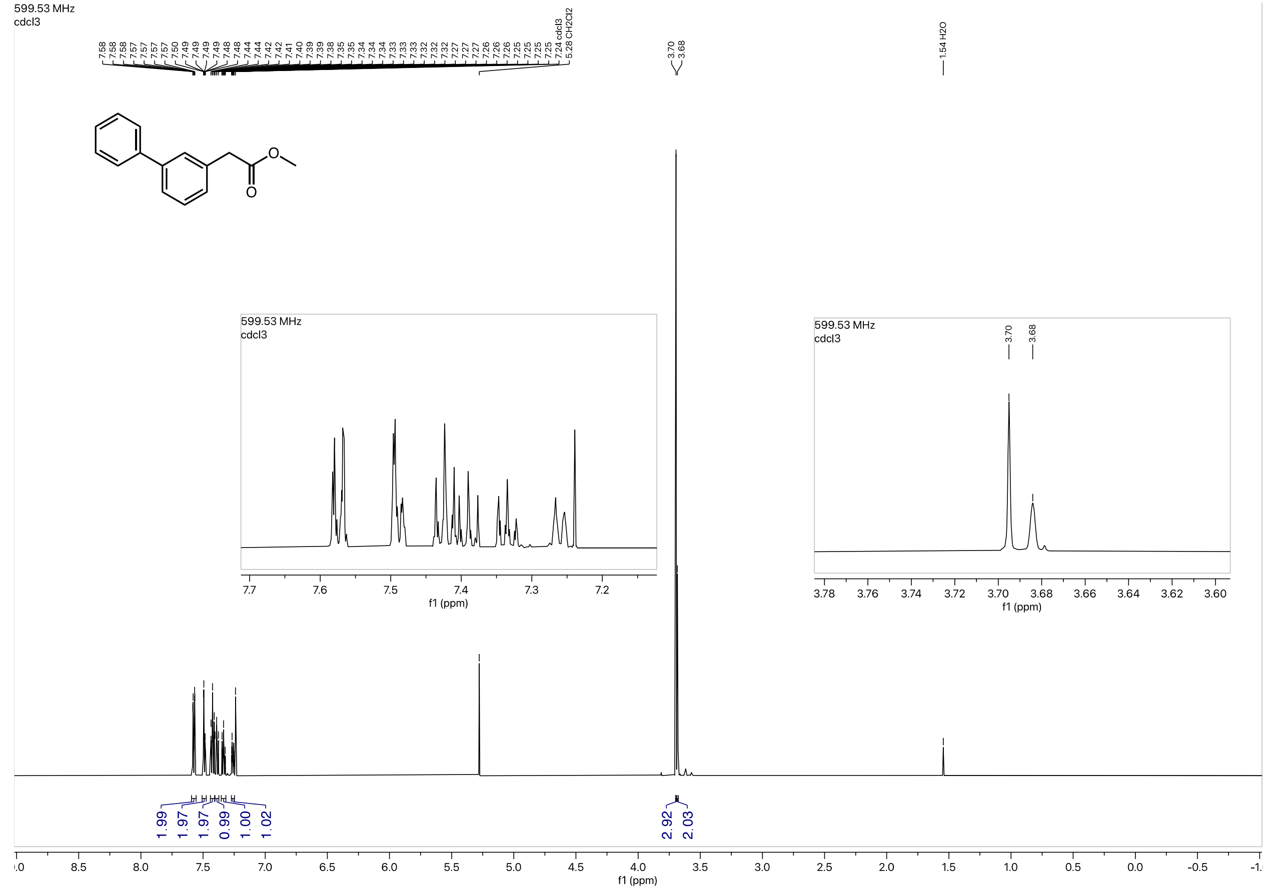

^{13}C NMR of **S2**

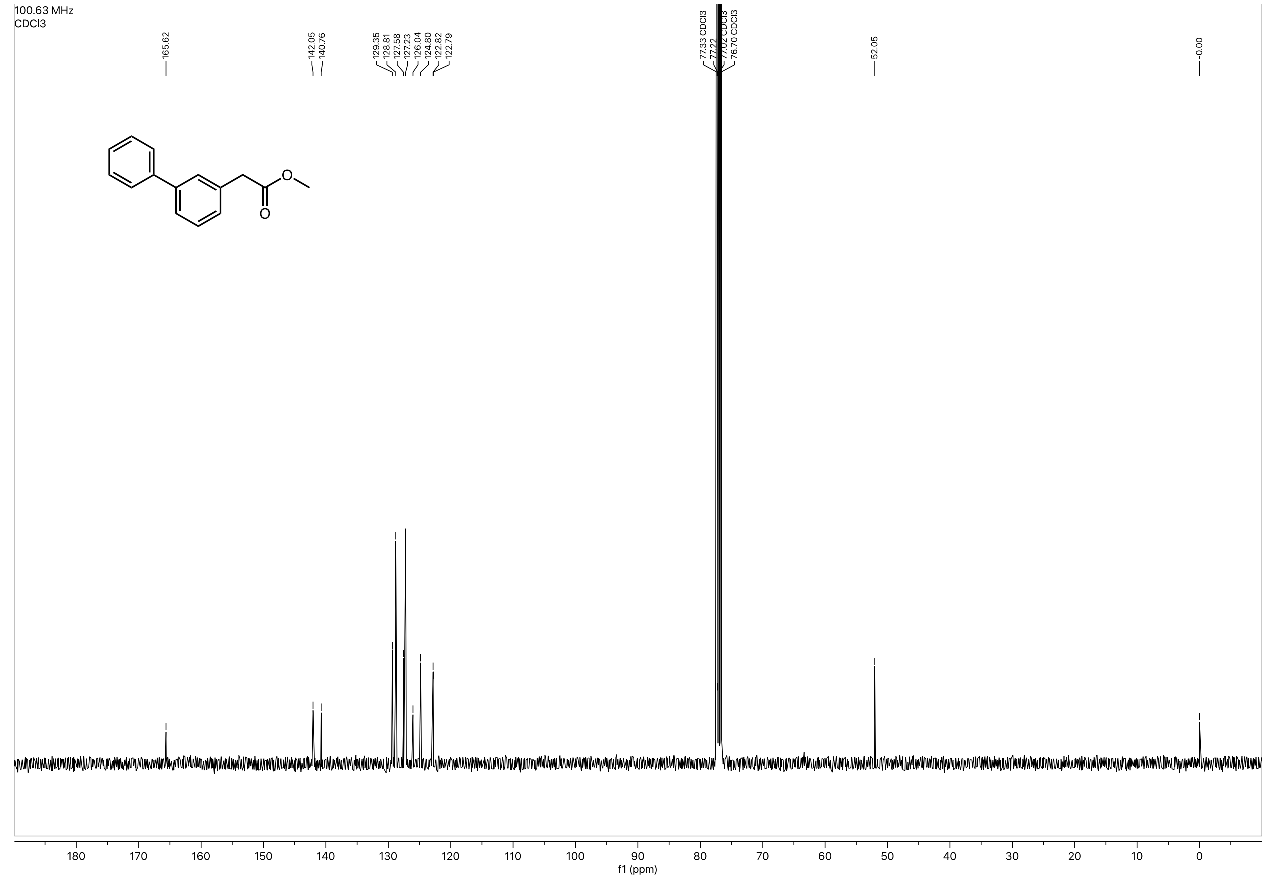

^{1}H NMR of **S3**

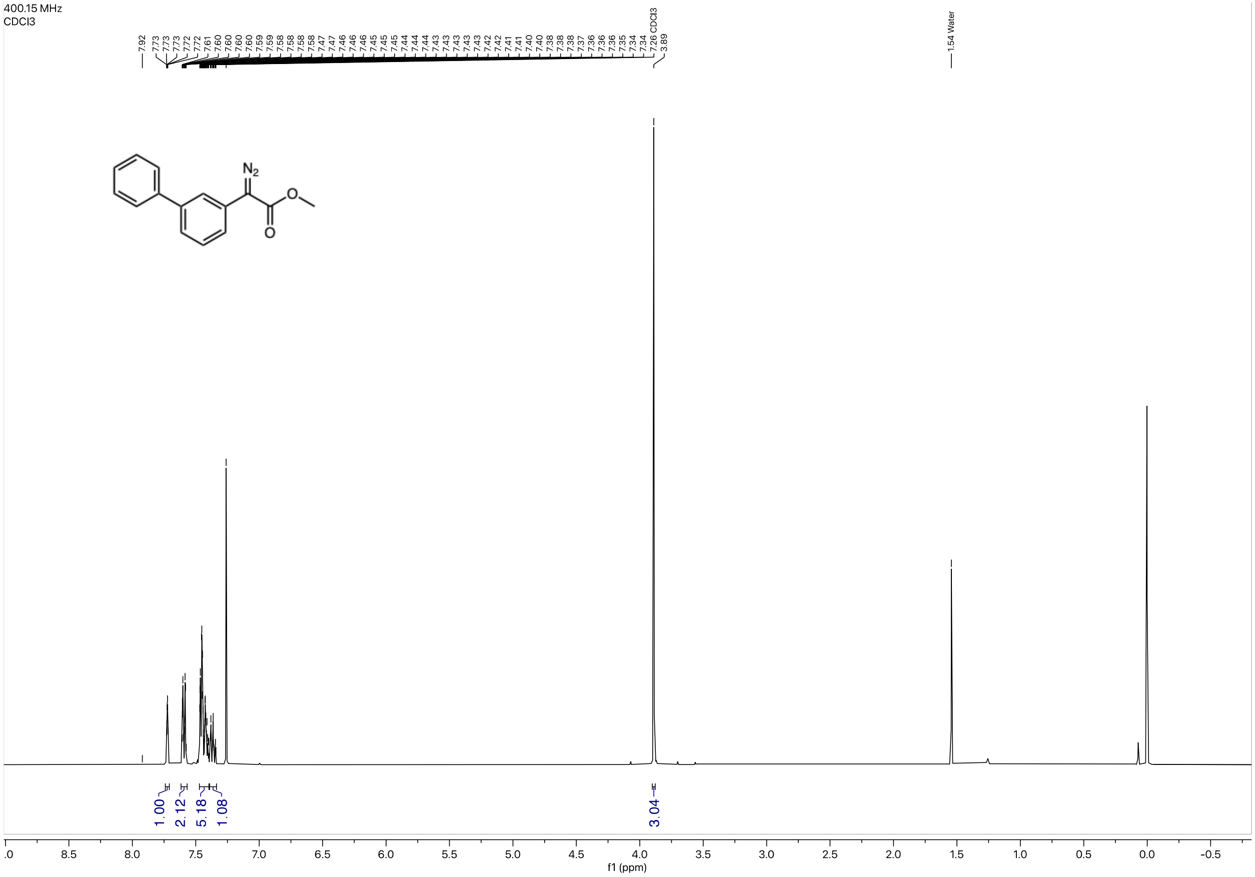

^{13}C NMR of **S3**

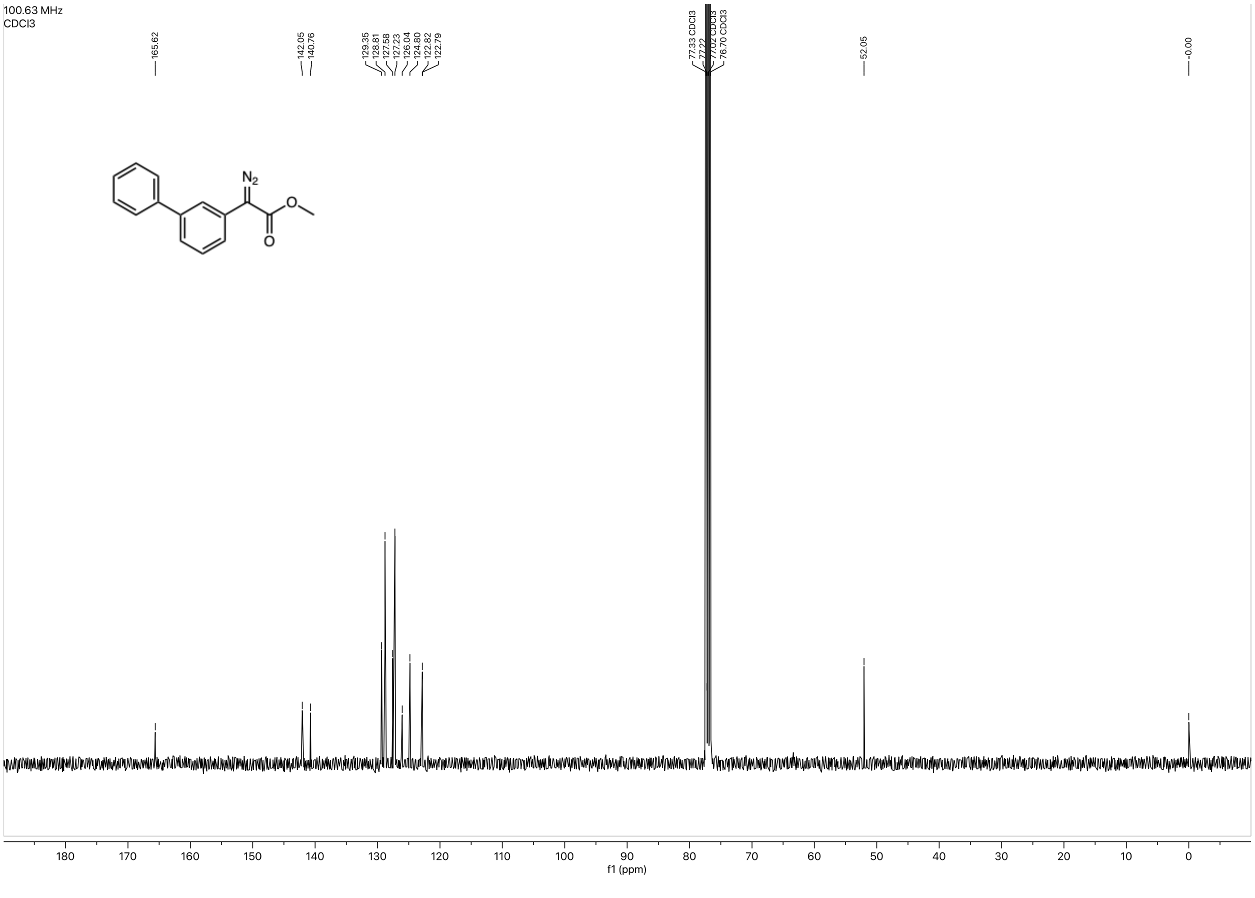

^{1}H NMR of **S4**

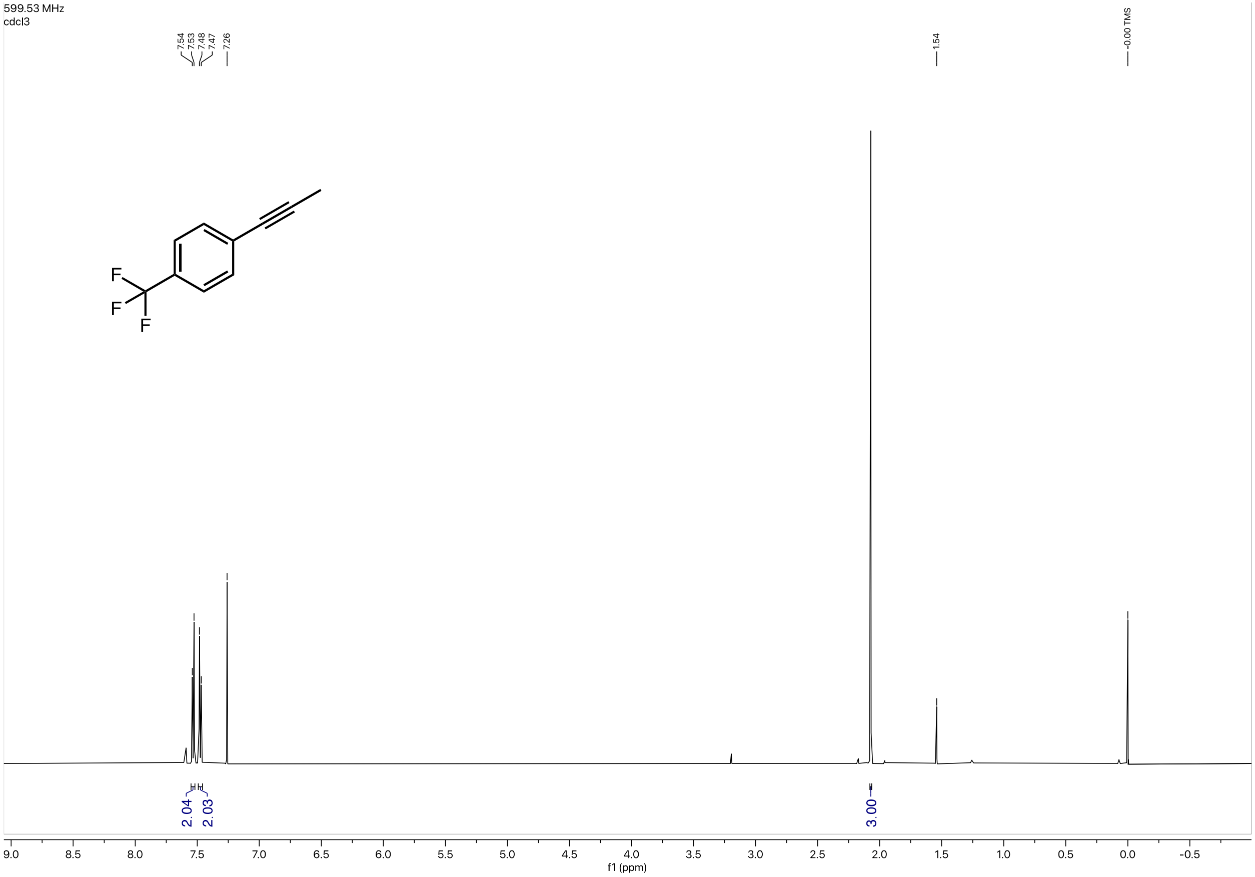

599.53 MHz
cdcl3
7.54
7.53
7.48
7.47
7.26
1.54
-0.00 TMS
F
F
F
2.04
2.03
3.00
9.0
8.5
8.0
7.5
7.0
6.5
6.0
5.5
5.0
4.5
4.0
3.5
3.0
2.5
2.0
1.5
1.0
0.5
0.0
-0.5
f1 (ppm)

^{1}H NMR of **S5**

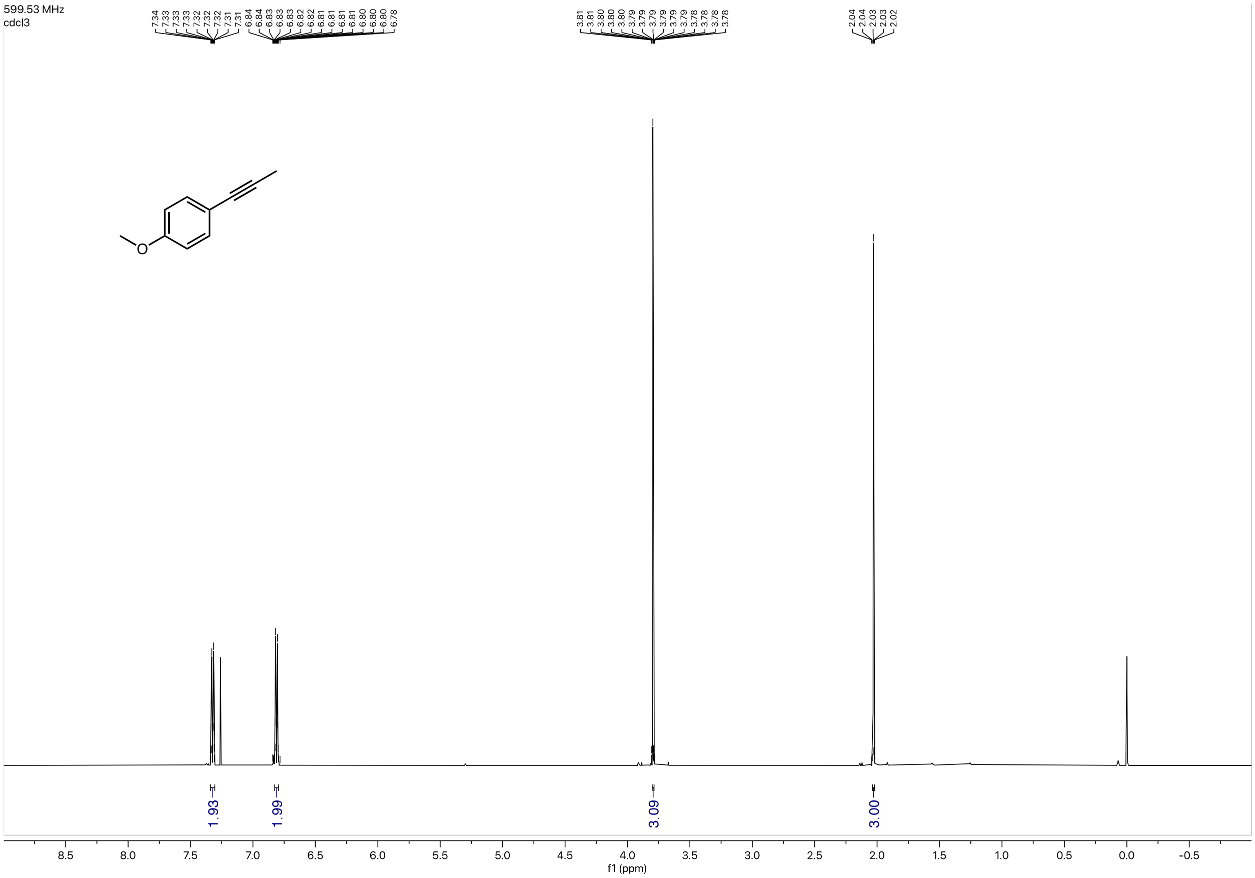

^{1}H NMR of **S6**

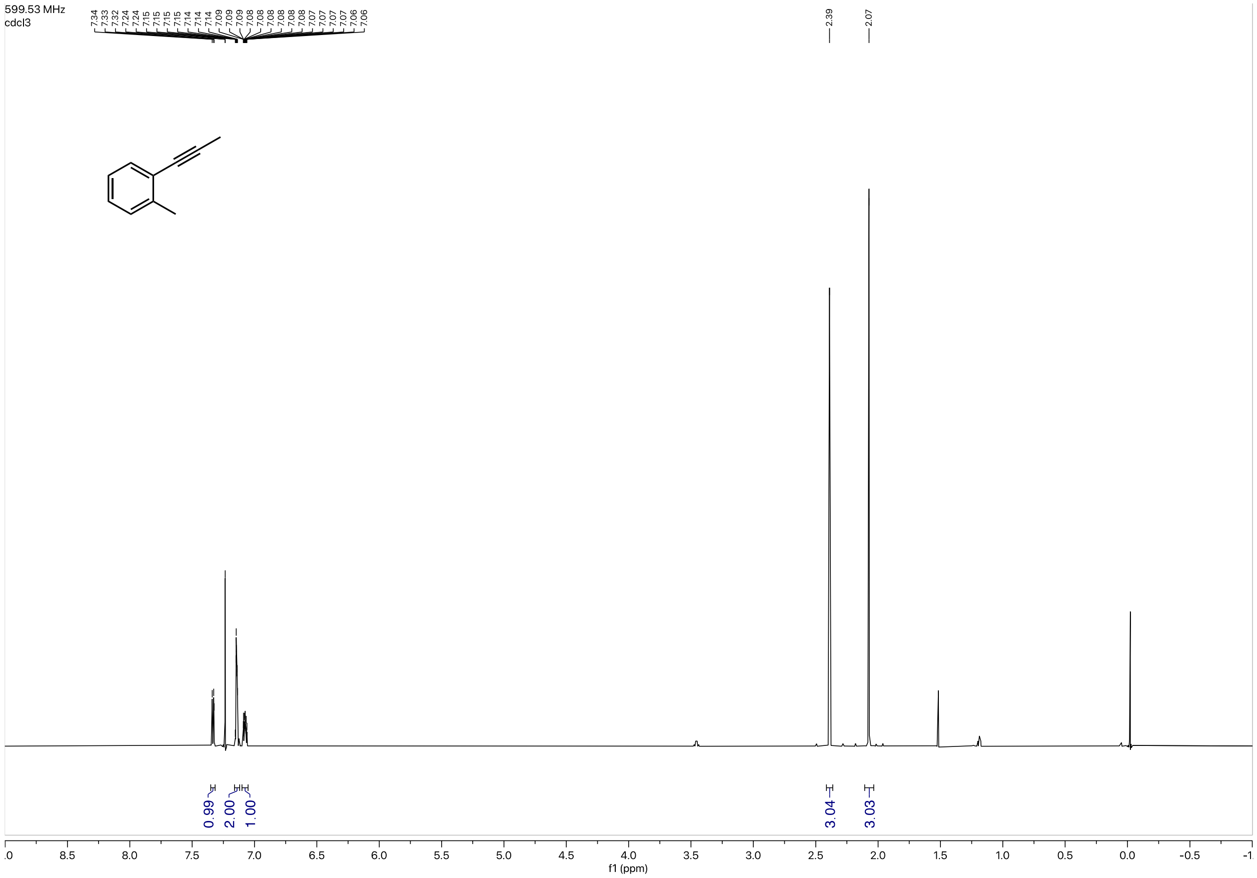
599.53 MHz
cdcl3
7.34
7.33
7.32
7.24
7.24
7.15
7.15
7.15
7.15
7.14
7.14
7.09
7.09
7.09
7.08
7.08
7.08
7.08
7.08
7.07
7.07
7.07
7.06
7.06
2.39
2.07
0.99
2.00
1.00
3.04
3.03
f1 (ppm)

^{1}H NMR of **S7**

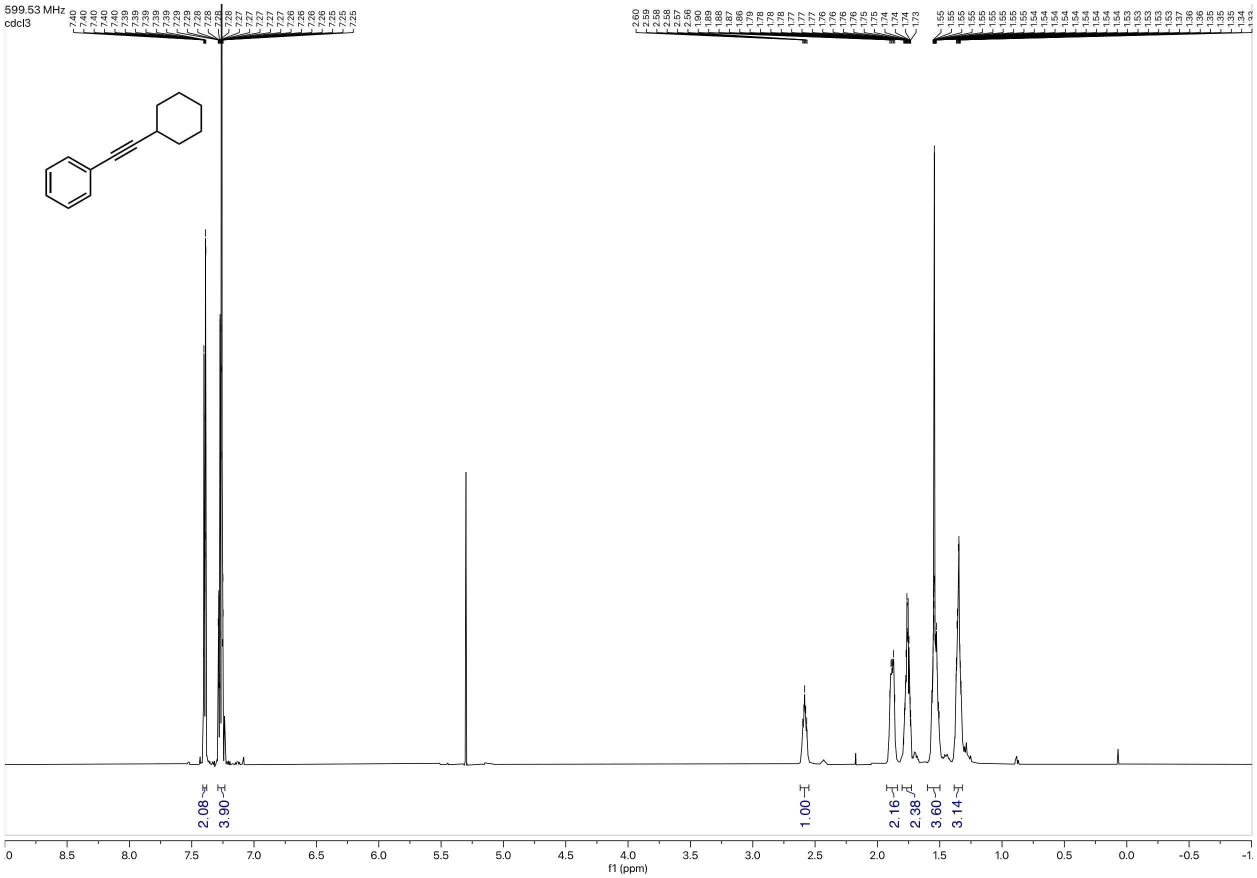

^{1}H NMR of **S8**

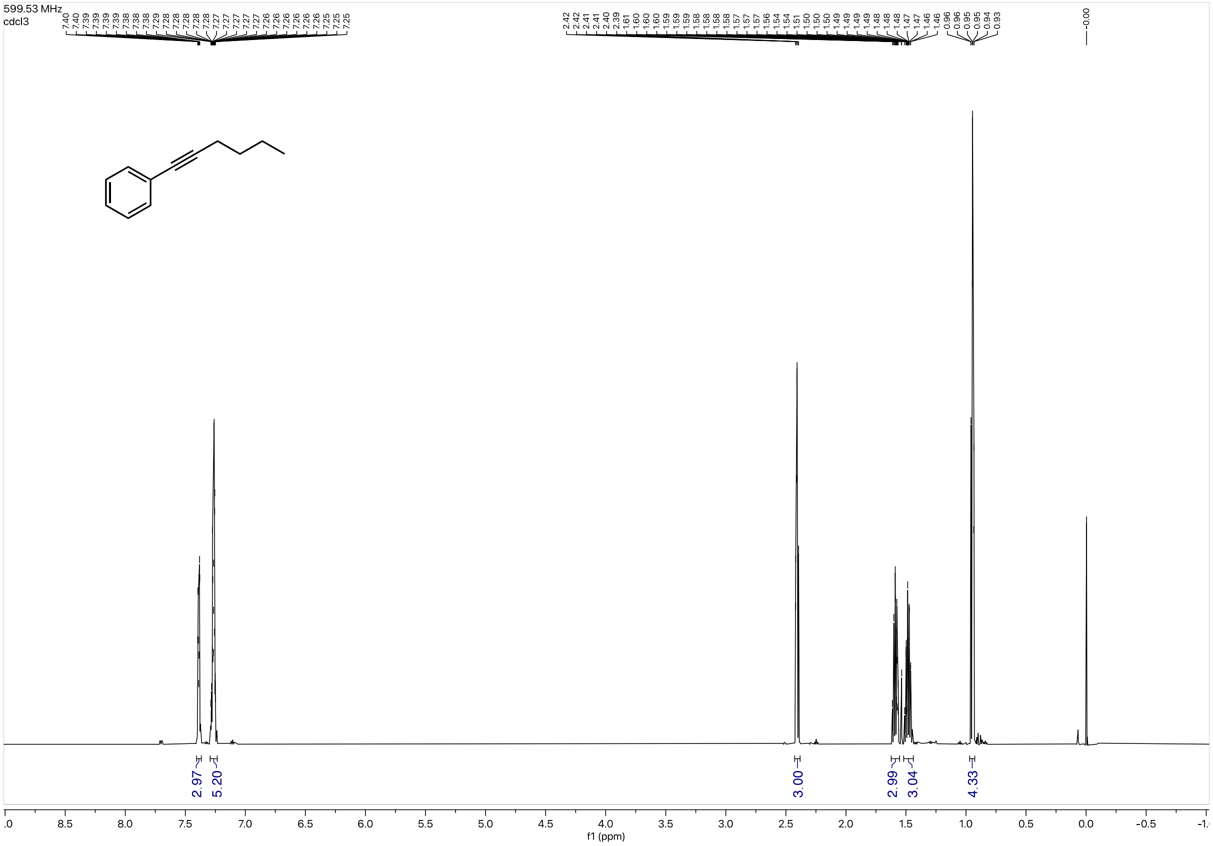

^{1}H NMR of **18**

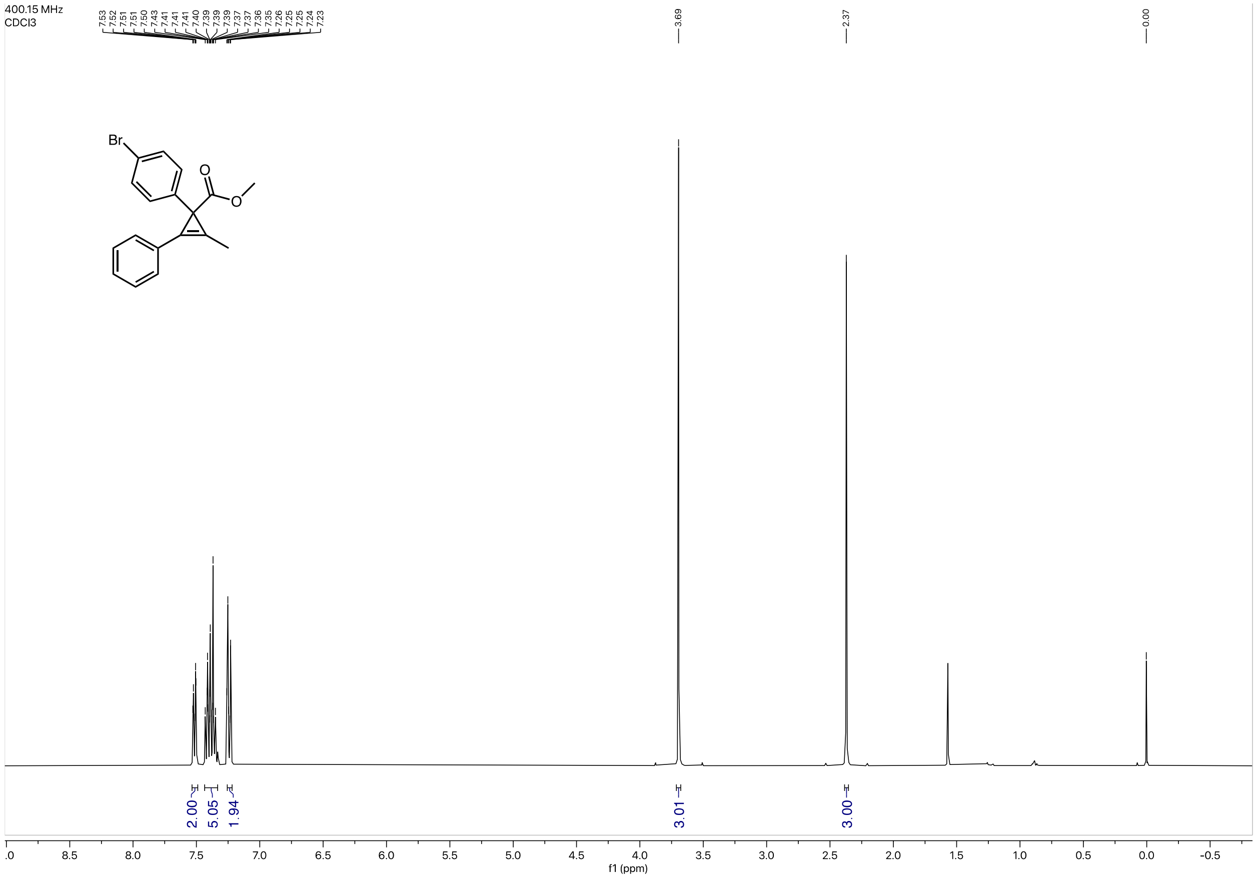
400.15 MHz
CDCl3
7.53
7.52
7.51
7.50
7.43
7.41
7.41
7.40
7.39
7.39
7.39
7.37
7.37
7.36
7.35
7.26
7.25
7.24
7.23
3.69
2.37
0.00
Br
O
O
2.00
5.05
1.94
3.01
3.00
f1 (ppm)

^{1}H NMR of **19a**

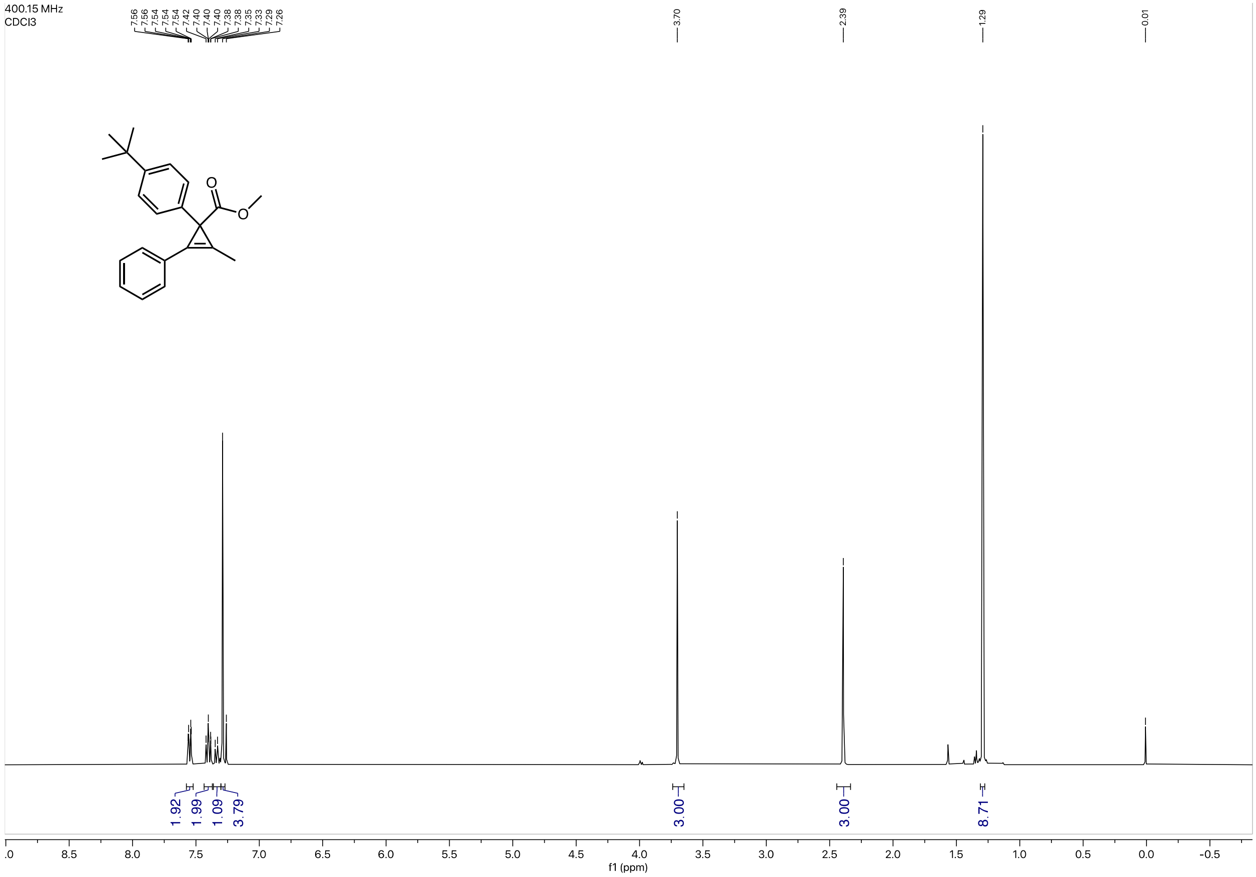
400.15 MHz
CDCl3
7.56
7.56
7.54
7.54
7.54
7.42
7.40
7.40
7.40
7.38
7.38
7.35
7.33
7.29
7.26
3.70
2.39
1.29
0.01
1.92
1.99
1.09
3.79
3.00
3.00
8.71
f1 (ppm)

^{1}H NMR of **19b**

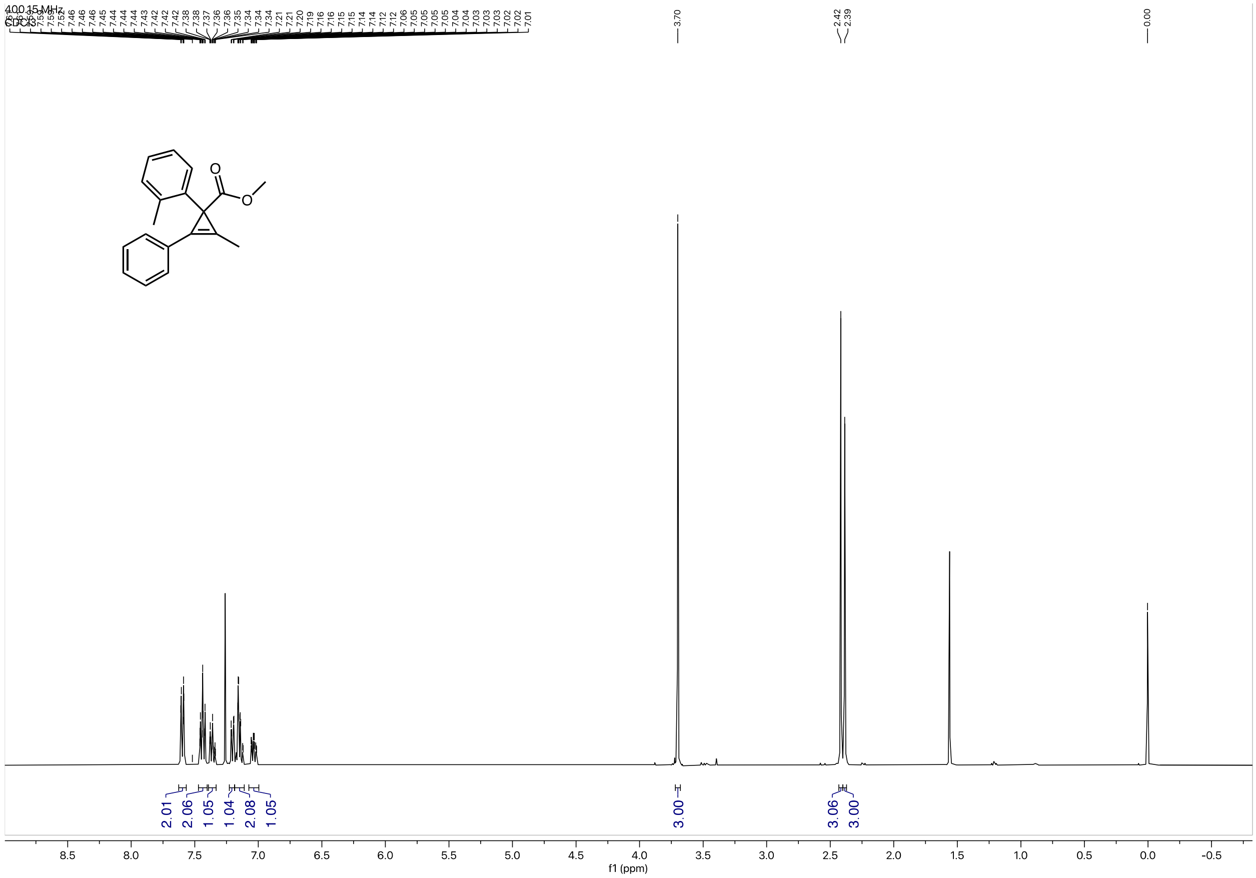

^{1}H NMR of **19c**

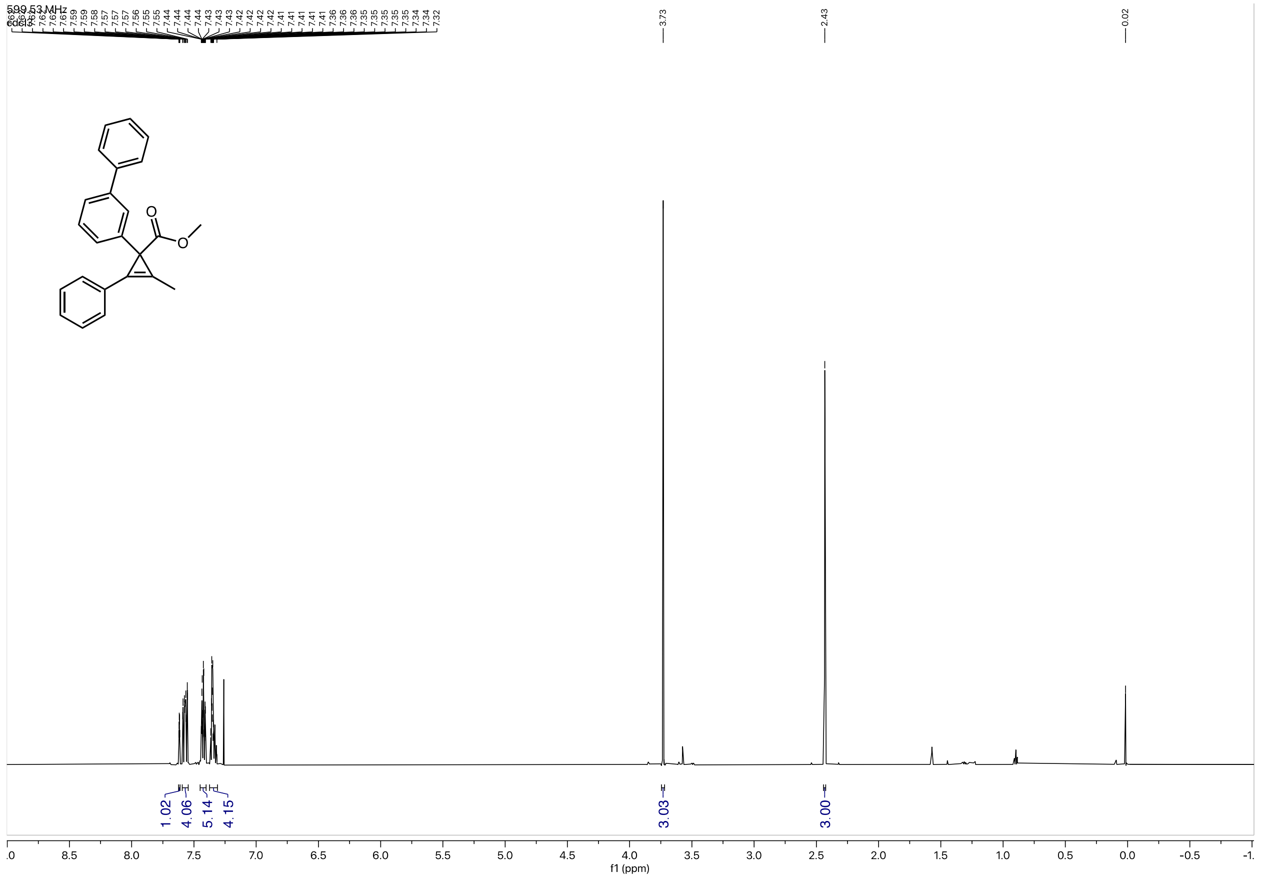

^{1}H NMR of **19d**

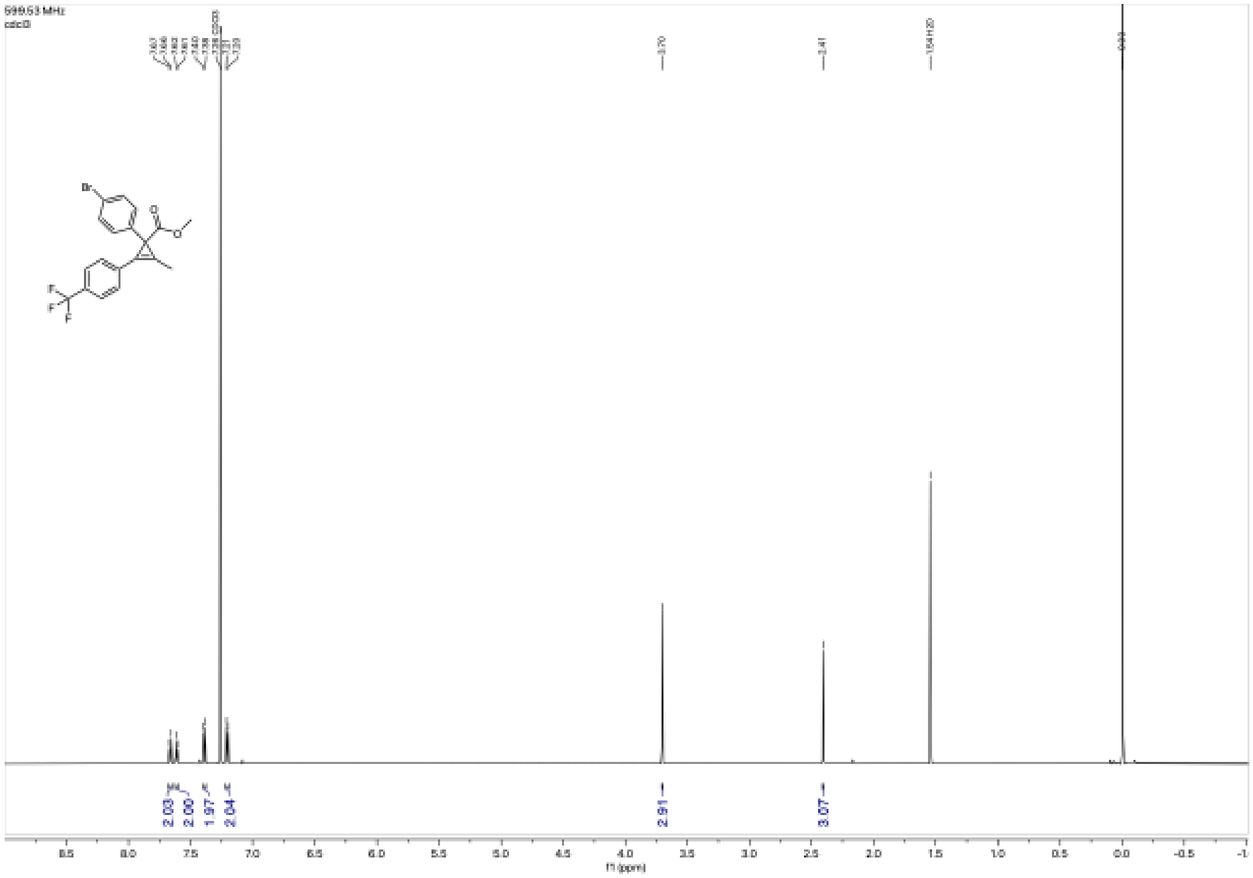

^{13}C NMR of **19d**

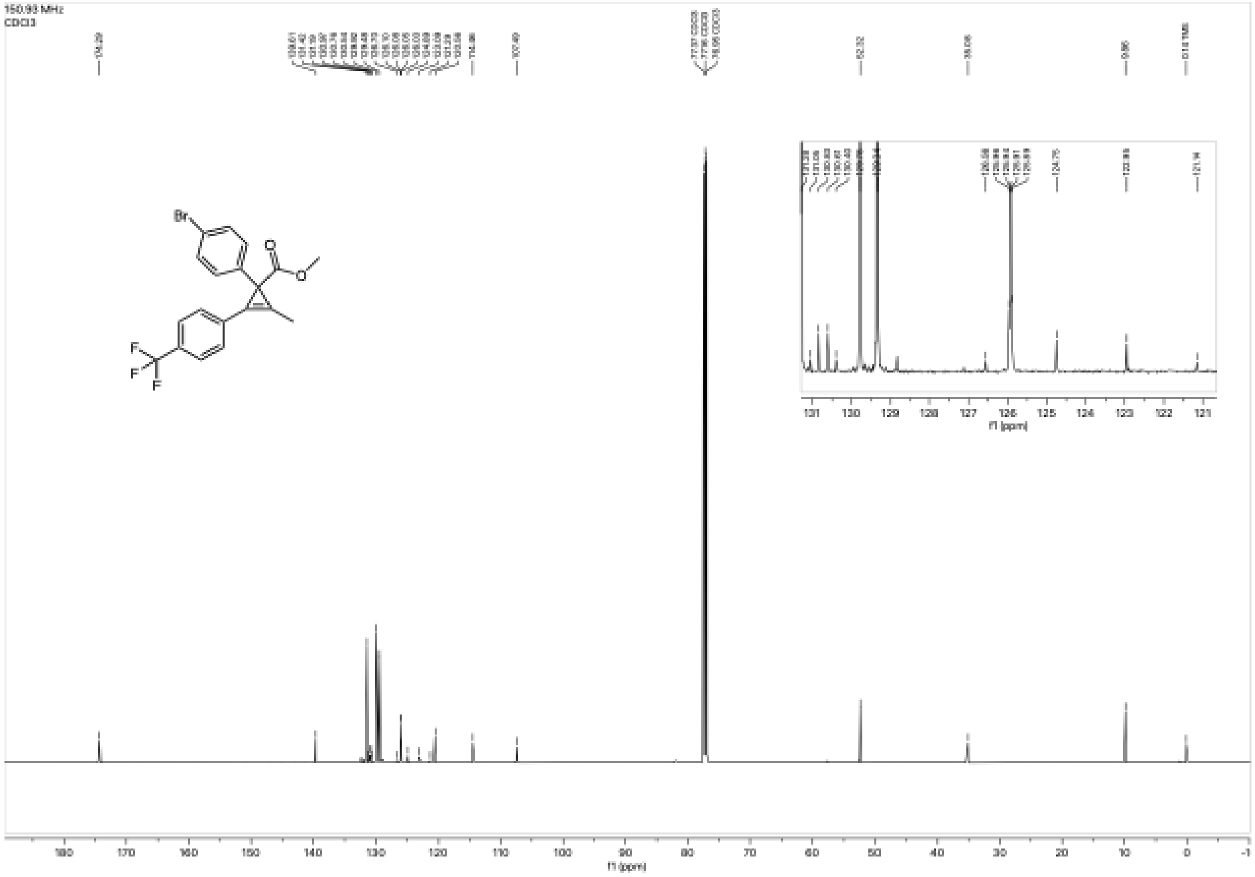

^{19}F NMR of **19d**

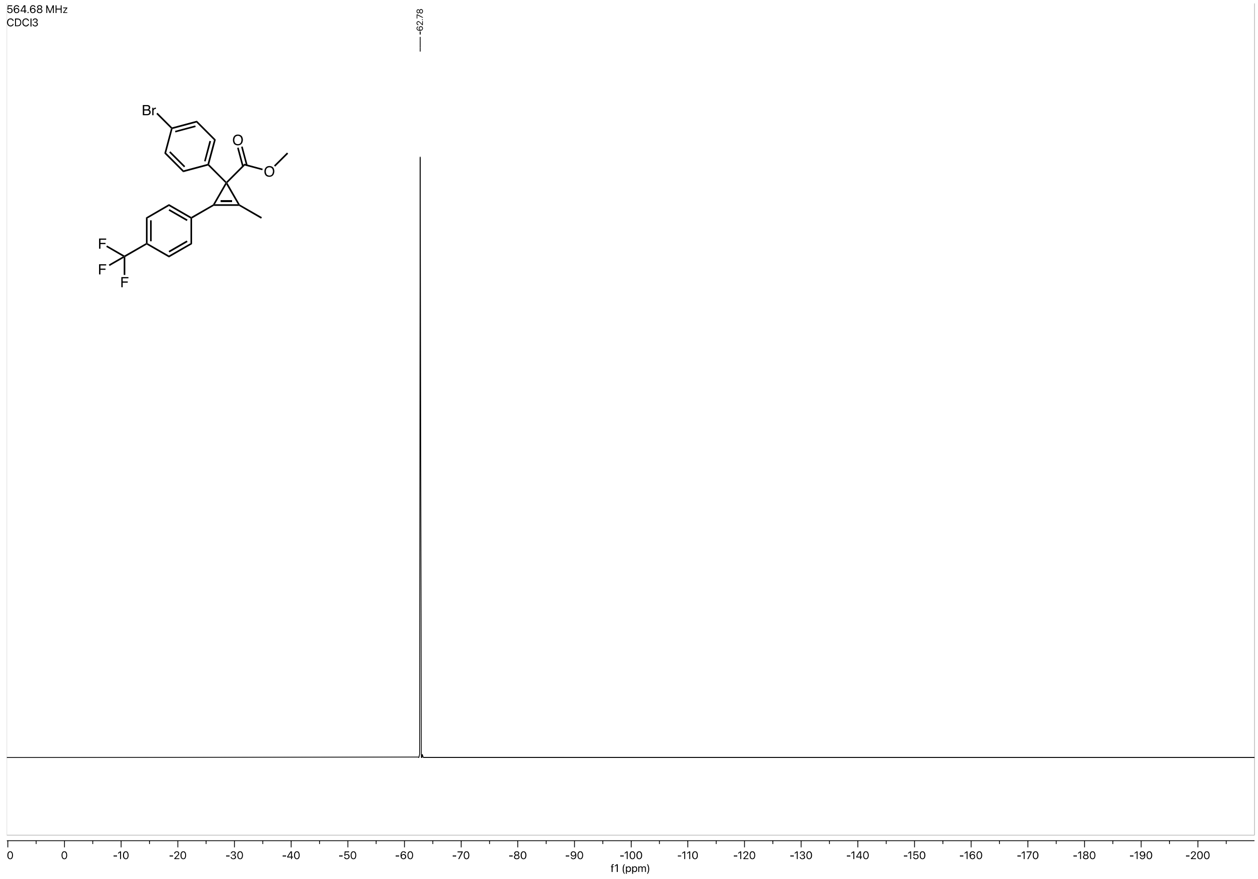

^{1}H NMR of **19e**

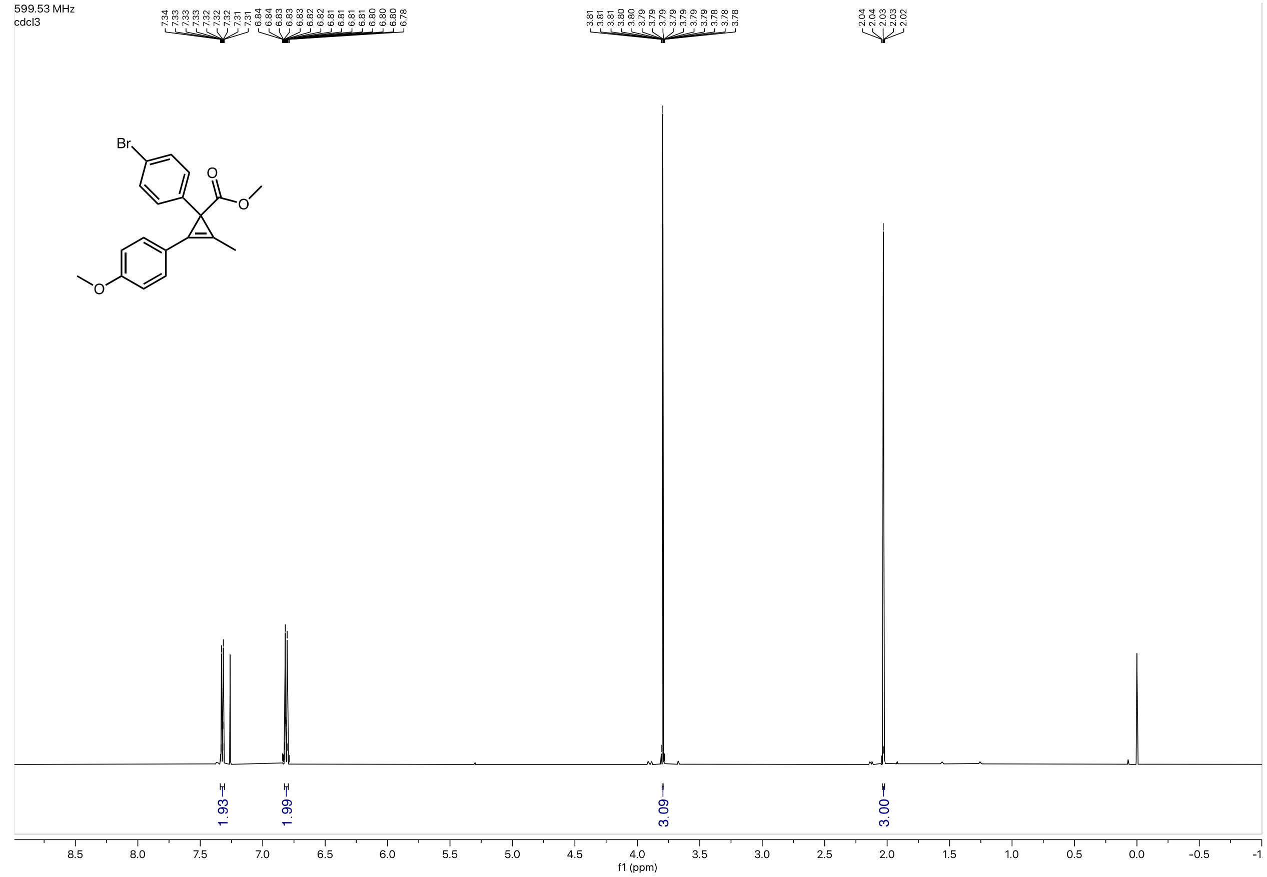

^{13}C NMR of **19e**

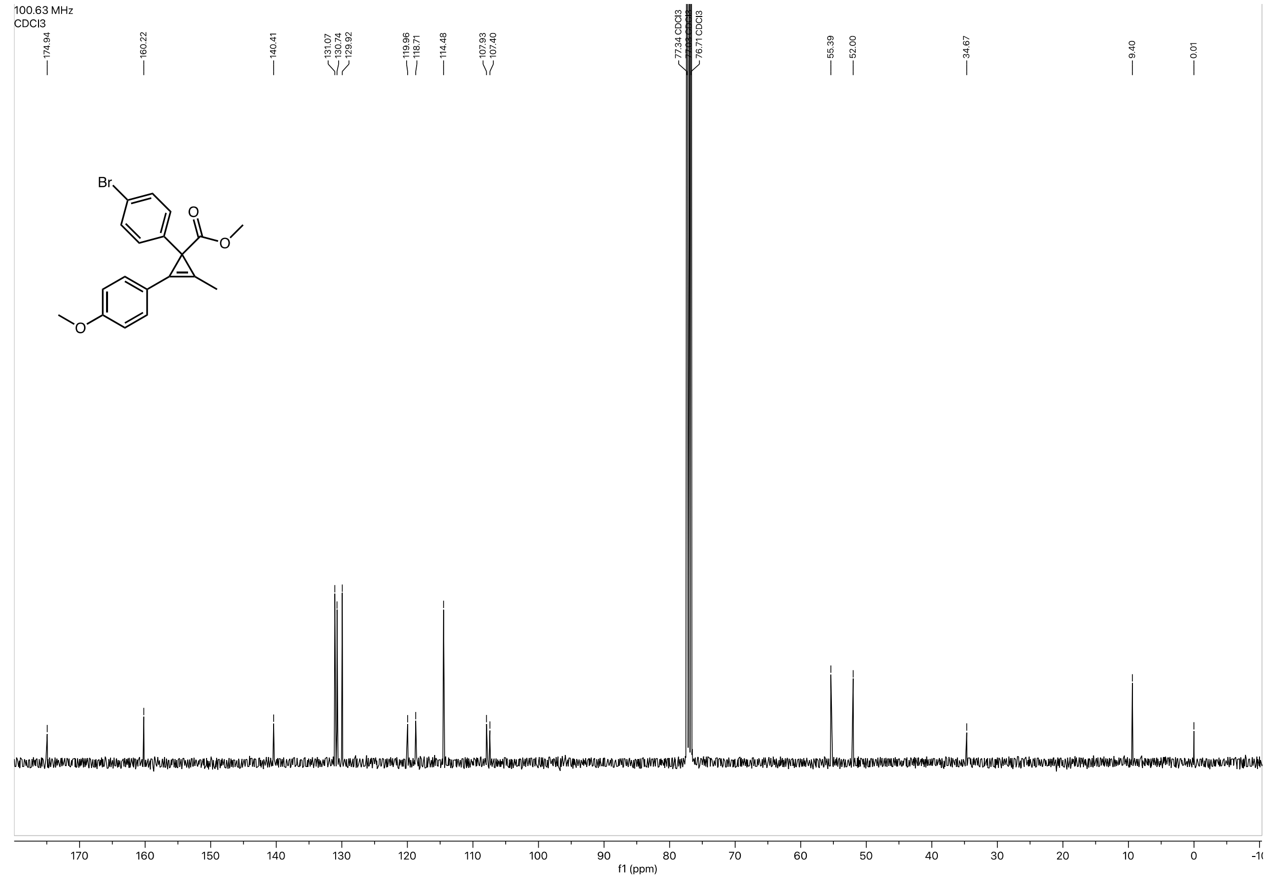

^{1}H NMR of **19f**

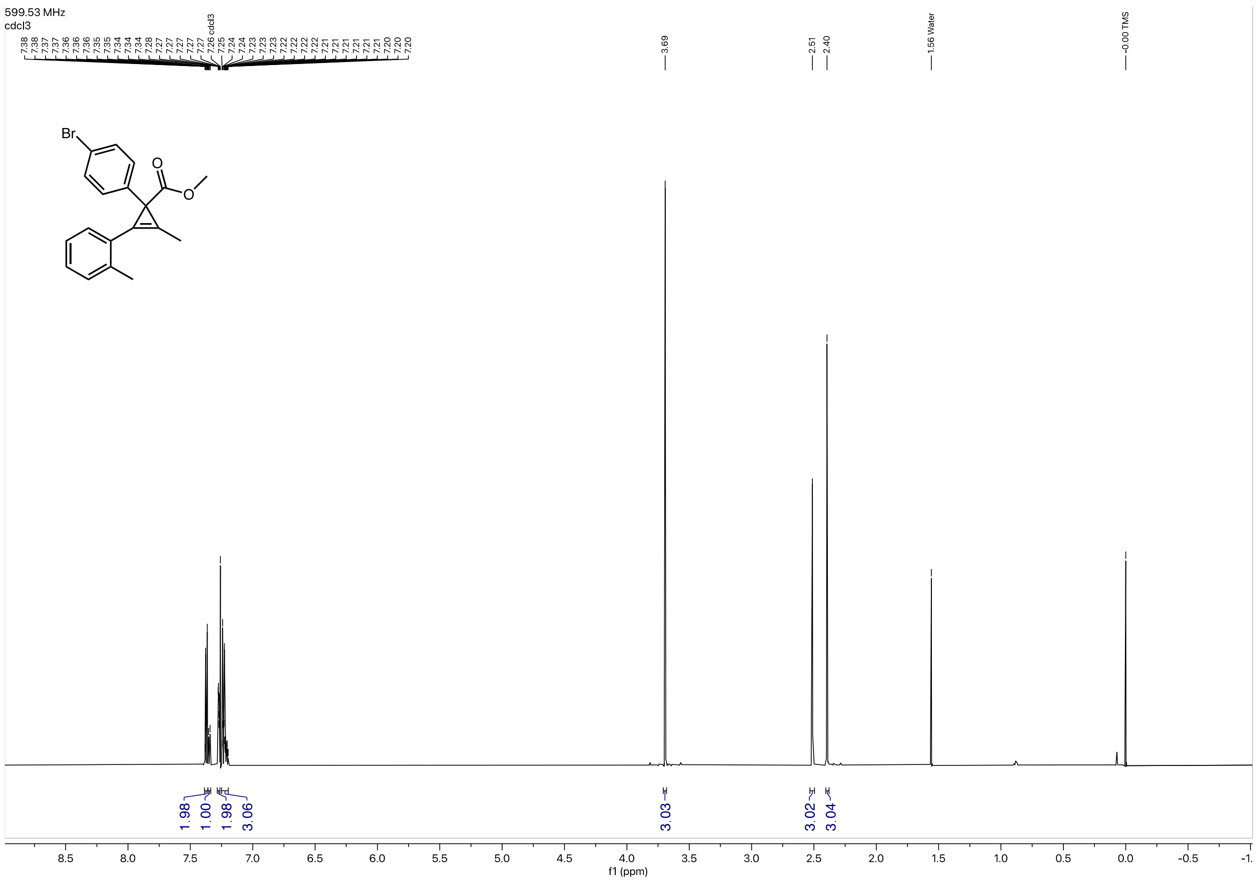

^{13}C NMR of **19f**

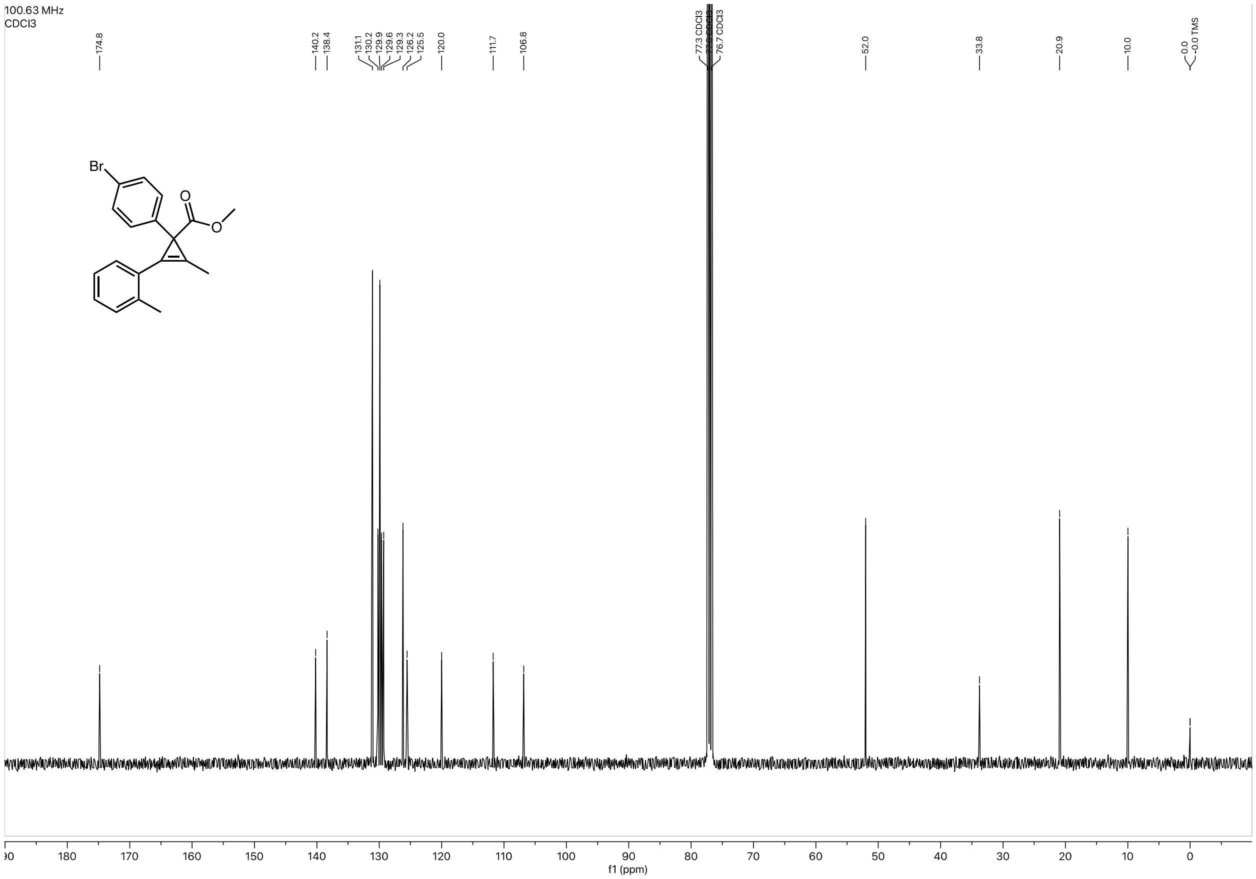

^{1}H NMR of **22a**

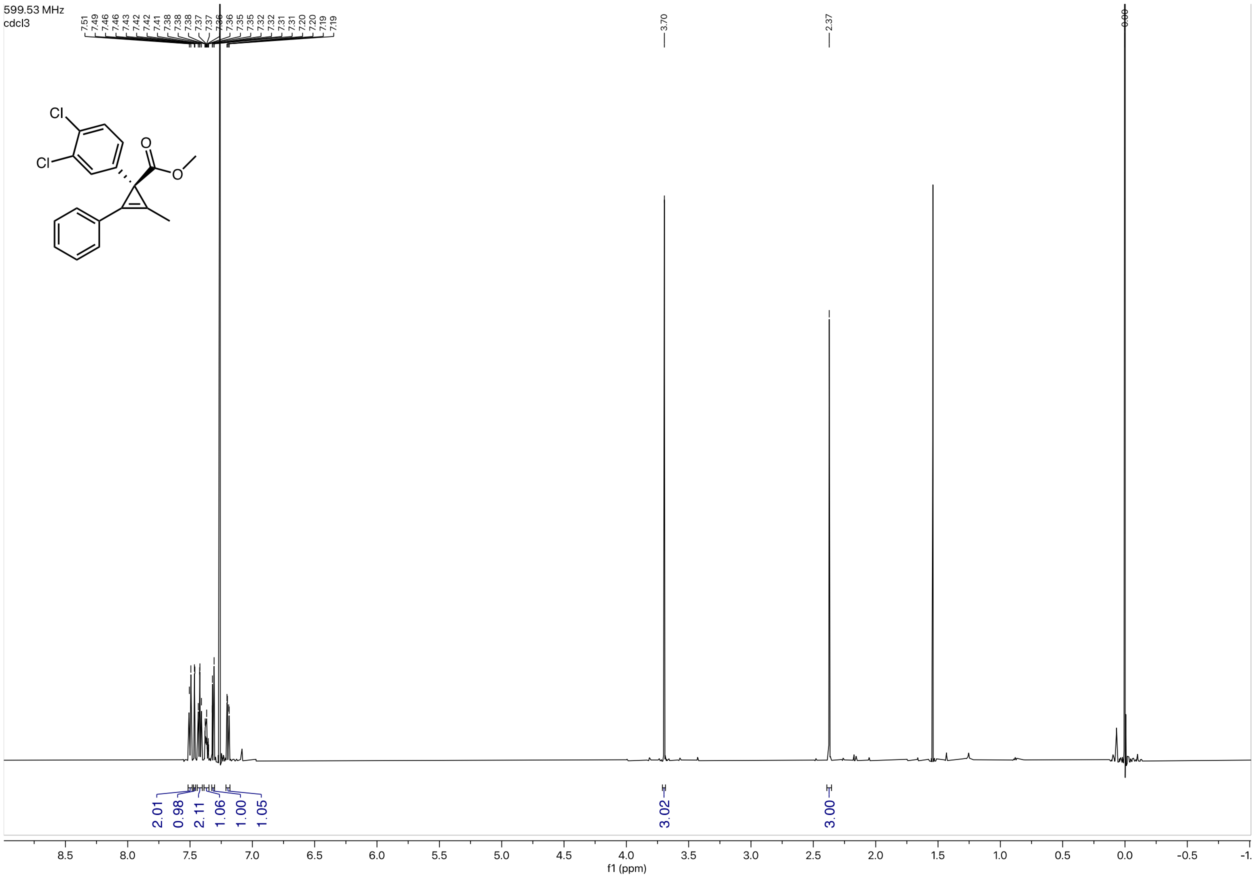

599.53 MHz
cdcl3
7.51
7.49
7.46
7.46
7.43
7.42
7.42
7.41
7.38
7.38
7.38
7.37
7.37
7.36
7.36
7.35
7.35
7.32
7.32
7.31
7.31
7.20
7.20
7.19
7.19
3.70
2.37
0.00
Cl
Cl
O
O
2.01
0.98
2.11
1.06
1.00
1.05
3.02
3.00
8.5
8.0
7.5
7.0
6.5
6.0
5.5
5.0
4.5
4.0
3.5
3.0
2.5
2.0
1.5
1.0
0.5
0.0
-0.5
f1 (ppm)

^{1}H NMR of **22b**

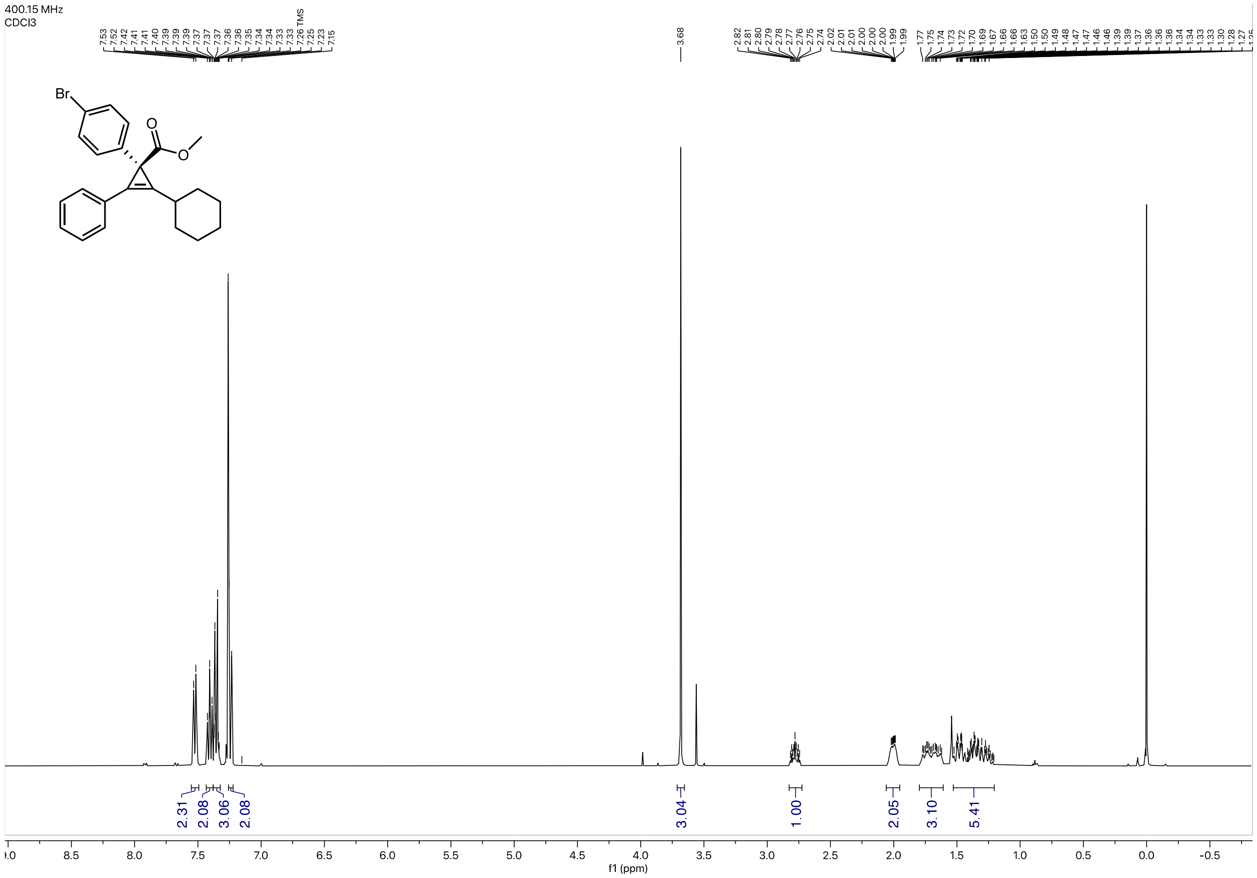

^{13}C NMR of **22b**

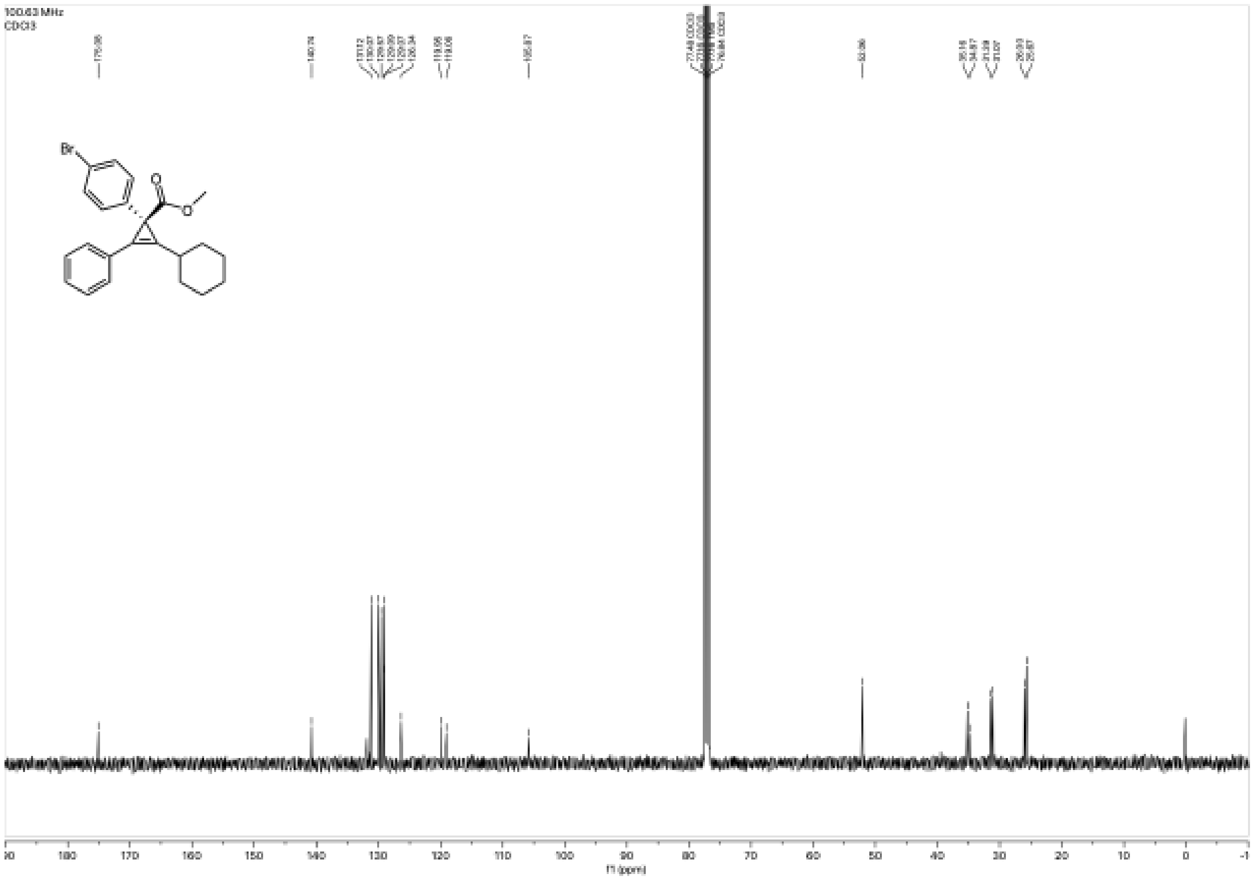

^{1}H NMR of **22c**

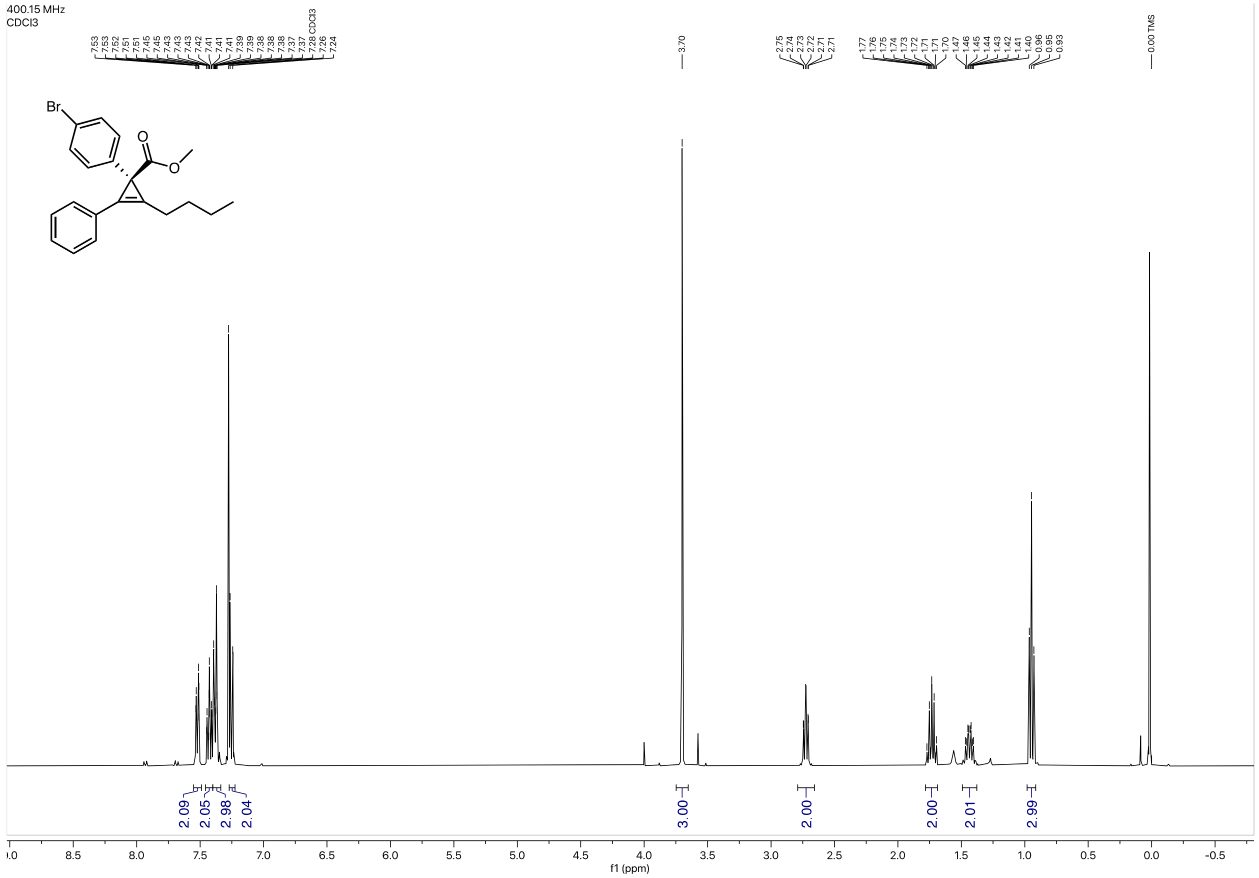

^{13}C NMR of **22c**

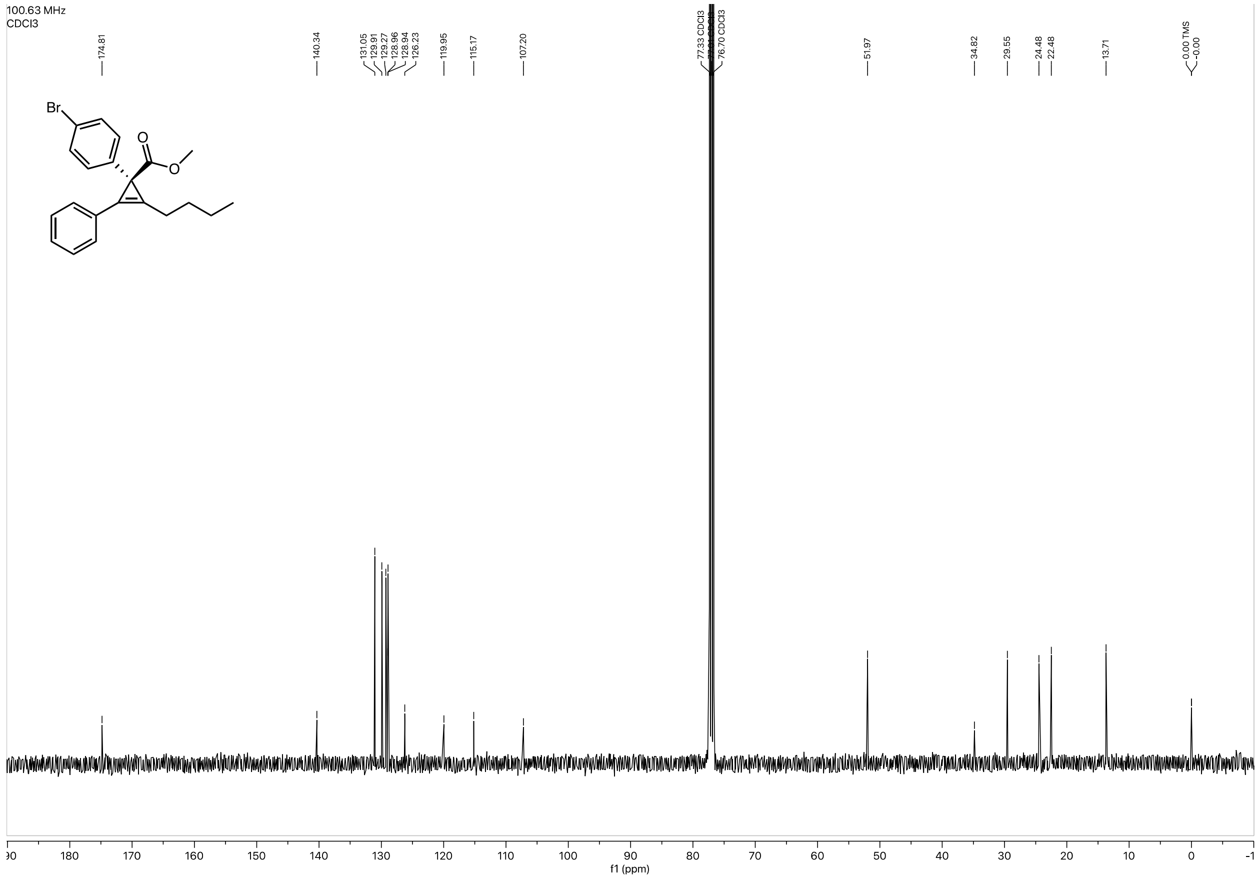

^1H NMR of **22d**

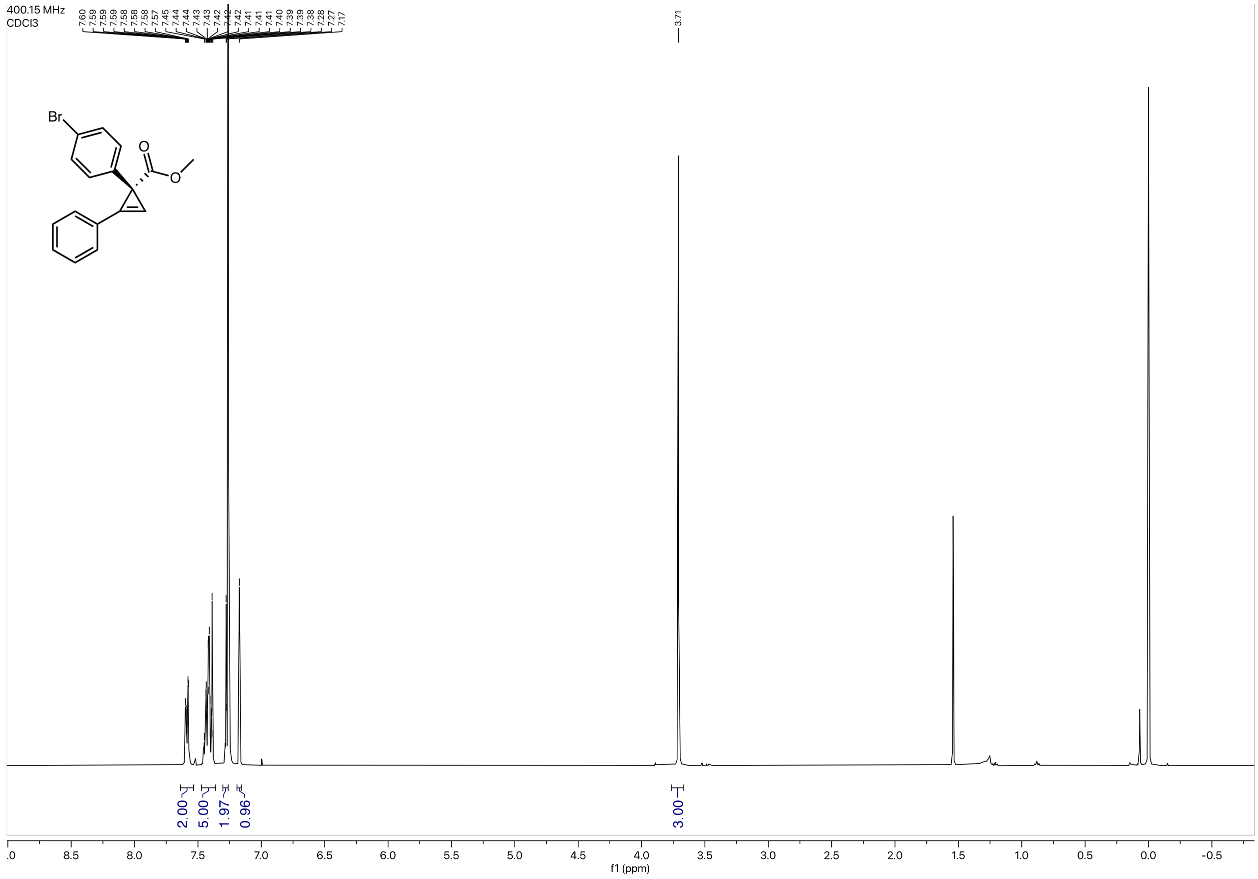
400.15 MHz
CDCl3
7.60
7.59
7.59
7.58
7.58
7.58
7.57
7.45
7.44
7.44
7.43
7.43
7.42
7.42
7.42
7.41
7.41
7.40
7.39
7.39
7.38
7.28
7.27
7.17
3.71
Br
O
O
2.00
5.00
1.97
0.96
3.00
f1 (ppm)

VIIIb. NMR of Bicyclo[2.1.0]pentane Products

^{1}H NMR of **18a** (-50 ºC)

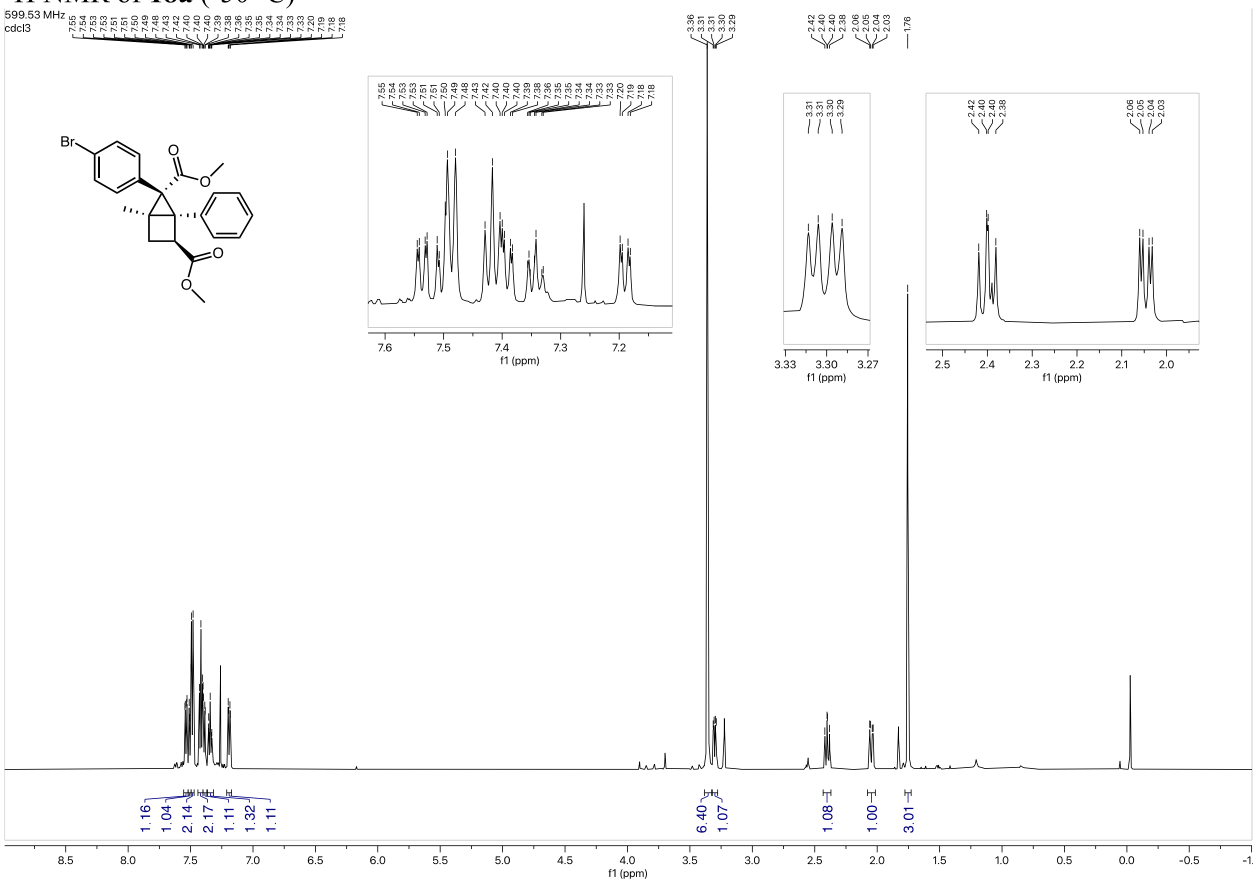

^{13}C NMR of **18a**

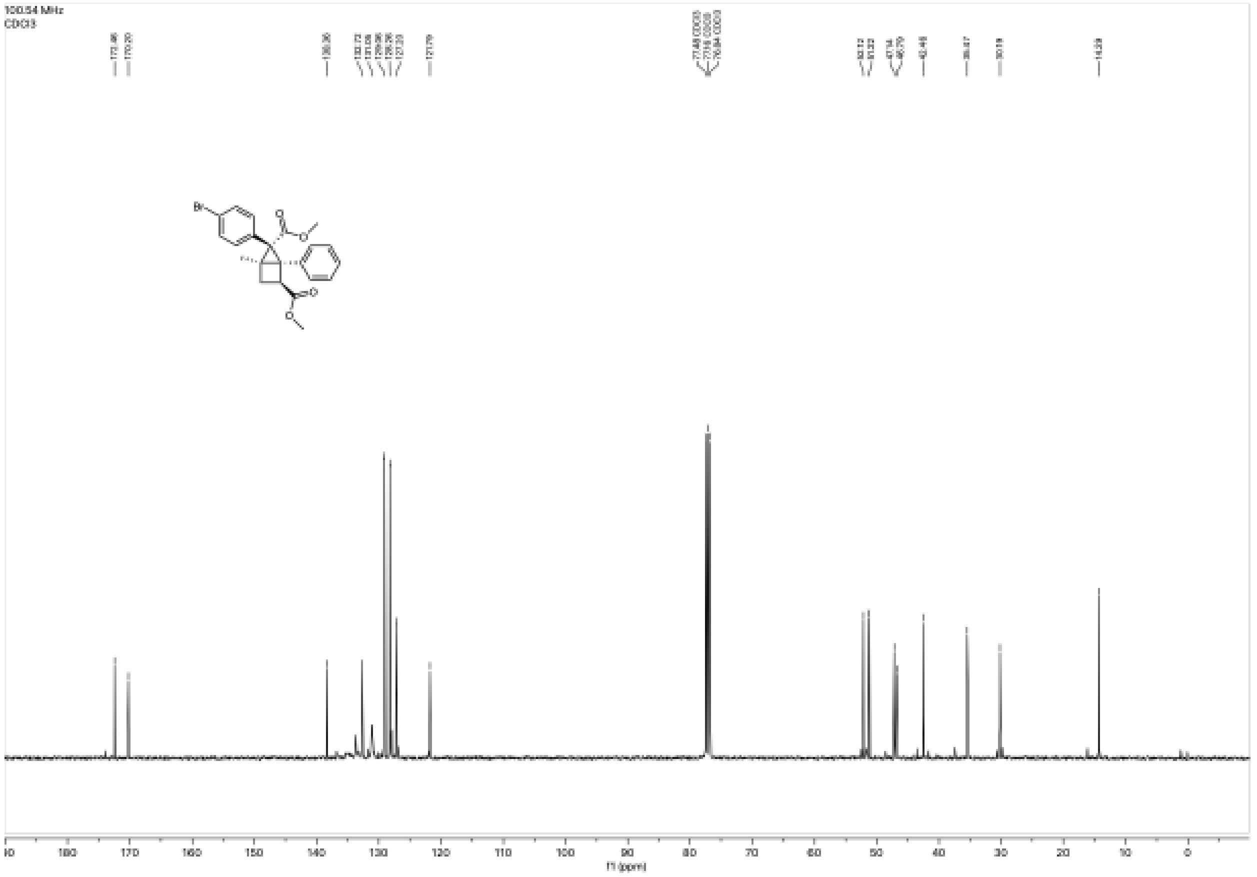

^{1}H NMR of **21a** (-50 ºC)

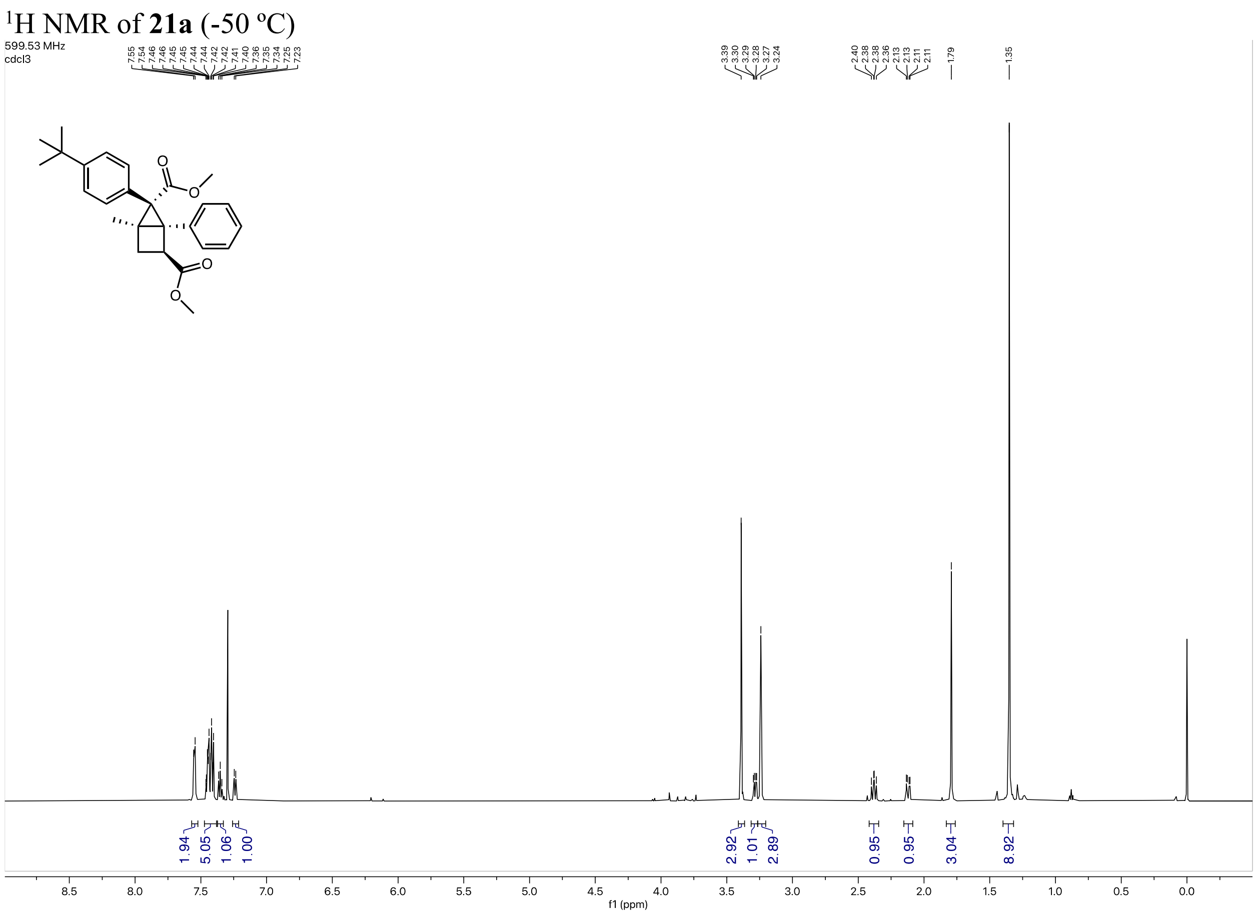

^{13}C NMR of **21a**

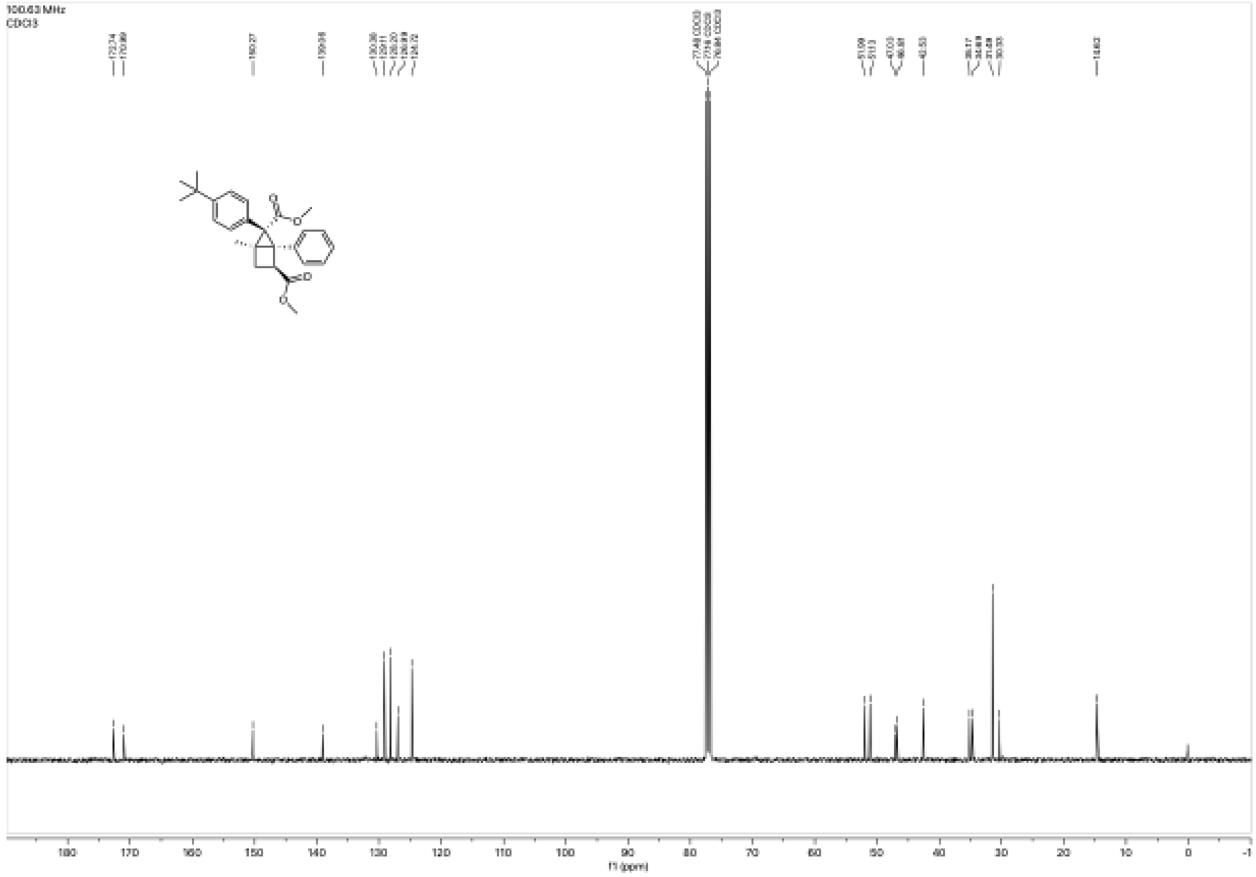

^{1}H NMR of **21b** (-50 ºC)

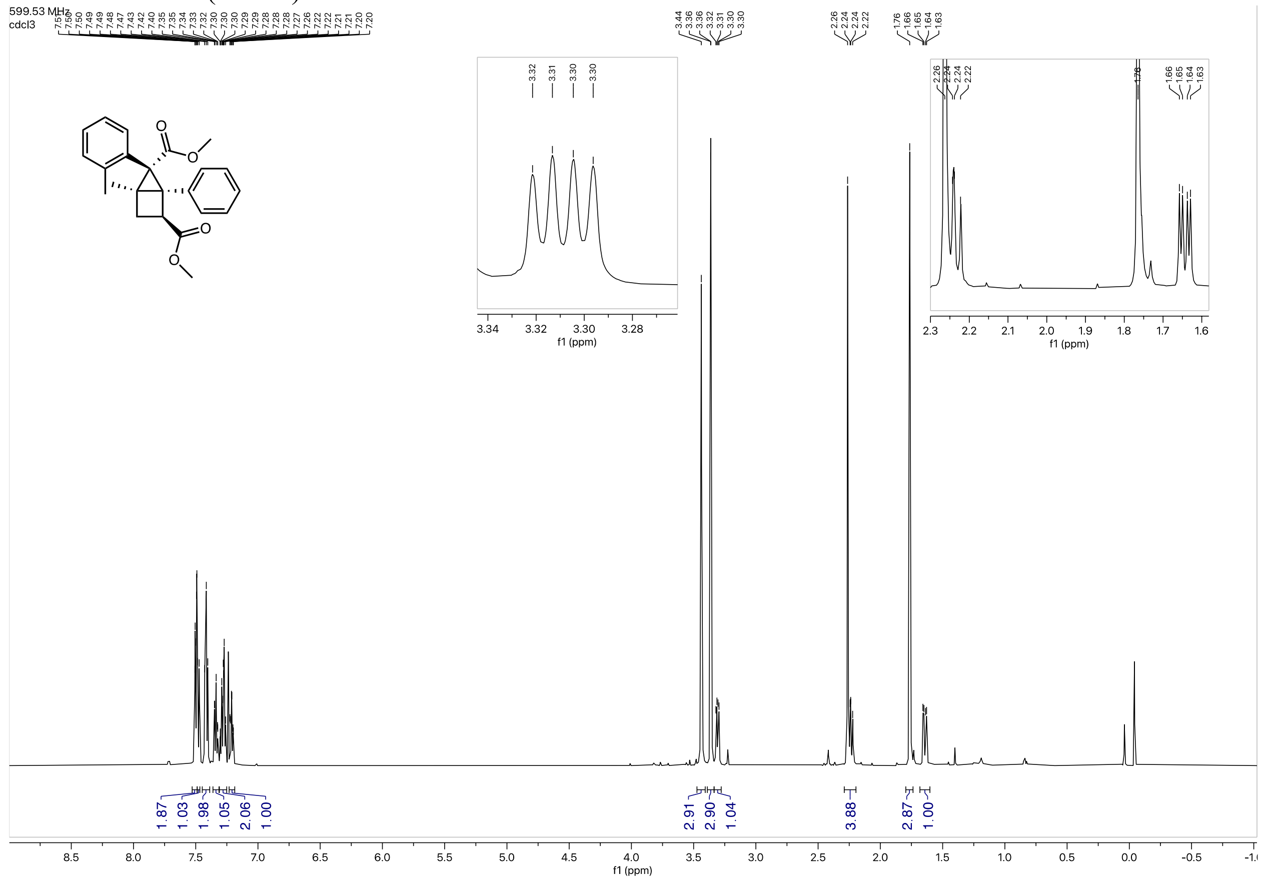

^{13}C NMR of **21b**

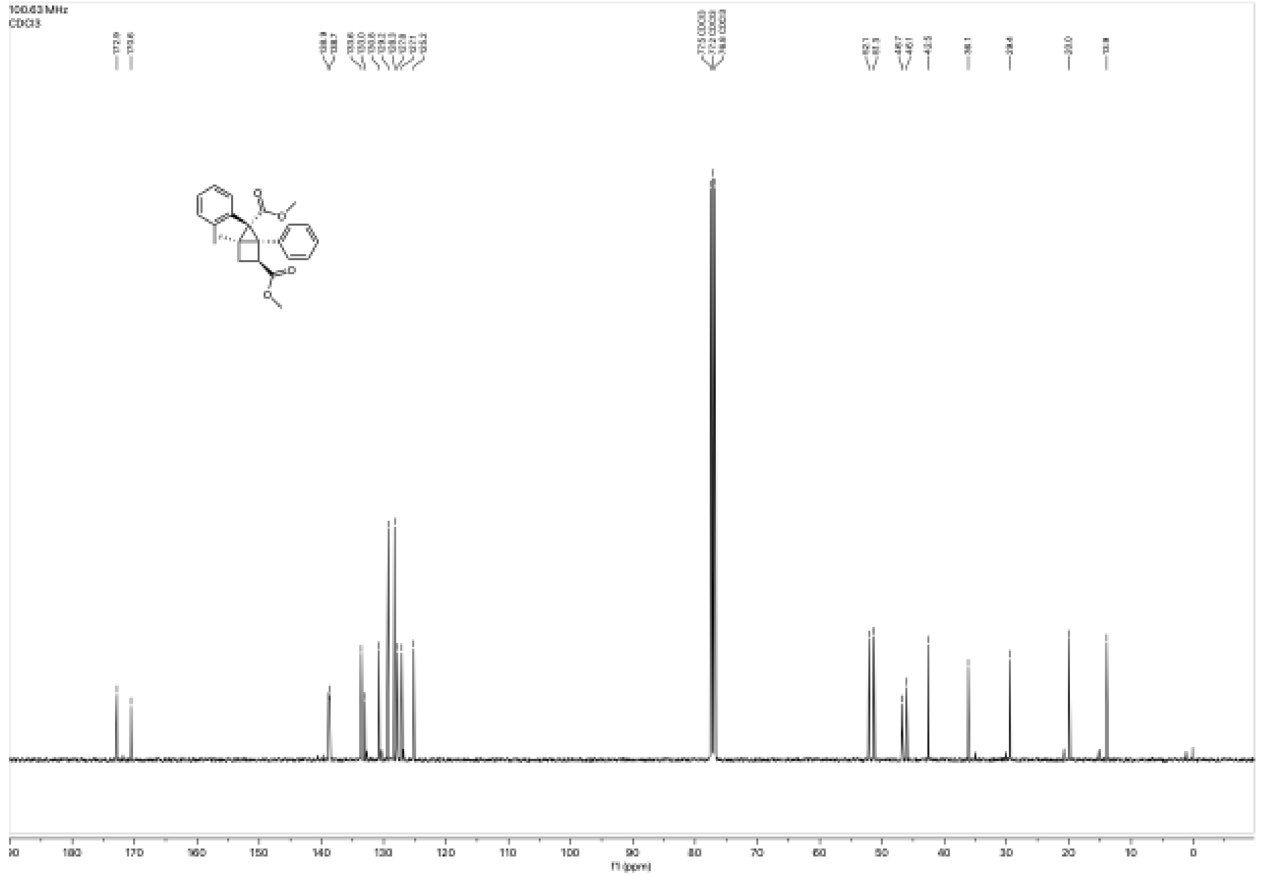

^{1}H NMR of **21c** (55 ºC)

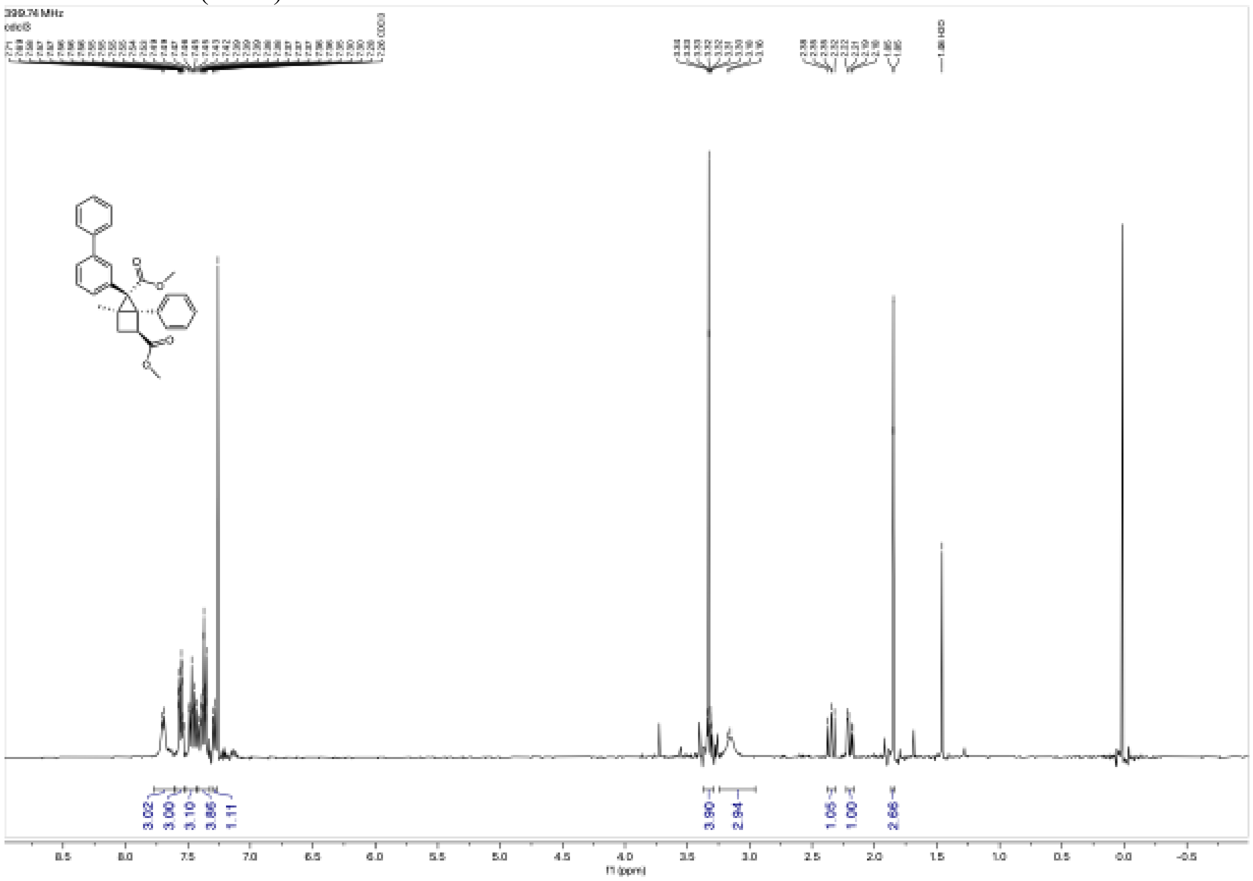

^{13}C NMR of **21c**

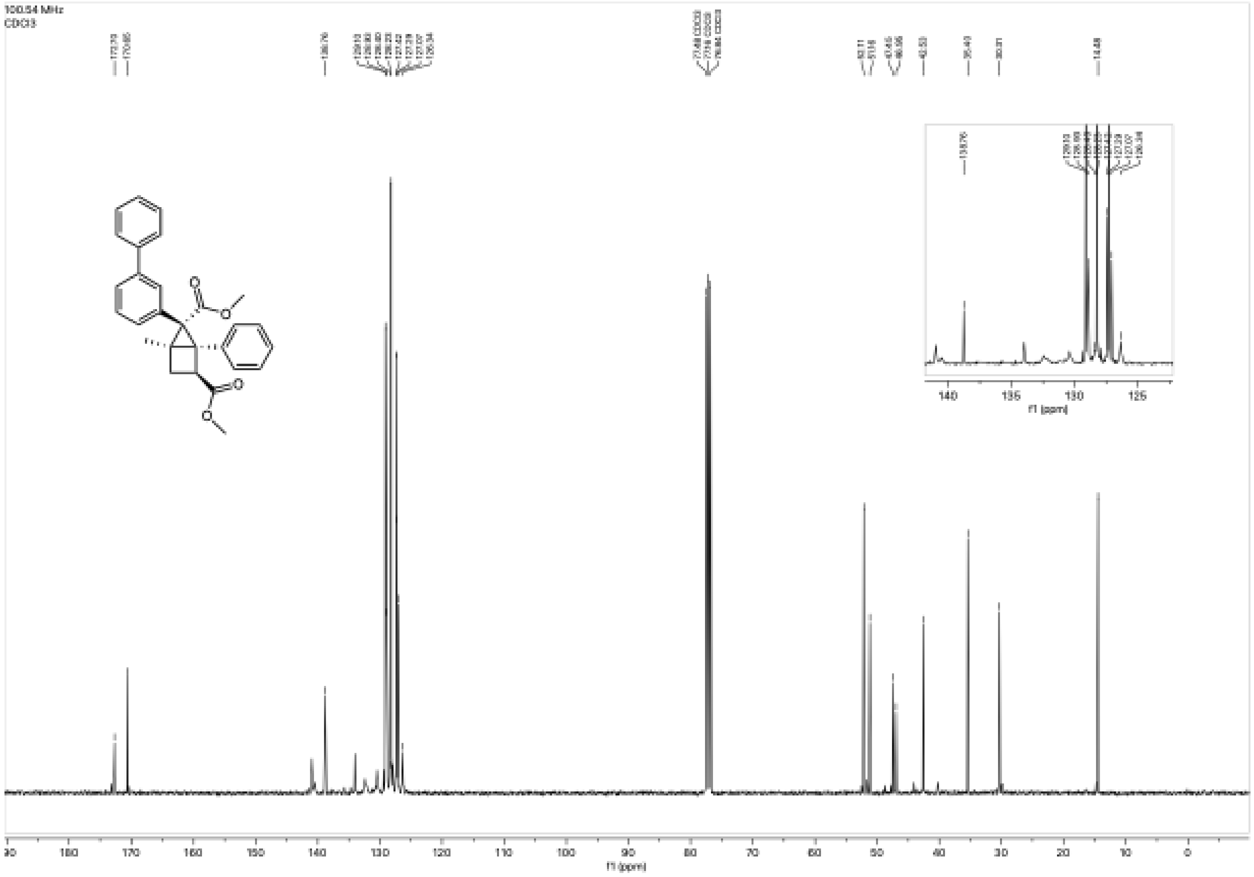

^{1}H NMR of **21d** (-50 ºC)

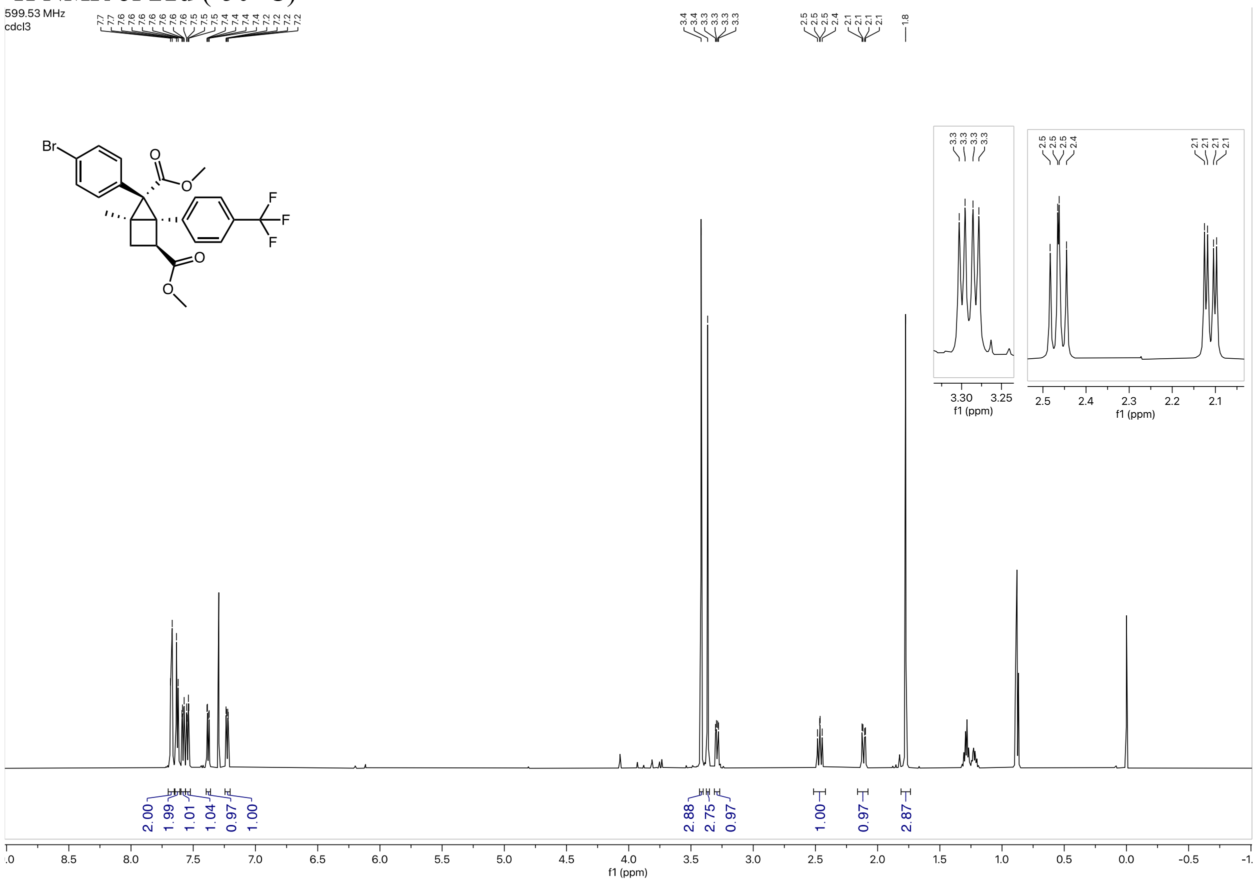

^{13}C NMR of **21d**

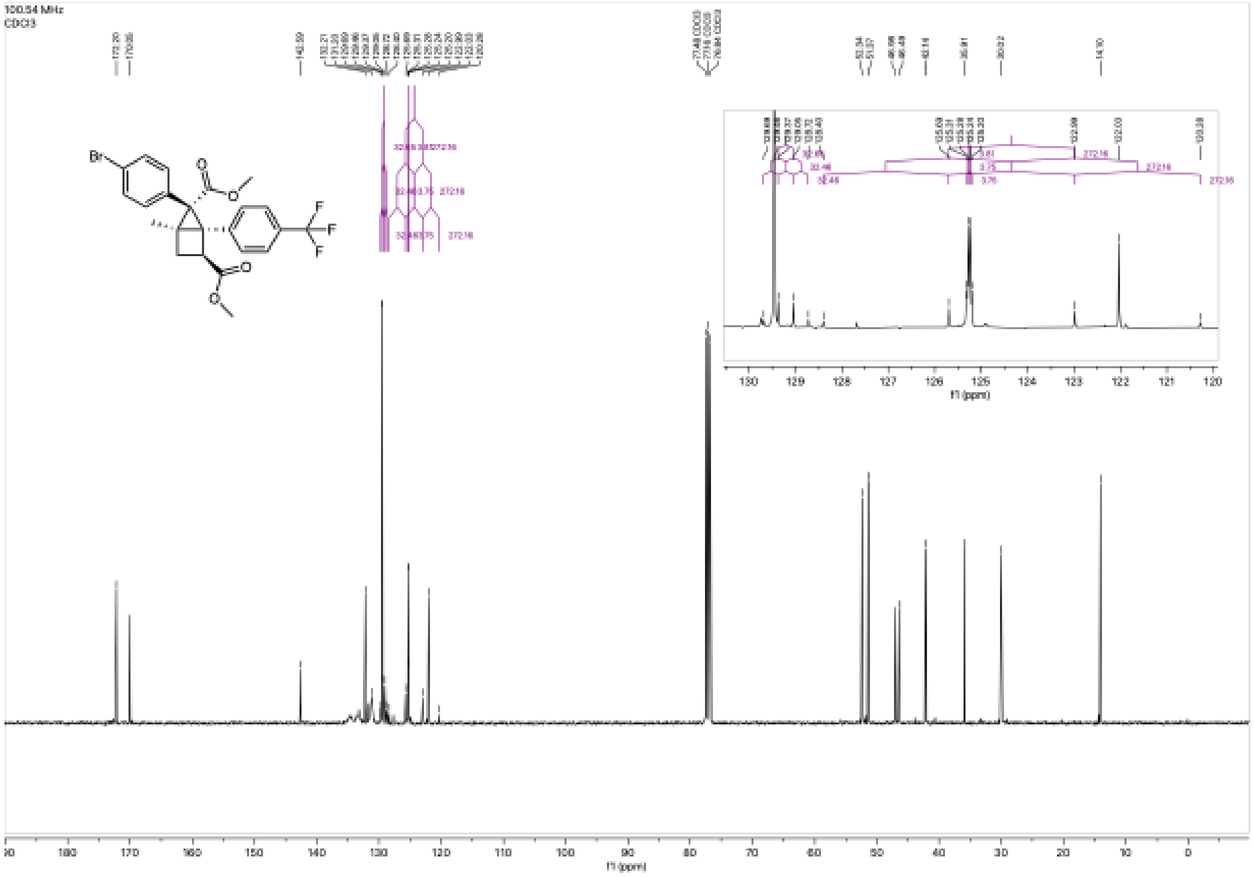

^{19}F NMR of **21d**

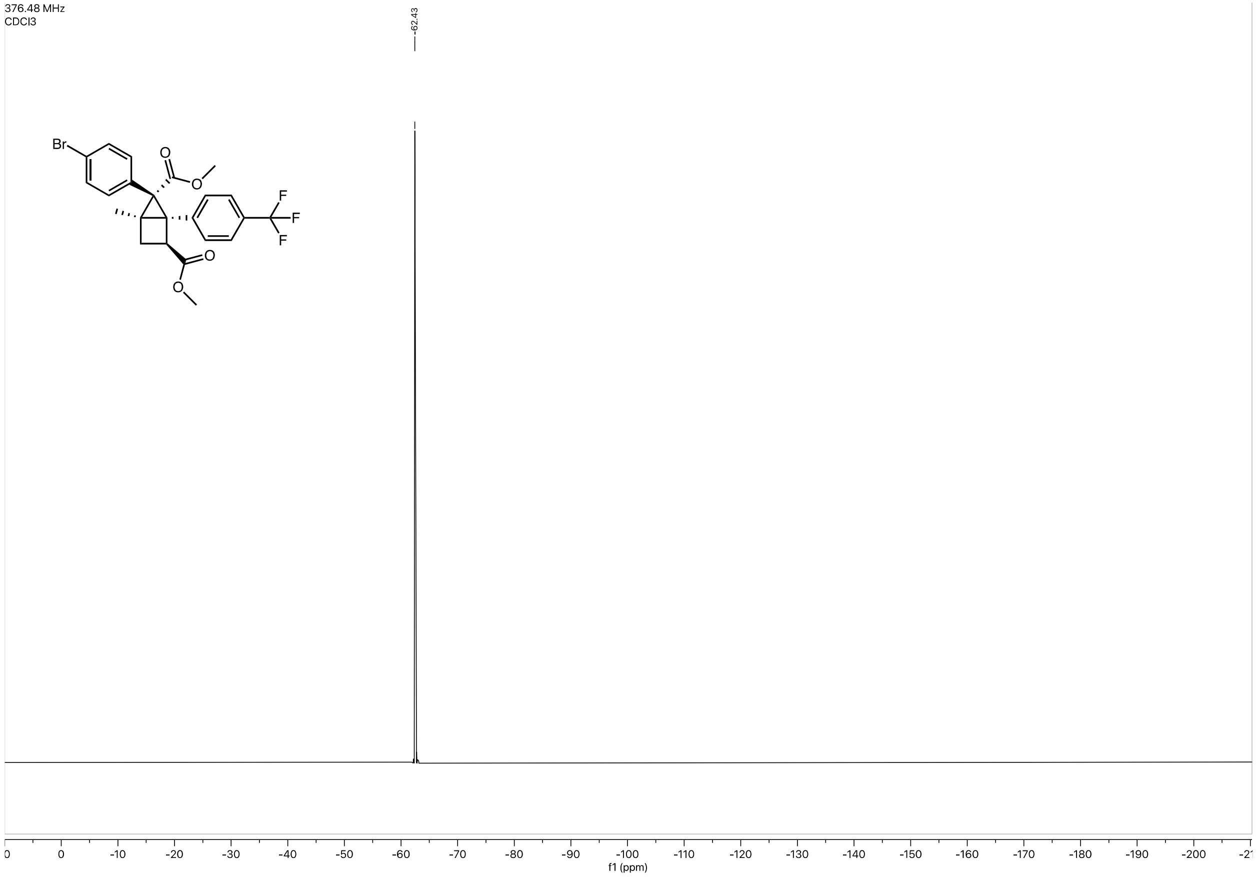

^{1}H NMR of **21e** (-20 ºC)

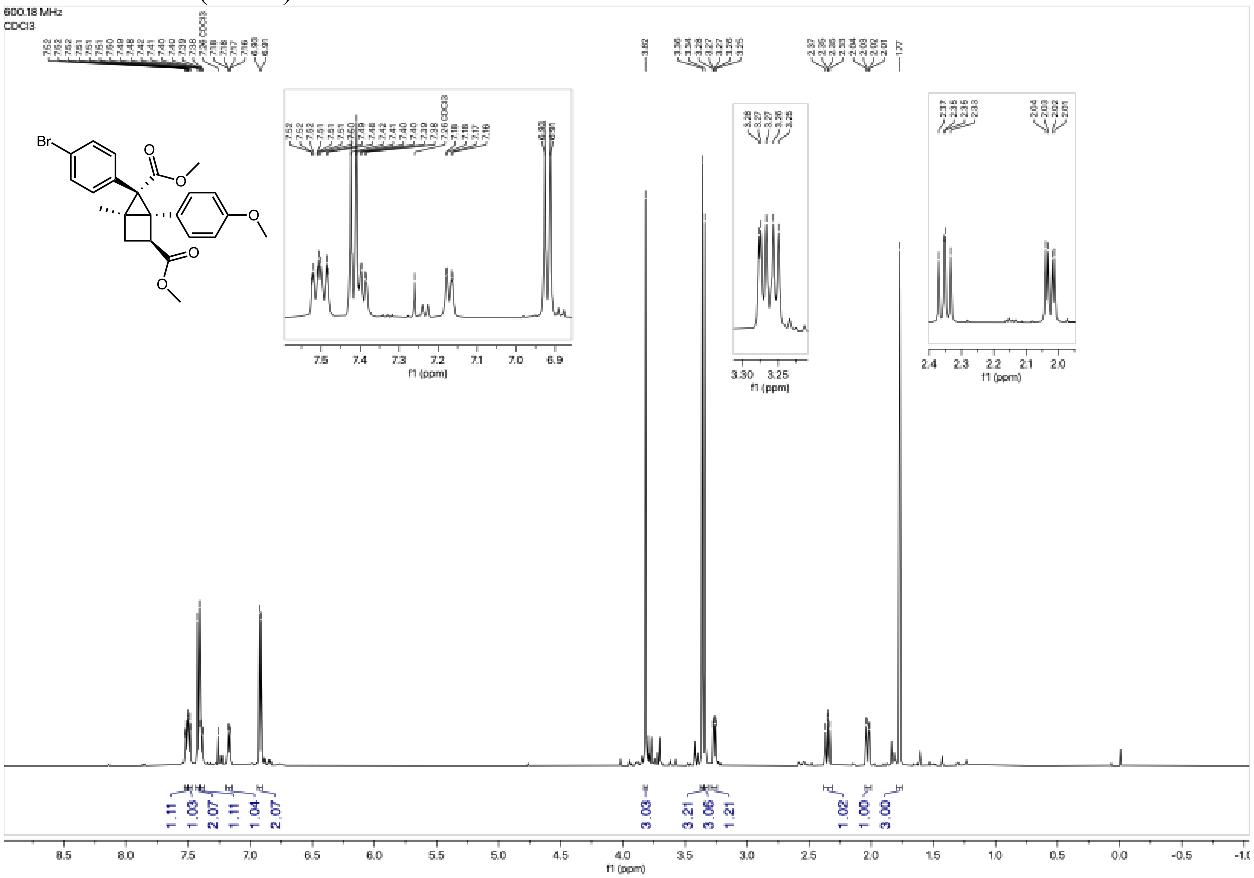

^{13}C NMR of **21e**

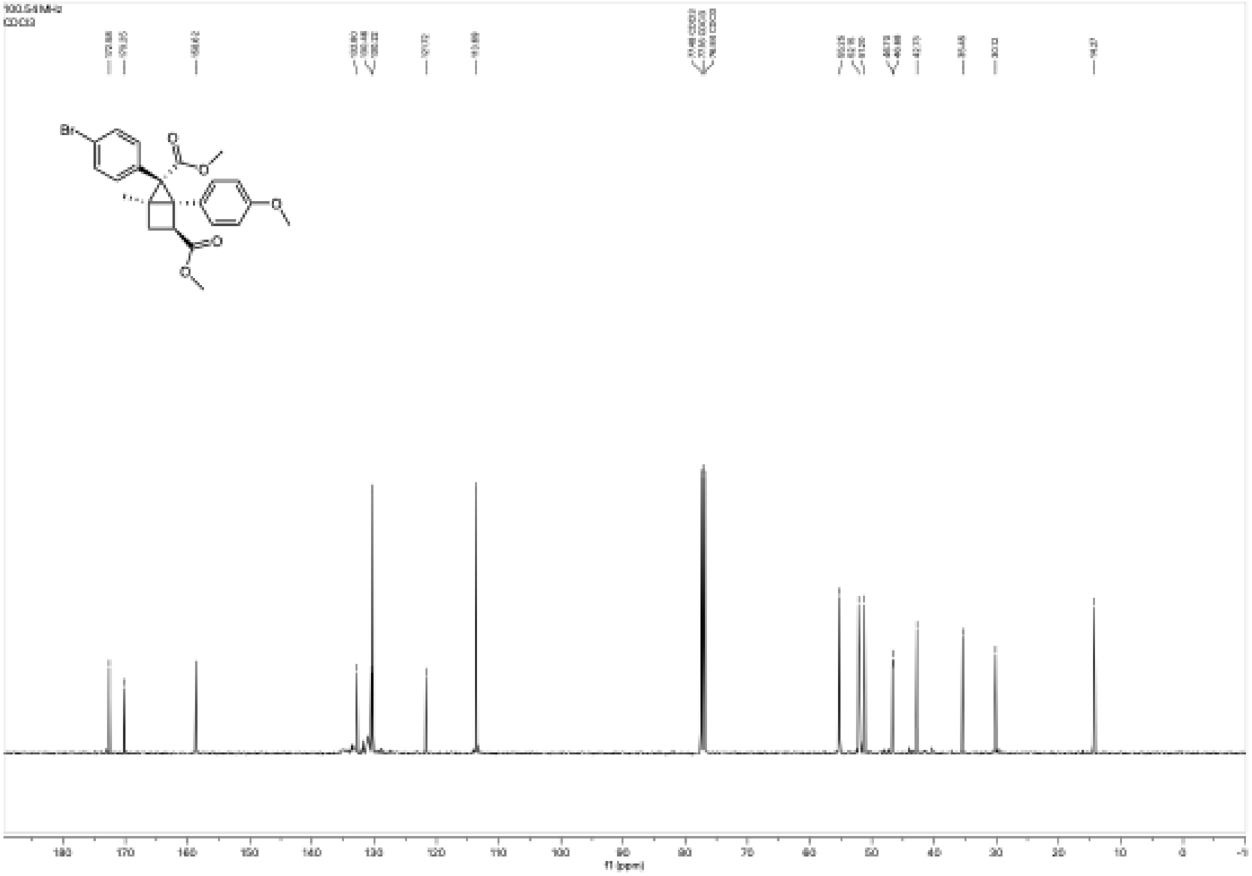

^{1}H NMR of **21f** (-20 ºC)

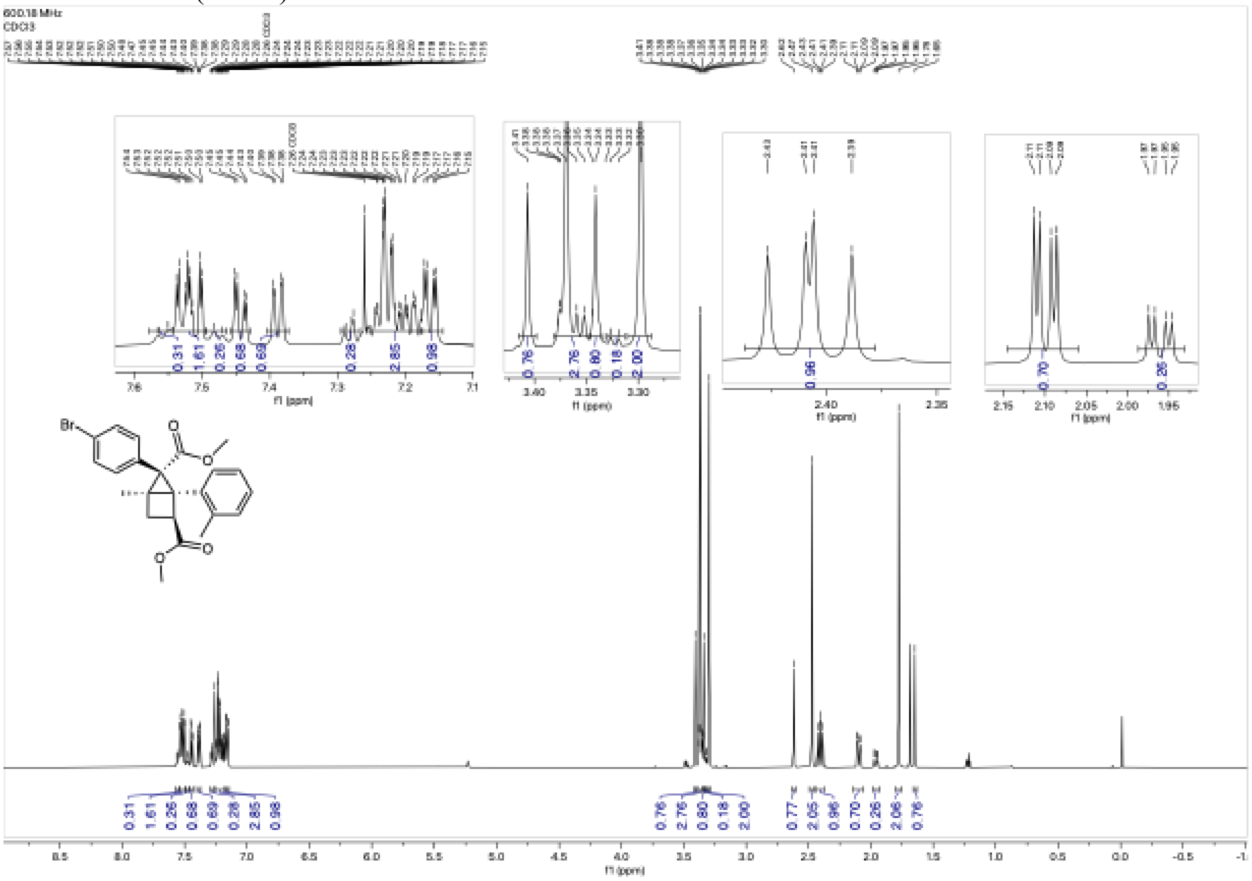

^{13}C NMR of **21f**

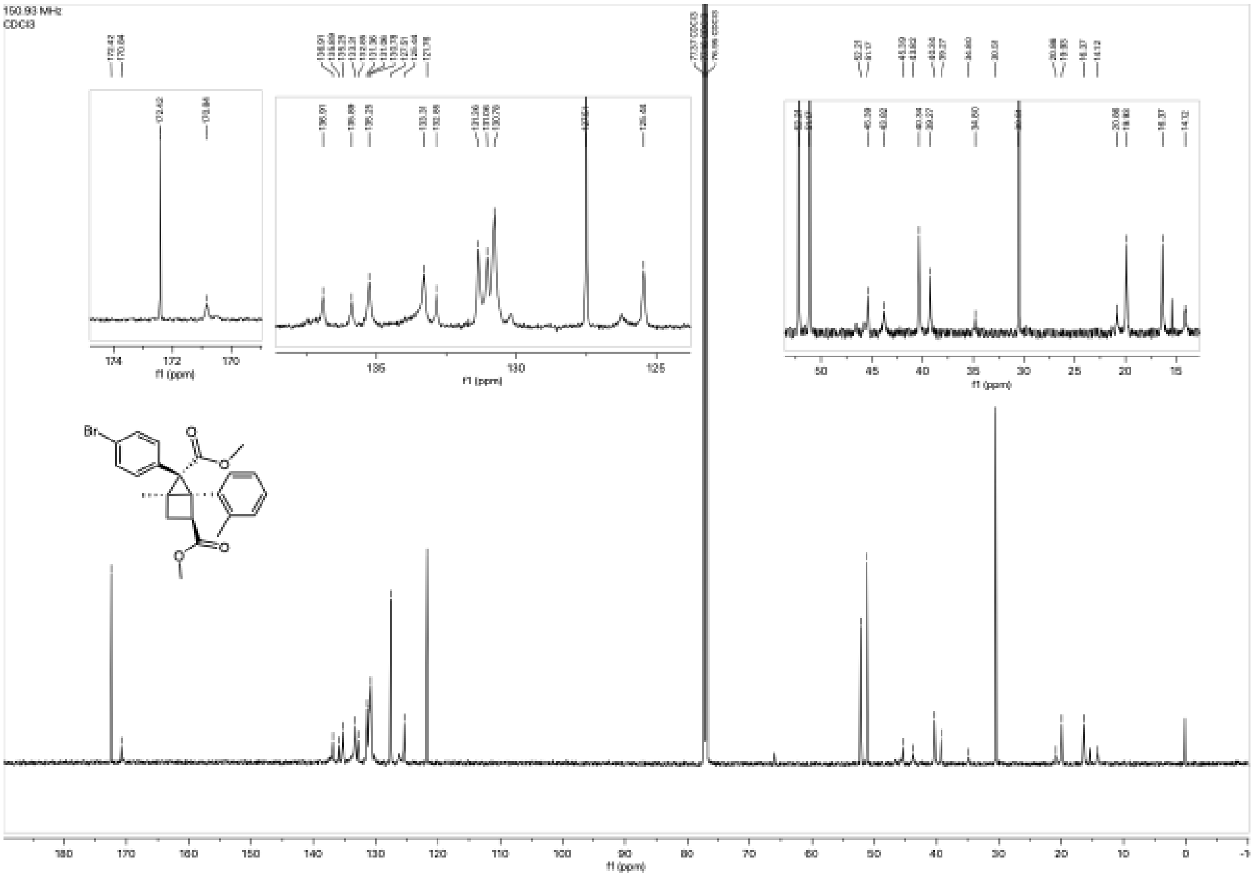

^{1}H NMR of **21g** (-35 ºC)

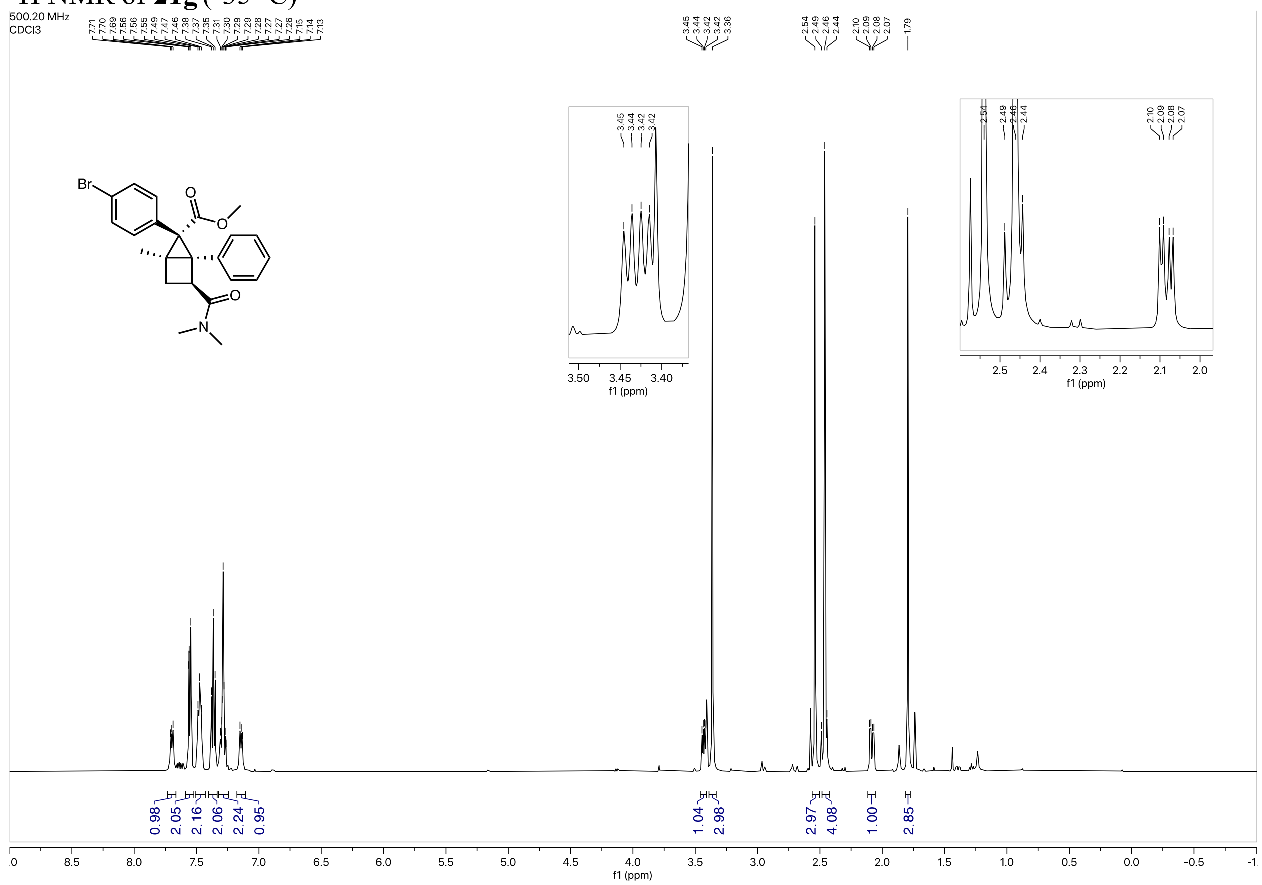

^{13}C NMR of **21g** (JMOD)

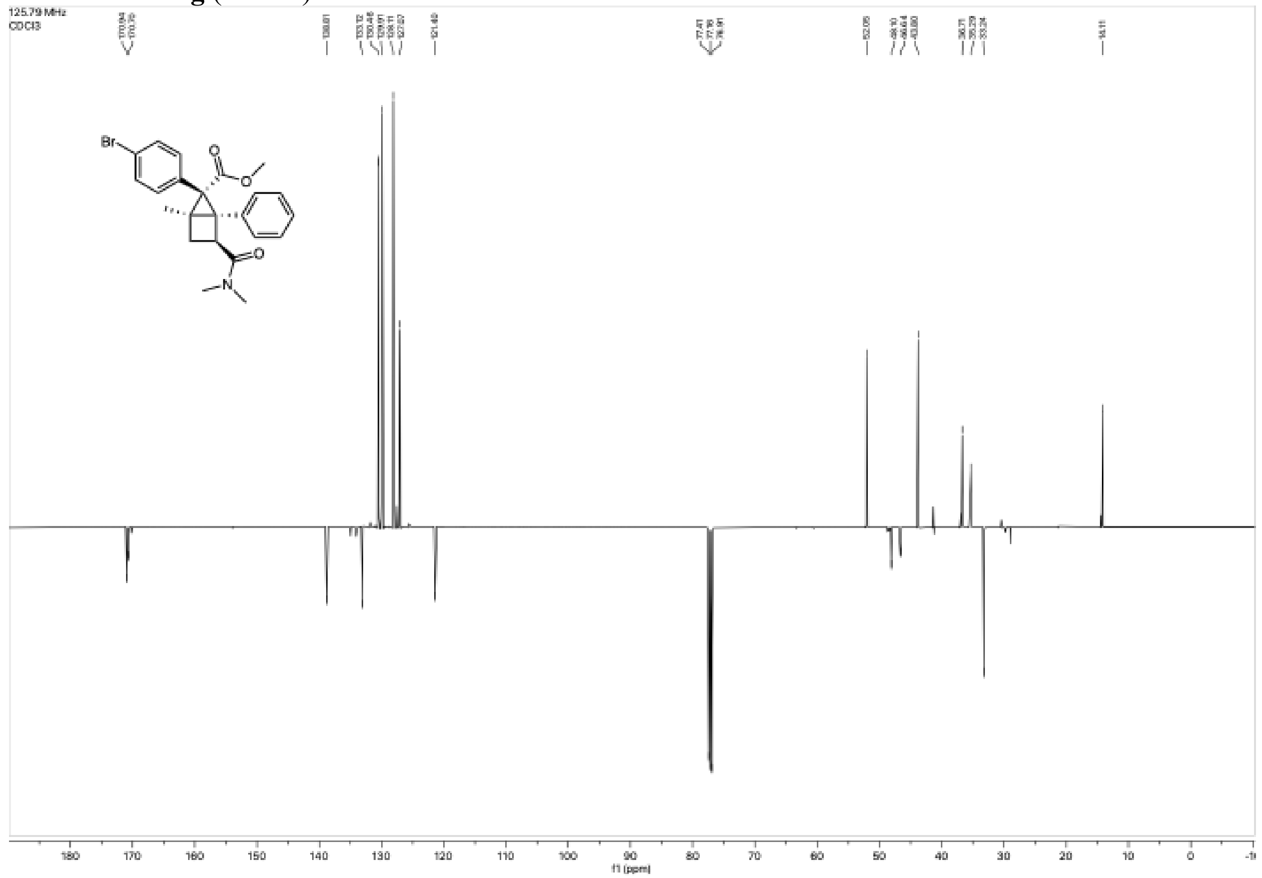

^{1}H NMR of **21h** (-35 ºC)

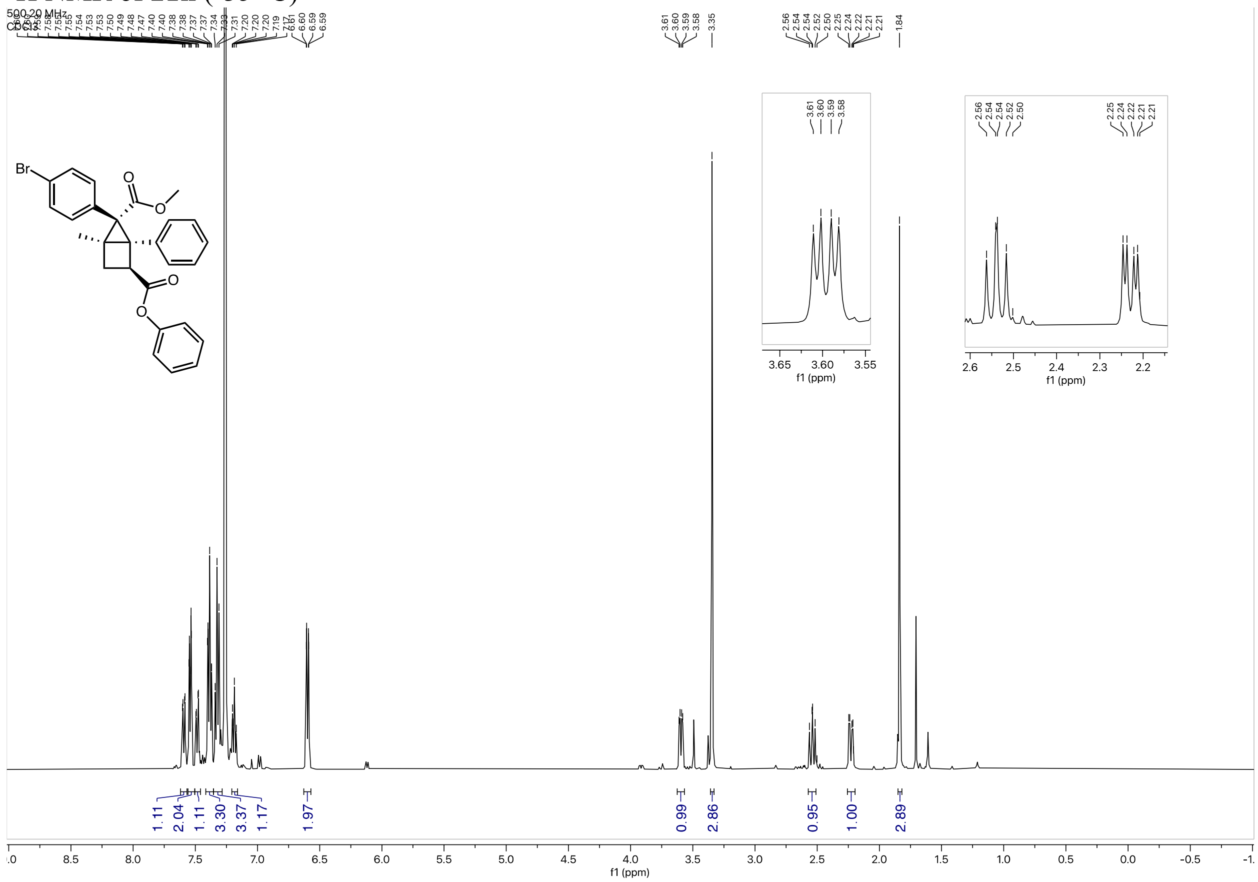

^{13}C NMR of **21h**

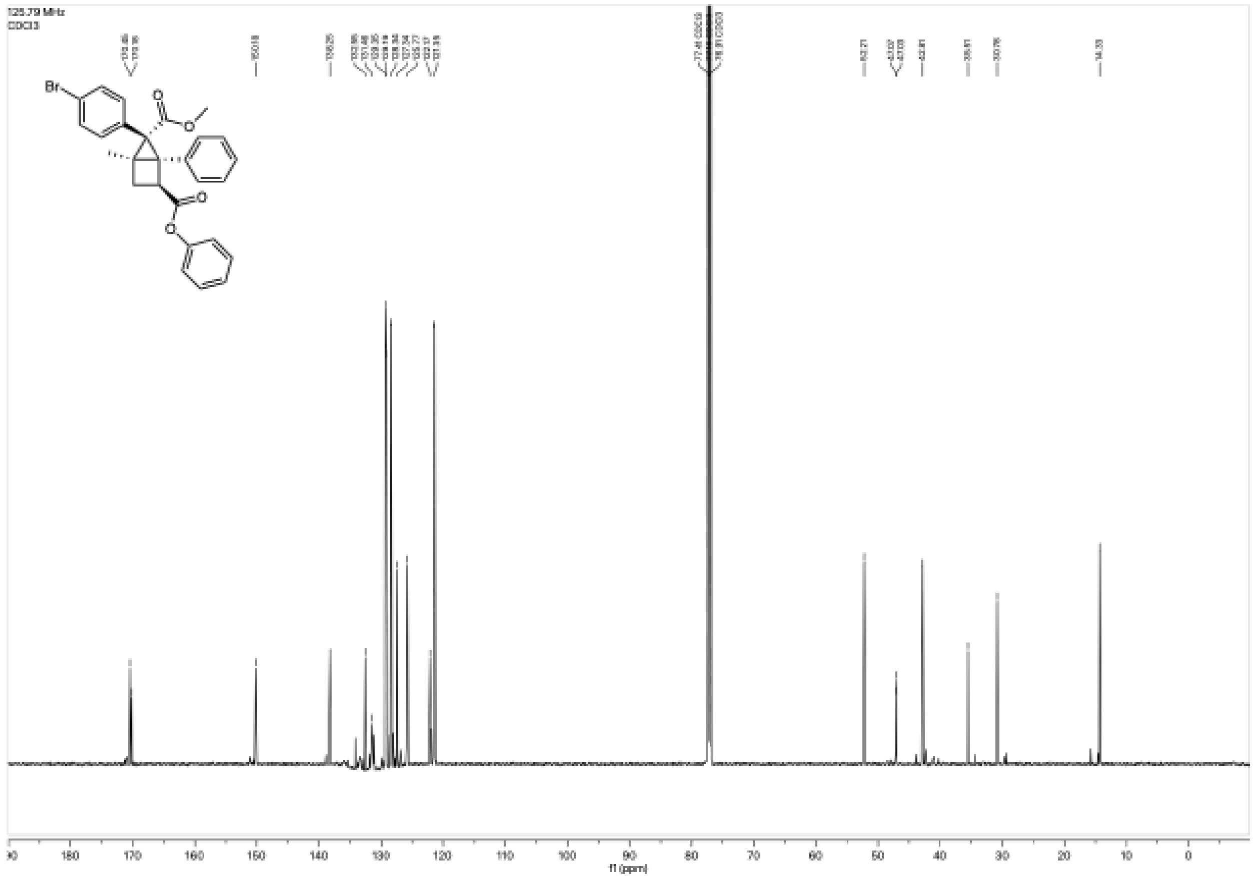

^{1}H NMR of **21i** (-35 ºC)

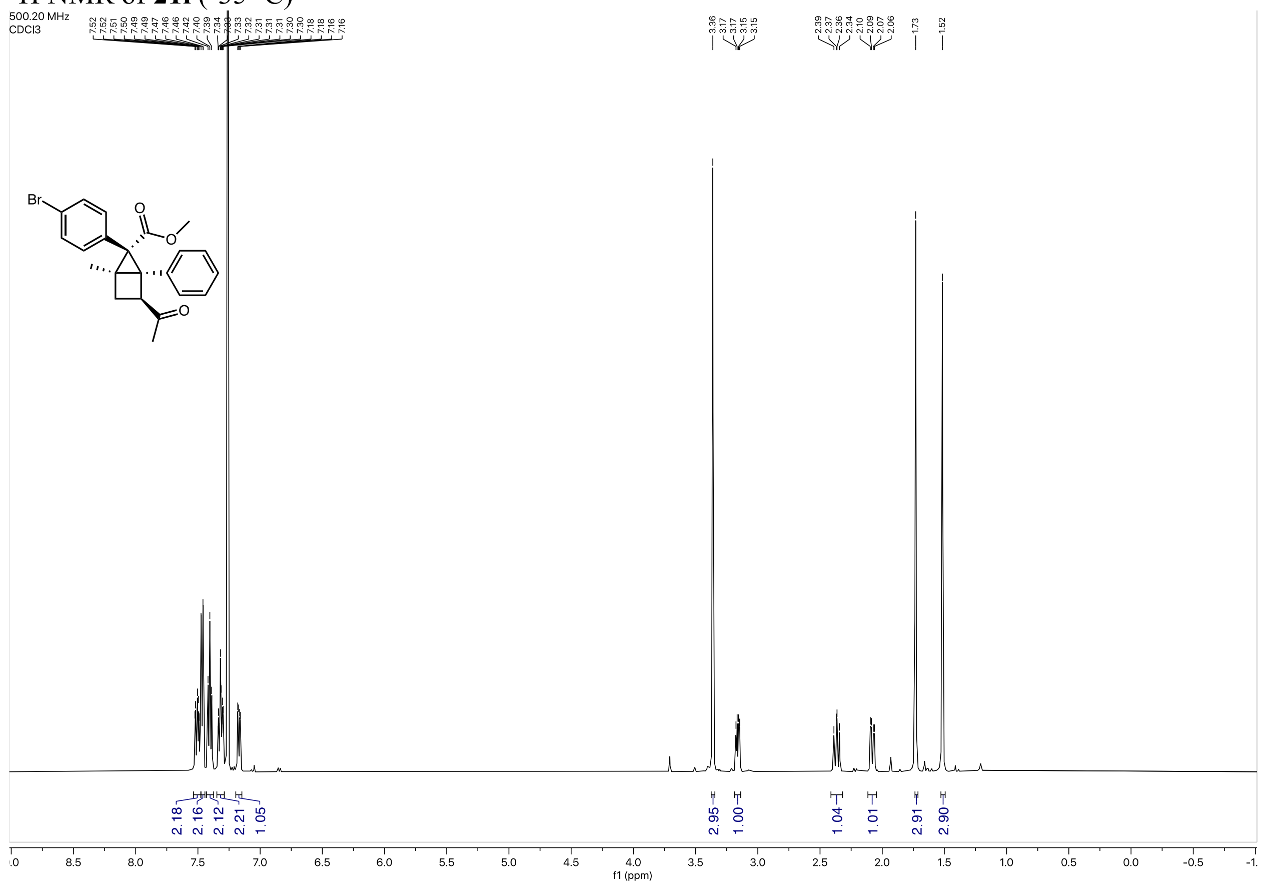

^{13}C NMR of **21i**

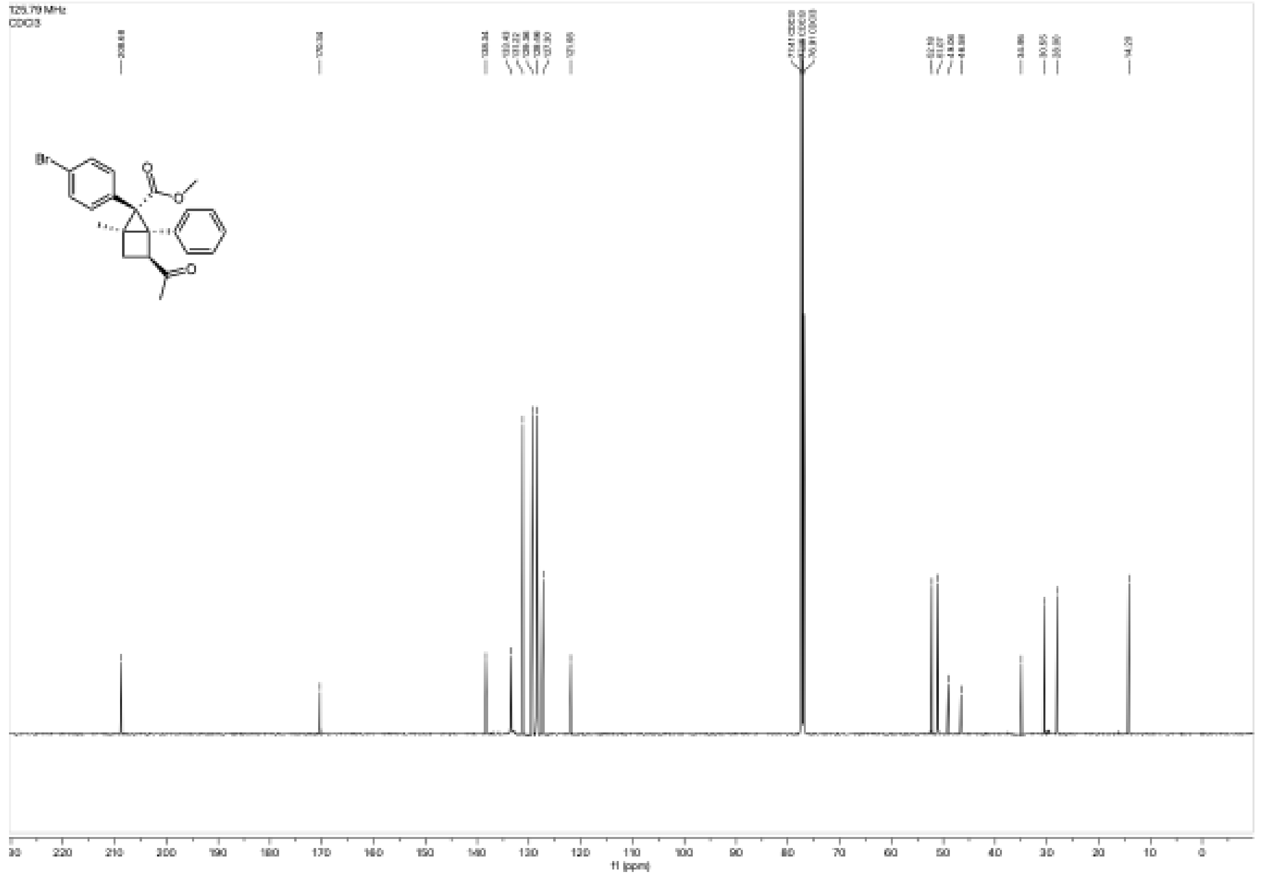

^{1}H NMR of **21j** major diastereomer (-35 ºC)

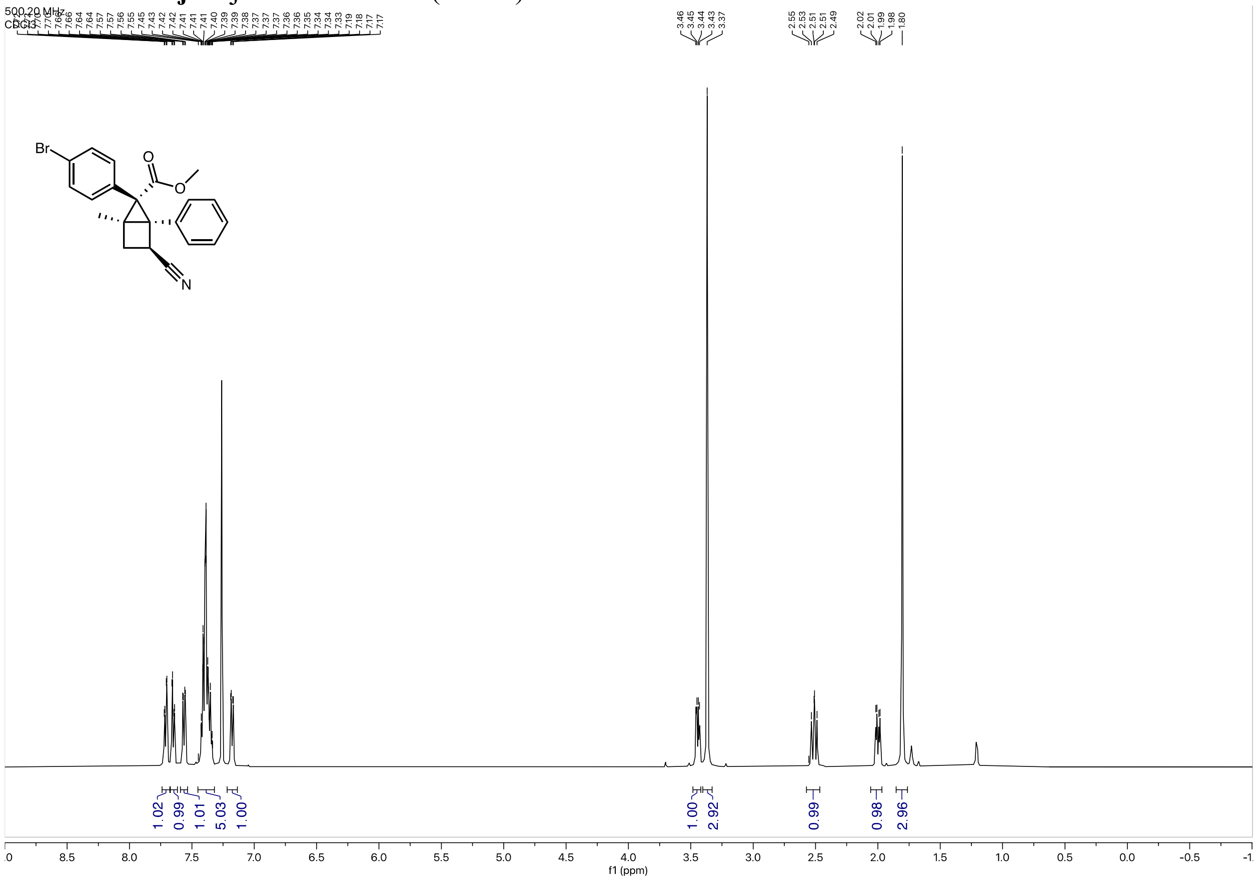

^{13}C NMR of **21j** major diastereomer

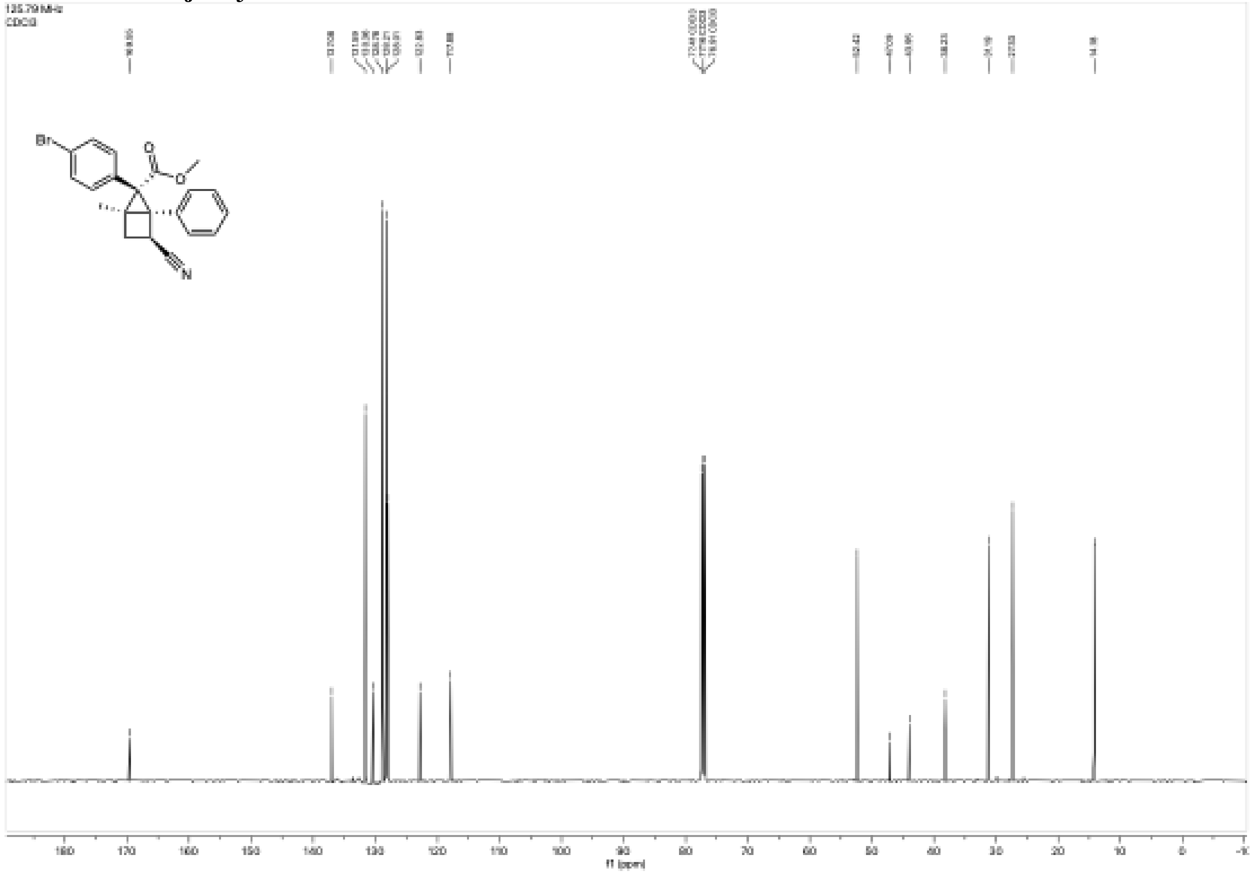

^{1}H NMR of **21j** minor diastereomer (-35 ºC)

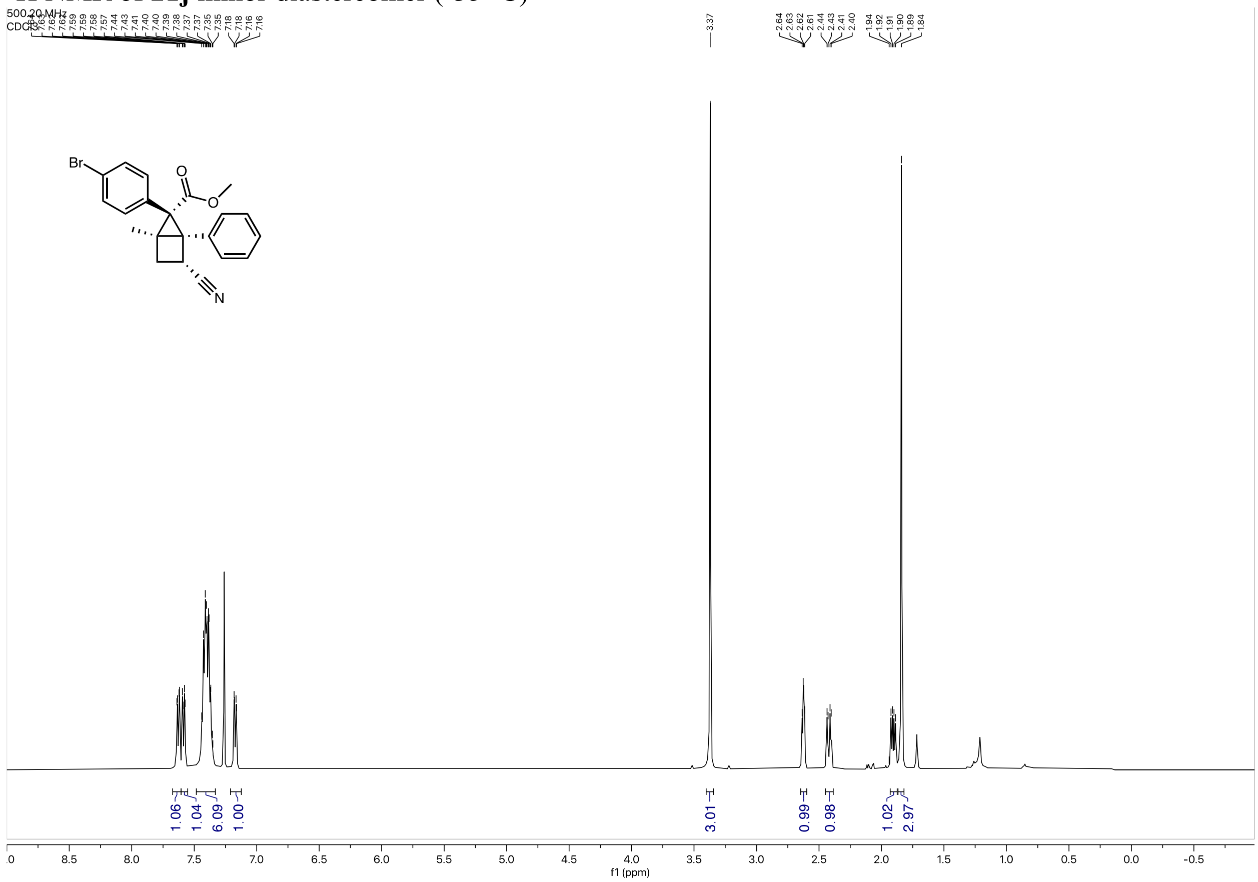

^{13}C NMR of **21j** minor diastereomer

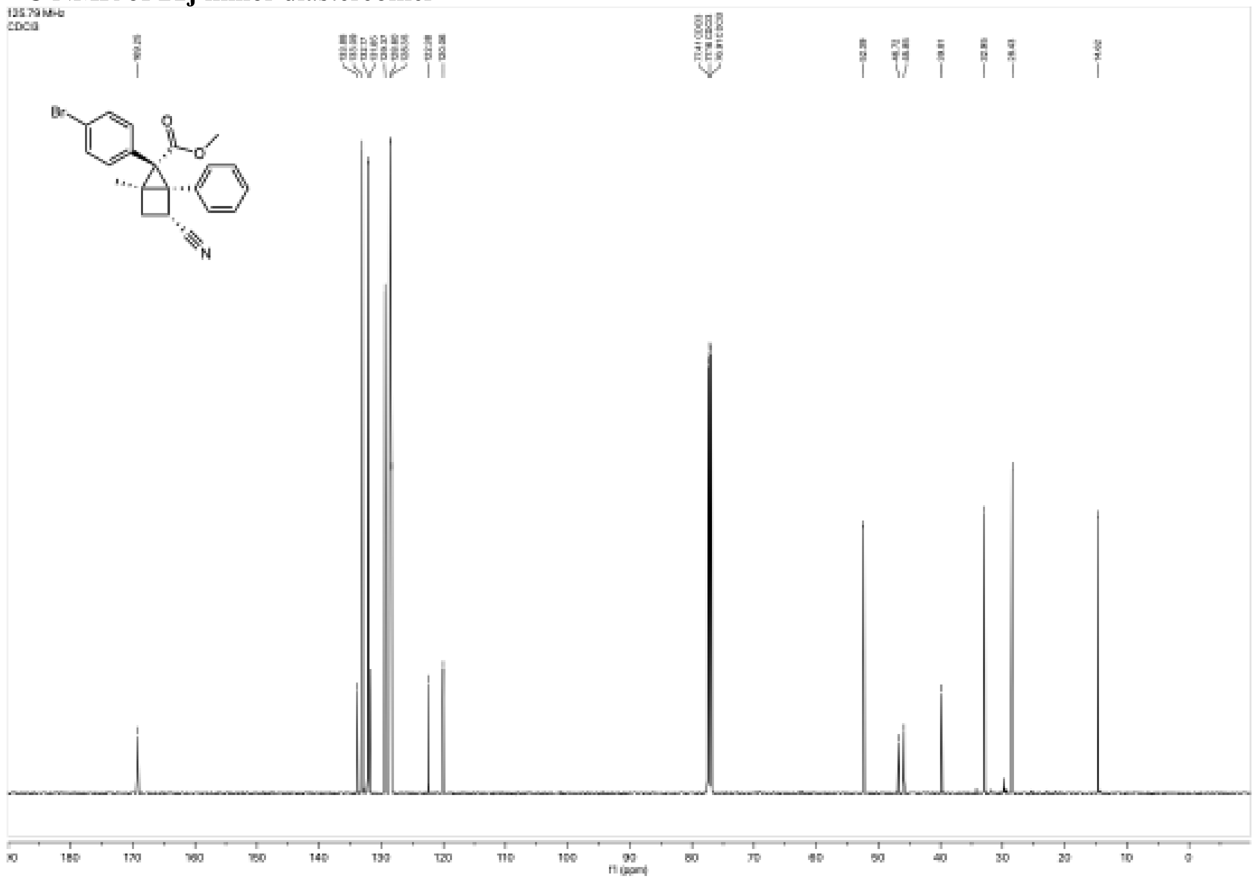

^{1}H NMR of **21k** (-35 ºC)

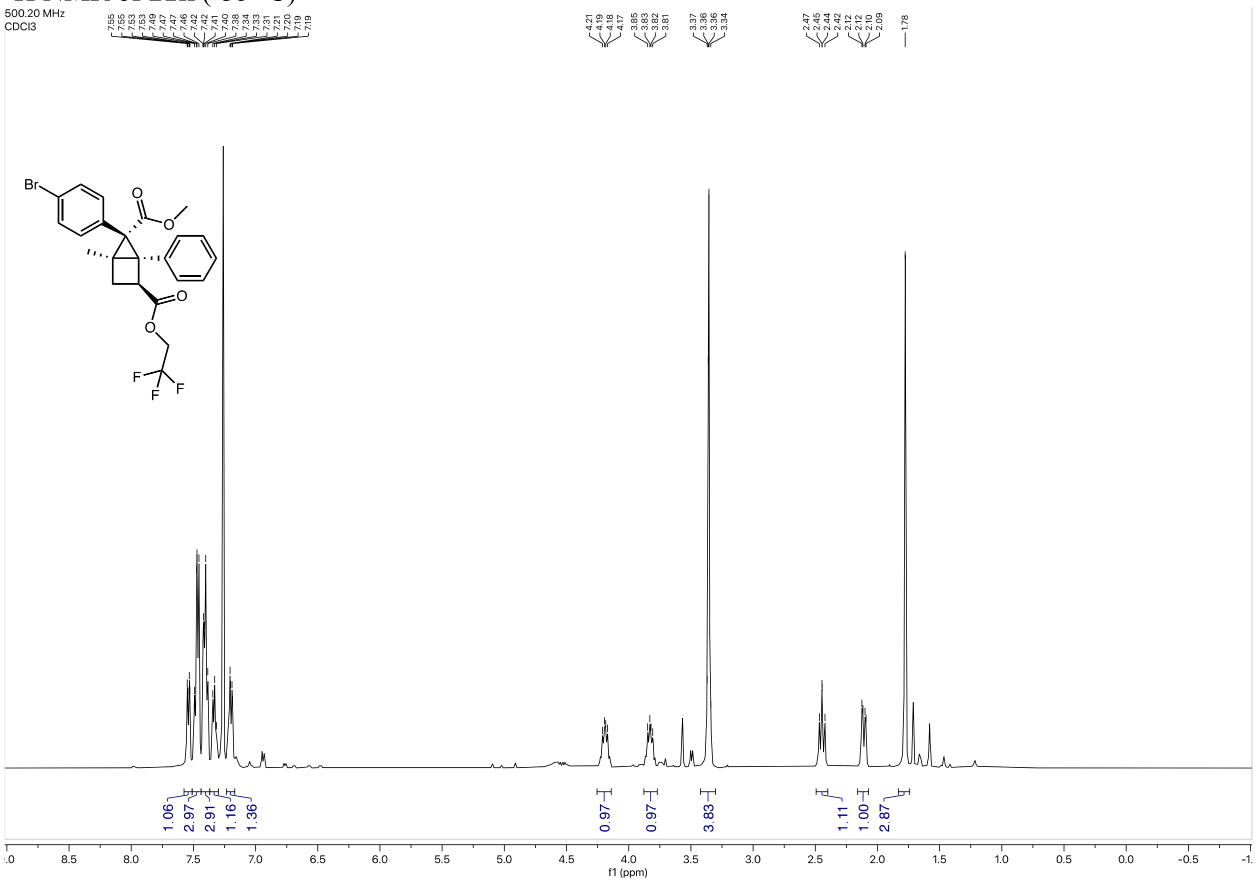

^{13}C NMR of **21k** (-35 ºC)

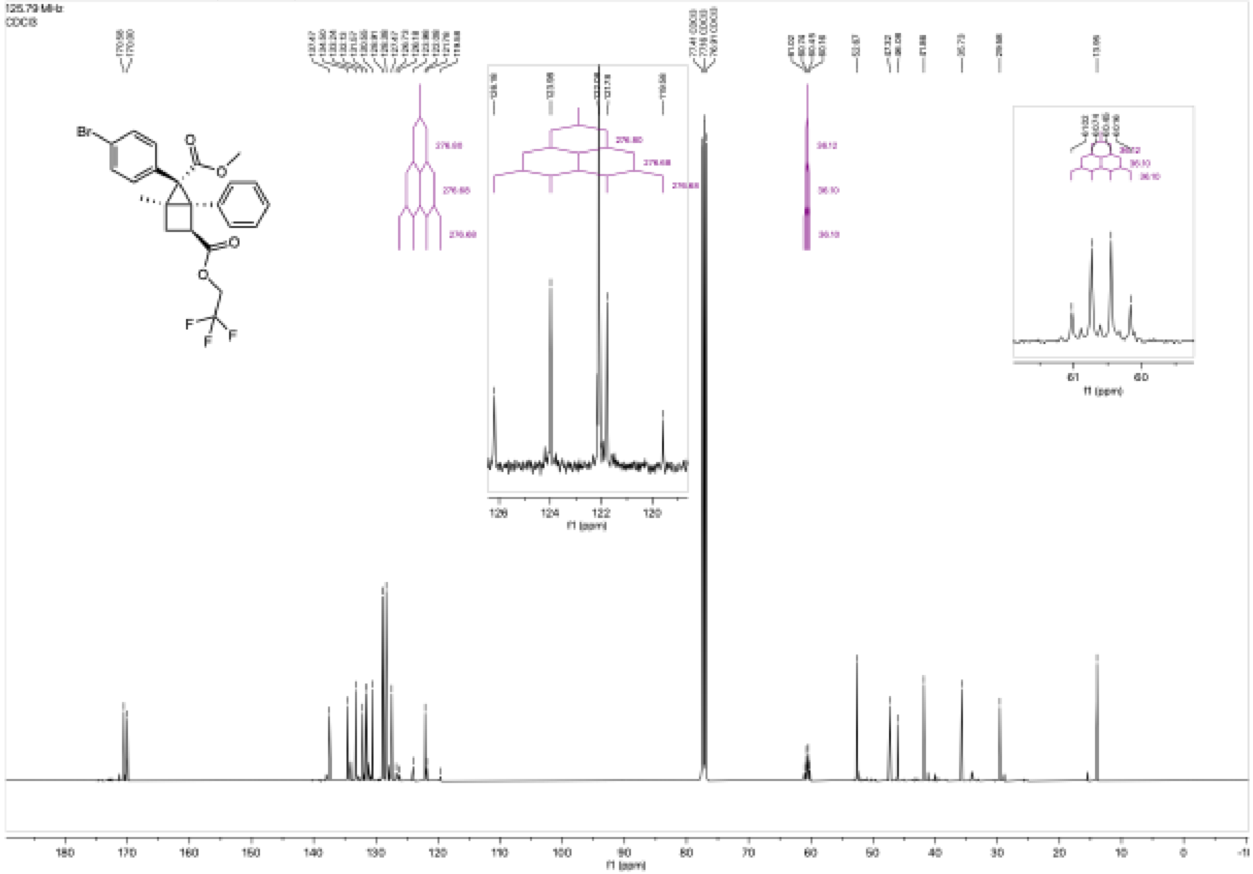

^{19}F NMR of **21k**

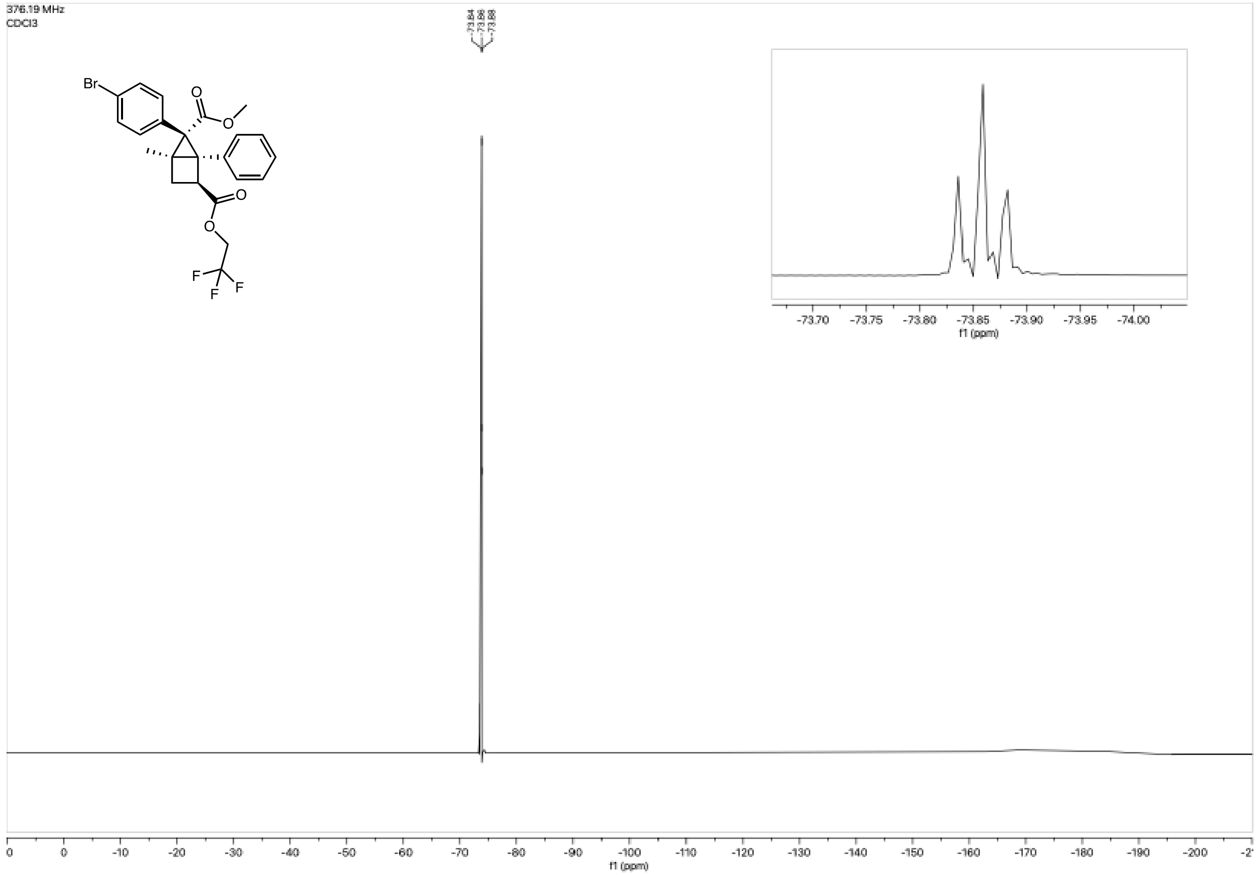

^{1}H NMR of **21l** (-35 ºC)

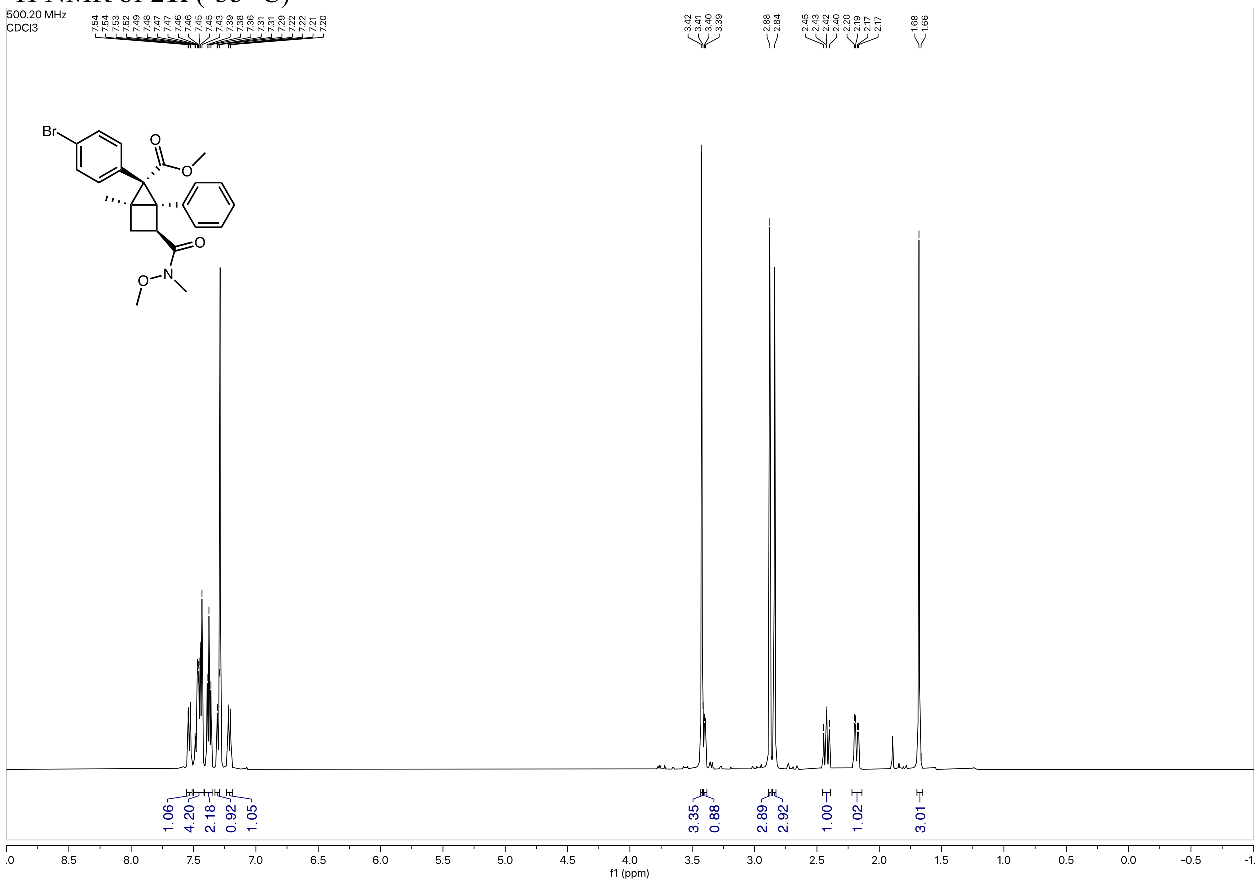

^{13}C NMR of **21l** (-35 ºC)

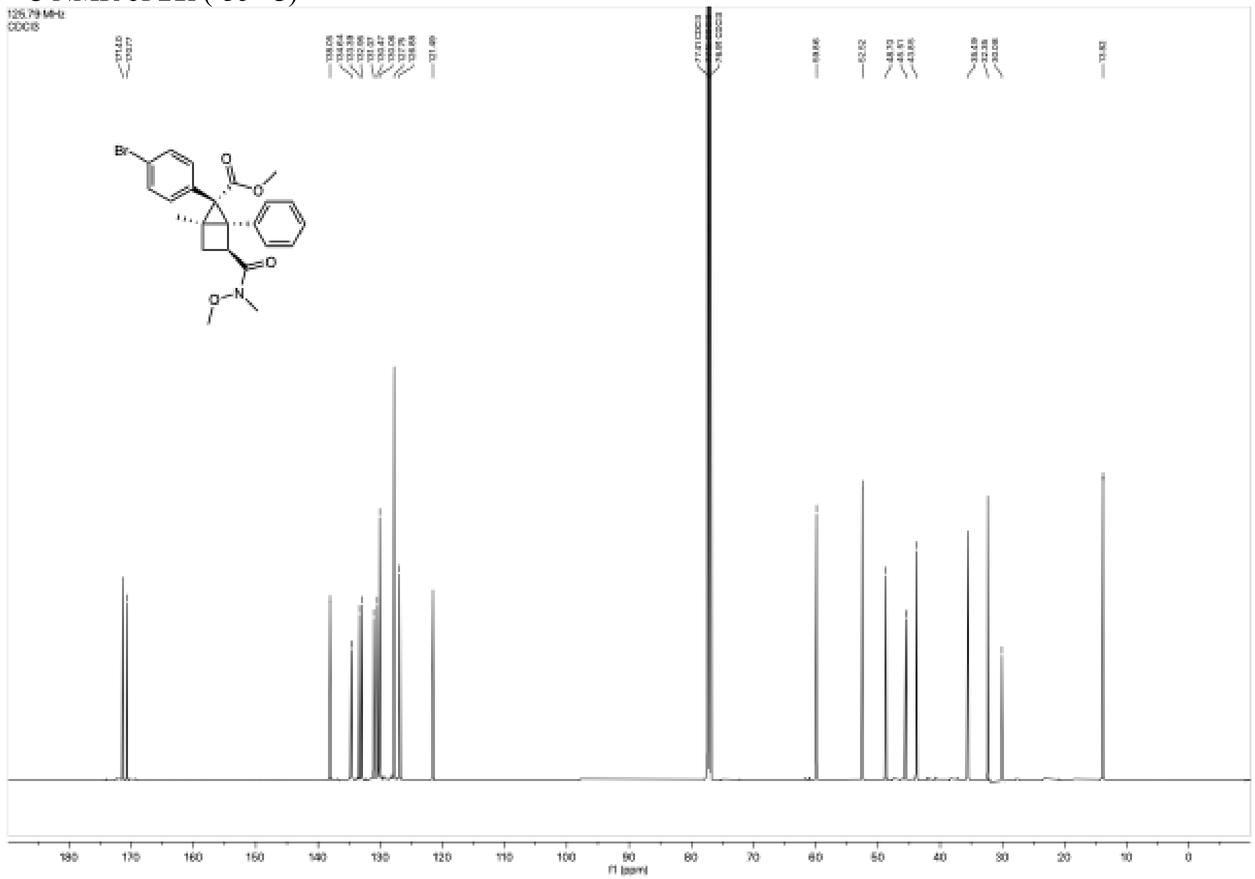

^{1}H NMR of **23a** (-50 ºC)

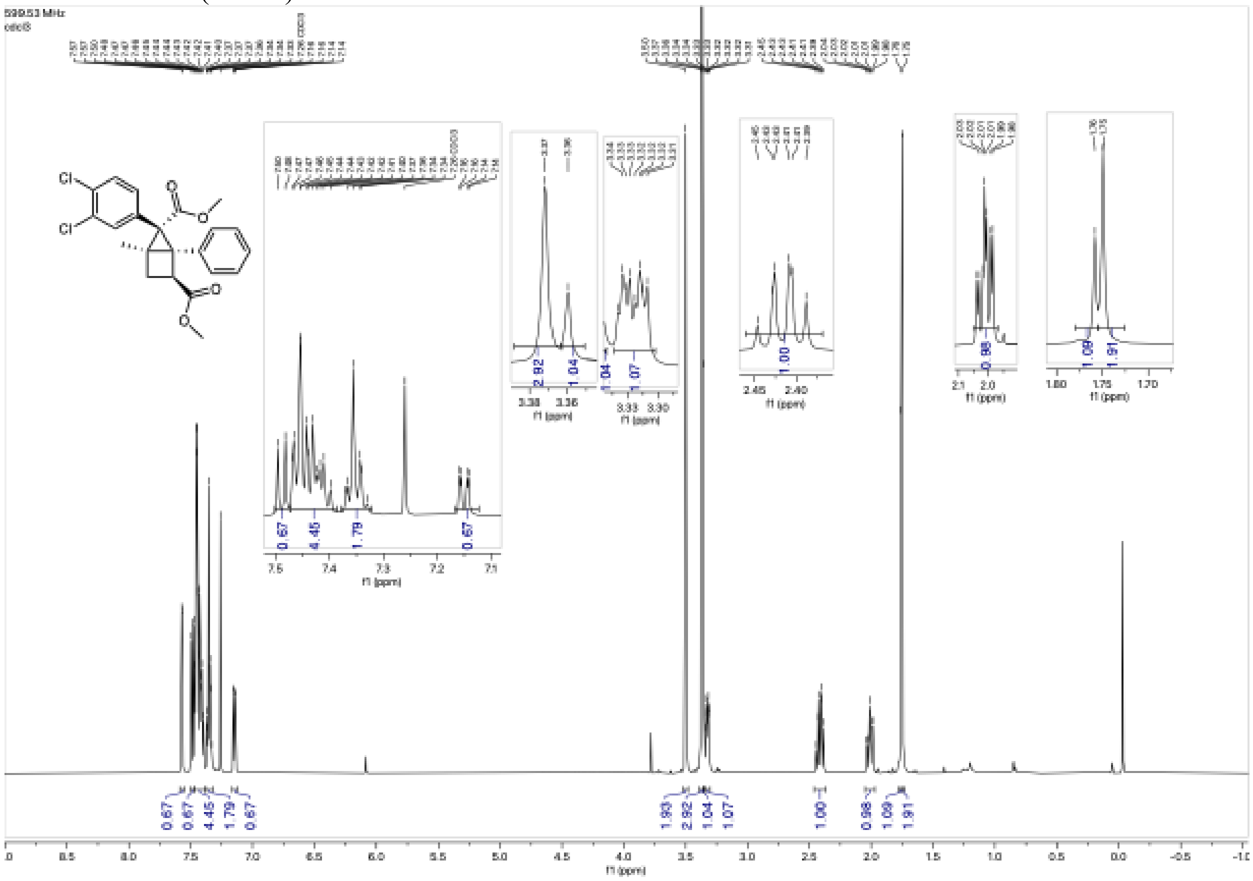

^{13}C NMR of **23a**

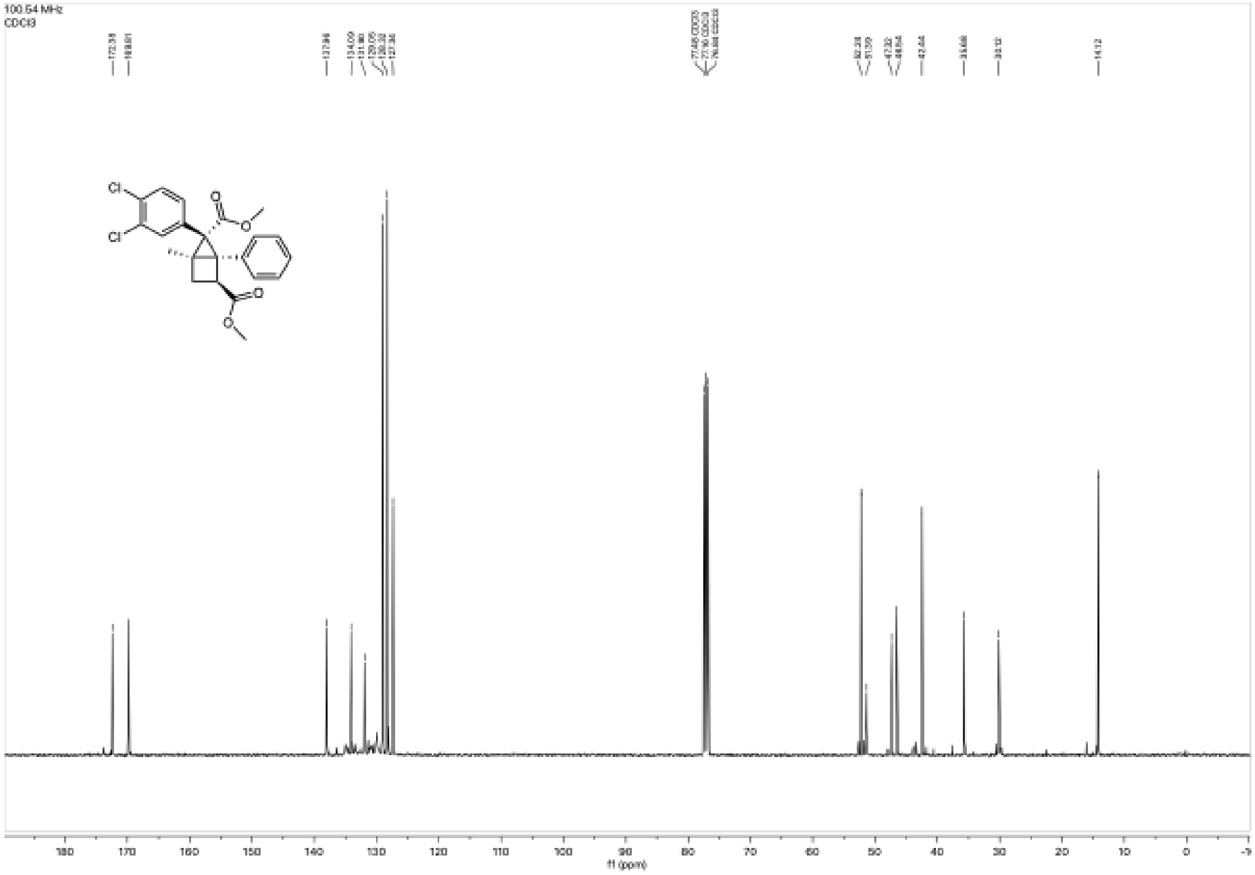

^{1}H NMR of **23b** (-50 ºC)

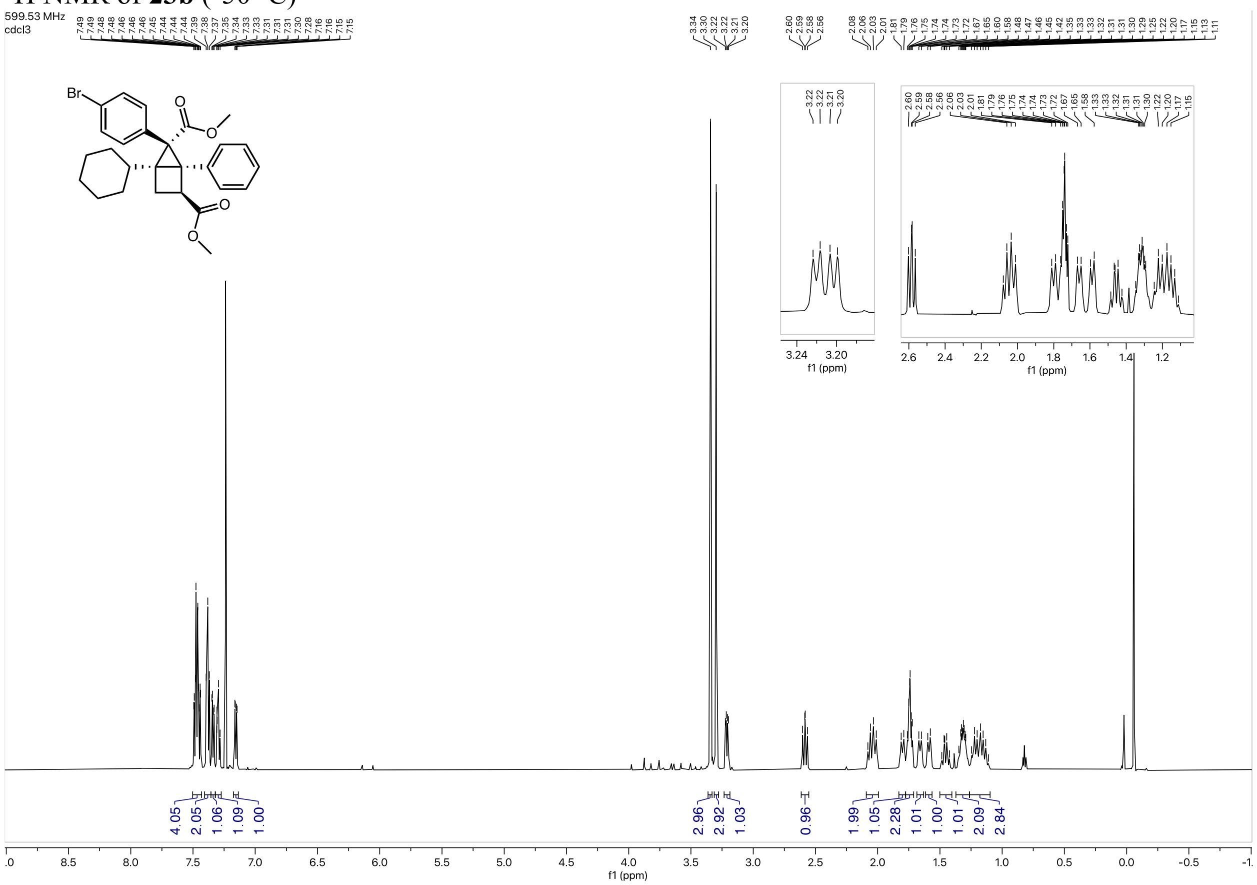

^{13}C NMR of **23b**

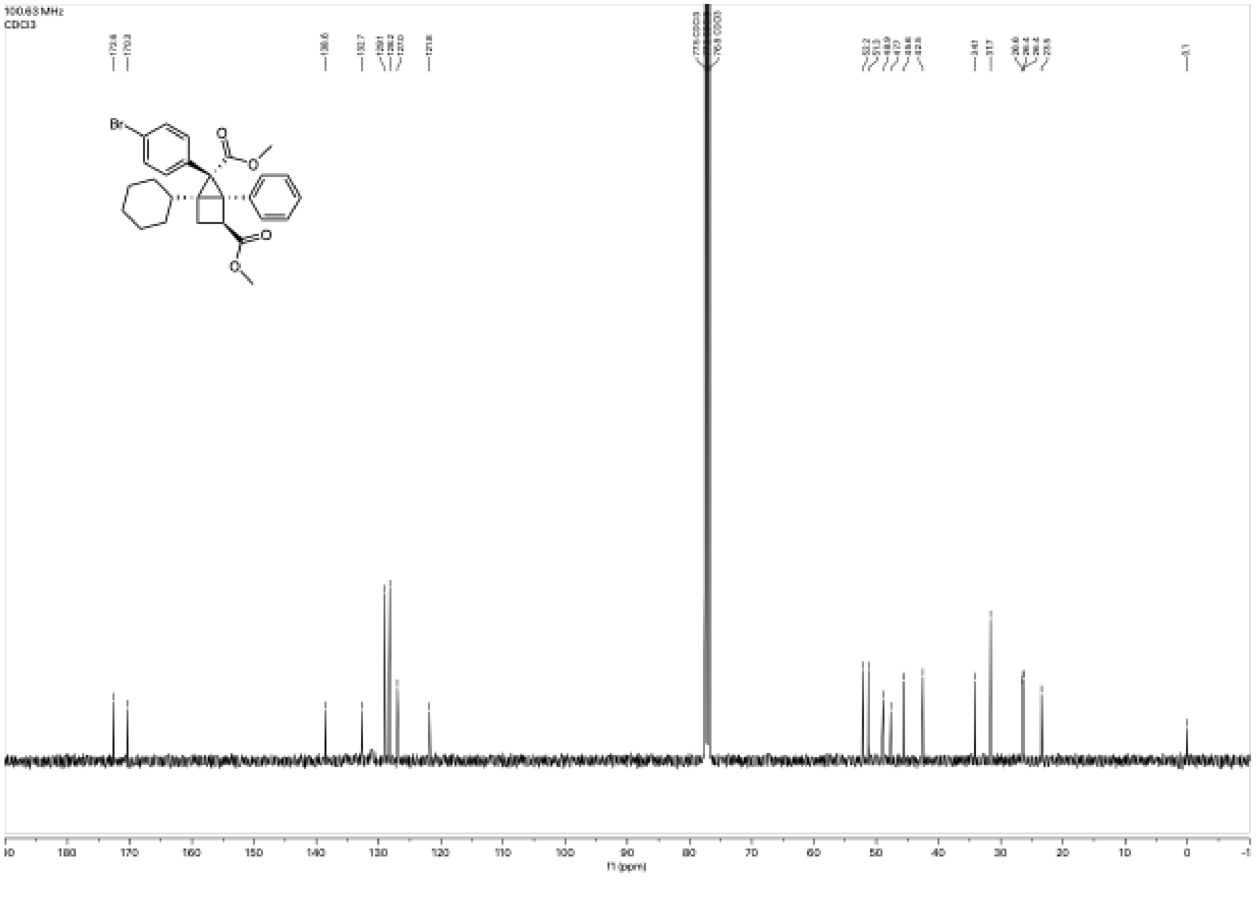

^{1}H NMR of **23c** (-50 ºC)

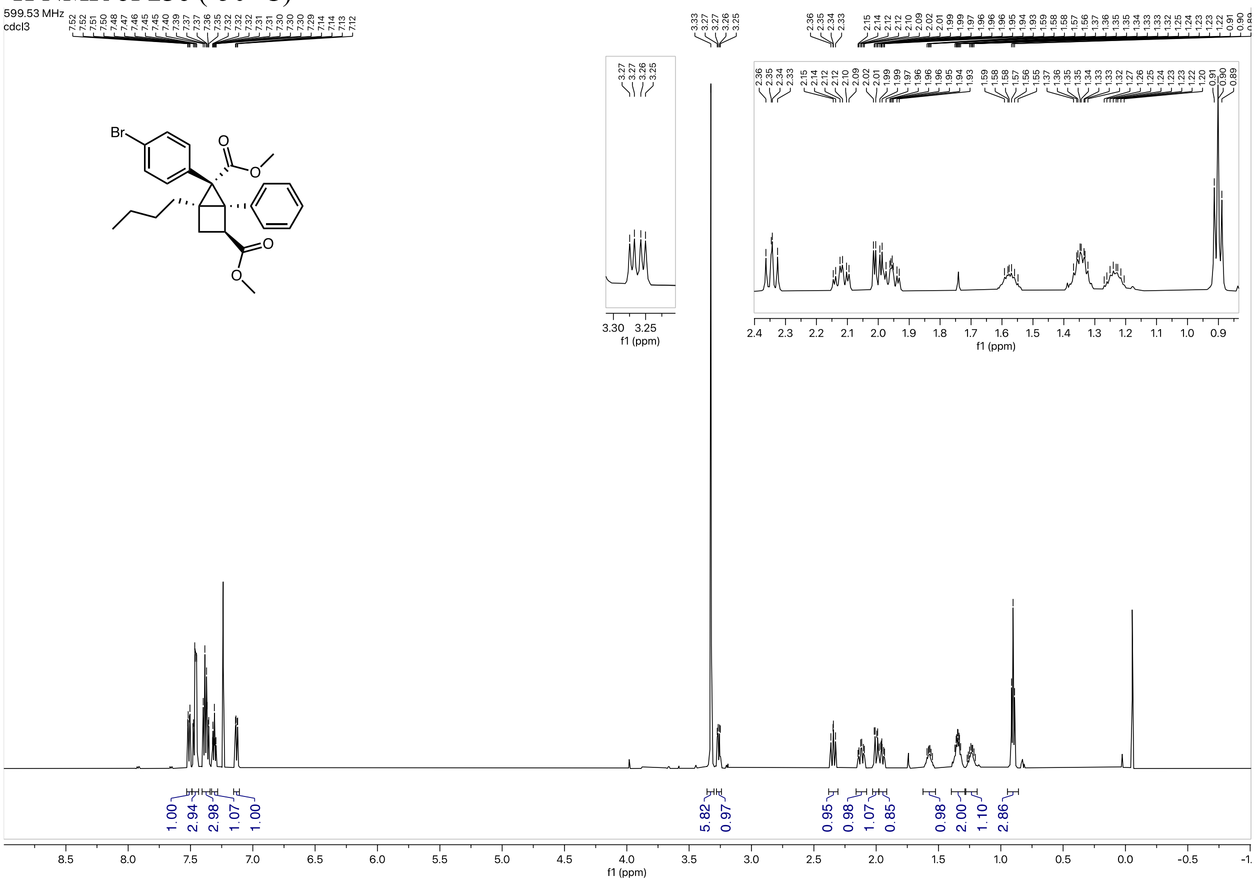

^{13}C NMR of **23c**

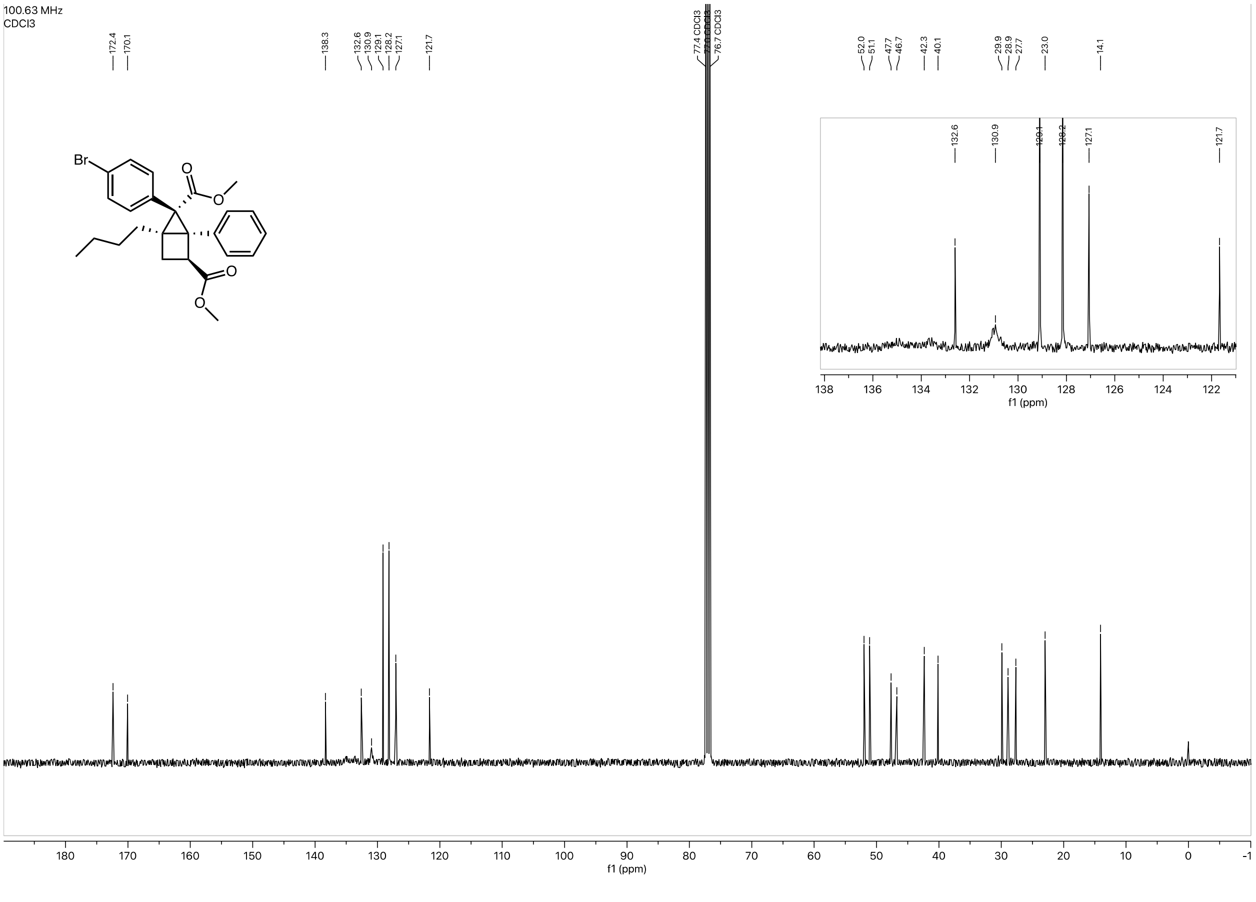

^{1}H NMR of **23d** (-20 ºC)

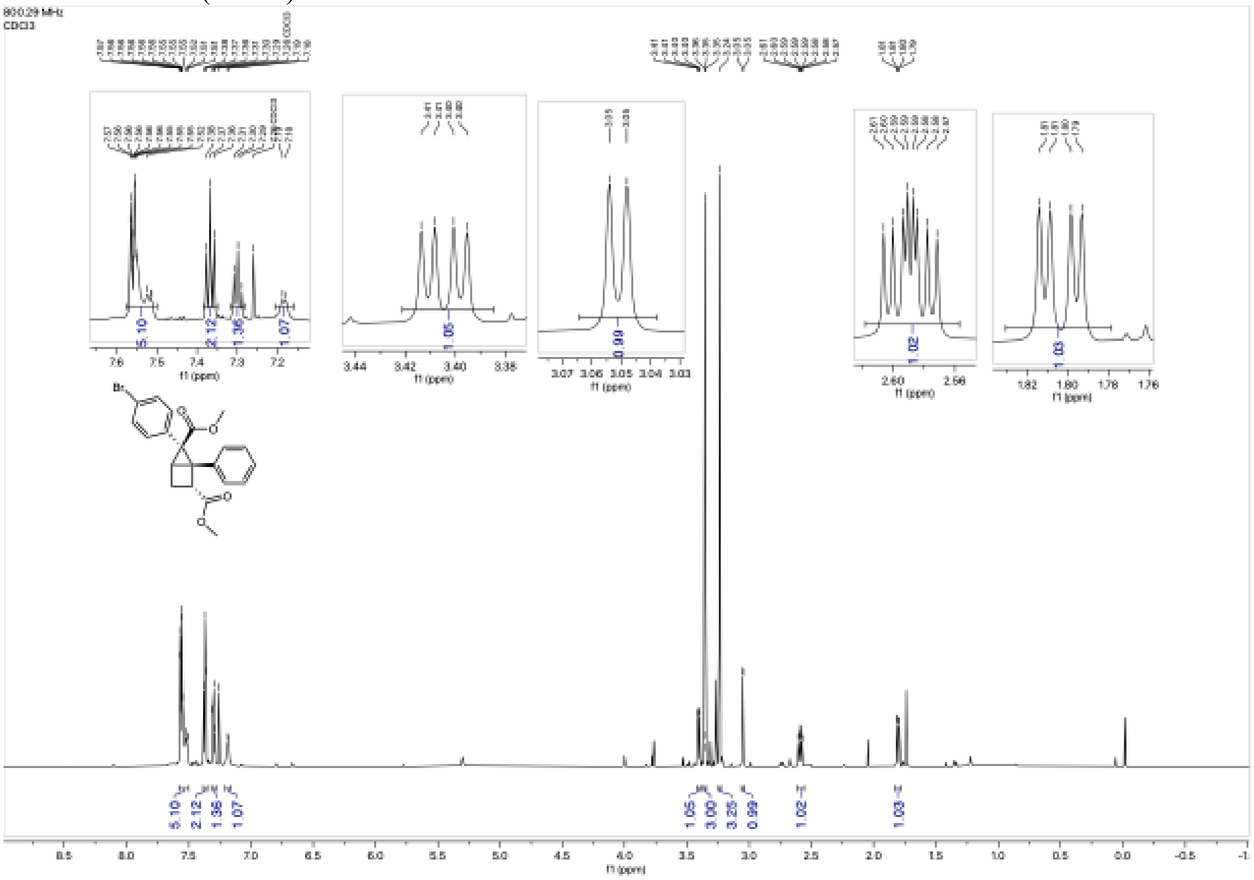

^{13}C NMR of **23d**

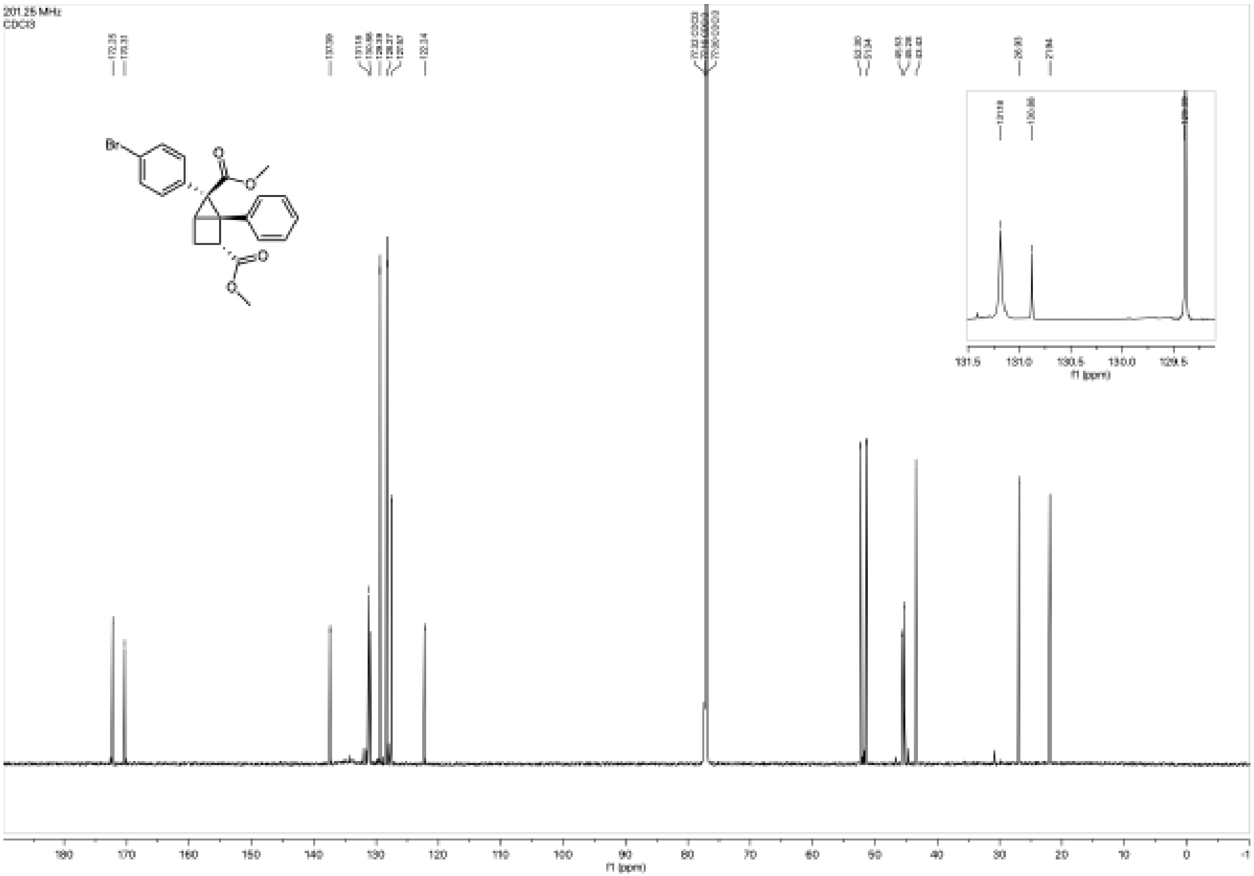

IX. CHIRAL SFC

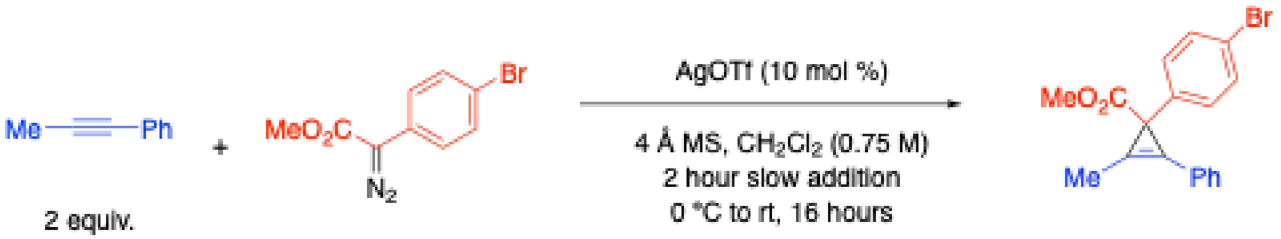
AgOTf (10 mol %)
4 Å MS, CH2Cl2 (0.75 M)
2 hour slow addition
0 °C to rt, 16 hours
2 equiv.

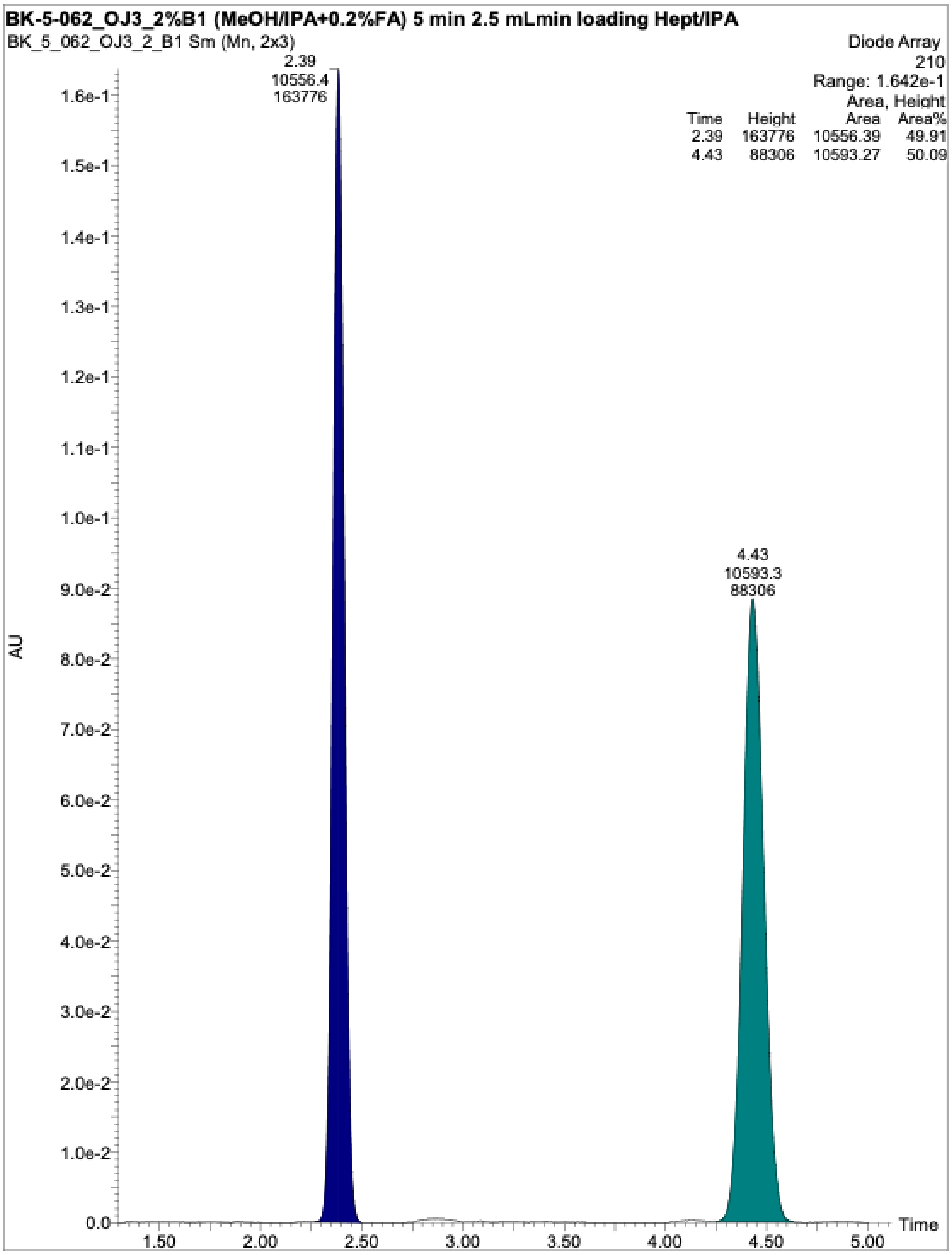
BK-5-062_OJ3_2%B1 (MeOH/IPA+0.2%FA) 5 min 2.5 mLmin loading Hept/IPA
BK_5_062_OJ3_2_B1 Sm (Mn, 2x3)
Diode Array
210
Range: 1.642e-1
Area, Height
Time Height Area Area%
2.39 163776 10556.39 49.91
4.43 88306 10593.27 50.09
2.39
10556.4
163776
4.43
10593.3
88306
AU
Time

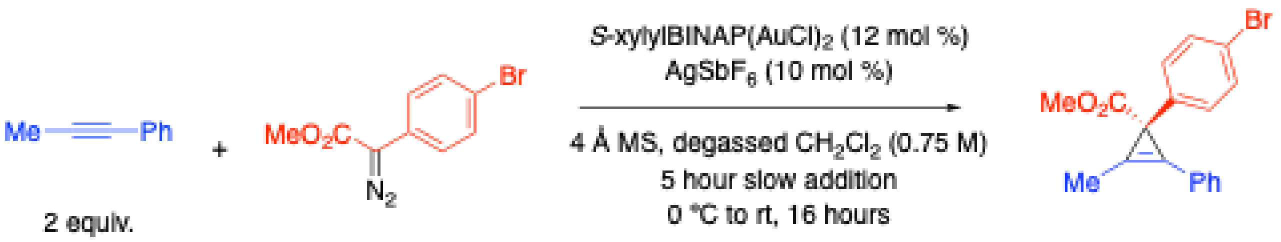
S-xylylBINAP(AuCl)2 (12 mol %)
AgSbF6 (10 mol %)
4 Å MS, degassed CH2Cl2 (0.75 M)
5 hour slow addition
0 °C to rt, 16 hours
Me
Ph
2 equiv.
MeO2C
Br
N2
MeO2C
Br
Me
Ph

ee = 90%

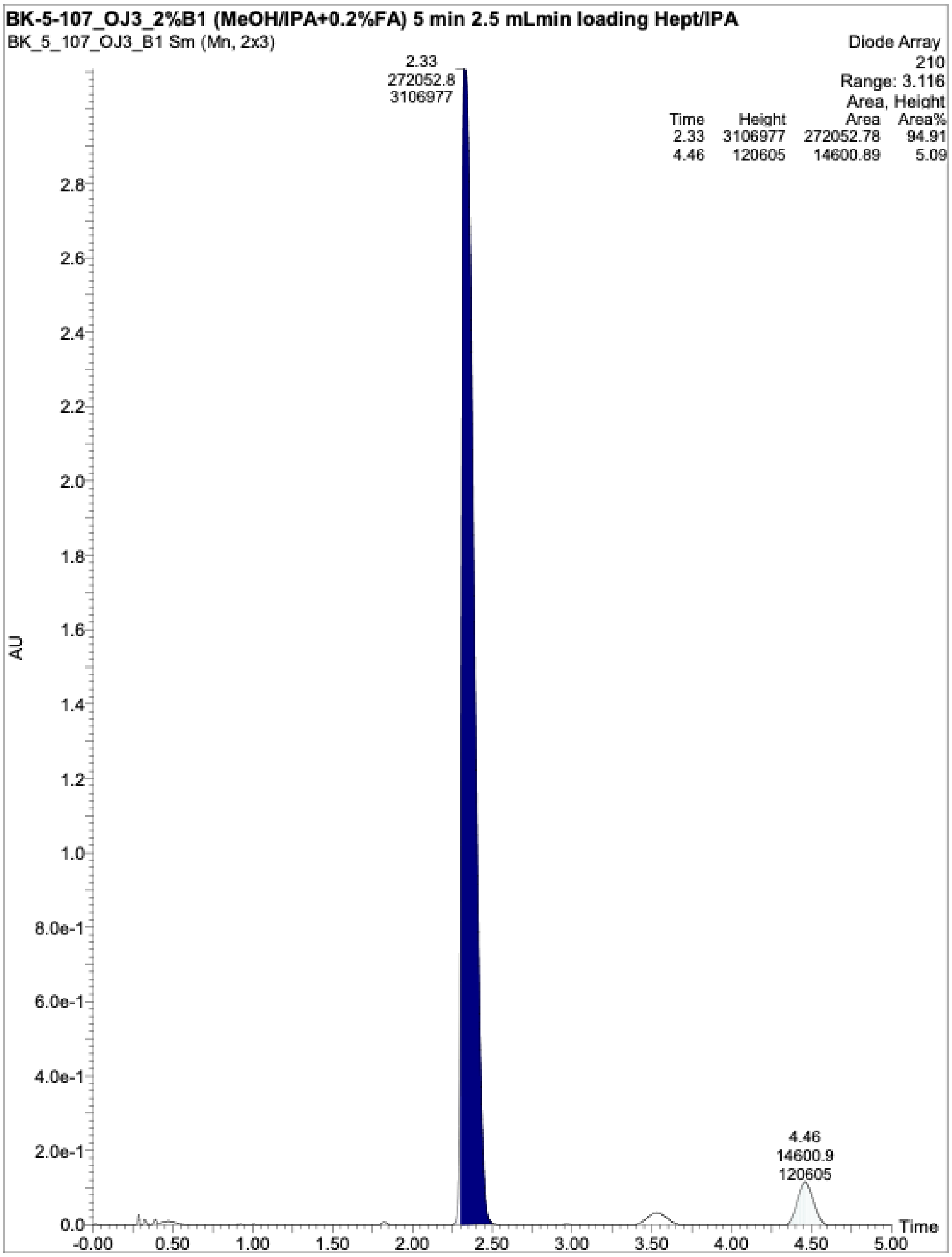
BK-5-107_OJ3_2%B1 (MeOH/IPA+0.2%FA) 5 min 2.5 mLmin loading Hept/IPA
BK_5_107_OJ3_B1 Sm (Mn, 2x3)
Diode Array
210
Range: 3.116
Area, Height
Time Height Area Area%
2.33 3106977 272052.78 94.91
4.46 120605 14600.89 5.09
2.33
272052.8
3106977
4.46
14600.9
120605
AU
Time

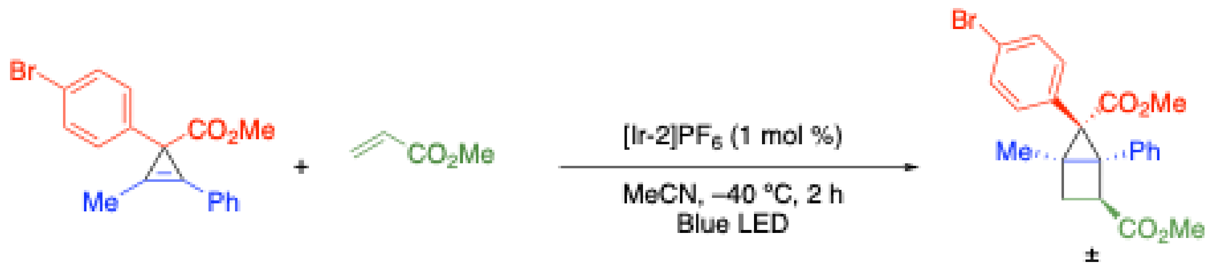

Br
CO2Me
Me
Ph
+
CO2Me
[Ir-2]PF6 (1 mol %)
MeCN, –40 °C, 2 h
Blue LED
Br
CO2Me
Me
Ph
CO2Me
±

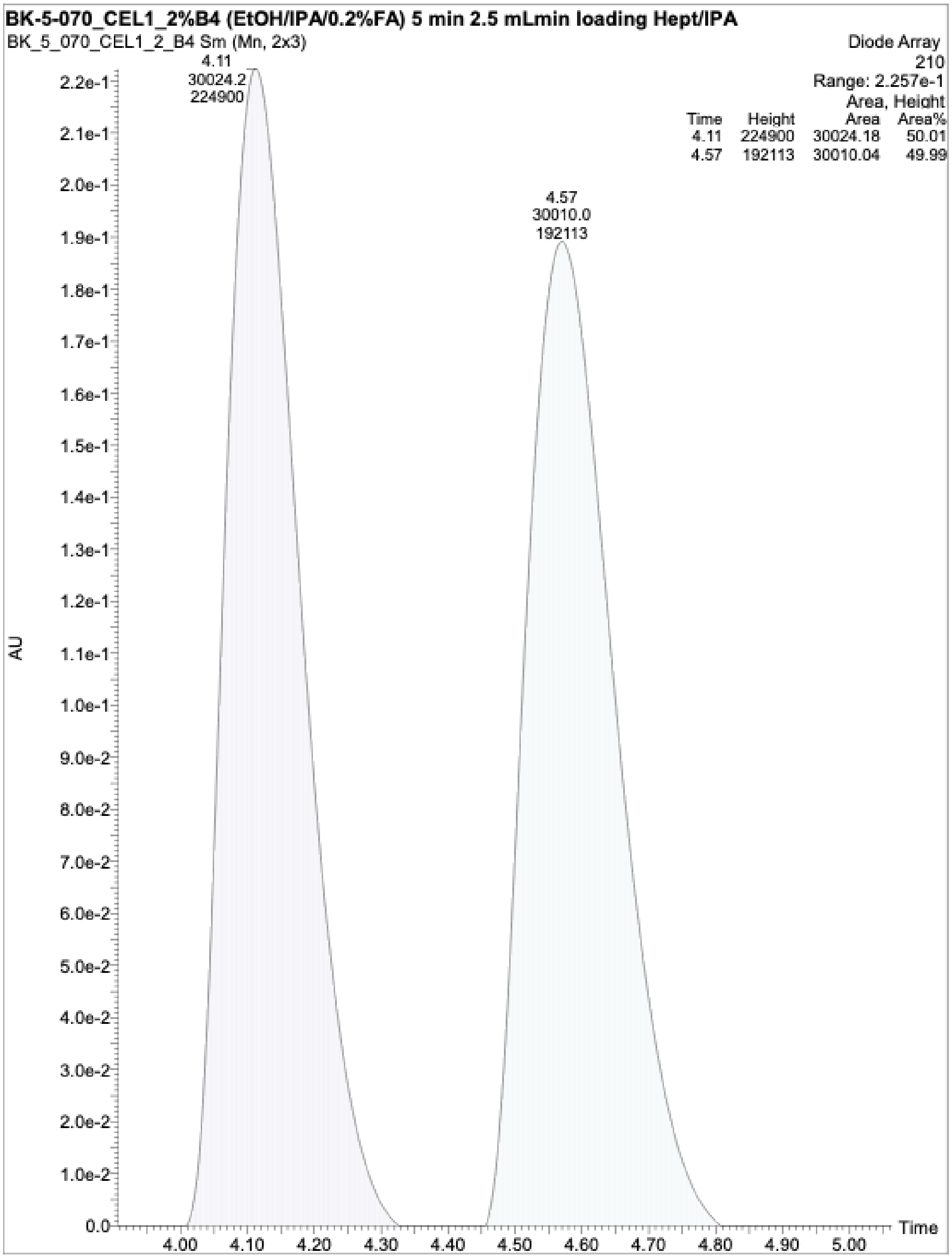

BK-5-070_CEL1_2%B4 (EtOH/IPA/0.2%FA) 5 min 2.5 mLmin loading Hept/IPA
BK_5_070_CEL1_2_B4 Sm (Mn, 2x3)
Diode Array
210
Range: 2.257e-1
Area, Height
Time Height Area Area%
4.11 224900 30024.18 50.01
4.57 192113 30010.04 49.99
4.11
30024.2
224900
4.57
30010.0
192113
AU
Time

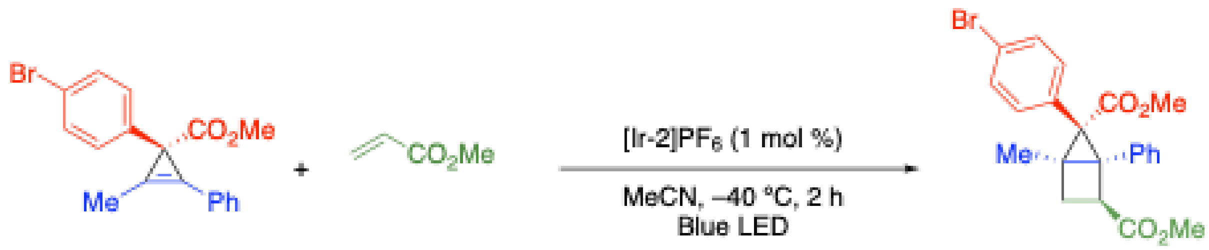
Br
CO2Me
Me
Ph
+
CO2Me
[Ir-2]PF6 (1 mol %)
MeCN, –40 °C, 2 h
Blue LED
Br
CO2Me
Me
Ph
CO2Me

ee = 91.5%

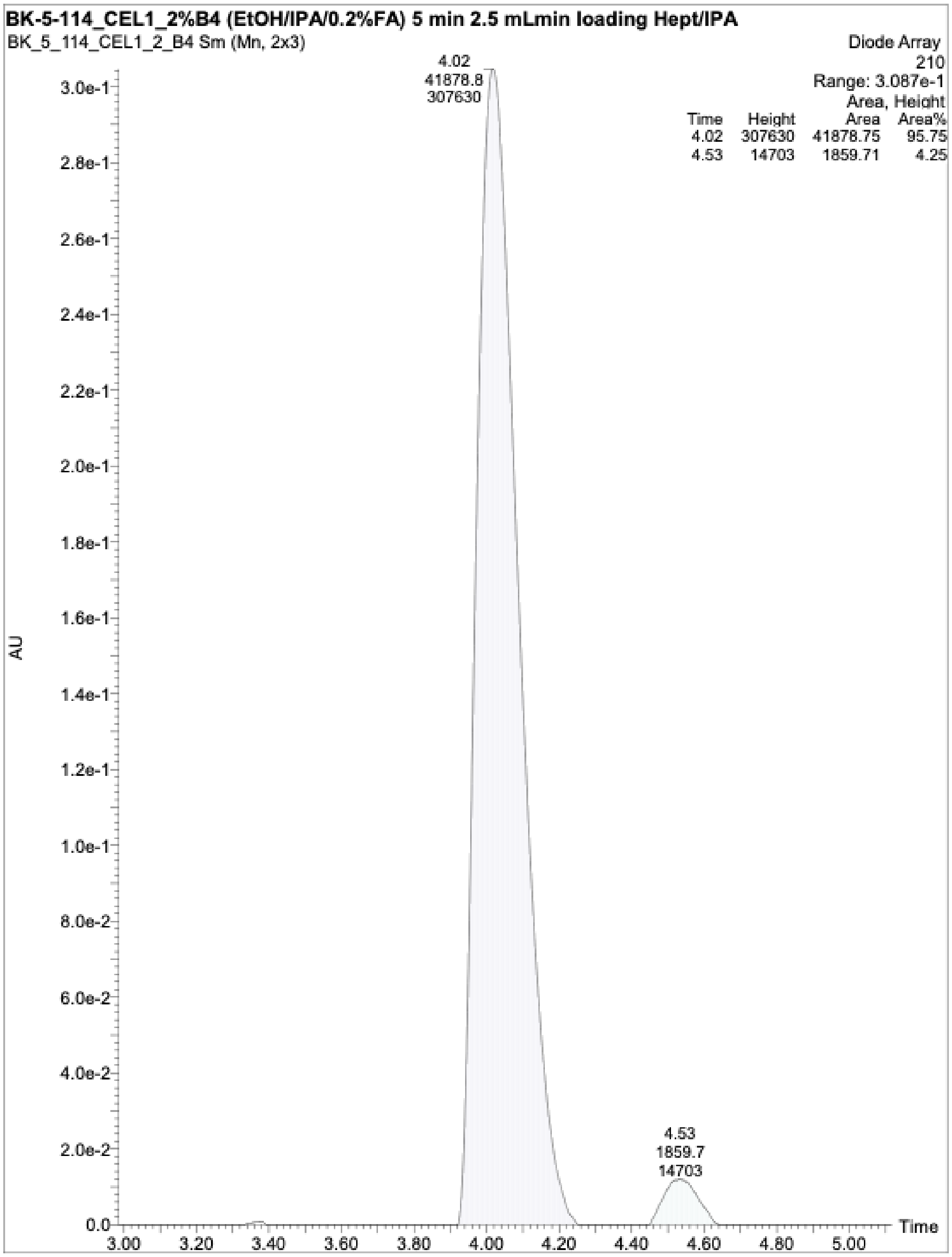
BK-5-114_CEL1_2%B4 (EtOH/IPA/0.2%FA) 5 min 2.5 mLmin loading Hept/IPA
BK_5_114_CEL1_2_B4 Sm (Mn, 2x3)
Diode Array
210
Range: 3.087e-1
Area, Height
Time Height Area Area%
4.02 307630 41878.75 95.75
4.53 14703 1859.71 4.25
4.02
41878.8
307630
4.53
1859.7
14703
AU
Time

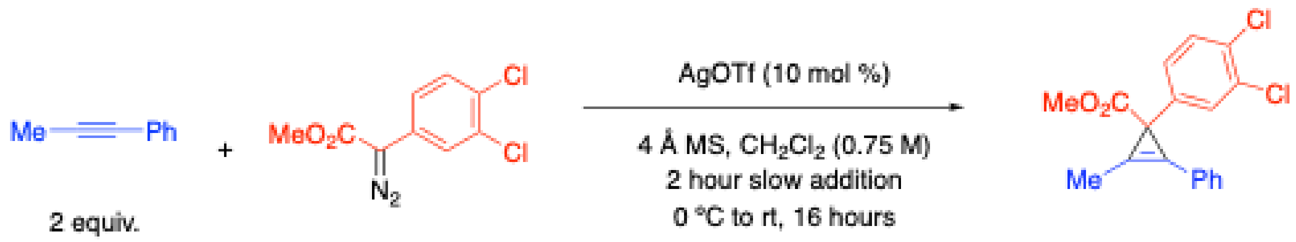
Me
Ph
2 equiv.
+
MeO2C
N2
Cl
Cl
AgOTf (10 mol %)
4 Å MS, CH2Cl2 (0.75 M)
2 hour slow addition
0 °C to rt, 16 hours
MeO2C
Cl
Cl
Me
Ph

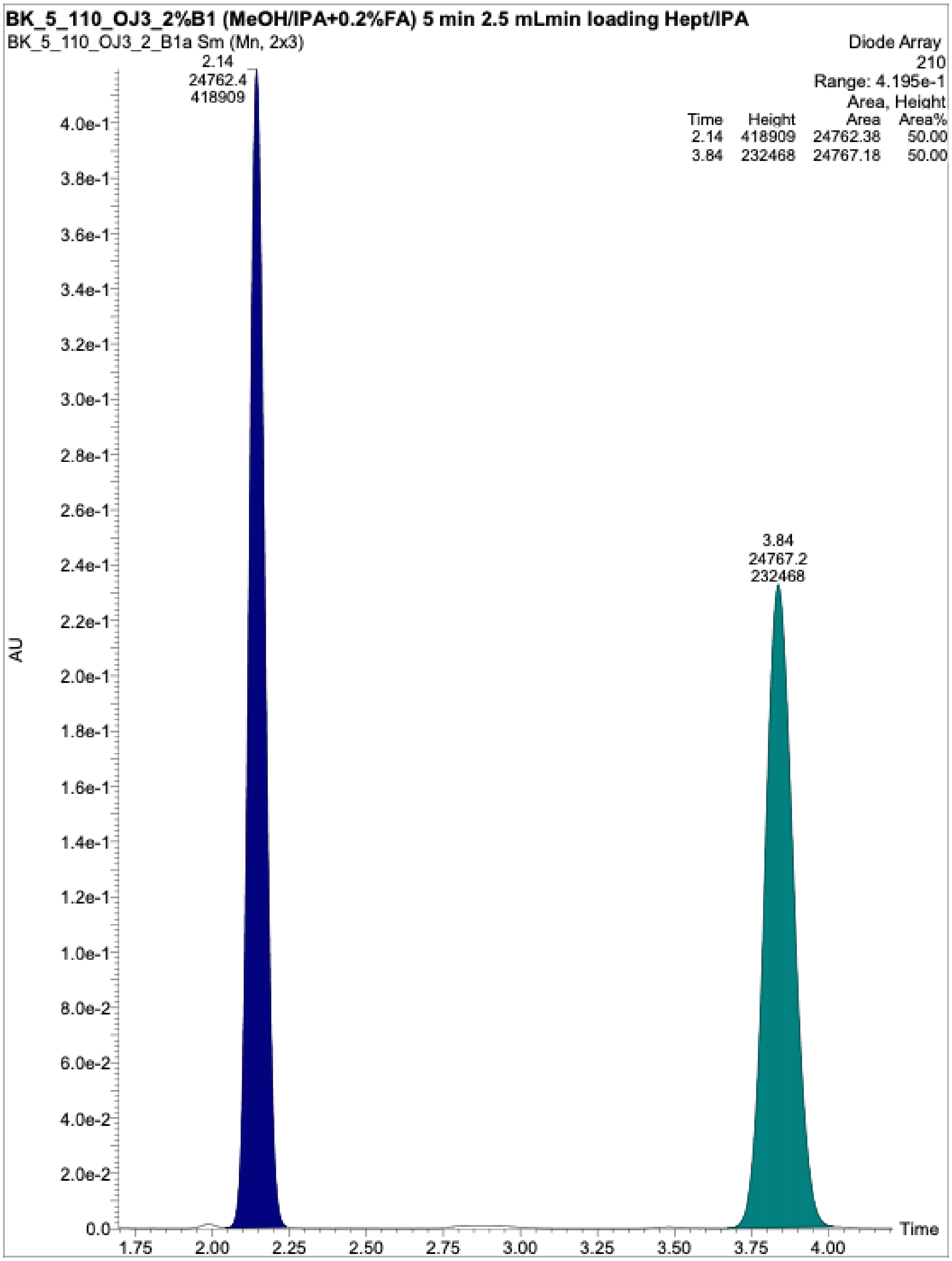
BK_5_110_OJ3_2%B1 (MeOH/IPA+0.2%FA) 5 min 2.5 mLmin loading Hept/IPA
BK_5_110_OJ3_2_B1a Sm (Mn, 2x3)
Diode Array
210
Range: 4.195e-1
Area, Height
Time Height Area Area%
2.14 418909 24762.38 50.00
3.84 232468 24767.18 50.00
2.14
24762.4
418909
3.84
24767.2
232468
AU
Time

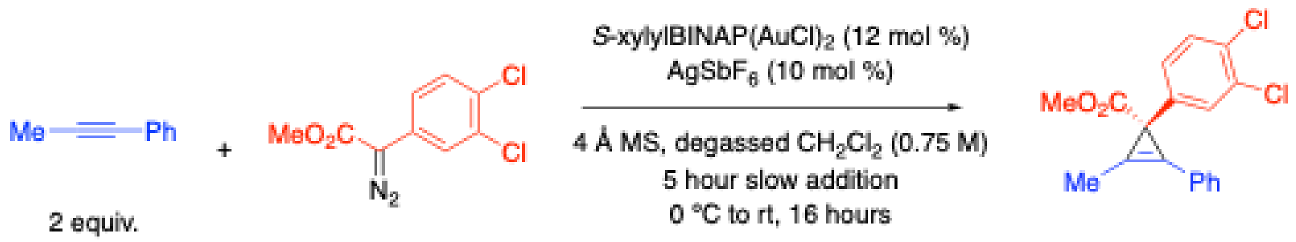
Me
Ph
2 equiv.
MeO2C
Cl
Cl
N2
S-xylylBINAP(AuCl)2 (12 mol %)
AgSbF6 (10 mol %)
4 Å MS, degassed CH2Cl2 (0.75 M)
5 hour slow addition
0 °C to rt, 16 hours
MeO2C
Cl
Cl
Me
Ph

ee = 90%

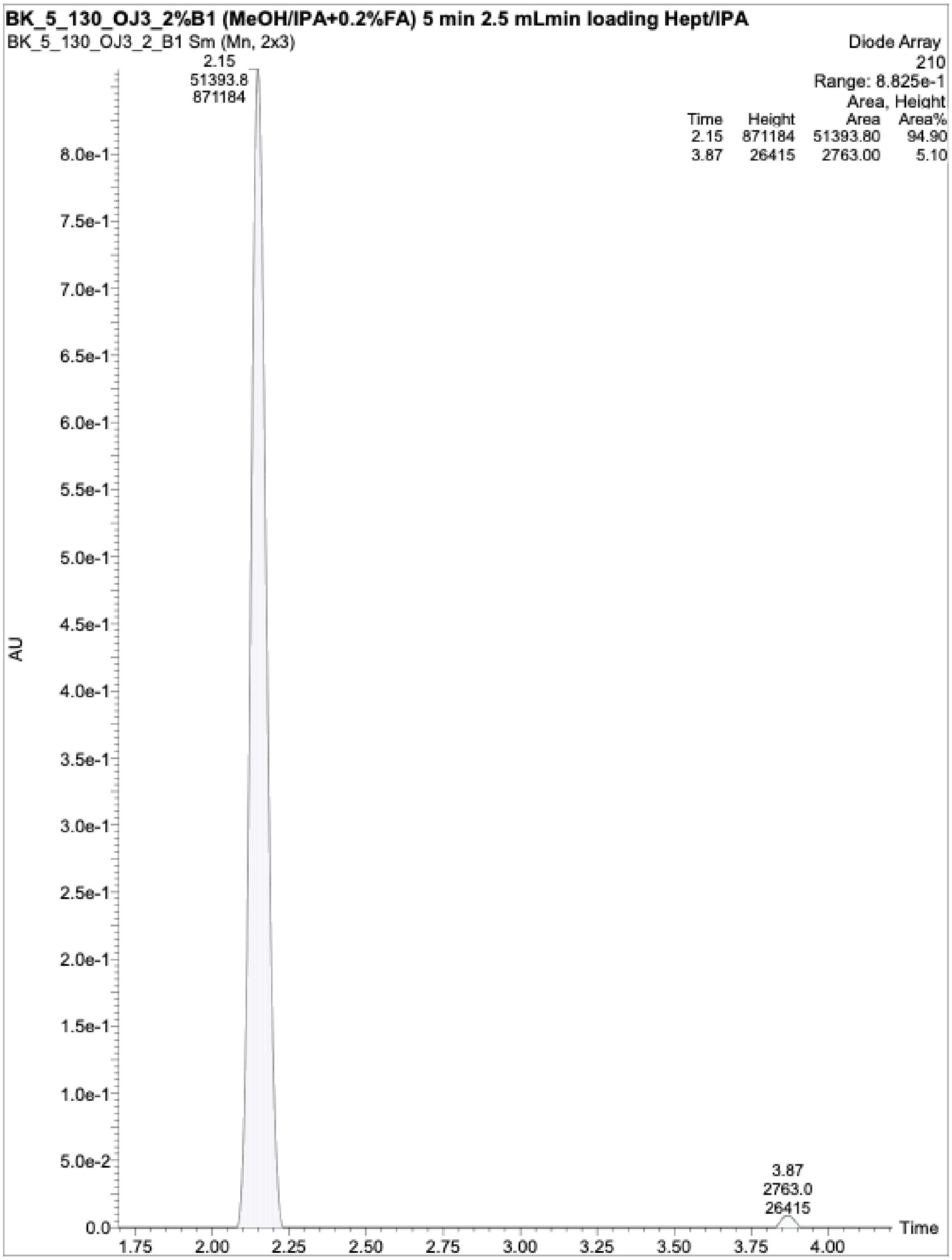
BK_5_130_OJ3_2%B1 (MeOH/IPA+0.2%FA) 5 min 2.5 mLmin loading Hept/IPA
BK_5_130_OJ3_2_B1 Sm (Mn, 2x3)
Diode Array
210
Range: 8.825e-1
Area, Height
Time Height Area Area%
2.15 871184 51393.80 94.90
3.87 26415 2763.00 5.10
2.15
51393.8
871184
3.87
2763.0
26415
AU
Time

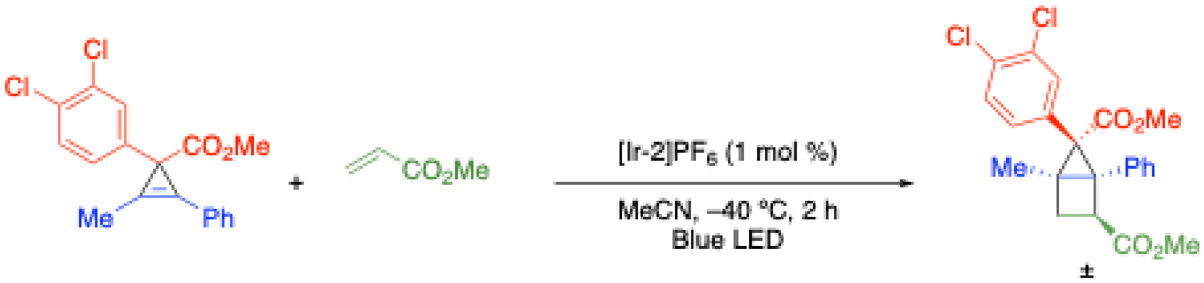

Cl
Cl
CO2Me
Me
Ph
+
CO2Me
[Ir-2]PF6 (1 mol %)
MeCN, –40 °C, 2 h
Blue LED
Cl
Cl
CO2Me
Me
Ph
CO2Me
±

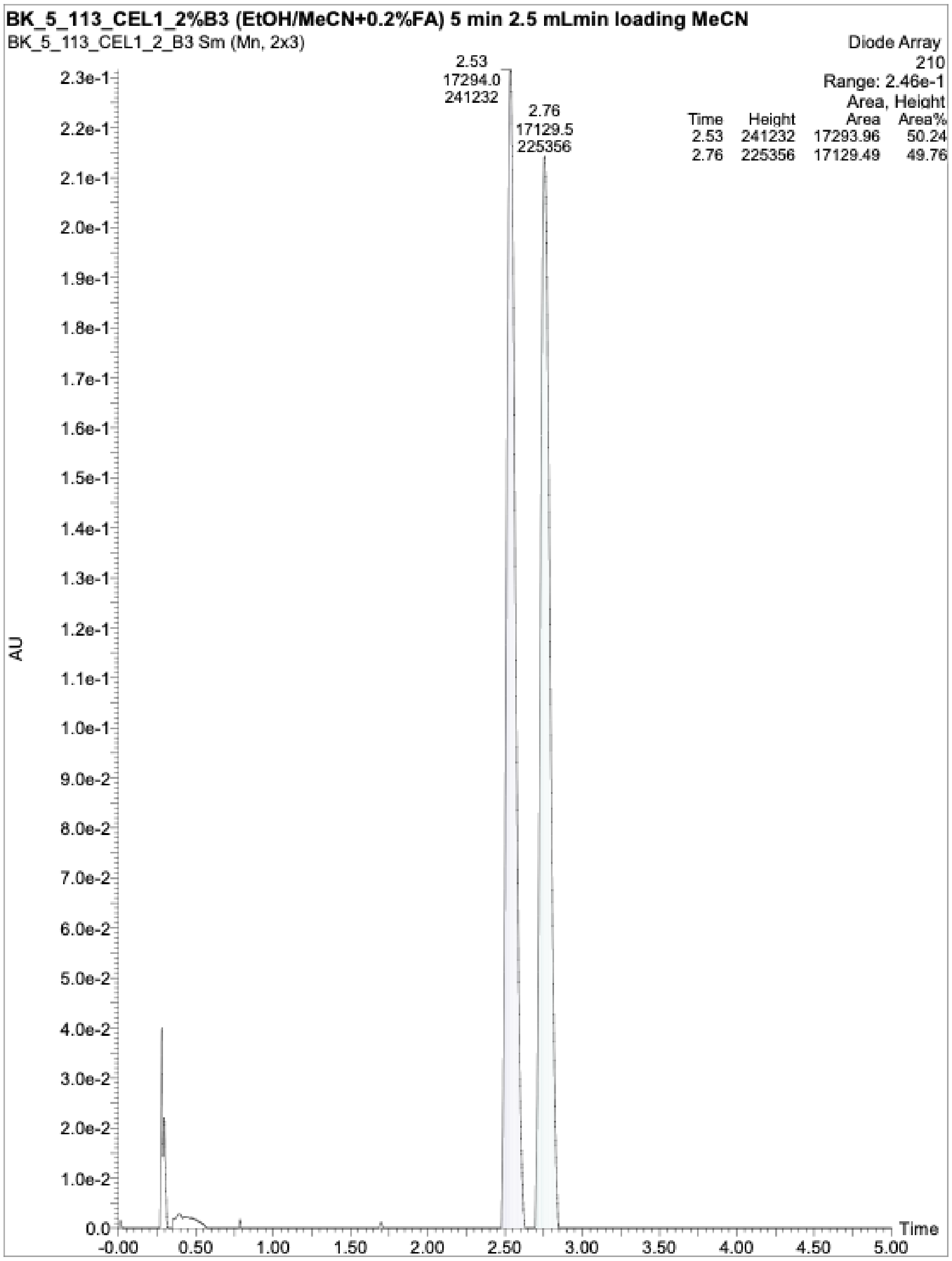

BK_5_113_CEL1_2%B3 (EtOH/MeCN+0.2%FA) 5 min 2.5 mLmin loading MeCN
BK_5_113_CEL1_2_B3 Sm (Mn, 2x3)
Diode Array
210
Range: 2.46e-1
Area, Height
Time Height Area Area%
2.53 241232 17293.96 50.24
2.76 225356 17129.49 49.76
2.53
17294.0
241232
2.76
17129.5
225356
AU
Time

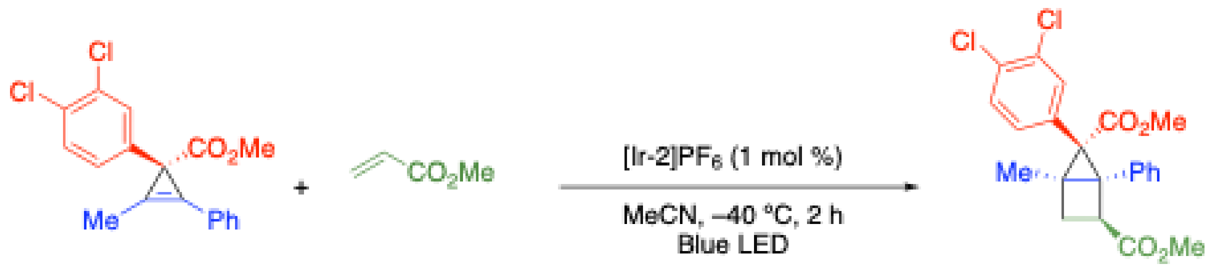
Cl
Cl
CO2Me
Me
Ph
+
CO2Me
[Ir-2]PF6 (1 mol %)
MeCN, –40 °C, 2 h
Blue LED
Cl
Cl
CO2Me
Me
Ph
CO2Me

ee = 89%

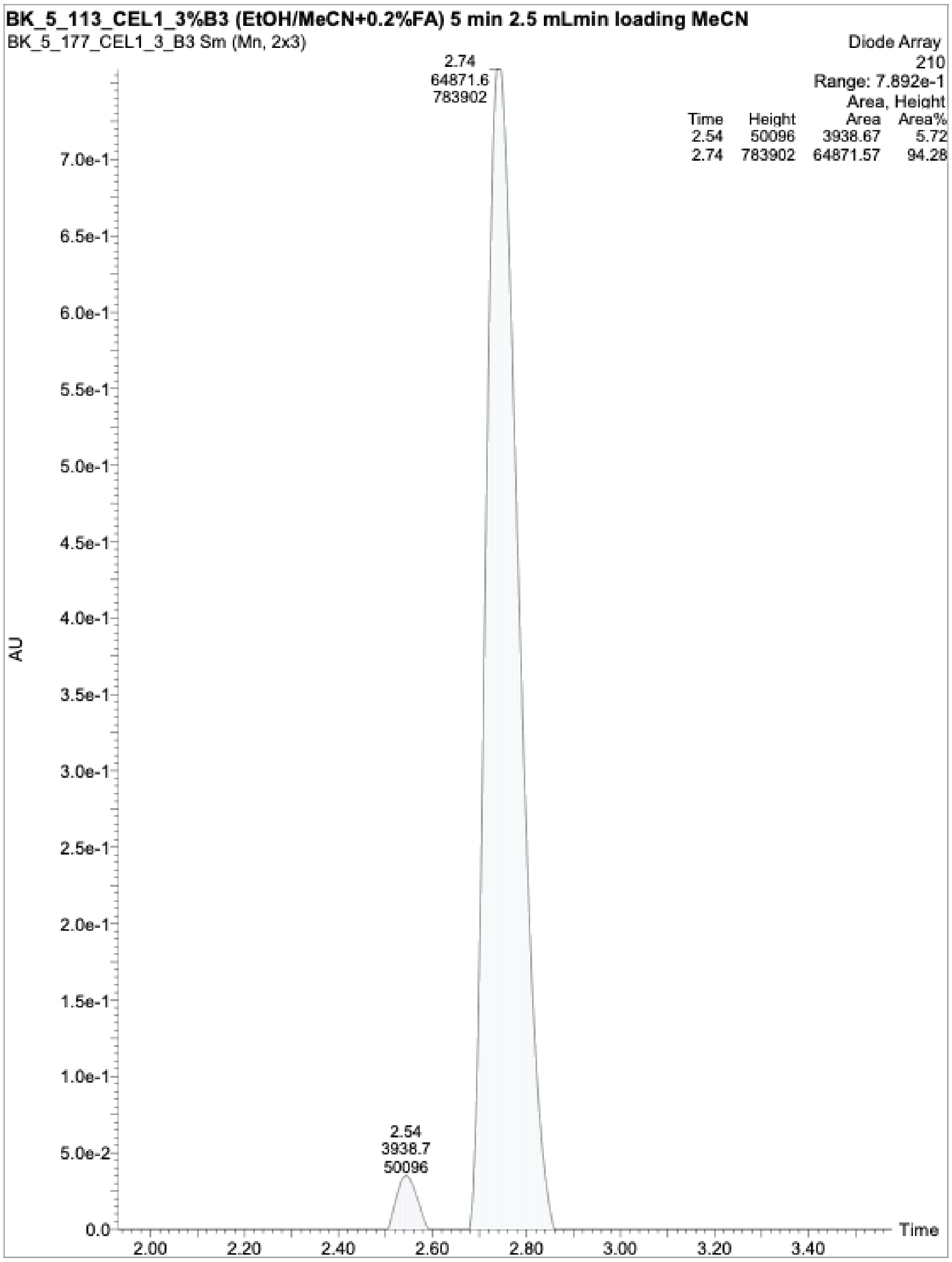
BK_5_113_CEL1_3%B3 (EtOH/MeCN+0.2%FA) 5 min 2.5 mLmin loading MeCN
BK_5_177_CEL1_3_B3 Sm (Mn, 2x3)
Diode Array
210
Range: 7.892e-1
Area, Height
Time Height Area Area%
2.54 50096 3938.67 5.72
2.74 783902 64871.57 94.28
2.74
64871.6
783902
2.54
3938.7
50096
AU
Time

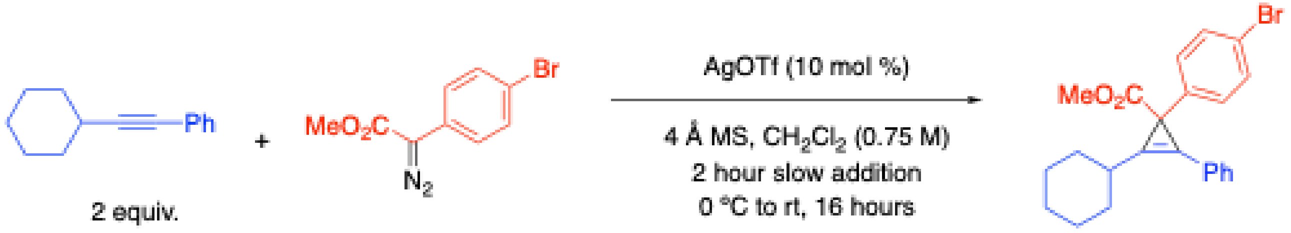
Ph
2 equiv.
+
MeO2C
N2
Br
AgOTf (10 mol %)
4 Å MS, CH2Cl2 (0.75 M)
2 hour slow addition
0 °C to rt, 16 hours
MeO2C
Br
Ph

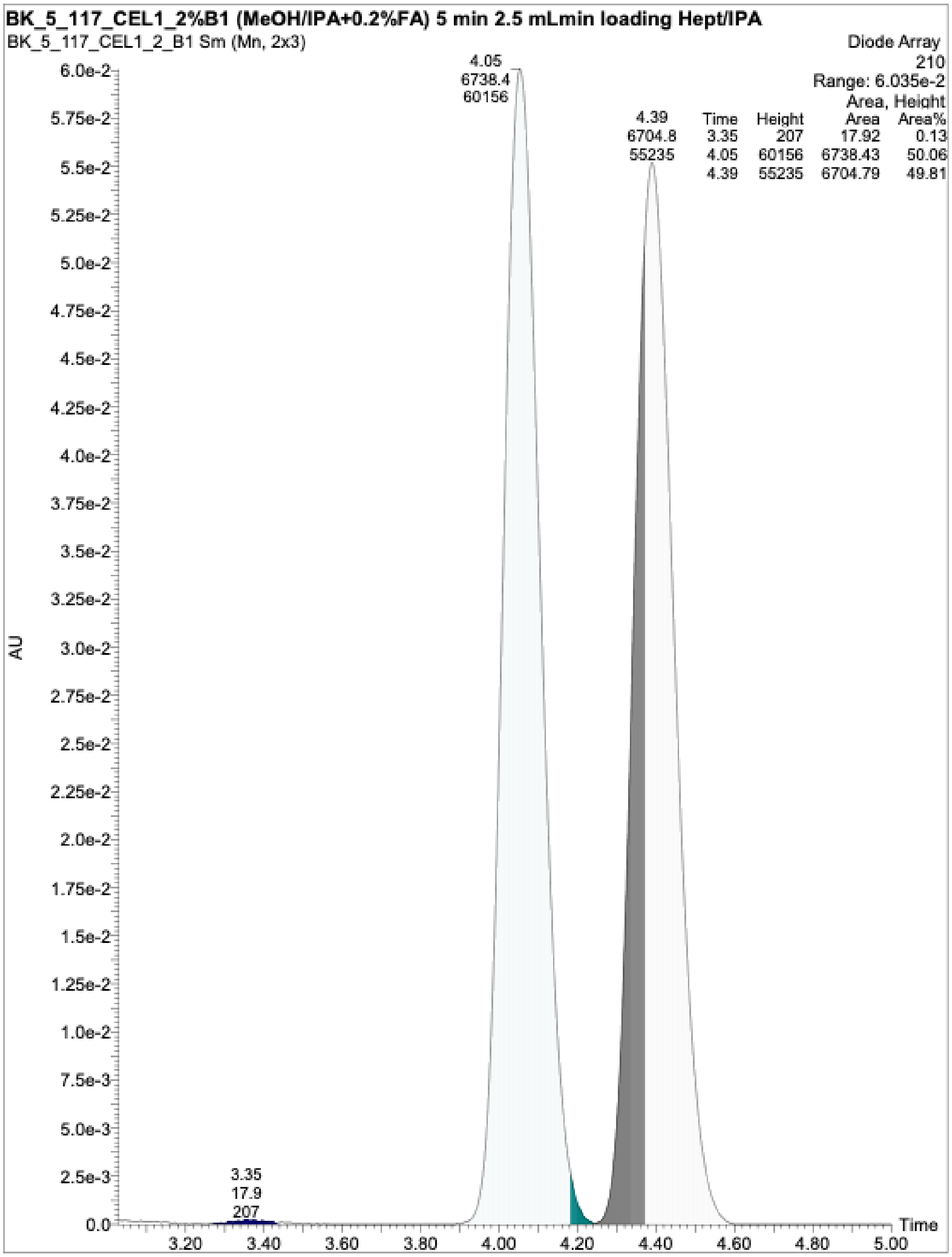
BK_5_117_CEL1_2%B1 (MeOH/IPA+0.2%FA) 5 min 2.5 mLmin loading Hept/IPA
BK_5_117_CEL1_2_B1 Sm (Mn, 2x3)
Diode Array
210
Range: 6.035e-2
Area, Height
Time Height Area Area%
3.35 207 17.92 0.13
4.05 60156 6738.43 50.06
4.39 55235 6704.79 49.81
4.05
6738.4
60156
4.39
6704.8
55235
3.35
17.9
207
AU
Time

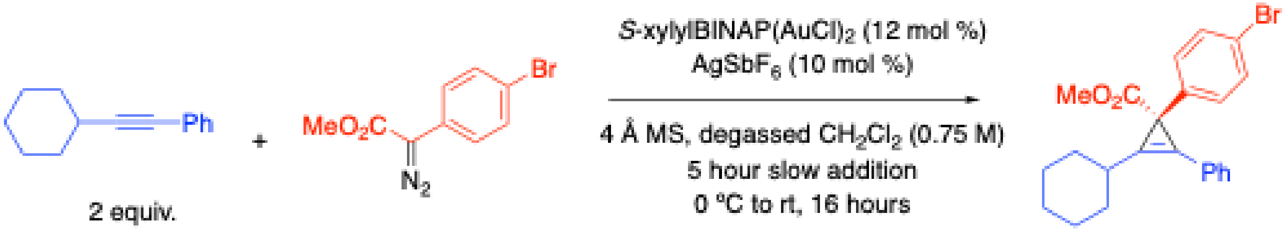
Ph
2 equiv.
+
MeO2C
Br
N2
S-xylylBINAP(AuCl)2 (12 mol %)
AgSbF6 (10 mol %)
4 Å MS, degassed CH2Cl2 (0.75 M)
5 hour slow addition
0 °C to rt, 16 hours
MeO2C
Br
Ph

ee = 88%

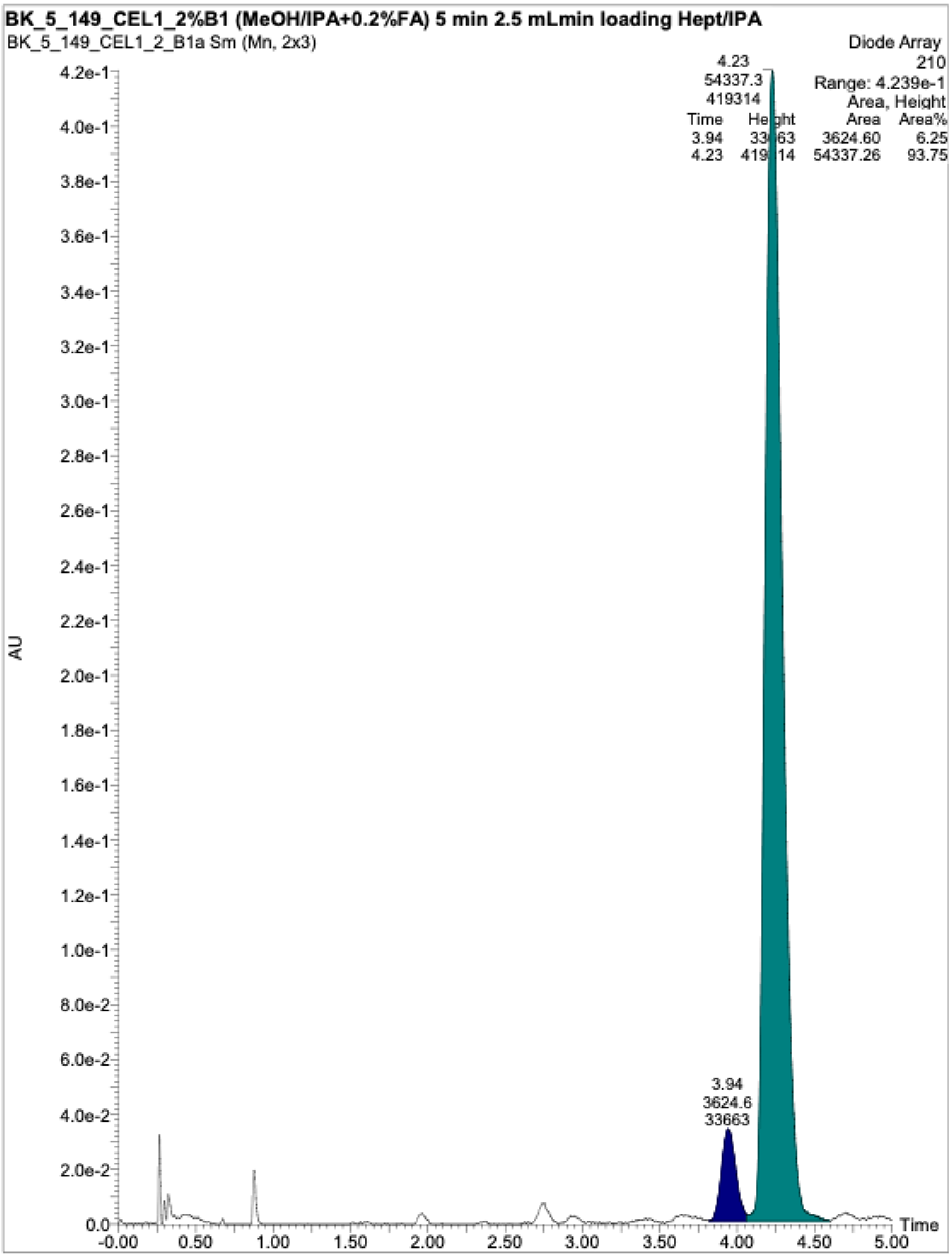
BK_5_149_CEL1_2%B1 (MeOH/IPA+0.2%FA) 5 min 2.5 mLmin loading Hept/IPA
BK_5_149_CEL1_2_B1a Sm (Mn, 2x3)
Diode Array
210
Range: 4.239e-1
Area, Height
Time Height Area Area%
3.94 33663 3624.60 6.25
4.23 419314 54337.26 93.75
4.23
54337.3
419314
3.94
3624.6
33663
AU
Time

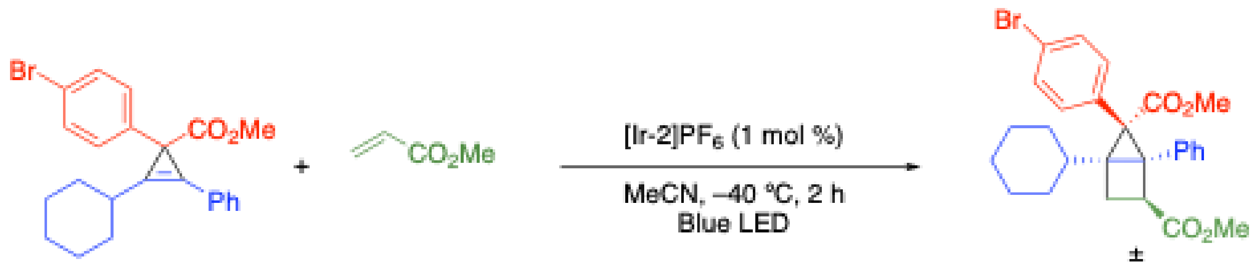

Br
CO2Me
Ph
+
CO2Me
[Ir-2]PF6 (1 mol %)
MeCN, –40 °C, 2 h
Blue LED
Br
CO2Me
Ph
CO2Me
±

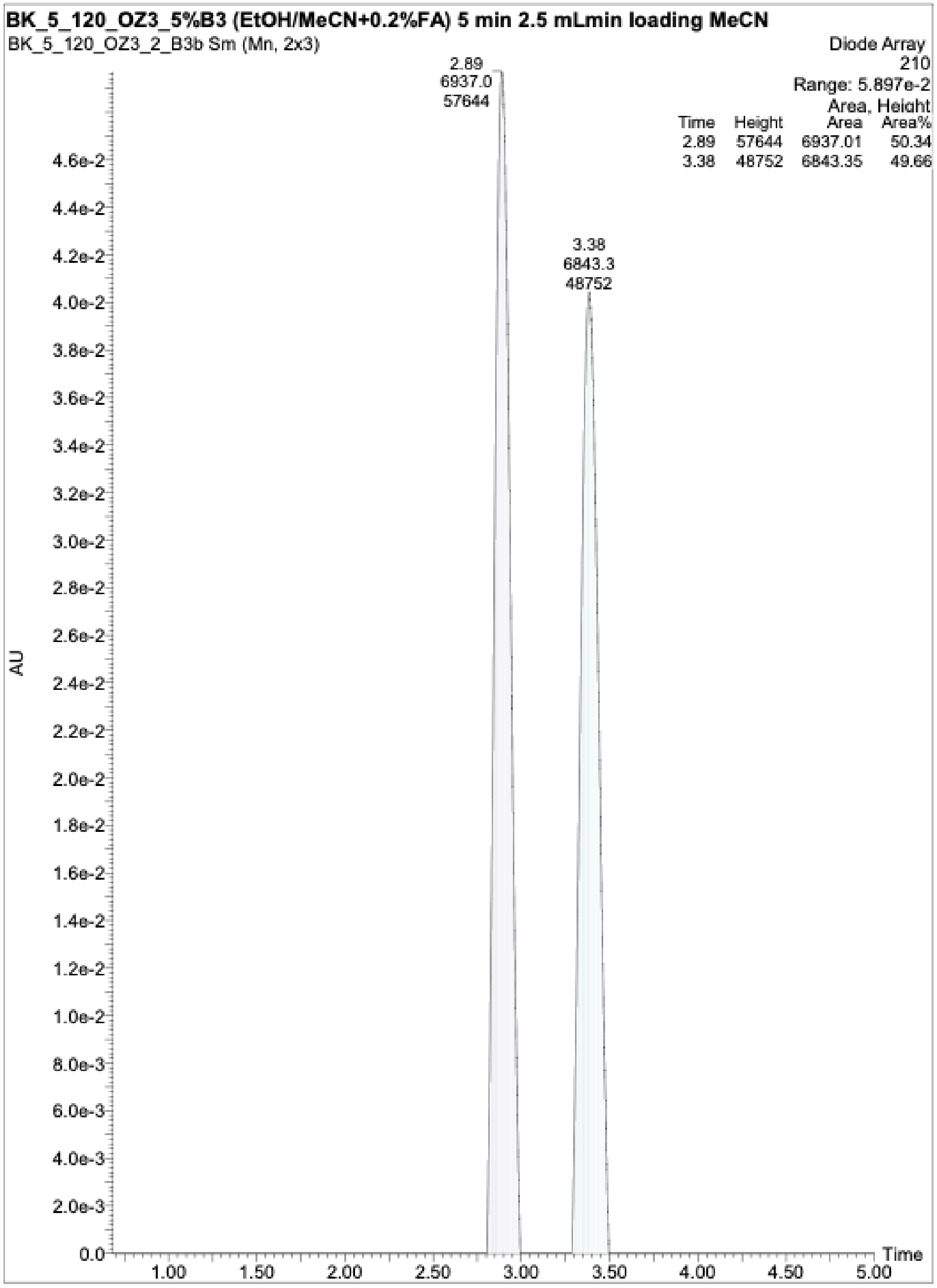

BK_5_120_OZ3_5%B3 (EtOH/MeCN+0.2%FA) 5 min 2.5 mLmin loading MeCN
BK_5_120_OZ3_2_B3b Sm (Mn, 2x3)
Diode Array
210
Range: 5.897e-2
Area, Height
Time Height Area Area%
2.89 57644 6937.01 50.34
3.38 48752 6843.35 49.66
2.89
6937.0
57644
3.38
6843.3
48752
AU
Time

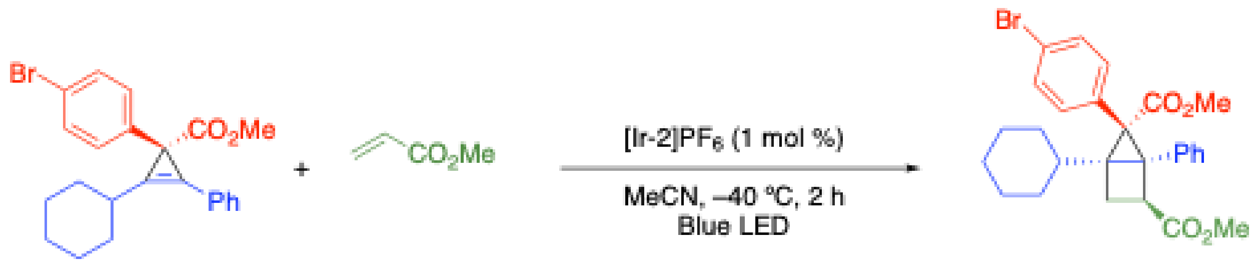

Br
CO2Me
Ph
CO2Me
[Ir-2]PF6 (1 mol %)
MeCN, –40 °C, 2 h
Blue LED
Br
CO2Me
Ph
CO2Me

ee = 85%

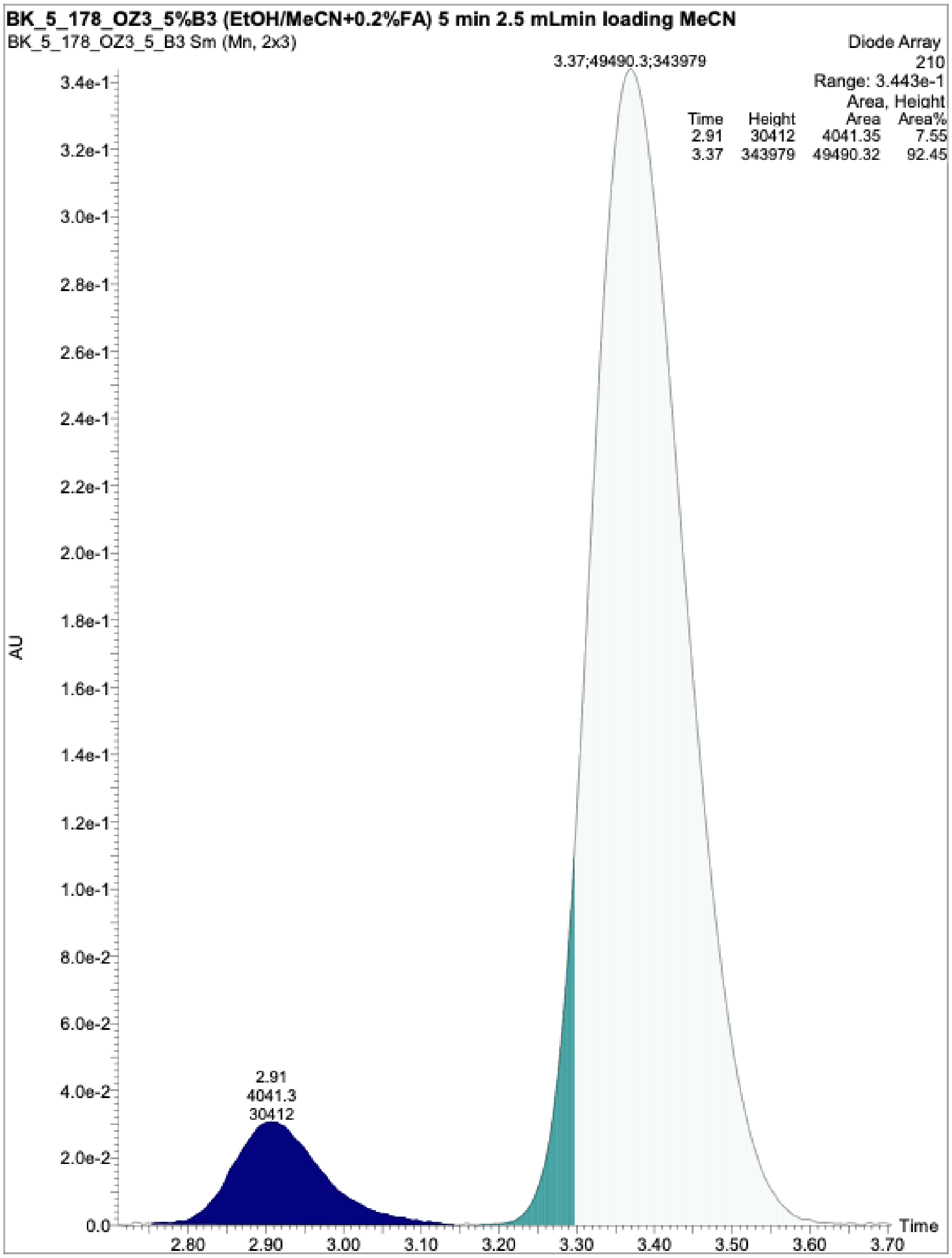

BK_5_178_OZ3_5%B3 (EtOH/MeCN+0.2%FA) 5 min 2.5 mLmin loading MeCN
BK_5_178_OZ3_5_B3 Sm (Mn, 2x3)
Diode Array
210
Range: 3.443e-1
Area, Height
3.37;49490.3;343979
Time Height Area Area%
2.91 30412 4041.35 7.55
3.37 343979 49490.32 92.45
2.91
4041.3
30412
AU
Time

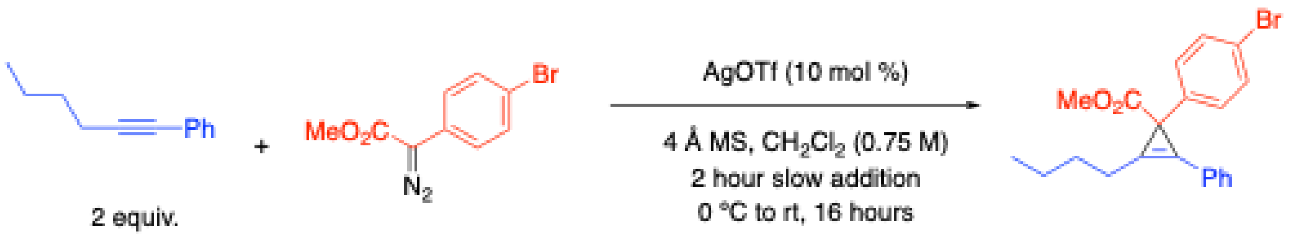

Br
Ph
MeO2C
N2
2 equiv.
AgOTf (10 mol %)
4 Å MS, CH2Cl2 (0.75 M)
2 hour slow addition
0 °C to rt, 16 hours

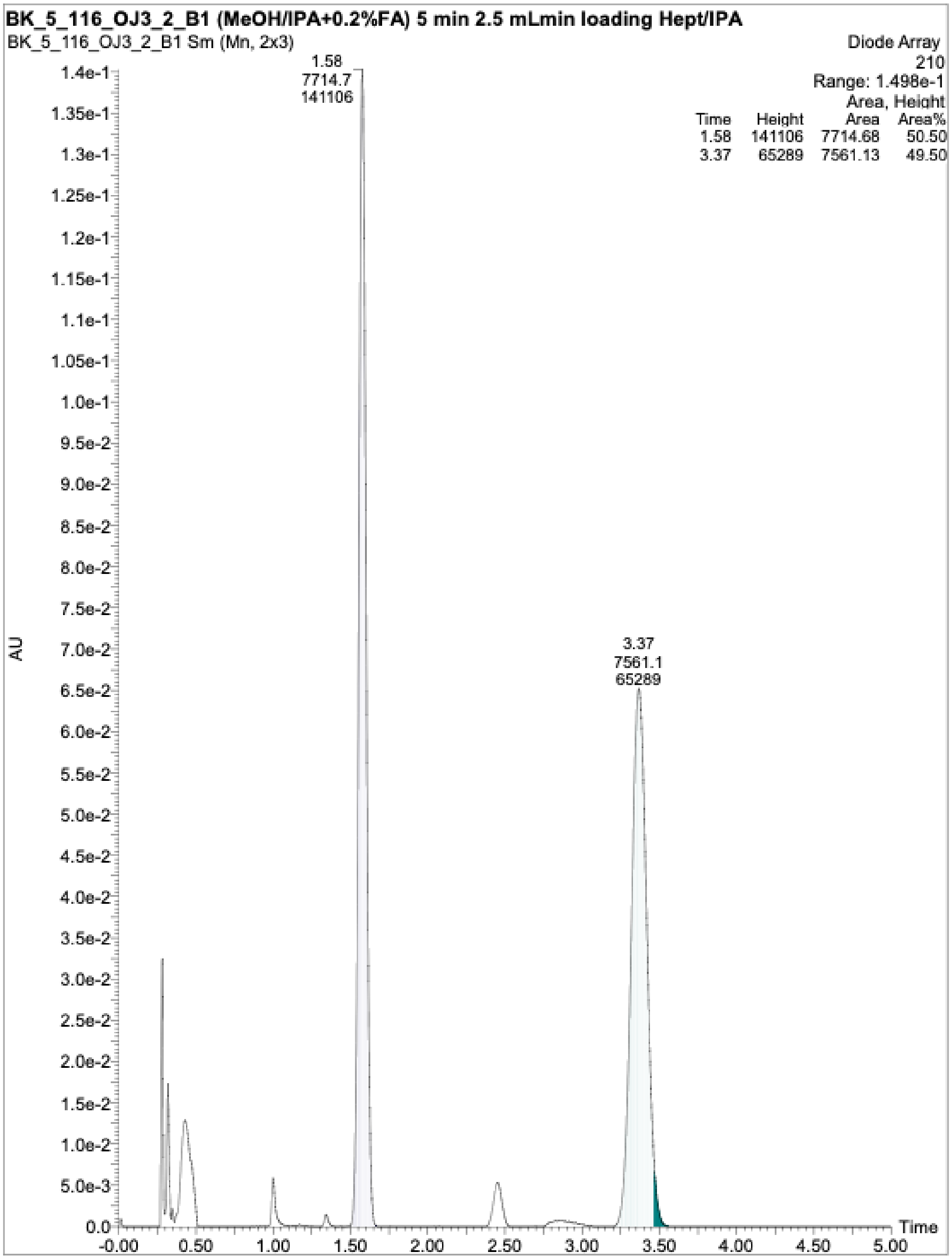

BK_5_116_OJ3_2_B1 (MeOH/IPA+0.2%FA) 5 min 2.5 mLmin loading Hept/IPA
BK_5_116_OJ3_2_B1 Sm (Mn, 2x3)
Diode Array
210
Range: 1.498e-1
Area, Height
Time Height Area Area%
1.58 141106 7714.68 50.50
3.37 65289 7561.13 49.50
1.58
7714.7
141106
3.37
7561.1
65289
AU
Time

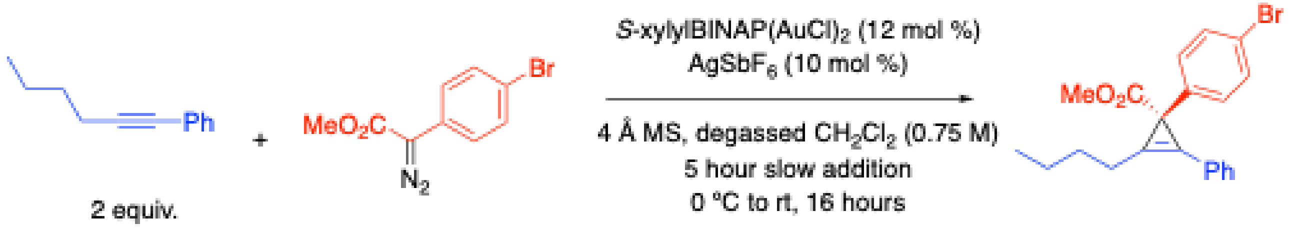
S-xylylBINAP(AuCl)2 (12 mol %)
AgSbF6 (10 mol %)
4 Å MS, degassed CH2Cl2 (0.75 M)
5 hour slow addition
0 °C to rt, 16 hours
2 equiv.

ee = 91%

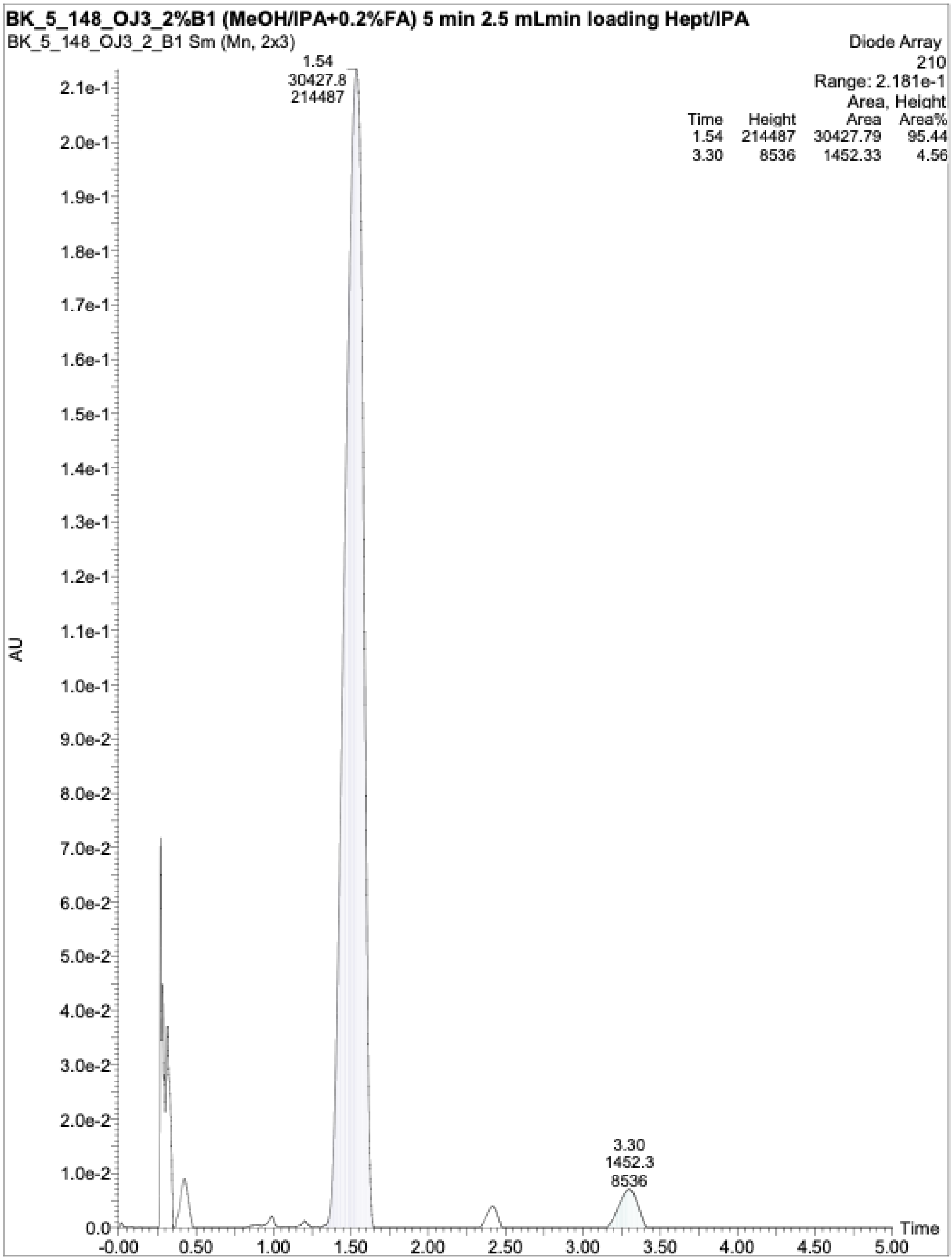
BK_5_148_OJ3_2%B1 (MeOH/IPA+0.2%FA) 5 min 2.5 mLmin loading Hept/IPA
BK_5_148_OJ3_2_B1 Sm (Mn, 2x3)
Diode Array
210
Range: 2.181e-1
Area, Height
Time Height Area Area%
1.54 214487 30427.79 95.44
3.30 8536 1452.33 4.56
1.54
30427.8
214487
3.30
1452.3
8536
AU
Time

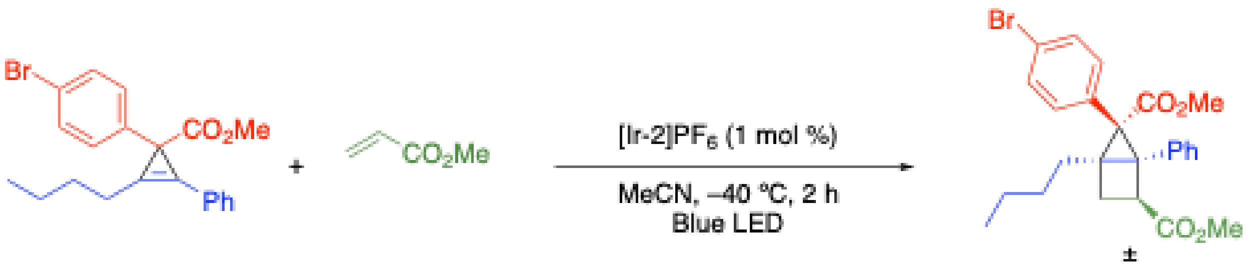
Br
CO2Me
Ph
+
CO2Me
[Ir-2]PF6 (1 mol %)
MeCN, –40 °C, 2 h
Blue LED
Br
CO2Me
Ph
CO2Me
±

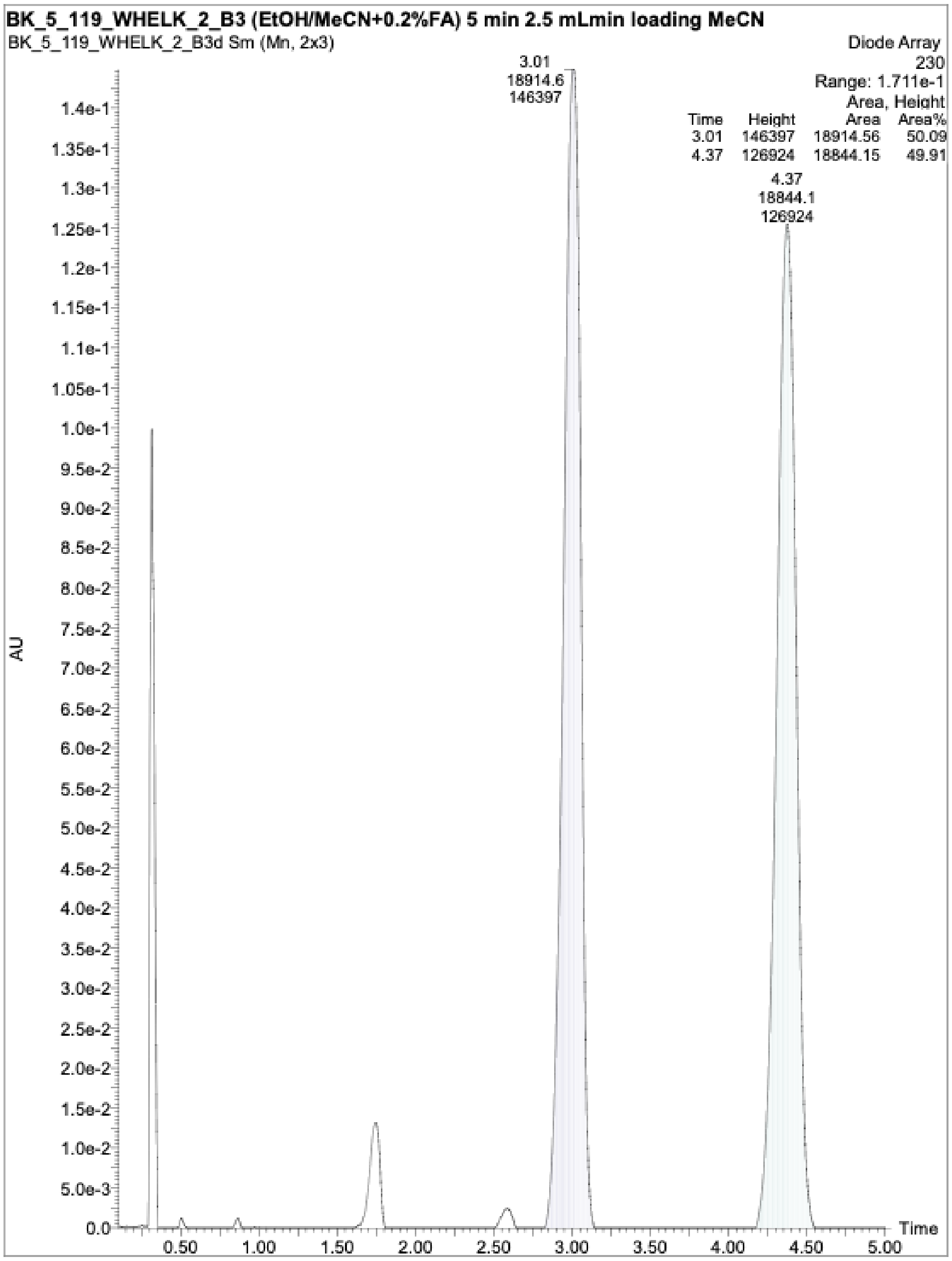
BK_5_119_WHELK_2_B3 (EtOH/MeCN+0.2%FA) 5 min 2.5 mLmin loading MeCN
BK_5_119_WHELK_2_B3d Sm (Mn, 2x3)
Diode Array
230
Range: 1.711e-1
Area, Height
Time Height Area Area%
3.01 146397 18914.56 50.09
4.37 126924 18844.15 49.91
3.01
18914.6
146397
4.37
18844.1
126924
AU
Time

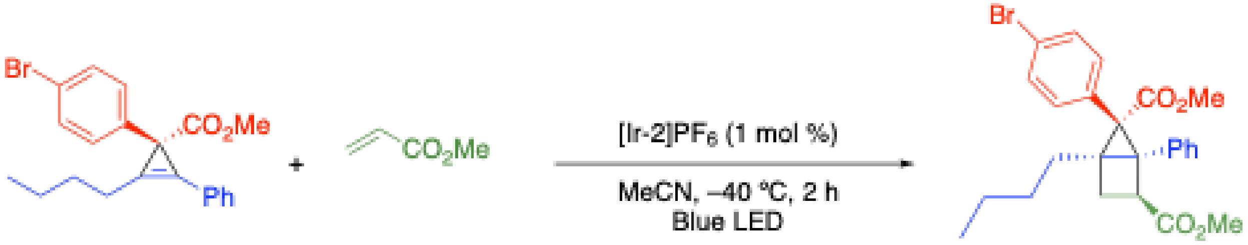

Br
CO2Me
Ph
CO2Me
[Ir-2]PF6 (1 mol %)
MeCN, –40 °C, 2 h
Blue LED
Br
CO2Me
Ph
CO2Me

ee = 91%

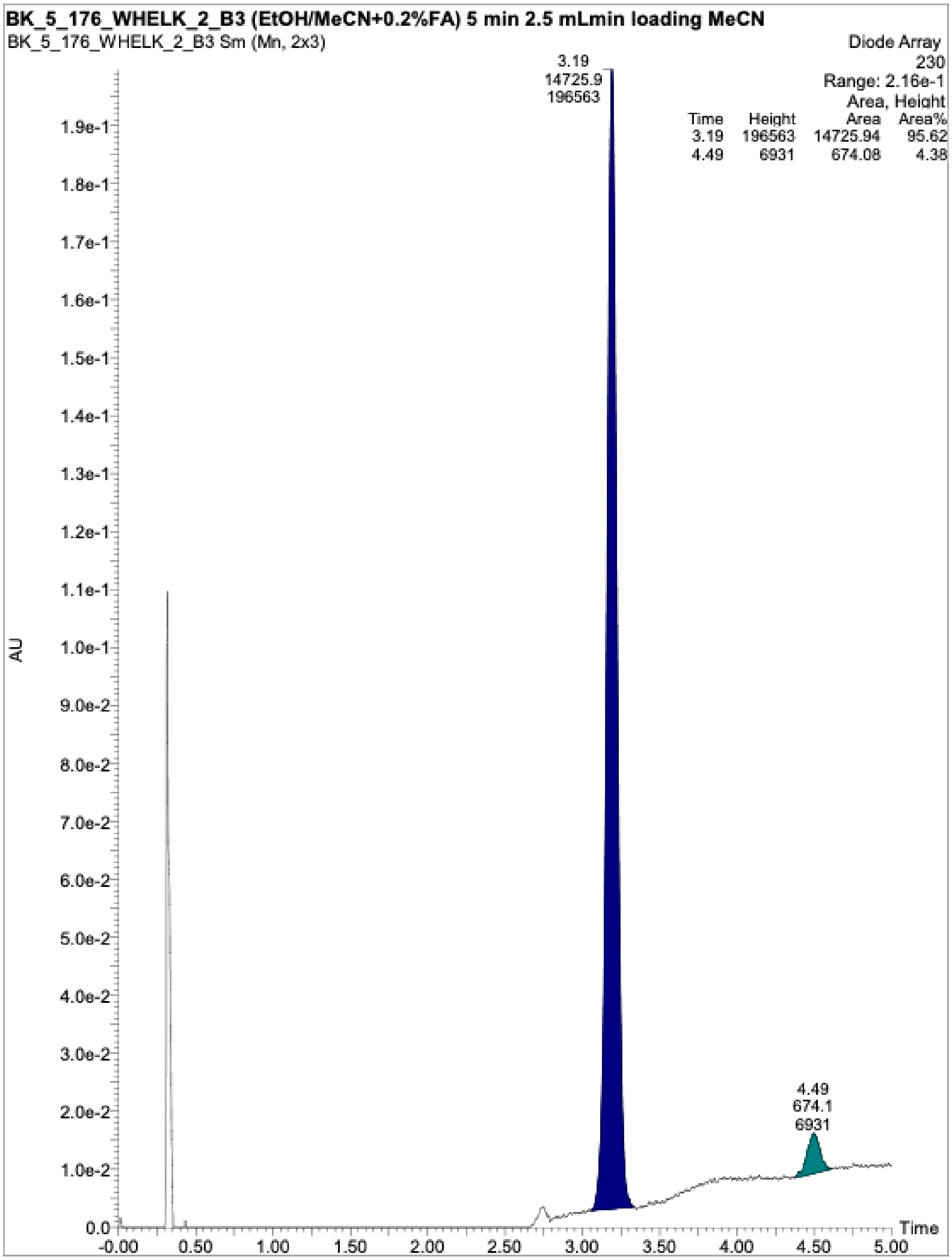

BK_5_176_WHELK_2_B3 (EtOH/MeCN+0.2%FA) 5 min 2.5 mLmin loading MeCN
BK_5_176_WHELK_2_B3 Sm (Mn, 2x3)
Diode Array
230
Range: 2.16e-1
Area, Height
Time Height Area Area%
3.19 196563 14725.94 95.62
4.49 6931 674.08 4.38
3.19
14725.9
196563
4.49
674.1
6931
AU
Time

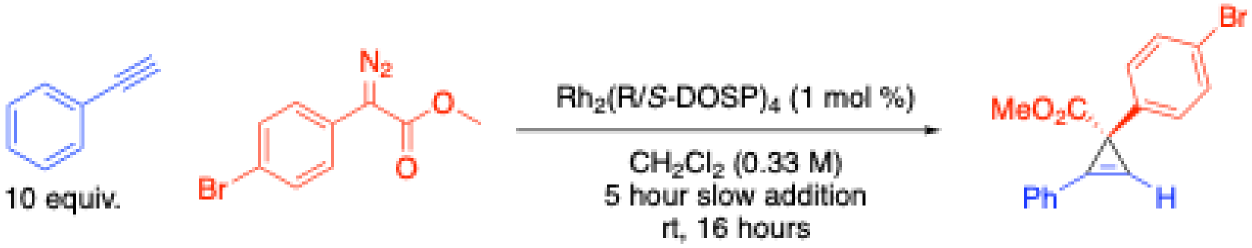
10 equiv.
Rh2(R/S-DOSP)4 (1 mol %)
CH2Cl2 (0.33 M)
5 hour slow addition
rt, 16 hours

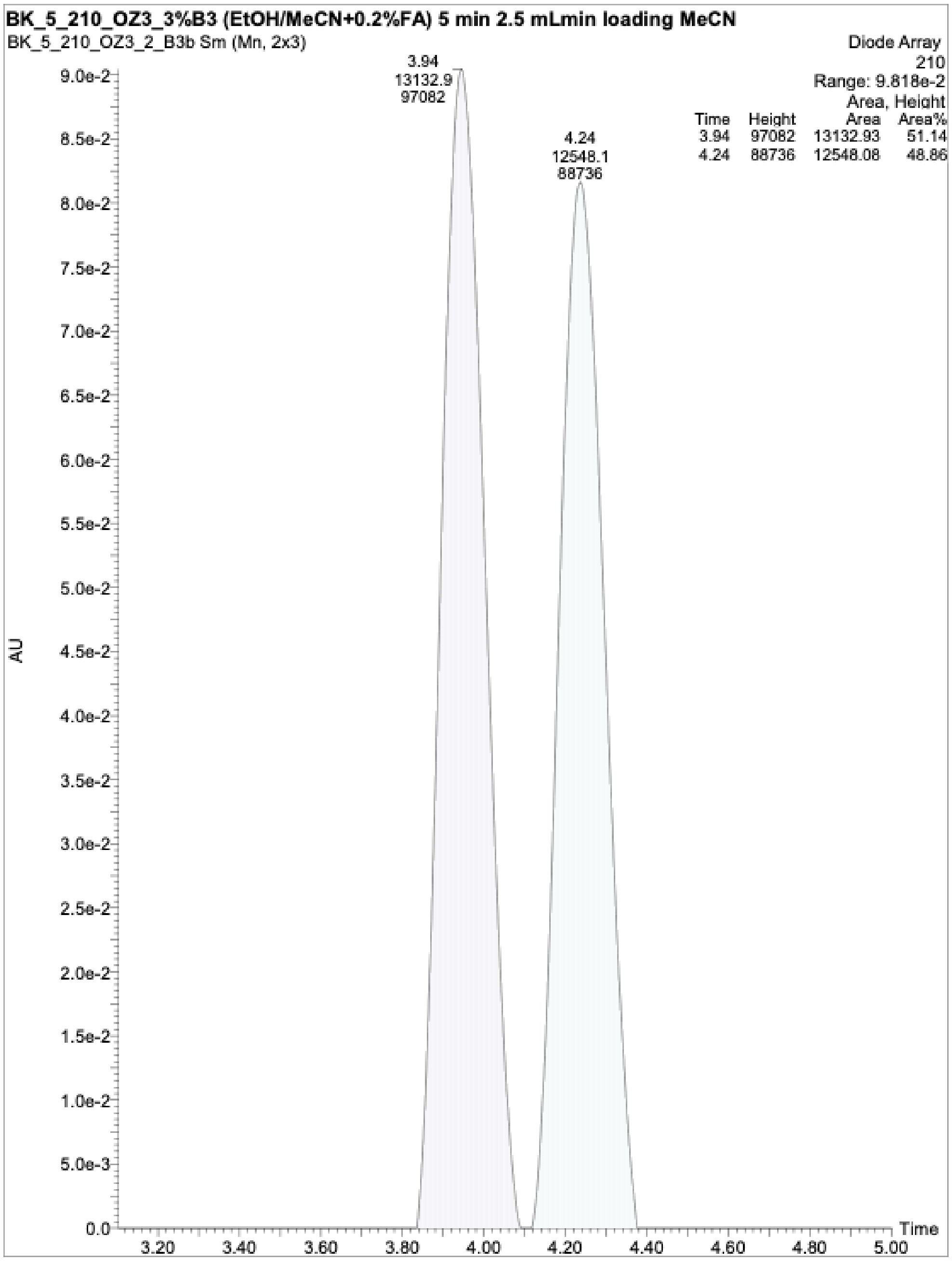
BK_5_210_OZ3_3%B3 (EtOH/MeCN+0.2%FA) 5 min 2.5 mLmin loading MeCN
BK_5_210_OZ3_2_B3b Sm (Mn, 2x3)
Diode Array
210
Range: 9.818e-2
Area, Height
Time Height Area Area%
3.94 97082 13132.93 51.14
4.24 88736 12548.08 48.86
AU
Time

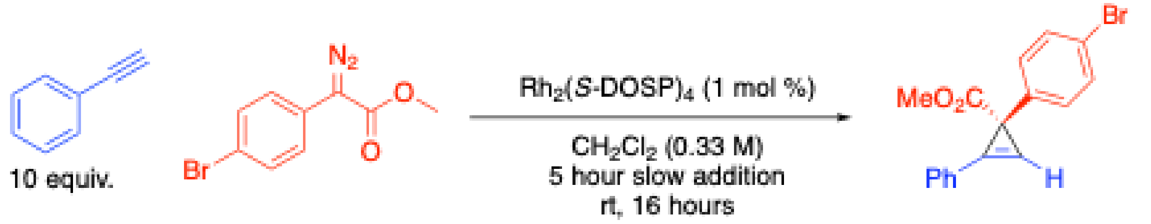

ee = 92%

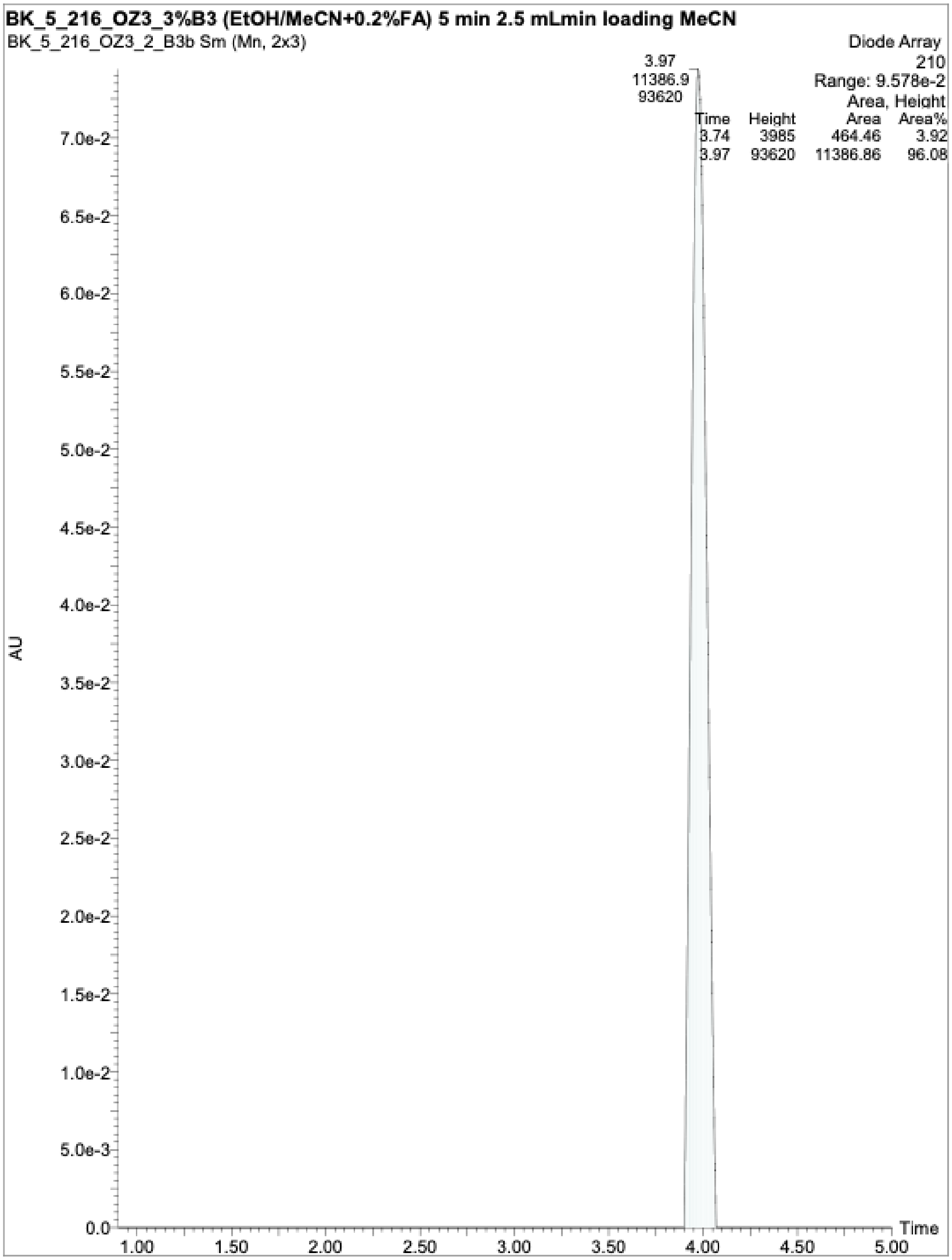

Time	Height	Area	Area%
3.74	3985	464.46	3.92
3.97	93620	11386.86	96.08

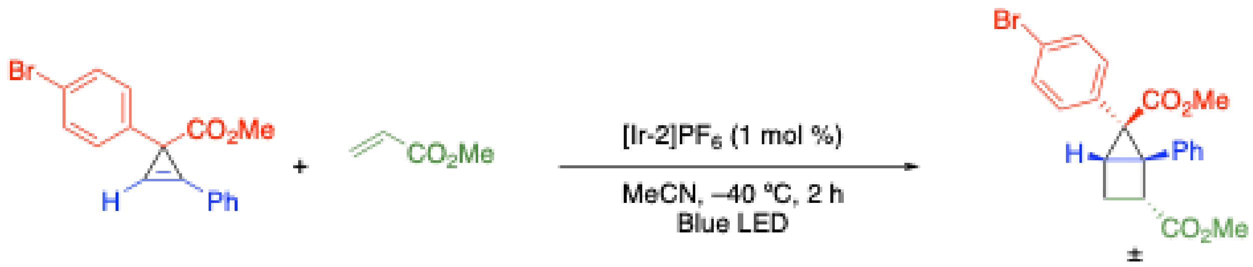
Br
CO2Me
H
Ph
+
CO2Me
[Ir-2]PF6 (1 mol %)
MeCN, –40 °C, 2 h
Blue LED
Br
CO2Me
H
Ph
CO2Me
±

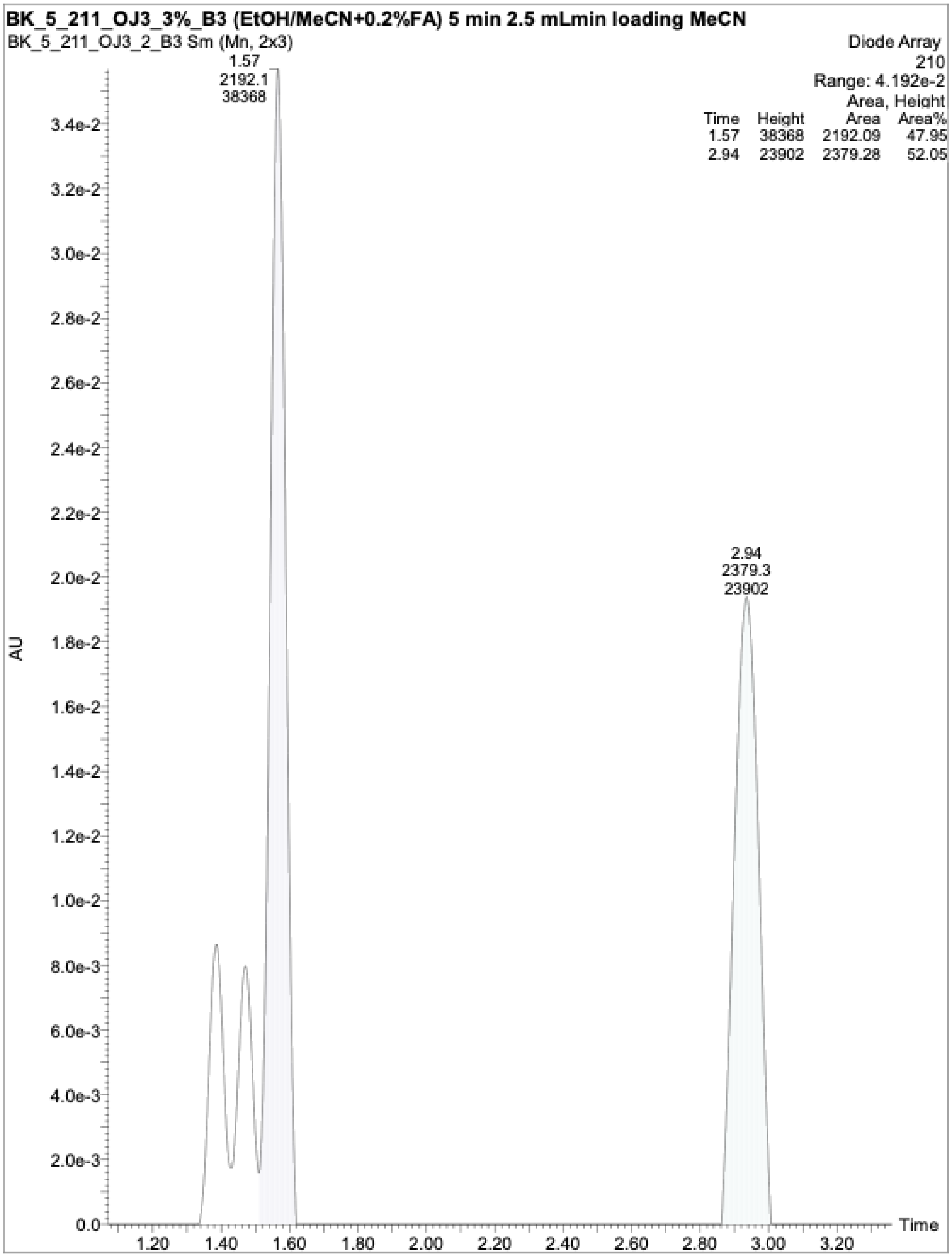
BK_5_211_OJ3_3%_B3 (EtOH/MeCN+0.2%FA) 5 min 2.5 mLmin loading MeCN
BK_5_211_OJ3_2_B3 Sm (Mn, 2x3)
Diode Array
210
Range: 4.192e-2
Area, Height
Time Height Area Area%
1.57 38368 2192.09 47.95
2.94 23902 2379.28 52.05
1.57
2192.1
38368
2.94
2379.3
23902
AU
Time

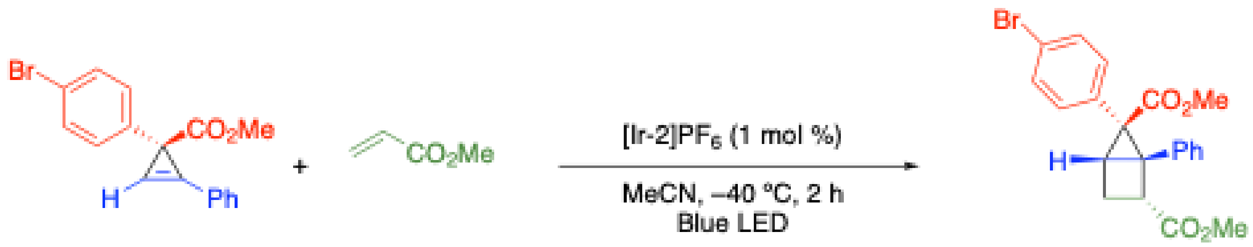
Br
CO2Me
H
Ph
+
CO2Me
[Ir-2]PF6 (1 mol %)
MeCN, –40 °C, 2 h
Blue LED
Br
CO2Me
H
Ph
CO2Me

ee = 90%

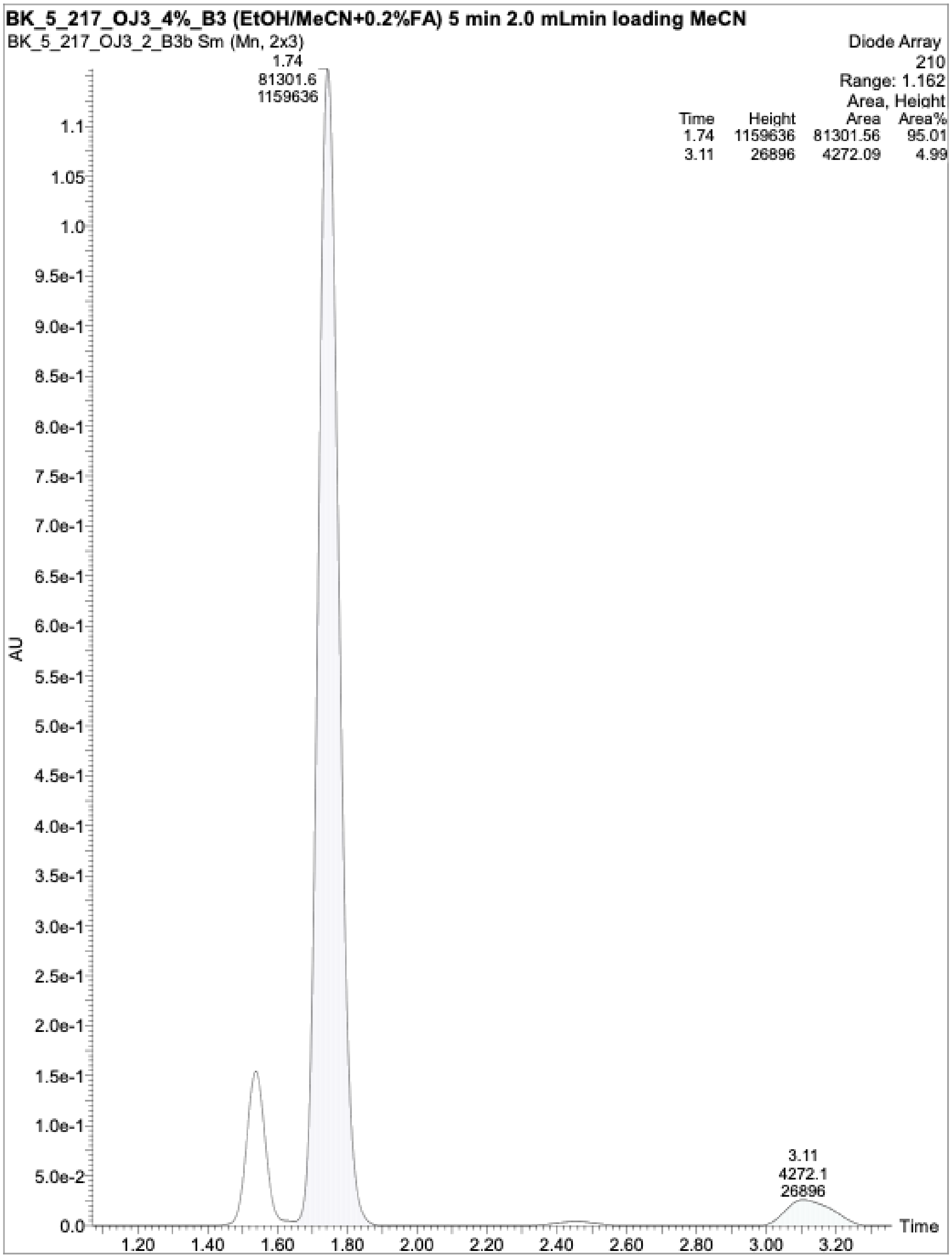
BK_5_217_OJ3_4%_B3 (EtOH/MeCN+0.2%FA) 5 min 2.0 mLmin loading MeCN
BK_5_217_OJ3_2_B3b Sm (Mn, 2x3)
Diode Array
210
Range: 1.162
Area, Height
Time Height Area Area%
1.74 1159636 81301.56 95.01
3.11 26896 4272.09 4.99
1.74
81301.6
1159636
3.11
4272.1
26896
AU
Time

X. X-ray Crystallography Data

HB963-BK-5-114

EMORY UNIVERSITY | X-ray Crystallography Center

R_1=3.93%

Submitted by: **Brockton Keen, Davies Lab**

Solved by: **Michelle Lee, John Bacsa**

Crystal Data and Experimental

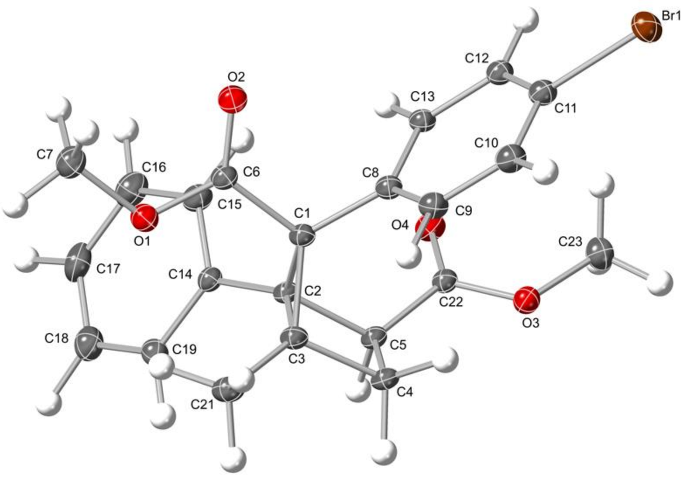

Experimental. Single colorless prism-shaped crystals of HB963-BK-5-114 were crystallised from heptane by slow cooling. A suitable crystal with dimensions 0.12 × 0.10 × 0.06 mm^3 was selected and mounted on a loop with paratone on a XtaLAB Synergy, Dualflex, HyPix diffractometer. The crystal was kept at a steady T = 100.00(10) K during data collection. The structure was solved with the ShelXT (Sheldrick, 2015) solution program using dual methods and by using Olex2 1.5-alpha (Dolomanov et al., 2009) as the graphical interface. The model was refined with olex2.refine 1.5-alpha (Bourhis et al., 2015) using full matrix least squares minimisation on $\boldsymbol{F^2}$.

Crystal Data. $C_{22}H_{21}BrO_4$, M_r = 429.313, triclinic, P-1 (No. 2), a = 6.9804(4) Å, b = 11.4395(8) Å, c = 12.9653(10) Å, α = 69.942(7)°, β = 89.772(6)°, γ = 87.756(5)°, V = 971.72(12) Å^3, T = 100.00(10) K, Z = 2, Z' = 1, μ(Mo K_α) = 2.140, 22003 reflections measured, 6580 unique (R_{int} = 0.0465) which were used in all calculations. The final wR_2 was 0.0578 (all data) and R_1 was 0.0393 (I≥2 σ(I)).

**Compound**	HB963-BK-5-114
Formula	$C_{22}H_{21}BrO_4$
$D_{calc.}$/ g cm^{-3}	1.467
μ/mm^{-1}	2.140
Formula Weight	429.313
Colour	colorless
Shape	prism-shaped
Size/mm^3	0.12×0.10×0.06
T/K	100.00(10)
Crystal System	triclinic
Space Group	P-1
a/Å	6.9804(4)
b/Å	11.4395(8)
c/Å	12.9653(10)
α/°	69.942(7)
β/°	89.772(6)
γ/°	87.756(5)
V/Å^3	971.72(12)
Z	2
Z'	1
Wavelength/Å	0.71073
Radiation type	Mo K_α
Θ_{min}/°	2.93
Θ_{max}/°	33.02
Measured Refl's.	22003
Indep't Refl's	6580
Refl's I≥2 σ(I)	4828
R_{int}	0.0465
Parameters	433
Restraints	384
Largest Peak	0.6818
Deepest Hole	-0.6628
GooF	1.0208
wR_2 (all data)	0.0578
wR_2	0.0527
R_1 (all data)	0.0697
R_1	0.0393

Structure Quality Indicators

**Reflections:**	d min (MoKα) 2Θ=66.0° 0.65	I/σ(I) 15.7	Rint m=3.34 4.65%	Full 50.5° 90% to 66.0° 99.3
**Refinement:**	Shift 0.001	Max Peak 0.7	Min Peak -0.7	GooF 1.021

A colorless prism-shaped-shaped crystal with dimensions 0.12 × 0.10 × 0.06 mm^3 was mounted on a loop with paratone. Data were collected using a XtaLAB Synergy, Dualflex, HyPix diffractometer equipped with an Oxford Cryosystems low-temperature device operating at T = 100.00(10) K.

Data were measured using ω scans with Mo K_α radiation. The diffraction pattern was indexed and the total number of runs and images was based on the strategy calculation from the program CrysAlisPro 1.171.42.89a (Rigaku OD, 2023). The maximum resolution that was achieved was Θ = 33.02° (0.65 Å).

The unit cell was refined using CrysAlisPro 1.171.42.89a (Rigaku OD, 2023) on 3110 reflections, 14% of the observed reflections.

Data reduction, scaling and absorption corrections were performed using CrysAlisPro 1.171.42.89a (Rigaku OD, 2023). The final completeness is 99.26 % out to 33.02° in Θ. A numerical absorption correction based on gaussian integration over a multifaceted crystal model was performed using CrysAlisPro 1.171.42.74a (Rigaku Oxford Diffraction, 2022). An empirical absorption correction using spherical harmonics, implemented in SCALE3 ABSPACK scaling algorithm was also applied. The absorption coefficient μ of this material is 2.140 mm^{-1} at this wavelength (λ = 0.71073Å) and the minimum and maximum transmissions are 0.836 and 1.000.

The structure was solved and the space group P-1 (# 2) determined by the ShelXT (Sheldrick, 2015) structure solution program using using dual methods and refined by full matrix least squares minimisation on $\boldsymbol{F}^2$ using version of olex2.refine 1.5-alpha (Bourhis et al., 2015). All atoms, even hydrogen atoms, were refined anisotropically. Hydrogen atom positions were located from the electron densities and freely refined using Hirshfeld scattering factors. Refinement was by using NoSpherA2, an implementation of non-spherical atom-form-factors (F. Kleemiss, H. Puschmann, O. Dolomanov, S.Grabowsky - https://doi.org/10.1039/D0SC05526C – 2020). NoSpherA2 implementation of HAR makes use of tailor-made aspherical atomic form factors calculated from a Hirshfeld-partitioned electron density (ED) not from spherical-atom form factors. The ED was calculated from a Gaussian basis set single determinant SCF wavefunction from DFT using selected functionals for a fragment of this crystal. This fragment was embedded in an electrostatic crystal field by employing cluster charges. The following options were used:SOFTWARE: ORCA PARTITIONING: NoSpherA2 INT ACCURACY: Normal METHOD: PBE BASIS SET: def2-TZVP CHARGE: 0 MULTIPLICITY: 1 SOLVATION: Hexane DATE: 2023-10-19_12-22-21

There is a single formula unit in the asymmetric unit, which is represented by the reported sum formula. In other words: Z is 2 and Z' is 1. The moiety formula is C22 H21 Br O4.

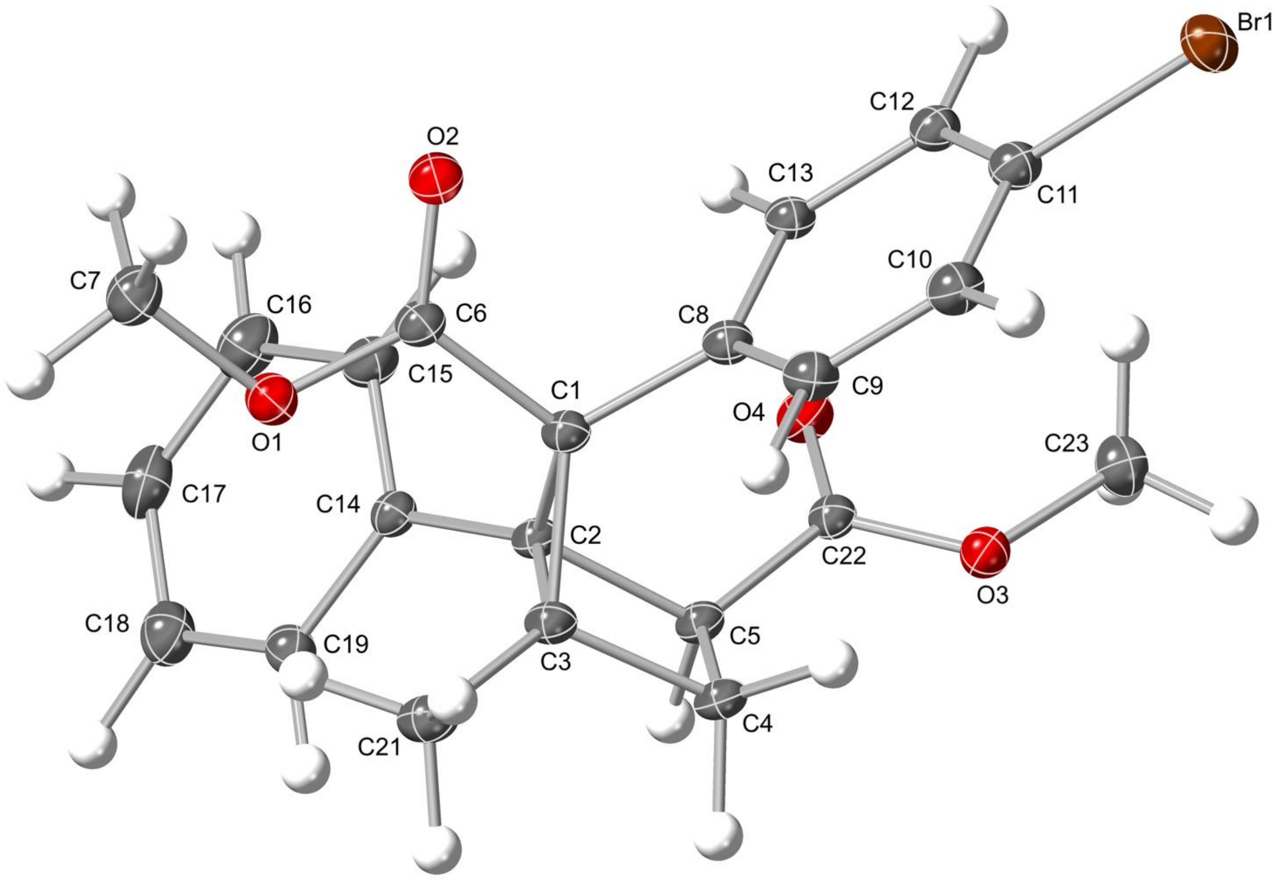

Figure 1 The molecular structure depicted using thermal ellipsoids (50% probability level for non-hydrogen atoms). Although there are chiral atoms in this structure; the crystals were racemic.

Data Plots: Diffraction Data

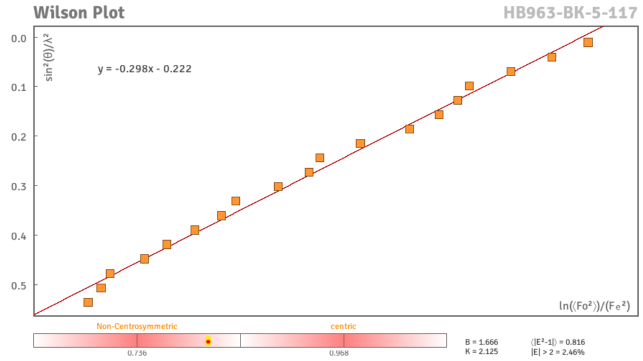

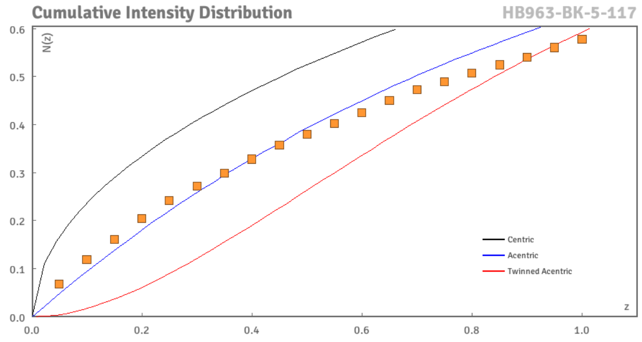

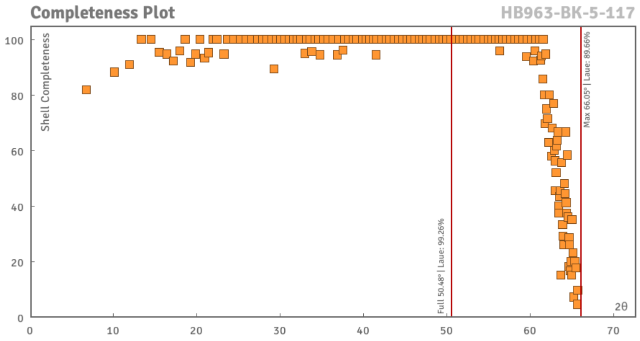

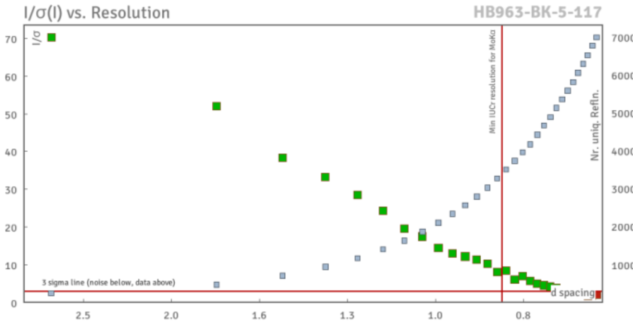

Data Plots: Refinement and Data

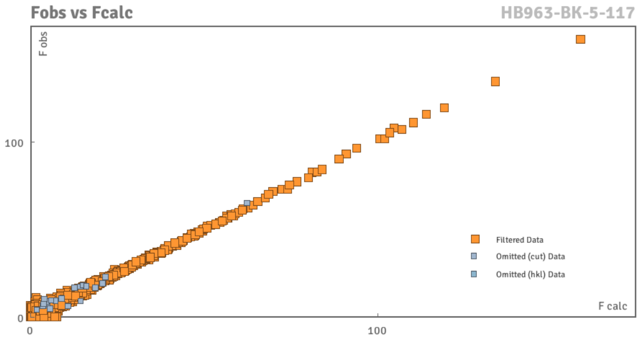

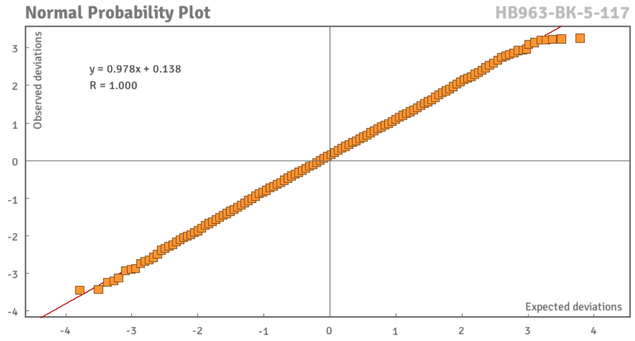

Reflection Statistics

Total reflections (after filtering)	22003	Unique reflections	6580
Completeness	0.897	Mean I/σ	15.15
hkl_{max} collected	(10, 16, 19)	hkl_{min} collected	(-10, -17, -19)
hkl_{max} used	(10, 17, 19)	hkl_{min} used	(-10, -16, 0)
Lim d_{max} collected	100.0	Lim d_{min} collected	0.36
d_{max} used	6.96	d_{min} used	0.65
Friedel pairs	5002	Friedel pairs merged	1
Inconsistent equivalents	1	R_{int}	0.0465
R_{sigma}	0.0636	Intensity transformed	0
Omitted reflections	0	Omitted by user (OMIT hkl)	129
Multiplicity	(5546, 3435, 1464, 776, 263, 87, 22, 9, 2, 1)	Maximum multiplicity	13
Removed systematic absences	0	Filtered off (Shel/OMIT)	0

Table 1: Fractional Atomic Coordinates (×10^4) and Equivalent Isotropic Displacement Parameters (Å^2×10^3) for HB963-BK-5-114. U_{eq} is defined as 1/3 of the trace of the orthogonalised U_{ij}.

Atom	x	y	z	U_{eq}
Br1	2272.3(2)	929.10(14)	4016.83(13)	24.85(5)
O1	5437.9(14)	7842.8(9)	421.5(8)	21.6(2)
O2	3124.3(15)	6631.3(10)	1296.0(10)	38.2(3)
O3	8959.3(14)	3326.5(8)	4300.7(7)	18.2(2)
O4	7825.6(14)	4979.5(9)	4708.9(7)	18.9(2)
C1	6332.2(19)	5844.6(12)	1746.9(10)	13.9(3)
C2	7985.6(19)	6369.4(12)	2217.7(10)	14.0(3)
C3	8342.5(19)	5896.1(12)	1259.5(10)	15.2(3)
C4	9551(2)	4773.8(13)	2014.9(11)	15.9(3)
C5	9288.2(19)	5302.5(12)	2960.4(10)	14.2(3)

Atom	x	y	z	U_{eq}
C6	4804(2)	6795.3(13)	1141.9(11)	17.7(3)
C7	3966(2)	8728.8(15)	-168.3(14)	25.4(3)
C8	5429.9(19)	4625.8(12)	2344.7(11)	15.0(3)
C9	5385(2)	3717.7(13)	1852.2(12)	18.1(3)
C10	4465(2)	2608.5(13)	2349.1(12)	19.7(3)
C11	3572(2)	2424.5(12)	3351.5(11)	17.5(3)
C12	3576(2)	3312.2(12)	3863.0(11)	16.1(3)
C13	4509.8(19)	4416.0(12)	3343.6(11)	14.7(3)
C14	7945.6(19)	7632.3(12)	2304.3(10)	14.4(3)
C15	6548(2)	7995.1(13)	2917.7(11)	20.2(3)
C16	6646(2)	9128.7(14)	3089.0(12)	25.6(3)
C17	8143(2)	9908.1(13)	2657.3(12)	26.0(3)
C18	9525(2)	9564.0(13)	2025.8(13)	25.5(3)
C19	9433(2)	8428.6(13)	1849.1(12)	20.0(3)
C21	8882(2)	6541.6(15)	82.5(12)	21.5(3)
C22	8583.7(19)	4542.7(12)	4076.5(11)	14.2(3)
C23	8445(3)	2527.0(14)	5375.6(13)	24.8(3)

Table 2: Anisotropic Displacement Parameters (×10[4]) for HB963-BK-5-114. The anisotropic displacement factor exponent takes the form: $-2\pi^2[h^2a^{*2} \times U_{11} + ... +2hka^* \times b^* \times U_{12}]$

Atom	U_{11}	U_{22}	U_{33}	U_{23}	U_{13}	U_{12}
Br1	25.03(9)	17.92(7)	33.77(9)	-7.12(6)	6.25(6)	-11.07(6)
O1	16.8(5)	22.1(4)	21.0(5)	-1.0(3)	-1.7(3)	-1.1(3)
O2	12.5(4)	27.7(6)	55.5(7)	0.8(3)	-0.6(3)	9.8(5)
O3	18.5(5)	17.4(4)	18.2(5)	0.8(4)	0.3(4)	-5.6(4)
O4	21.1(6)	22.1(5)	15.3(5)	2.2(4)	0.4(4)	-9.1(4)
C1	11.5(5)	17.9(5)	12.1(6)	-0.2(3)	-1.9(4)	-5.0(4)
C2	10.8(7)	17.0(6)	14.3(6)	0.7(4)	-0.6(5)	-5.5(5)
C3	13.0(7)	20.1(6)	13.0(6)	0.0(5)	0.3(5)	-6.4(5)
C4	11.7(7)	20.8(7)	16.0(7)	2.5(5)	-0.6(5)	-7.7(5)
H4a	13(2)	29(7)	25(7)	2.0(15)	1.9(14)	-12(4)
H4b	27(7)	22(3)	28(7)	-1.6(18)	-5(4)	-8.5(18)
C5	11.4(7)	17.0(6)	14.4(6)	1.4(5)	-1.6(5)	-5.7(5)
H5	12(3)	23(6)	18(6)	-1(2)	-1.0(19)	-9(3)
C6	12.3(4)	20.0(4)	18.0(6)	0.5(3)	-2.9(3)	-2.8(3)
C7	21.2(7)	24.7(6)	25.6(8)	1.9(4)	-5.4(5)	-2.9(5)
H7a	34(6)	39(7)	36(7)	-5(3)	-9(3)	-9(3)
H7b	42(7)	33(5)	33(5)	-7(2)	2(2)	-4(2)
H7c	38(7)	40(7)	34(6)	10(3)	-1(3)	-9(3)
C8	13.0(7)	17.5(6)	15.4(6)	-0.6(5)	-0.6(5)	-6.9(5)
C9	18.0(8)	20.6(7)	18.3(7)	-1.7(5)	1.0(6)	-9.8(5)
H9	49(10)	43(8)	30(4)	-17(5)	20(3)	-21(3)
C10	19.0(8)	20.4(7)	23.1(7)	-0.9(5)	-0.6(5)	-12.0(5)
H10	50(12)	28(5)	29(6)	-16(4)	15(5)	-18(3)
C11	15.0(7)	17.5(6)	21.9(7)	-1.7(5)	-0.4(5)	-9.1(5)
C12	13.5(7)	16.6(6)	19.4(7)	-1.0(5)	-0.7(5)	-7.5(5)
H12	35(9)	26(7)	26(4)	-13(4)	12(3)	-15(3)
C13	11.9(7)	16.9(6)	16.6(7)	-1.2(5)	0.0(5)	-7.2(5)
H13	19(9)	22(4)	25(6)	-1(3)	0(4)	-14(2)
C14	13.2(7)	15.1(6)	14.4(6)	1.0(4)	-2.5(5)	-4.6(5)
C15	19.2(8)	20.9(7)	22.3(7)	1.8(5)	1.7(6)	-9.8(5)
H15	42(6)	39(6)	58(10)	-18(3)	30(4)	-34(4)
C16	30.9(9)	23.9(7)	25.8(8)	4.9(6)	-1.4(6)	-14.0(6)
H16	73(8)	43(8)	94(11)	-21(4)	51(4)	-49(4)
C17	32.8(9)	16.3(7)	28.5(8)	4.0(6)	-10.1(6)	-7.4(6)

Atom	U_{11}	U_{22}	U_{33}	U_{23}	U_{13}	U_{12}
H17	53(10)	33(4)	84(11)	-11(3)	16(6)	-37(3)
C18	23.3(9)	19.0(7)	32.8(8)	-2.4(5)	-6.1(6)	-6.8(6)
H18	37(5)	32(7)	69(11)	-15(3)	14(4)	-26(4)
C19	16.1(8)	18.5(6)	23.1(7)	-2.3(5)	-0.8(6)	-4.1(5)
H19	35(6)	37(8)	62(9)	-11(3)	25(4)	-25(4)
C21	16.8(8)	29.4(8)	16.9(7)	2.1(6)	0.8(6)	-6.5(6)
H21a	18(2)	48(8)	38(7)	2.0(13)	3.6(13)	-13(4)
H21b	31(7)	43(6)	27(5)	-1(3)	-3(3)	-15(3)
H21c	38(8)	34(3)	51(7)	8.6(17)	1(3)	-6.8(16)
C22	12.6(7)	16.7(6)	13.2(6)	0.5(5)	-2.0(5)	-5.0(5)
C23	27.2(10)	21.7(7)	21.5(8)	-0.7(6)	1.4(6)	-2.4(6)
H23a	54(8)	49(7)	34(5)	-9(3)	-4(3)	-15(3)
H23b	29(2)	61(8)	35(7)	-4.5(13)	3.9(13)	-13(4)
H23c	56(8)	30(3)	68(8)	6.9(19)	4(4)	-13.1(19)

Table 3: Bond Lengths in Å for HB963-BK-5-114.

Atom	Atom	Length/Å
Br1	C11	1.8964(13)
O1	C6	1.3321(15)
O1	C7	1.4356(17)
O2	C6	1.1998(17)
O3	C22	1.3355(15)
O3	C23	1.4336(17)
O4	C22	1.2078(16)
C1	C2	1.5416(17)
C1	C3	1.5316(19)
C1	C6	1.5055(18)
C1	C8	1.5050(17)
C2	C3	1.5320(18)
C2	C5	1.5355(18)
C2	C14	1.4862(18)
C3	C4	1.5423(18)
C3	C21	1.5033(19)
C4	H4a	1.088(14)
C4	H4b	1.107(14)
C4	C5	1.5492(19)
C5	H5	1.096(13)
C5	C22	1.5020(18)
C7	H7a	1.061(16)
C7	H7b	1.054(15)
C7	H7c	1.069(17)
C8	C9	1.3948(18)
C8	C13	1.3928(19)
C9	H9	1.035(14)
C9	C10	1.3919(18)
C10	H10	1.110(15)
C10	C11	1.392(2)
C11	C12	1.3905(19)
C12	H12	1.097(14)
C12	C13	1.3966(18)
C13	H13	1.114(14)
C14	C15	1.3944(19)
C14	C19	1.3987(18)
C15	H15	1.106(16)
C15	C16	1.393(2)
C16	H16	1.039(17)
C16	C17	1.388(2)
C17	H17	1.082(16)
C17	C18	1.393(2)
C18	H18	1.109(16)
C18	C19	1.398(2)
C19	H19	1.077(16)
C21	H21a	1.054(16)
C21	H21b	1.104(16)
C21	H21c	1.063(17)
C23	H23a	1.038(16)
C23	H23b	1.049(17)
C23	H23c	1.062(17)

Table 4: Bond Angles in ° for HB963-BK-5-114.

Atom	Atom	Atom	Angle/°
C7	O1	C6	114.96(11)
C23	O3	C22	115.98(11)
C3	C1	C2	59.80(8)
C6	C1	C2	114.78(11)
C6	C1	C3	119.44(11)
C8	C1	C2	124.05(11)
C8	C1	C3	121.66(11)
C8	C1	C6	109.55(11)
C3	C2	C1	59.78(8)
C5	C2	C1	110.27(10)
C5	C2	C3	90.06(10)
C14	C2	C1	124.40(12)

Atom	Atom	Atom	Angle/°
C14	C2	C3	132.92(11)
C14	C2	C5	121.65(11)
C2	C3	C1	60.42(8)
C4	C3	C1	107.81(11)
C4	C3	C2	90.49(10)
C21	C3	C1	126.74(12)
C21	C3	C2	132.44(12)
C21	C3	C4	121.26(12)
H4a	C4	C3	112.7(7)
H4b	C4	C3	118.0(8)
H4b	C4	H4a	107.6(11)
C5	C4	C3	89.17(10)
C5	C4	H4a	110.7(7)
C5	C4	H4b	117.8(8)
C4	C5	C2	90.10(10)
H5	C5	C2	108.5(7)
H5	C5	C4	111.3(7)
C22	C5	C2	118.78(11)
C22	C5	C4	122.91(11)
C22	C5	H5	104.5(7)
O2	C6	O1	121.77(13)
C1	C6	O1	115.53(12)
C1	C6	O2	122.70(12)
H7a	C7	O1	110.0(9)
H7b	C7	O1	106.4(9)
H7b	C7	H7a	109.9(12)
H7c	C7	O1	111.6(8)
H7c	C7	H7a	107.6(13)
H7c	C7	H7b	111.4(13)
C9	C8	C1	119.42(12)
C13	C8	C1	121.51(12)
C13	C8	C9	118.89(12)
H9	C9	C8	118.3(8)
C10	C9	C8	121.16(14)
C10	C9	H9	120.5(9)
H10	C10	C9	121.3(8)
C11	C10	C9	118.48(13)
C11	C10	H10	120.2(8)
C10	C11	Br1	118.61(10)
C12	C11	Br1	119.41(10)

Atom	Atom	Atom	Angle/°
C12	C11	C10	121.96(13)
H12	C12	C11	120.7(8)
C13	C12	C11	118.21(13)
C13	C12	H12	121.1(8)
C12	C13	C8	121.29(13)
H13	C13	C8	119.7(7)
H13	C13	C12	119.0(7)
C15	C14	C2	121.09(12)
C19	C14	C2	119.43(13)
C19	C14	C15	119.21(13)
H15	C15	C14	119.7(8)
C16	C15	C14	120.47(14)
C16	C15	H15	119.9(8)
H16	C16	C15	120.0(9)
C17	C16	C15	120.39(15)
C17	C16	H16	119.5(9)
H17	C17	C16	123.0(9)
C18	C17	C16	119.52(14)
C18	C17	H17	117.5(9)
H18	C18	C17	119.2(9)
C19	C18	C17	120.33(14)
C19	C18	H18	120.3(9)
C18	C19	C14	120.06(14)
H19	C19	C14	121.1(9)
H19	C19	C18	118.8(9)
H21a	C21	C3	111.7(8)
H21b	C21	C3	111.0(8)
H21b	C21	H21a	106.3(12)
H21c	C21	C3	111.4(9)
H21c	C21	H21a	110.9(13)
H21c	C21	H21b	105.3(12)
O4	C22	O3	124.03(12)
C5	C22	O3	111.73(11)
C5	C22	O4	124.20(12)
H23a	C23	O3	111.3(9)
H23b	C23	O3	112.7(9)
H23b	C23	H23a	109.8(14)
H23c	C23	O3	106.7(10)
H23c	C23	H23a	108.5(14)
H23c	C23	H23b	107.6(14)

Table 5: Torsion Angles in ° for HB963-BK-5-114.

Atom	Atom	Atom	Atom	Angle/°
Br1	C11	C10	C9	178.74(11)
Br1	C11	C12	C13	-178.68(10)
O1	C6	C1	C2	46.83(13)
O1	C6	C1	C3	-21.11(14)
O1	C6	C1	C8	-168.25(12)
O2	C6	C1	C2	-132.85(15)
O2	C6	C1	C3	159.21(14)
O2	C6	C1	C8	12.07(16)
O3	C22	C5	C2	136.43(10)
O3	C22	C5	C4	25.47(13)
O4	C22	C5	C2	-45.79(15)
O4	C22	C5	C4	-156.76(13)

Atom	Atom	Atom	Atom	Angle/˚
C1	C2	C3	C4	-110.29(9)
C1	C2	C3	C21	114.39(11)
C1	C2	C5	C4	54.28(11)
C1	C2	C5	C22	-74.10(12)
C1	C2	C14	C15	59.52(14)
C1	C2	C14	C19	-126.45(13)
C1	C3	C2	C5	113.60(9)
C1	C3	C2	C14	-110.56(10)
C1	C3	C4	C5	-62.23(11)
C1	C8	C9	C10	-176.19(12)
C1	C8	C13	C12	176.13(12)
C2	C3	C4	C5	-3.28(10)
C2	C5	C4	C3	3.27(10)
C2	C14	C15	C16	173.13(13)
C2	C14	C19	C18	-173.09(12)
C3	C4	C5	C22	128.34(9)
C8	C9	C10	C11	0.44(16)
C8	C13	C12	C11	-0.53(15)
C9	C10	C11	C12	0.00(16)
C10	C11	C12	C13	0.04(16)
C14	C15	C16	C17	-0.45(16)
C14	C19	C18	C17	0.16(16)
C15	C16	C17	C18	1.67(17)
C16	C17	C18	C19	-1.53(17)

Table 6: Hydrogen Fractional Atomic Coordinates (×10[4]) and Equivalent Isotropic Displacement Parameters (Å[2]×10[3]) for HB963-BK-5-114. U_{eq} is defined as 1/3 of the trace of the orthogonalised U_{ij}.

Atom	x	y	z	U_{eq}
H4a	11050(20)	4787(13)	1773(11)	21(3)
H4b	9050(20)	3828(14)	2147(11)	26(3)
H5	10610(20)	5694(12)	3125(11)	17(3)
H7a	3000(20)	8296(14)	-538(12)	37(4)
H7b	4660(20)	9438(14)	-780(12)	37(3)
H7c	3160(20)	9094(15)	364(12)	39(4)
H9	6090(20)	3877(14)	1116(12)	38(5)
H10	4400(20)	1895(14)	1949(12)	33(4)
H12	2840(20)	3155(13)	4645(12)	27(4)
H13	4510(20)	5141(13)	3738(11)	21(4)
H15	5370(20)	7370(15)	3283(13)	42(5)
H16	5650(30)	9365(16)	3591(16)	63(6)
H17	8280(30)	10794(15)	2772(15)	52(5)
H18	10760(20)	10165(15)	1720(14)	44(5)
H19	10530(20)	8177(15)	1370(14)	42(5)
H21a	10380(20)	6502(15)	-26(12)	35(4)
H21b	8250(20)	6090(15)	-457(12)	33(4)
H21c	8330(20)	7478(16)	-215(13)	43(4)
H23a	9040(30)	2808(16)	5981(13)	45(4)
H23b	6960(30)	2465(16)	5479(12)	43(4)
H23c	9020(30)	1623(16)	5467(14)	53(4)

Citations

CrysAlisPro Software System, Rigaku Oxford Diffraction, (2023).

L.J. Bourhis and O.V. Dolomanov and R.J. Gildea and J.A.K. Howard and H. Puschmann, The Anatomy of a Comprehensive Constrained, Restrained, Refinement Program for the Modern Computing Environment - Olex2 Disected, *Acta Cryst. A*, (2015), **A71**, 59-71.

O.V. Dolomanov and L.J. Bourhis and R.J. Gildea and J.A.K. Howard and H. Puschmann, Olex2: A complete structure solution, refinement and analysis program, *J. Appl. Cryst.*, (2009), **42**, 339-341.

Sheldrick, G.M., ShelXT-Integrated space-group and crystal-structure determination, *Acta Cryst.*, (2015), **A71**, 3-8.
